# Wasserstein F-tests for Fréchet regression on Bures-Wasserstein manifolds

**Published:** 2025

**Authors:** Haoshu Xu, Hongzhe Li

**Affiliations:** Graduate Group in Applied Mathematics and Computational Science, University of Pennsylvania, Philadelphia, PA 19104, USA; Department of Biostatistics, Epidemiology and Informatics, University of Pennsylvania, Philadelphia, PA 19104, USA

**Keywords:** Fréchet regression, Functional calculus, Hypothesis testing, Optimal transport, Wasserstein distance

## Abstract

This paper addresses regression analysis for covariance matrix-valued outcomes with Euclidean covariates, motivated by applications in single-cell genomics and neuroscience where covariance matrices are observed across many samples. Our analysis leverages Fréchet regression on the Bures-Wasserstein manifold to estimate the conditional Fréchet mean given covariates x. We establish a non-asymptotic uniform $\sqrt{n}$-rate of convergence (up to logarithmic factors) over covariates with $\|x\|≲\sqrt{\text{log}\;n}$ and derive a pointwise central limit theorem to enable statistical inference. For testing covariate effects, we devise a novel test whose null distribution converges to a weighted sum of independent chi-square distributions, with power guarantees against a sequence of contiguous alternatives. Simulations validate the accuracy of the asymptotic theory. Finally, we apply our methods to a single-cell gene expression dataset, revealing age-related changes in gene co-expression networks.

## 1 Introduction

In modern data analysis, positive definite matrices frequently arise across various fields, including medical imaging (Dryden et al., 2009; Fillard et al., 2007), neuroscience (Friston, 2011; Kong et al., 2020; Hu et al., 2021), signal processing (Arnaudon et al., 2013), and computer vision (Caseiro et al., 2012). For example, in large-scale single-cell RNA-seq data, individual-specific covariance matrices can be estimated and interpreted as co-expression networks among a set of genes. In neuroimaging, covariance matrices (or correlation matrices after standardization) of multiple brain regions are used to summarize functional connectivity, providing insights into brain network organization. A central challenge in these applications is performing regression analysis where the outcome is a covariance matrix and the covariates are Euclidean.

Several regression models have been proposed for covariance matrix outcomes. Chiu et al. (1996) developed a method that models the elements of the logarithm of the covariance matrix as a linear function of the covariates. Hoff and Niu (2012) proposed a regression model where the covariance matrix is expressed as a quadratic function of the explanatory variables. Zou et al. (2017) linked the matrix outcome to a linear combination of similarity matrices derived from the covariates and examined the asymptotic properties of different estimators under this framework. Zhao et al. (2021) introduced the Covariate Assisted Principal (CAP) regression model for multiple covariance matrix outcomes, focusing on identifying linear projections of the covariance matrices associated with the covariates. Kong et al. (2020) and Hu et al. (2021) developed linear and nonparametric regression methods for high-dimensional, low-rank matrices using nuclear norm regularization. However, these approaches often either impose specific structural assumptions on the covariance matrices or require the estimation of a large number of parameters.

In this paper, we focus on general regression analysis where the responses are symmetric positive-definite (SPD) matrices and the predictors are Euclidean within the framework of the Fréchet regression model(Petersen and Müller, 2019). This model defines a global regression function that links response data in an arbitrary metric space with Euclidean predictors. A key consideration in this context is the choice of an appropriate metric for SPD matrices. Various metrics are available, including the Log-Euclidean metric (Arsigny et al., 2007), the Cholesky metric (Dryden et al., 2009) and the Log-Cholesky metric (Lin, 2019). Each of these metrics has distinct properties and implications for regression analysis. However, the Bures-Wasserstein metric W, introduced by Bures (1969), stands out for its desirable mathematical and geometric properties. It is defined for any pair of SPD matrices $A,B\in {𝒮}_{d}^{++}$ as

(1) $$W(A,B)={\left[\text{tr}\;A+\text{tr}\;B-2\;\text{tr}{\left(A^{1/2}BA^{1/2}\right)}^{1/2}\right]}^{1/2}.$$

This distance possesses desirable properties under both scaling and rotation: for positive scalar c>0 and orthogonal matrix $O\in {𝒪}_{d}$, it satisfies

$$W(cA,cB)=c^{1/2}W(A,B)\;W\left(OAO^{⊤},OBO^{⊤}\right)=W(A,B)$$

Known as the Bures distance in quantum information theory (Bures, 1969), this metric is equivalent to the Wasserstein distance between two centered Gaussian distributions with specified covariance matrices. The Wasserstein distance, a fundamental concept in optimal transport (OT) theory (Villani, 2003, 2009), which blends optimization, analysis, and geometry, has become highly valuable in various statistical and machine learning applications (Abadie and Imbens, 2006; Deb and Sen, 2023; Arjovsky et al., 2017; Redko et al., 2017; Hallin et al., 2021). This equivalence provides the Bures-Wasserstein distance with a clear and meaningful probabilistic interpretation as the minimal “effort” required to transport one Gaussian distribution, defined by its covariance matrix, to another. In single-cell genomics, most measurement technologies are destructive, meaning that the same cell cannot be observed multiple times or profiled over time. Consequently, measurements at each time point are often modeled as distributions, making optimal transport techniques well-suited for analyzing the associated dynamics (Schiebinger et al., 2019; Bunne et al., 2022; Somnath et al., 2023; Bunne et al., 2023a,b). Given its connection to optimal transport theory, the Bures-Wasserstein metric is a natural choice for studying gene expression covariance matrices. When equipped with this metric, the space ${𝒮}_{d}^{++}$ becomes a Riemannian manifold, known as the Bures-Wasserstein manifold (Bhatia et al., 2019).

In contrast, the Log-Euclidean metric, the Cholesky metric and the Log-Cholesky metric lack clear practical interpretations and do not naturally align with the structure of distributions or physical models. In addition, the Log-Euclidean metric $d_{\text{LE}}$ is insensitive to scaling; specifically, $d_{\text{LE}}(cA,cB)=d_{\text{LE}}(A,B)$ for any c>0, making it less suitable for applications where the magnitude of matrices is a critical factor. The Log-Cholesky metric $d_{\text{LC}}$ treats the diagonal and off-diagonal components of the Cholesky factor $L_{A}$ differently, resulting in $d_{\text{LC}}(cA,cB)$ not being proportional to $d_{\text{LC}}(A,B)$ for c>0, which further limits its applicability. Additionally, both the Cholesky metric $d_{\text{C}}$ and the Log-Cholesky metric $d_{\text{LC}}$ lack rotational invariance; specifically, for $O\in {𝒪}_{d}$,

$$d_{\text{C}}\left(OAO^{⊤},OBO^{⊤}\right)\neq d_{\text{C}}\left(A,B\right),\;d_{\text{LC}}\left(OAO^{⊤},OBO^{⊤}\right)\neq d_{\text{LC}}\left(A,B\right).$$

These limitations reduce the practical utility of these metrics for applications involving SPD matrices where both scaling and rotation are essential, such as in aligning data from different sources (Ma et al., 2024).

Using the Bures-Wasserstein distance as the metric for SPD matrices in the Fréchet regression model, we consider independent and identically distributed (i.i.d.) pairs of predictor and response variables $\left(X_{1},Q_{1}\right),\ldots ,\left(X_{n},Q_{n}\right)\in R^{p}\times {𝒮}_{d}^{++}$. The objective is to conduct inference, particularly to test the effect of the covariate X on the response variable Q. Previous research on the Fréchet regression model has primarily focused on consistency in the asymptotic regime (Petersen and Müller, 2019; Chen and Müller, 2022), with inference being considered only in the specific case of one-dimensional (1D) density curves as response variables under the Wasserstein metric (Petersen et al., 2021). Notably, for any pair of 1D distributions μ,ν∈𝒫(R) with distribution functions $F_{\mu },F_{\nu }$, the 2-Wasserstein distance between them is given by ${\left[\int_{0}^{1}{\left|F_{\mu }^{-1}(t)-F_{\nu }^{-1}(t)\right|}^{2}dt\right]}^{1/2}$. Therefore, the 1D Wasserstein space is unique in that it has zero sectional curvature (Ambrosio et al., 2005), and can be *isometrically* embedded into a Hilbert space. This flat geometry allows for closed-form expressions in 1D Fréchet inference and provides well-understood solutions to various problems (Panaretos and Zemel, 2016; Chen et al., 2023; Bigot et al., 2017). However, most metric spaces of interest are nonlinear and exhibit nonzero curvature. For example, the Wasserstein space of distributions in d dimension is positively curved for d>1 (Ambrosio et al., 2005), with the Bures-Wasserstein manifold being a special case. Consequently, *isometric* embeddings of such curved metric spaces into a Hilbert space cannot be assumed, making it difficult to derive distributional results for Fréchet regression in these spaces. This complexity poses additional challenges for statistical inference, particularly in providing guarantees on significance levels and power.

### 1.1 Main contribution

We focus on Fréchet regression on the Bures-Wasserstein manifold, and our main contributions are threefold.

First, we establish a non-asymptotic $\sqrt{n}$-rate of convergence (up to log n factors) for the regression estimate $\hat{Q}(x)$ uniformly over the region $\|x\|≲\sqrt{\text{log}\;n}$. To the best of our knowledge, this is the first non-asymptotic uniform convergence result for Fréchet regression over a potentially diverging region. Beyond the standard assumptions of light tails for X and Q, we only require well-separation and a local curvature lower bound, which are mild conditions and are verified in a simple case. These results are crucial for later deriving the asymptotic null distribution and power of our proposed test for the association between covariance matrices and covariates.

Second, we derive a central limit theorem for the point estimate $\hat{Q}(x)$, leading to the construction of a pointwise confidence region. The covariance operator of the limiting Gaussian distribution is shown to have contributions from two sources: the intrinsic variability in Q and the imperfect information regarding X.

Third, we carefully construct a test statistic with a tractable asymptotic null distribution, which is represented as a weighted sum of $\chi_{p}^{2}$ distributions. The weights are determined by the covariance of the tangent vector, which can be interpreted as a generalization of classical noise variance. The proposed test is also shown to be powerful against a sequence of contiguous alternatives. To the best of our knowledge, this is the first test developed for Fréchet regression on a space with nonzero sectional curvature.

Finally, we validate our theoretical results through numerical simulations and real applications.

### 1.2 Related works

#### Statistical OT

In addition to advances in computational optimal transport (OT) (Cuturi, 2013; Peyré and Cuturi, 2019; Altschuler et al., 2017), there has been a growing interest in the statistical aspects of OT, where the stability of estimated densities (Weed and Berthet, 2019), Wasserstein distances (Barrio and Loubes, 2019; Mena and Niles-Weed, 2019; del Barrio et al., 2023; Altschuler et al., 2022), transport maps (Hütter and Rigollet, 2021; Pooladian and Niles-Weed, 2022; Manole et al., 2022; Gonzalez-Sanz et al., 2022; Pooladian et al., 2023; Manole et al., 2023), and Fréchet means (Agueh and Carlier, 2011; Kim and Pass, 2017; Le Gouic and Loubes, 2017; Le Gouic et al., 2022; Altschuler et al., 2021) are investigated in the presence of sampling noise.

For the Wasserstein Fréchet mean, Le Gouic et al. (2022) established a parametric rate of convergence for the empirical Fréchet mean in the more general Alexandrov spaces, which include the 2-Wasserstein space as a special case, by introducing a bi-extendibility condition that translates into regularity conditions on the Kantorovich potentials. This condition was later relaxed by Chewi et al. (2020) when establishing the linear rate of convergence for gradient descent algorithms over the Wasserstein space.

Taking this further, Panaretos and Zemel (2016) and Agueh and Carlier (2017) established central limit theorems for the empirical Fréchet mean of 1D distributions. Subsequently, a central limit theorem for multivariate Gaussians was established by exploiting the first-order differentiability of optimal transport maps (Kroshnin et al., 2021).

#### Fréchet mean

The Fréchet mean is a natural generalization of the concept of an average to abstract metric spaces. For its properties in general curved metric spaces, see Ohta (2012); Yokota (2016); Le Gouic et al. (2022) and references therein. The existence and uniqueness of the Fréchet mean in the context of Riemannian manifolds and Wasserstein spaces are established in Agueh and Carlier (2011); Kim and Pass (2017); Le Gouic and Loubes (2017). The asymptotic properties of the empirical Fréchet mean on a Riemannian manifold are addressed in Bhattacharya and Patrangenaru (2003, 2005); Le Gouic et al. (2022).

#### Fréchet regression

The Fréchet regression model can be viewed as an extension of the Fréchet mean by incorporating a weighted average and was first introduced by Petersen and Müller (2019). Petersen et al. (2021) proposed an F-test specifically for the case of 1D density responses. In their approach, uniform convergence was not necessary for inference because the problem could be embedded into a Hilbert space, allowing for explicit expressions. However, in our case, demonstrating uniform convergence is crucial to ensure that the contributions from the remainder term in the Taylor expansion are negligible.

It is worth noting that while the uniform convergence of Fréchet regression is also addressed in Petersen and Müller (2019) and Chen and Müller (2022), their results are asymptotic and only uniform over a fixed compact set of x. In contrast, our result is non-asymptotic, with uniformity achieved within a compact set that expands in diameter to accommodate the potential unboundedness of supp X.

#### Regression models on manifolds

It is also important to note that the Fréchet regression model is defined purely in terms of distance, making it applicable to any abstract metric space. Meanwhile, a separate line of research focuses on regression on manifolds (Yuan et al., 2012; Cornea et al., 2017; Lin et al., 2023; Chen et al., 2023). These regression models are grounded in the concept of tangent spaces from differential geometry. Because tangent spaces are linear, they allow regression on manifolds to essentially reduce to classical linear regression within these tangent spaces.

### 1.3 Organization

The remainder of the paper is organized as follows. In Section 2, we provide the necessary background on optimal transport. Section 3.1 formulates the Fréchet regression model, followed by the assumptions outlined in Section 3.2. The main results are presented in Section 4, where we demonstrate the uniform convergence of our estimator in Section 4.1 and propose our test along with theoretical guarantees in Section 4.2. Then, Section 5 presents a Riemannian gradient descent algorithm and numerical simulations to validate our theory. Finally, Section 6 applies our test to a single-cell gene expression dataset, revealing age-related changes in gene co-expression networks. The proofs of our theorems and technical lemmas are provided in the Appendix. We conclude with a discussion in Section 7.

### 1.4 Notation

We denote by Z and $R_{+}$ the set of integers and the set of non-negative real numbers. For any a,b∈R, we write a∨b=max(a,b) and a∧b=min(a,b). For any x>0, we write ${\text{log}}^{+}(x)≔\text{log}(x)\vee 1$. For any integer K≥1,[K]={1,…,K}. Given $z_{i}\in R$ for i∈[n], the set $\left\{z_{1},\ldots ,z_{n}\right\}$ is denoted by $z_{1}^{n}$. The Euclidean norm on $R^{p}$ is denoted ‖⋅‖. For any $x\in R^{p}$ and L>0, let $B_{x}(L)=B(x,L)=\left\{y\in R^{d}:\|x-y\|\leq L\right\}$. We denote by ${𝒮}_{d},{𝒮}_{d}^{+},{𝒮}_{d}^{++}$ the set of all d×d symmetric, positive semi-definite and positive definite matrices. For any real a<b, we define ${𝒮}_{d}(a,b)≔\left\{A\in {𝒮}_{d}:aI_{d}≺A≺bI_{d}\right\}$. The subscript d is omitted when it’s clear from context. Given any $A\in {𝒮}_{d}$, denote the largest and smallest eigenvalue of A by $\lambda_{\text{max}}(A)$ and $\lambda_{\text{min}}(A)$. The Frobenius norm and operator norm of a matrix A is denoted by $\|A{\|}_{\text{F}}$ and $\|A{\|}_{\text{op}}$. Given any matrix $A\in R^{m,n}$, let vec $A\in R^{mn}$ denote the vectorized A obtained by stacking columns of A. Given a random variable X and α>0, the $\psi_{\alpha }$-“norm” of X, denoted by $\|X{\|}_{\psi_{\alpha }}$, is defined in Appendix B.2. The support of a probability distribution is denoted by supp(⋅).

Given normed spaces Y and Z, let L(Y;Z) denote the space of all bounded linear operator from Y to Z. Given a function ϕ:Y→Z and integer k≥0, the k-th differential $d^{k}\phi$, its operator norm $\left\|d^{k}\phi \right\|$ and symmetric norm $\left\|\left|d^{k}\phi \right|\right\|$ are defined in Section 2.

Finally, the quantities C and c will refer to constants whose value may change from line to line. Given sequences ${\left(a_{n}\right)}_{n=1}^{\infty }$ and ${\left(b_{n}\right)}_{n=1}^{\infty }$, we write $a_{n}≲b_{n}$ if there exists C>0 such that $a_{n}\leq Cb_{n}$, and we also write $a_{n}≍b_{n}$ if $b_{n}≲a_{n}≲b_{n}$. The constant C is always permitted to depend on d and other problem parameters when they are clear from context.

## 2 Preliminaries

We provide a concise overview of fundamental concepts in optimal transport, along with associated differential properties, specifically focusing on the case of centered Gaussian distributions.

Given a Polish space (E,d), let ${𝒫}_{2}(E)$ denote the collection of all (Borel) probability measures μ on E such that $E_{X\sim \mu }d(X,y{)}^{2}<\infty$ for some y∈E. One can show that the definition of ${𝒫}_{2}(E)$ is independent of the choice of y. We specialize to the case when $E=R^{d}$ with Euclidean distance. For any pair of measures $\mu ,\nu \in {𝒫}_{2}\left(R^{d}\right)$, let Π(μ,ν) be the set of couplings of between μ and ν, that is, the collection of probability measures π on $R^{d}\times R^{d}$ such that if (X,Y)~π, then X~μ and Y~ν. The 2-Wasserstein distance between μ and ν is defined as

(2) $$W(\mu ,\nu )≔{\left[\underset{\pi \in \Pi (\mu ,\nu )}{\text{inf}}E_{(X,Y)\sim \pi }\|X-Y{\|}^{2}\right]}^{1/2}$$

Here, with a slight abuse of notation, we use W to denote both the Wasserstein distance between two distributions and the Bures-Wasserstein distance between two PSD matrices. This notation is justified by the fact that both distances coincide and have a closed-form expression (1) when we identify centered Gaussian distributions with their covariance matrices.

Let ${𝒫}_{2,\text{ac}}\left(R^{d}\right)$ denote the subset of measures in ${𝒫}_{2}\left(R^{d}\right)$ that are absolutely continuous with respect to the Lebesgue measure. Given $\mu_{0},\mu_{1}\in {𝒫}_{2,\text{ac}}\left(R^{d}\right)$, Brenier’s theorem guarantees the existence of a unique optimal coupling $\pi^{⋆}\in \Pi \left(\mu_{0},\mu_{1}\right)$ that achieves the minimum in (2) and that it is induced by the optimal transport map $T_{\mu_{0}}^{\mu_{1}}:R^{d}\to R^{d}$ in the sense that $T_{\mu_{0}}^{\mu_{1}}(X)\sim \mu_{1}$ whenever $X\sim \mu_{0}$. Specifically, when $\mu_{0},\mu_{1}$ are centered Gaussian distributions with covariance matrices Q,S, the optimal transport map is given by the linear map

(3) $$T_{Q}^{S}=S^{1/2}{\left(S^{1/2}QS^{1/2}\right)}^{-1/2}S^{1/2}=Q^{-1/2}{\left(Q^{1/2}SQ^{1/2}\right)}^{1/2}Q^{-1/2}$$

For completeness, additional background on the geometry of optimal transport is provided in Appendix A.1.

Kroshnin et al. (2021) showed that for any fixed $S\in {𝒮}_{d}^{++},T_{Q}^{S}$ is (Fréchet) differentiable with respect to Q, with the differential at Q denoted by $dT_{Q}^{S}$. They also showed that for fixed $S\in {𝒮}_{d}^{++}$, the squared Wasserstein distance $W^{2}(\cdot ,S):{𝒮}_{d}^{++}\to R$ is twice differentiable, with the corresponding 1st and 2nd differential $dW^{2}(Q,S),d^{2}W^{2}(Q,S)$ satisfying

(4) $$dW^{2}(Q,S)(X)=\left\langle I-T_{Q}^{S},X\right\rangle \;d^{2}W^{2}(Q,S)(X,Y)=-\left\langle X,dT_{Q}^{S}(Y)\right\rangle$$

In this paper, higher-order differentials $d^{k}T_{Q}^{S}$ are essential for the development of the theory. Hence, additional background on functional calculus is provided in Appendix A.2 for self-containedness.

## 3 Problem Formulation

In this section, we formulate the Fréchet regression model in Section 3.1 and outline the assumptions in Section 3.2.

### 3.1 Fréchet regression on Bures-Wasserstein manifold

Given a metric space (𝒴,d), let P be a probability distribution over $R^{p}\times 𝒴$ that generates random pairs (X,Y). The primary challenge in formulating a regression model between Y and X lies in the fact that the metric-space-valued response Y does not lend itself to linear operations. The Fréchet regression model (Petersen and Müller, 2019) seeks to generalize classical linear regression to a general metric space, building upon the concept of the conditional Fréchet mean, which we introduce first.

The unconditional Fréchet mean $E_{\text{Fréchet}}Y$ generalizes the notion of an average in a general metric space (𝒴,d) and is defined as:

(5) $$E_{\text{Fréchet}}Y≔\underset{y\in 𝒴}{\text{argmin}}\;E_{Y}d^{2}(y,Y)$$

The above definition is motivated by the fact that when 𝒴 is a Euclidean space, $E_{\text{Fréchet}}Y$ coincides with the classical expectation EY. Similarly, the conditional Fréchet mean, denoted by $E_{\text{Fréchet}}[Y|X=x]$, is defined as

(6) $$E_{\text{Fréchet}}\left[Y|X=x\right]≔\underset{y\in 𝒴}{\text{argmin}}\;E_{Y|X=x}\left[d^{2}(y,Y)|X=x\right]$$

The existence and uniqueness of both the conditional and unconditional Fréchet mean in metric spaces can be ensured under additional assumptions; see Petersen and Müller (2019) and references therein for further details.

With the notion of the conditional Fréchet mean established, the Fréchet regression model proposed by Petersen and Müller (2019) satisfies:

(7) $$E_{\text{Fréchet}}\left[Y|X=x\right]=\underset{y\in 𝒴}{\text{argmin}}\;E_{(X,Y)\sim P}\left[w(x,X)d^{2}(y,Y)\right]$$

where the weight function w is defined as

(8) $$w(x,X)=1+(x-\mu {)}^{⊤}\Sigma^{-1}(X-\mu ),\;\mu =EX,\Sigma =\text{Var}(X)$$

The objective function $E\left[w(x,X)d^{2}(y,Y)\right]$ on the right-hand side of (7) generalizes the Fréchet mean (5) by introducing a weighted expectation, similar in spirit to kernel estimators in non-parametric statistics (Wasserman, 2006). The specific choice of the weight w(x,X) in (8) is motivated by the requirement that under the classical linear regression model, the minimizer corresponds to the desired conditional expectation of Y at x. Specifically, when 𝒴=R and $E[Y|X=x]=a^{⊤}(x-\mu )+b$, one can verify that

(9) $$a^{⊤}(x-\mu )+b=\underset{y\in R}{\text{argmin}}\;E_{\left(X,Y\right)}\left[w(x,X)(y-Y{)}^{2}\right]$$

When specialized to the Bures-Wasserstein manifold $(𝒴,d)=\left({𝒮}_{d}^{++},W\right)$, we use $Q\in {𝒮}_{d}^{++}$ to denote the response. The existence and uniqueness of both the conditional and unconditional Fréchet mean are guaranteed under mild moment conditions. The following lemma follows from Theorem 2.1 in Kroshnin et al. (2021).

**Lemma 1** *On the Bures*-*Wasserstein manifold* $\left({𝒮}_{d}^{++},W\right)$, *if* EtrQ<+∞, *then the Fréchet mean* $E_{\text{Fréchet}}Q$ *exists uniquely in* ${𝒮}_{d}^{++}$. *If* E[trQ∣X=x]<+∞, *then the conditional Fréchet mean* $E_{\text{Fréchet}}[Y|X=x]$ *exists uniquely in* ${𝒮}_{d}^{++}$.

We denote $Q^{*}\left(x\right)≔E_{\text{Fréchet}}[Q|X=x]$ as the conditional Fréchet mean of Q given X=x, and our Fréchet regression model assumes

(10) $$Q^{*}\left(x\right)=\underset{S\in {𝒮}_{d}^{++}}{\text{argmin}}\;F\left(x,S\right),\;\text{where}\;F\left(x,S\right)≔E_{\left(X,Q\right)}\left[w(x,X)W^{2}(S,Q)\right]$$

In general, there is no closed-form expression for $Q^{*}(x)$, similar to the situation with Bures-Wasserstein barycenters (Agueh and Carlier, 2011). To gain intuition about the types of functional relationships between Q and X that the Fréchet regression model (10) captures, consider a special case where Q is concentrated on matrices that commute with each other. In this setting, for any commuting matrices $S,Q\in {𝒮}_{d}^{++}$, the Bures-Wasserstein distance (1) between Q and S simplifies to $W(Q,S)={\left\|Q^{1/2}-S^{1/2}\right\|}_{\text{F}}$, which forms an isometric Hilbert embedding of supp Q. Under this simplification, the Fréchet regression model (10) essentially reduces to a linear regression model on the square roots of the covariance matrices in a similar flavor as (9):

$$Q^{*}(x)=\underset{S\in {𝒮}_{d}^{++}}{\text{argmin}}\;E_{\left(X,Q\right)}\left[w(x,X){\left\|S^{1/2}-Q^{1/2}\right\|}_{\text{F}}^{2}\right]$$

A specific example of this is provided in Example 1 in Section 5.

### 3.2 Assumptions

To establish rigorous theoretical guarantees for estimation and hypothesis testing, we impose the following model assumptions, which we believe are both theoretically minimal and sufficiently general.

We start with conditions on the marginal distribution of the covariate X and the conditional distribution of Q given X, as outlined in Assumption 1 and 2.

**Assumption 1** X *is sub*-*Gaussian with* $\|X{\|}_{\psi_{2}}\leq C_{\psi_{2}}$ *and* $\lambda_{\text{min}}(\Sigma )\geq c_{\Sigma }$ *for some constants* $C_{\psi_{2}},c_{\Sigma }>0$.

**Assumption 2** Given X=x∈suppX, *the eigenvalues of* Q *are bounded away from* 0 *and infinity in the sense that*

(11) $$P\left(Q\in {𝒮}_{d}\left(\gamma_{\Lambda }(\|x-\mu \|{)}^{-1},\gamma_{\Lambda }(\|x-\mu \|)\right)|X=x\right)=1$$

*where* $\gamma_{\Lambda }:R^{+}\to R^{+}$ *is defined as*

(12) $$\gamma_{\Lambda }(t)≔c_{\Lambda }(t\vee 1{)}^{C_{\Lambda }}$$

*for some constants $c_{\Lambda }\geq 1$ and* $C_{\Lambda }\geq 0$.

Assumption 2 implies upper and lower bounds on both the conditional expectation $Q^{*}(x)$ (see Lemma 33) and noise. These bounds become progressively weaker as x→∞. In the literature on covariance matrix estimation, an upper bound on the population covariance matrix is commonly assumed (Cai et al., 2010). The lower bound on $\lambda_{\text{min}}\left(Q^{*}(x)\right)$ here is analogous to the uniform upper bound on the conditional densities in Fréchet regression for 1D density response curves (Petersen et al., 2021, Assumption T4), since density is inversely proportional to the standard deviation in a 1D location-scale family. Assumptions on both upper and lower bounds are natural in the context of optimal transport. For example, Hütter and Rigollet (2021), Manole et al. (2022), and Pooladian and Niles-Weed (2022) assumed smoothness and strong convexity of the Brenier potential for optimal transport map estimation, which translates to upper and lower bounds on the eigenvalues of the covariance matrix in the Gaussian case. Similarly, Altschuler et al. (2021) assume both upper and lower bounds on the eigenvalues to ensure a variance inequality proposed in Chewi et al. (2020), which is critical for proving the $\sqrt{n}$-convergence of the empirical barycenter and the linear convergence of a gradient descent algorithm on the Bures-Wasserstein manifold.

**Remark 2** *The bounds presented here depend on* $\gamma_{\Lambda }(\|x-\mu \|)$, which diverges as x→∞. *This is motivated by the behavior of the conditional mean* E(Y∣X=x) *in classical linear regression, which also diverges as* x→∞. *Specifically, when* μ=0 and $E[Y|X]=a^{⊤}X+b$, *it can be shown that* $\left|E[Y|X]|\leq \gamma_{\Lambda }(X\right)$ *with* $c_{\Lambda }=\|a\|+|b|$ *and* $C_{\Lambda }=1$. *In our context, we assume a polynomial growth rate $\gamma_{\Lambda }(t)≲t^{C_{\Lambda }}$*, *a condition permitted by* (12) *and commonly satisfied in practical applications*.

**Remark 3** The bounded noise assumption can be relaxed by assuming that Q *has a light tail conditional on* $Q^{*}(X)$. *Specifically, we can assume that*

$$P\left\{\left.\frac{\lambda_{\text{min}}\left(Q^{*}(x)\right)}{\lambda_{\text{min}}(Q)}\vee \frac{\lambda_{\text{max}}(Q)}{\lambda_{\text{max}}\left(Q^{*}(x)\right)}>t\right|X=x\right\}≲\text{exp}\left(-ct^{\alpha }\right),\;\forall t>0$$

*for some constant* α>0. *The proof presented in our paper remains valid by incorporating additional concentration arguments*.

Assumption 1 and 2, together with Lemma 1, imply that the conditional Fréchet mean $Q^{*}(x)$ is well-defined. Based on this, we consider the Fréchet regression model given by (10).

**Assumption 3** *The Fréchet regression model holds. In other words, for any* x∈suppX, equation (10) *is satisfied*.

To ensure estimation consistency, some regularity conditions on F(x,⋅) are needed. Specifically, Assumption 4 below concerns the global behavior of F(x,⋅) outside local neighborhoods around its minimizer $Q^{*}(x)$, while Assumption 5 focuses locally on the lower bound of the eigenvalue of the second differential of F(x,⋅) at $Q^{*}(x)$.

**Assumption 4** *For any* $x\in \text{supp}\;X,F(x,\cdot ):{𝒮}_{d}^{++}\to R$ *has a unique minimizer. Moreover, there exist constants* $\alpha_{F}\geq 1$ *and* $\delta_{F}>0$ *such that for any* x∈suppX *and any* (δ,Δ) *that satisfies* $0\leq \delta \leq \delta_{F}\leq \Delta$, *the following*

(13) $$\text{inf}\left\{F(x,S)-F\left(x,Q^{*}(x)\right):\delta \leq {\left\|S-Q^{*}(x)\right\|}_{\text{F}}\leq \Delta \right\}\geq \frac{\delta^{\alpha_{F}}}{\gamma_{F}(\|x-\mu \|,\Delta )}$$

*holds where $\gamma_{F}:R^{+}\times R^{+}\to R^{+}$ is defined as*

$$\gamma_{F}\left(t_{1},t_{2}\right)=c_{F}{\left(t_{1}\vee 1\right)}^{C_{F}}{\left(t_{2}\vee 1\right)}^{C_{F}}$$

*for constants* $c_{F}\geq 1,C_{F}\geq 0$.

**Remark 4** Assumption 3 states that $Q^{*}(x)$ is a minimizer of F(x,⋅), *while Assumption 4 further assumes that it is the unique minimizer to guarantee the consistency of our M*-*estimation*-*type estimator* (15). *Additionally, condition* (13) *is motivated by the well*-*separated*-*maximizer assumption in M*-*Estimation* (Van Der Vaart and Wellner, 1996, *Lemma 3.2.1*), *which is also assumed in* (Petersen and Müller, 2019). *In the special case when* X *and* Q *are independent, Lemma 34 establishes that Assumption 4 holds with* $\delta_{F}=1,\alpha_{F}=2,C_{F}=1$ *and some constant* $c_{F}$ *large enough. Here we allow for a polynomial dependence on* ‖x−μ‖ *in the definition of* $\gamma_{\lambda }$ *so that* (13) *is still expected to hold in the general case when* X *and* Q *are not independent*.

**Assumption 5** For any x∈suppX, *consider the symmetric linear operator*

$$E\left(-w(x,X)dT_{Q^{*}(x)}^{Q}\right):{𝒮}_{d}\to {𝒮}_{d},$$

*which is the second differential of* F(x,.⋅) as $Q^{*}(x)$. *This operator has a lower bound for its minimum eigenvalue given by*:

(14) $$\lambda_{\text{min}}\left(-Ew(x,X)dT_{Q^{*}(x)}^{Q}\right)\geq \frac{1}{\gamma_{\lambda }(\|x-\mu \|)}$$

*where* $\gamma_{\lambda }:R^{+}\times R^{+}\to R^{+}$ *is defined as*

$$\gamma_{\lambda }(t)≔c_{\lambda }(t\vee 1{)}^{C_{\lambda }}$$

*for some constants* $c_{\lambda }\geq 1$ *and* $C_{\lambda }\geq 0$.

**Remark 5** *We provide some clarification on the term* $E\left(-w(x,X)dT_{Q^{*}(x)}^{Q}\right)$ *here. It is equal to the second differential of* $F(x,\cdot ):{𝒮}_{d}^{++}\to R$ *at* $Q^{*}(x)$. *Note that under the Fréchet regression model* (10), $Q^{*}(x)$ *is assumed to be the minimizer of the objective function* $F(x,\cdot ):{𝒮}_{d}^{++}\to R$. *As a result, the second differential of* F(x,⋅) *at* $Q^{*}(x)$ *is positive semi*-*definite. Similar to the non*-*singular Fisher information assumption in maximum likelihood estimation* (van der Vaart, 1998), *we further assume that the second differential* $E\left(-w(x,X)T_{Q^{*}(x)}^{Q}\right)$ *has positive eigenvalues that are lower*-*bounded as in* (14). *For relevant concepts in functional calculus, see* Appendix A.2*, and for the justification that* $E\left(-w(x,X)dT_{Q^{*}(x)}^{Q}\right)$ *is equal to the second differential, see* Appendix B.1.

**Remark 6** *In the special case when* X *and* Q *are independent, which is a consequence of Assumption 6 and the null hypothesis of no effect* (21) *below, one can show that (Lemma 34 in* Appendix B.3*) Assumption 5 holds for* $C_{\lambda }=0$ *and* $c_{\lambda }$ *large enough. Again, dependence on* ‖x−μ‖ *is allowed in order to account for the possible unboundedness of* x *when* X *and* Q *are independent*.

Finally, we assume conditional independence between X and Q given $Q^{*}(X)$ for hypothesis testing. However, this assumption is not necessary for the results on uniform convergence (Theorem 7) or the central limit theorem (Theorem 11).

**Assumption 6** X *and* Q *are independent conditional on* $Q^{*}(X)$.

## 4 Statistical Inference for Fréchet Regression on Bures-Wasserstein manifold

Building on the assumptions outlined in Section 3, we now focus on hypothesis testing within the Fréchet regression model. We begin by establishing the uniform convergence of the Fréchet regression estimator and proving a central limit theorem in Section 4.1. The uniform convergence is not only theoretically important but also crucial for determining the asymptotic size and power of our proposed test. In Section 4.2.1, we introduce the test statistic, followed by an analysis of its asymptotic null distribution and asymptotic power in Section 4.2.2.

### 4.1 Estimation under the Fréchet regression model

We define the Fréchet regression estimator as follows. For $\rho =n^{-1}$, let the empirical mean and the regularized covariance estimator be given by

$$\overset{‾}{X}=n^{-1}\sum_{i=1}^{n}X_{i},\;{\;\hat{\Sigma }}_{\rho }=n^{-1}\sum_{i-1}^{n}\left(X_{i}-\overset{‾}{X}\right){\left(X_{i}-\overset{‾}{X}\right)}^{⊤}+\rho I_{p}.$$

The estimator is then defined as

(15) $${\hat{Q}}_{\rho }\left(x\right)≔\underset{S\in {𝒮}_{d}^{++}}{\text{argmin}}\;F_{n,\rho }\left(x,S\right),\;F_{n,\rho }(x,S)≔\frac{1}{n}\sum_{i=1}^{n}\left[w_{n,\rho }\left(x,X_{i}\right)W^{2}\left(S,Q_{i}\right)\right]$$

where $w_{n,\rho }\left(x,X_{i}\right)=1+(x-\overset{‾}{X}){\hat{\Sigma }}_{\rho }^{-1}\left(X_{i}-\overset{‾}{X}\right)$.

The estimator was initially studied by Petersen and Müller (2019) with ρ=0, where its asymptotic properties in a general metric space were analyzed under additional assumptions on the covering number, based on the theory of M-estimation. Here, we introduce an additional regularization term, $\rho I_{p}$, to the empirical covariance matrix. This modification offers several benefits: First, similar to ridge regression, the regularization term enhances numerical stability due to the involvement of the estimated precision matrix in $w_{n,\rho }$. Next, as shown in Theorem 7, beyond providing a high-probability bound for the estimation error in (16), it enables us to establish in (17) a uniform non-asymptotic $\sqrt{n}$-rate of convergence for the expected estimation error defined as:

$$E\underset{\|x-\mu \|≲\sqrt{\text{log}\;n}}{\text{sup}}{\left\|{\hat{Q}}_{\rho }(x)-Q^{*}(x)\right\|}_{\text{F}}.$$

For ρ=0, as studied by Petersen and Müller (2019), it is challenging even to prove the existence of such a moment. However, by setting $\rho =n^{-1}$, we can show that this moment exists and converges uniformly at a parametric rate. Similar results have been reported in the literature on high-dimensional precision matrix estimation (Tony Cai and Luo, 2011; Cai et al., 2016). Furthermore, the inclusion of the regularization term $n^{-1}I_{p}$ does not affect the asymptotic central limit theorem (Theorem 11) or the asymptotic level and power of our test (Theorem 16, 18). This is because it only introduces a bias on the order of $n^{-1}$ which is negligible compared to the typical stochastic variation of order $n^{-1/2}$. In fact, ρ can be set to any $n^{-c}$ with a constant c>1/2. For simplicity, we fix $\rho =n^{-1}$ unless otherwise specified.

Our results on the non-asymptotic uniform convergence of the estimator are summarized in Theorem 7 below.

**Theorem 7** *Let* $1\leq L_{n}\leq C_{L}\sqrt{\text{log}\;n}$ *for some constant* $C_{L}>0$. *Suppose Assumption 1.5 hold. Then*

1. *for* $\rho \in \left\{0,n^{-1}\right\}$ *and any arbitrarily large fixed constant* τ>0, *with probability at least* $1-C_{3}n^{-\tau }$, *we have*
(16) $$\underset{\|x-\mu \|\leq L_{n}}{\text{sup}}{\left\|{\hat{Q}}_{\rho }(x)-Q^{*}(x)\right\|}_{\text{F}}\leq C_{1}\frac{{\text{log}}^{C_{2}}\;n}{\sqrt{n}}$$
  *for some constants* $C_{1},C_{2},C_{3}>0$ *independent of* n.
2. *for* $\rho =n^{-1}$, *we have*
(17) $$E\underset{\|x-\mu \|\leq L_{n}}{\text{sup}}{\left\|{\hat{Q}}_{\rho }(x)-Q^{*}(x)\right\|}_{\text{F}}\leq C_{4}\frac{{\text{log}}^{C_{5}}\;n}{\sqrt{n}}$$
  *for some constants $C_{4},C_{5}>0$ independent of* n.

**Remark 8** *The factor* $\sqrt{\text{log}\;n}$ *in the upper bound of diameter* $L_{n}$ *can be refined to* ${\text{log}}^{\xi_{L}}\;n$ *for an arbitrarily large fixed constant* $\xi_{L}>0$. *One implication of Theorem 7 is that for* $\rho \in \left\{0,n^{-1}\right\}$, *we have*

(18) $$\underset{i\in [n]}{\text{max}}{\left\|{\hat{Q}}_{\rho }\left(X_{i}\right)-Q^{*}\left(X_{i}\right)\right\|}_{\text{F}}≲\frac{\text{polylog}(n)}{\sqrt{n}}$$

*with probability at least $1-O\left(n^{-\tau }\right)$ for any fixed* τ>0. *This result is crucial for deriving the asymptotic size and power of our proposed test.* Petersen and Müller (2019) *investigated asymptotic uniform convergence over a fixed compact set. In our context, their results state that for a fixed* L>0,

$$\underset{\|x-\mu \|\leq L}{\text{sup}}W\left({\hat{Q}}_{0}(x),Q^{*}(x)\right)=O_{p}\left(n^{-\frac{1}{2\left(\alpha^{\prime }-1\right)}}\right)$$

*for any* $\alpha^{\prime }>2$. *However, their results do not guarantee* (18) *unless the norm of the covariate* X *is bounded by* L, *and therefore are insufficient for deriving the size and power of our test when* X *has unbounded support. Consequently, we employ non*-*asymptotic bounds and derive* (18) *from* (16).

**Remark 9** *We use the Frobenius norm to quantify error here instead of the Wasserstein distance for the following reasons:*

- *Differential properties of $W^{2}(\cdot ,\cdot )$*: *The Wasserstein distance is used in the objective function* (10) *due to its favorable properties under scaling and rotation, as well as its meaningful probabilistic interpretation, as discussed in* Section 1. *After adopting the Bures*-*Wasserstein metric in our formulation of the Fréchet regression model, we need to leverage the differential properties of this metric to establish the statistical properties of the estimator. These differential properties are more naturally expressed using the Frobenius norm. For instance, for any* $Q,\tilde{Q}\in {𝒮}_{d}^{++}$*and any* $A\in {𝒮}_{d}$, *we have*
$$d_{Q}W^{2}(Q,\tilde{Q})(A)=\left\langle I_{d}-T_{Q}^{\tilde{Q}},A\right\rangle$$
  *where the Frobenius inner product is used, and* $T_{Q}^{\tilde{Q}}$ *is the optimal transport map from the normal distribution* N(0,Q) *to* $N(0,\tilde{Q})$. *Similarly, existing Taylor expansions for the Bures*-*Wasserstein metric are also formulated in terms of the Frobenius norm. Hence, it is more straightforward to express the estimation error using the Frobenius norm. For further details on the differential properties of* W, *see* Appendix B.1.
- *Relationship Between Frobenius Norm and Wasserstein Distance: The Frobenius norm and the Wasserstein distance are closely related. Specifically, Lemma 21 in* Appendix B.1 *shows that for any* $Q,\tilde{Q}\in {𝒮}_{d}^{++}$, *the Wasserstein distance* $W(Q,\tilde{Q})$ *can be upper*-*bounded in terms of their Euclidean distance*. *Consequently, we can derive analogous results for the Wasserstein distance, as in Corollary 10 below. In fact, the two rates are equivalent up to a* polylog (n) *factor in our setting*.

**Corollary 10** *Let* $1\leq L_{n}\leq C_{L}\sqrt{\text{log}\;n}$ *for some constant* $C_{L}>0$. *Suppose Assumption 1*-*5 hold. Then*

1. *for* $\rho \in \left\{0,n^{-1}\right\}$ *and any arbitrarily large fixed constant* τ>0, *with probability at least* $1-C_{W3}n^{-\tau }$, *we have*
$$\underset{\|x-\mu \|\leq L_{n}}{\text{sup}}W\left({\hat{Q}}_{\rho }(x),Q^{*}(x)\right)\leq C_{W1}\frac{{\text{log}}^{C_{W2}}\;n}{\sqrt{n}}$$
  *for some constants $C_{W1},C_{W2},C_{W3}>0$ independent of* n.
2. *for* $\rho =n^{-1}$, *we have*
$$E\underset{\|x-\mu \|\leq L_{n}}{\text{sup}}W\left({\hat{Q}}_{\rho }(x),Q^{*}(x)\right)\leq C_{W4}\frac{{\text{log}}^{C_{W5}}\;n}{\sqrt{n}}$$
  *for some constants* $C_{W4},C_{W5}>0$ *independent of* n.

To the best of our knowledge, Theorem 7 and Corollary 10 provide the first non-asymptotic uniform convergence results for Fréchet regression over a potentially diverging region. The proof of Theorem 7 is intricate, and we provide an outline here. First, we use an approach similar to Agueh and Carlier (2011) to establish that the largest eigenvalues of the estimates ${\hat{Q}}_{\rho }(x)$ are uniformly bounded from above. Next, we show that ${\hat{Q}}_{\rho }(x)$ converges uniformly at a slow rate by employing the chaining method. Special attention is required here due to the Hölder continuity of the squared distance $W^{2}$; see Lemma 38 for details. Finally, we achieve a uniform fast rate of convergence by solving a quadratic inequality related to the convergence rate. A detailed proof of Theorem 7 is provided in Appendix C.

Theorem 7 implies the pointwise consistency of the Fréchet regression estimator (15). Moving one step further, we establish a pointwise central limit theorem that is elusive in general metric spaces with nonzero curvature (Petersen and Müller, 2019). In order to present the theorem, we pause to introduce several notations. First, for any $x\in R^{p}$, define $\vec{x}≔{\left(\begin{array}{ll} 1 & x^{⊤} \end{array}\right)}^{⊤}$. For any random vector $X\in R^{p}$ with covariance matrix Σ, denote $\vec{\Sigma }=E\vec{X}{\vec{X}}^{⊤}\in R^{\left(p+1)\times (p+1\right)}$. Next, let ${\left[E\left(-w(x,X)dT_{Q^{*}(x)}^{Q}\right)\right]}^{-1}$ denote the inverse of $E\left(-w(x,X)dT_{Q^{*}(x)}^{Q}\right)$ which is a linear operator in $L\left({𝒮}_{d};{𝒮}_{d}\right)$ (Appendix B.1). Last, given elements $x_{1},x_{2}$ of a Hilbert space ℋ, the tensor product operator $x_{1}⊗x_{2}:ℋ\to ℋ$ is defined by $\left(x_{1}⊗x_{2}\right)y=\left\langle x_{1},y\right\rangle x_{2}$ for any y∈ℋ; see Hsing and Eubank (2015) for properties of the tensor product operator and its role in the central limit theorem for random elements of a Hilbert space.

With these notations in place, the central limit theorem for the Fréchet regression estimator ${\hat{Q}}_{\rho }(x)$ is stated as Theorem 11.

**Theorem 11** *Let* $\rho \in \left\{0,n^{-1}\right\}$. *Suppose Assumption 1*-*5 hold. Then for any fixed* x∈suppX, *the following central limit theorem*

(19) $$\sqrt{n}\left[{\hat{Q}}_{\rho }(x)-Q^{*}(x)\right]\overset{w}{\to }{\left[E_{\left(X,Q\right)}\left(-w(x,X)dT_{Q^{*}(x)}^{Q}\right)\right]}^{-1}Z_{x},$$

*holds. Here* $Z_{x}\sim 𝒩\left(0,\Xi_{x}\right)$ *is a Gaussian random element of $R^{d\times d}$ with covariance operator* $\Xi_{x}$ *equal to* $EV_{x}⊗V_{x}$ *where*

$$V_{x}=V_{x,1}+V_{x,2}\;V_{x,1}=w(x,X)\left(T_{Q^{*}(x)}^{Q}-I_{d}\right)\;V_{x,2}=-\left({\vec{x}}^{⊤}{\vec{\Sigma }}^{-1}\left(\vec{X}{\vec{X}}^{⊤}-\vec{\Sigma }\right){\vec{\Sigma }}^{-1}⊗I_{d}\right)\cdot \left(E\vec{X}⊗\left(T_{Q^{*}(x)}^{Q}-I_{d}\right)\right)$$

**Remark 12** *Since a linear transformation of a multivariate normal distribution is still normal, Theorem 11 implies that asymptotically the entries of* $\sqrt{n}\left({\hat{Q}}_{\rho }(x)-Q^{*}(x)\right)$ *jointly follow a multivariate normal distribution in* $R^{d^{2}}$ *when vectorized. More specifically, we have*

(20) $$\sqrt{n}\left(\text{vec}\;{\hat{Q}}_{\rho }\left(x\right)-\text{vec}\;Q^{*}(x)\right)\overset{w}{\to }𝒩\left(0,\Omega_{x}\right)$$

*Here* $\Omega_{x}=H_{x}^{-1}E\left(\text{vec}{\;V}_{x}\right){\left(\text{vec}\;V_{x}\right)}^{⊤}H_{x}^{-1}$ *where* $H_{x}\in R^{d^{2}\times d^{2}}$ is the matrix representing the invertible linear operator $E\left(-w(x,X)dT_{Q^{*}(x)}^{Q}\right)$; *see* Appendix B.1 *for the expression of* $H_{x}$.

**Remark 13** Theorem 11 can be utilized to obtain confidence regions for entries of $Q^{*}(x)$. *The asymptotic variance can be estimated using the plug*-*in method as follows*:

$${\hat{\Xi }}_{x}=\frac{1}{n}\sum_{i=1}^{n}V_{x,i}⊗V_{x,i}$$

*where*

$$V_{x,i}=V_{x,1,i}+V_{x,2,i}\;V_{x,1,i}=w_{n,\rho }\left(x,X_{i}\right)\left(T_{{\hat{Q}}_{\rho }(x)}^{Q_{i}}-I_{d}\right)\;V_{x,2,i}=\left(-{\vec{x}}^{⊤}{\hat{\vec{\Sigma }}}_{\rho }^{-1}\left({\vec{X}}_{i}{\vec{X}}_{i}^{⊤}-\hat{\vec{\Sigma }}\right){\hat{\vec{\Sigma }}}_{\rho }^{-1}⊗I_{d}\right)\cdot \left(\frac{1}{n}\sum_{j=1}^{n}{\vec{X}}_{j}⊗\left(T_{{\hat{Q}}_{\rho }(x)}^{Q_{j}}-I_{d}\right)\right)$$

*Similarly*, ${\left[E_{\left(X,Q\right)}\left(-w(x,X)dT_{Q^{*}(x)}^{Q}\right)\right]}^{-1}$ *can be estimated by*

$${\left[\frac{1}{n}\sum_{i=1}^{n}\left(-w_{n,\rho }\left(x,X_{i}\right)dT_{{\hat{Q}}_{\rho }(x)}^{Q_{i}}\right)\right]}^{-1}$$

*In* Figure 1 *in* Section 5.2, *the asymptotic variance of each entry of* $Q^{*}(x)$ *is extracted using* (20) *and appropriate indexing*

Theorem 11 shows that the covariance operator $\Xi_{x}$ has contributions from both $V_{x,1}$ and $V_{x,2}$. If the expectation μ and the covariance matrix Σ of the predictor X are known, and we obtain an estimate ${\hat{Q}}_{0}^{*}(x)$ of $Q^{*}(x)$ by directly optimizing an ‘oracle’ objective function $F_{n}^{*}(x,S)$ defined as $F_{n}^{*}(x,S)=\frac{1}{n}\sum_{i=1}^{n}w\left(x,X_{i}\right)W^{2}\left(S,Q_{i}\right)$, then ${\hat{Q}}_{0}^{*}(x)$ would follow a central limit theorem with covariance operator exactly equal to $EV_{1,x}⊗V_{1,x}$. When μ and Σ are unknown and empirical estimates $\hat{\mu }$ and $\hat{\Sigma }$ are plugged in as in (15), there is an additional contribution to $\Xi_{x}$ from $V_{x,2}$. Theorem 11 is supported by numerical experiments in Section 5, and the proof is given in Appendix D. To the best of our knowledge, the expression for $\Xi_{x}$ cannot be further simplified in general. However, under the hypothesis that X and Q are independent, which can be a consequence of the null hypothesis in Section 4.2 and Assumption 6, the term $V_{x,2}$ vanishes, leaving $\Xi_{x}$ with contributions only from $V_{x,1}$. This result is summarized in Corollary 14 below; see Appendix E for the proof.

**Corollary 14** *Instate the assumptions in Theorem 11. If* X *and* Q *are independent, then*

$$\sqrt{n}\left[{\hat{Q}}_{\rho }(x)-Q^{*}(x)\right]\overset{w}{\to }{\left(E_{\left(X,Q\right)}\left(-w(x,X)dT_{Q^{*}(x)}^{Q}\right)\right)}^{-1}Z_{x}^{\prime },$$

*holds where* $Z_{x}^{\prime }\sim 𝒩\left(0,\Xi_{x}^{\prime }\right)$ *and*

$$\Xi_{x}^{\prime }=E\left[w(x,X)\left(T_{Q^{*}}^{Q}-I_{d}\right)⊗\left(T_{Q^{*}}^{Q}-I_{d}\right)w(x,X)\right]$$

### 4.2 Hypothesis testing

In this section, we focus on testing the global null hypothesis of no effects within the Fréchet regression model on the Bures-Wasserstein manifold, defined as follows:

(21) $${ℋ}_{0}:Q^{*}\left(x\right)\equiv Q^{*}\;\text{for}\;\text{some}\;\text{unknown}\;Q^{*},$$

where $Q^{*}(x)$ is constant across all x.

We begin by providing the motivation and the formulation of the test statistic in Section 4.2.1. Subsequently, we explore its asymptotic null distribution in Section 4.2.2, followed by an analysis of the asymptotic size and power of the proposed test.

#### 4.2.1 Test statistic

Note that a key distinction between the Fréchet regression model (10) and classical linear or generalized linear regression models is that the conditional expectation in the Fréchet model is determined by an optimization problem, rather than through a link function. Consequently, there is no parameter analogous to the slope β found in linear models. Therefore, the null hypothesis can only be tested by directly aggregating comparisons between the estimated predictions ${\hat{Q}}_{\rho }\left(X_{i}\right)$ and the Fréchet mean $Q^{*}$.

Given the uniform consistency of the Fréchet regression estimator ${\hat{Q}}_{\rho }(x)$ in Theorem 7, we propose the following test statistic for testing the null hypothesis in (21).


(22) $${\hat{𝒯}}_{\rho }=\sum_{i=1}^{n}{\left\|{\hat{H}}_{\rho }\cdot \left({\hat{Q}}_{\rho }\left(X_{i}\right)-{\hat{Q}}_{\rho }(\overset{‾}{X})\right)\right\|}_{\text{F}}^{2},\;\text{where}\;{\hat{H}}_{\rho }=-\frac{1}{n}\sum_{i=1}^{n}dT_{{\hat{Q}}_{\rho }(\overset{‾}{X})}^{Q_{i}}$$

To develop some intuition about ${\hat{𝒯}}_{\rho }$, consider that under the null hypothesis (21), the “difference” between ${\hat{Q}}_{\rho }\left(X_{i}\right)$ and $Q^{*}$ should be small. Since $Q^{*}$ is unknown, we estimate it by ${\hat{Q}}_{\rho }(\overset{‾}{X})$. Note that ${\hat{Q}}_{\rho }(\overset{‾}{X})$ coincides with the Fréchet mean of $Q_{1},\ldots ,Q_{n}$. Specifically, by definition (15), we have $w_{n,\rho }\left(\overset{‾}{X},X_{i}\right)=1$, which implies:

$${\hat{Q}}_{\rho }(\overset{‾}{X})=\underset{S\in {𝒮}_{d}^{++}}{\text{argmin}}\frac{1}{n}\sum_{i=1}^{n}W^{2}\left(S,Q_{i}\right)$$

This is precisely the Fréchet mean of $Q_{1},\ldots ,Q_{n}$. Therefore, a sensible test statistic would be of the form

$${𝒯}_{f}=\sum_{i=1}^{n}f\left({\hat{Q}}_{\rho }\left(X_{i}\right),{\hat{Q}}_{\rho }(\overset{‾}{X})\right)$$

where $f:R^{d\times d}\times R^{d\times d}\to R$ is some function that measures the “difference” between ${\hat{Q}}_{\rho }\left(X_{i}\right)$ and ${\hat{Q}}_{\rho }(\overset{‾}{X})$. The function f is expected to satisfy f(⋅,⋅)≥0,f(Q,Q)=0 and f(Q,S)=f(S,Q). Assuming tightness of the second-order Taylor approximation, the uniform consistency of ${\hat{Q}}_{\rho }(x)$ (Theorem 7) then implies

(23) $${𝒯}_{f}\approx \sum_{i=1}^{n}\left\langle \frac{1}{2}H_{f}\left({\hat{Q}}_{\rho }\left(X_{i}\right)-{\hat{Q}}_{\rho }(\overset{‾}{X})\right),{\hat{Q}}_{\rho }\left(X_{i}\right)-{\hat{Q}}_{\rho }(\overset{‾}{X})\right\rangle$$

where $H_{f}$ is the Hessian of f at ${\hat{Q}}_{\rho }(\overset{‾}{X})$. This justifies the quadratic form of (22).

Furthermore, the specification of $H_{f}$ should depend on the detailed distribution of ${\hat{Q}}_{\rho }\left(X_{i}\right)-{\hat{Q}}_{\rho }(\overset{‾}{X})$ such that the quadratic form in (23) has a well-defined asymptotic distribution. To derive the asymptotic distribution of ${\hat{Q}}_{\rho }\left(X_{i}\right)-{\hat{Q}}_{\rho }(\overset{‾}{X})$, we rely on the optimality condition $\sum_{j}w_{n,\rho }\left(x,X_{j}\right)\left(T_{{\hat{Q}}_{\rho }(x)}^{Q_{j}}-I_{d}\right)=0$. Under the null hypothesis (21), and assuming tightness of the first order approximation of the optimality conditions at $X_{i}$ and $\overset{‾}{X}$ we have

$$0\approx \sum_{j}w_{n,\rho }\left(X_{i},X_{j}\right)\left(T_{Q^{*}}^{Q_{j}}-I_{d}\right)+\sum_{j}w_{n,\rho }\left(X_{i},X_{j}\right)dT_{Q^{*}}^{Q_{j}}\left({\hat{Q}}_{\rho }\left(X_{i}\right)-Q^{*}\right)\;0\approx \sum_{j}w_{n,\rho }\left(\overset{‾}{X},X_{j}\right)\left(T_{Q^{*}}^{Q_{j}}-I_{d}\right)+\sum_{j}w_{n,\rho }\left(\overset{‾}{X},X_{j}\right)dT_{Q^{*}}^{Q_{j}}\left({\hat{Q}}_{\rho }(\overset{‾}{X})-Q^{*}\right)$$

Take the difference and rearrange, we arrive at

$$\left(-\frac{1}{n}\sum_{j}dT_{Q^{*}}^{Q_{j}}\right)\cdot \sqrt{n}\left({\hat{Q}}_{\rho }\left(X_{i}\right)-Q^{*}\right)\approx \underset{a_{1}\left(X_{i}\right)}{\underbrace{\frac{1}{\sqrt{n}}\sum_{j}\left(w_{n,\rho }\left(X_{i},X_{j}\right)-1\right)\left(T_{Q^{*}}^{Q_{j}}-I_{d}\right)}}\;+\underset{a_{2}\left(X_{i}\right)}{\underbrace{\frac{1}{\sqrt{n}}\sum_{j}\left(w_{n,\rho }\left(X_{i},X_{j}\right)-1\right)dT_{Q^{*}}^{Q_{j}}\left({\hat{Q}}_{\rho }\left(X_{i}\right)-Q^{*}\right)}}$$

It can be shown that $a_{2}\left(X_{i}\right)$ is negligible compared to $a_{1}\left(X_{i}\right)$, leading to

$$\left(-\frac{1}{n}\sum_{j}dT_{Q^{*}}^{Q_{j}}\right)\cdot \sqrt{n}\left({\hat{Q}}_{\rho }\left(X_{i}\right)-Q^{*}\right)\approx a_{1}\left(X_{i}\right).$$

Specifically, the intuition behind the negligibility is as follows. The null hypothesis (21) and Assumption 6 imply that X and Q are independent. As a result, $\frac{1}{\sqrt{n}}\sum_{j}\left(w\left(x,X_{j}\right)-1\right)dT_{Q^{*}}^{Q_{j}}$ has zero expectation and is of order $O_{p}(1)$ by the central limit theorem, suggesting that

$$\frac{1}{\sqrt{n}}\sum_{j}\left(w_{n,\rho }\left(X_{i},X_{j}\right)-1\right)dT_{Q^{*}}^{Q_{j}}=O_{p}(1)$$

by an approximation argument. Then the consistency of ${\hat{Q}}_{\rho }(x)$ implies that $a_{2}\left(X_{i}\right)$ is of order $o_{p}(1)$, making it negligible compared to $a_{1}\left(X_{i}\right)$.

Further calculations confirm a tractable asymptotic distribution for $\sum {\left\|a_{1}\left(X_{i}\right)\right\|}^{2}$, which suggests setting $H_{f}=\left(-\frac{1}{n}\sum_{j}dT_{Q^{*}}^{Q_{j}}\right)⊗\left(-\frac{1}{n}\sum_{j}dT_{Q^{*}}^{Q_{j}}\right)$. Since $Q^{*}$ is unknown, $H_{f}$ is approximated by ${\hat{H}}_{\rho }⊗{\hat{H}}_{\rho }$, leading directly to our test statistic ${\hat{𝒯}}_{\rho }$ as defined in (22).

**Remark 15** Petersen et al. (2021) *proposed the following test statistic for the case where responses are 1D densities, which has a simpler form compared to ours:*

$${\hat{𝒯}}_{\rho ,1\text{D}}=\sum_{i=1}^{n}W^{2}\left({\hat{Q}}_{\rho }\left(X_{i}\right),{\hat{Q}}_{\rho }(\overset{‾}{X})\right)$$

*with* ρ=0. *However, their results rely heavily on the isometric Hilbert embedding of the 1D Wasserstein space and do not generalize to higher dimensions. In contrast, our test statistic* (22) *is applicable in any dimension and is motivated by the Wald statistic used in generalized linear models. Moreover, one can show that* ${\hat{𝒯}}_{\rho }$ *and* ${\hat{𝒯}}_{n,1\text{D}}$ *are equivalent in the special case of 1D Gaussian distributions. Since our test statistic* (22) *can also be viewed as a generalization of the numerator of the global* F-*test in multiple linear regression, we refer to* ${\hat{𝒯}}_{\rho }$ *in* (22) *as the Wasserstein* F-*statistic following* Petersen et al. (2021).

#### 4.2.2 Theoretical properties

We now discuss the theoretical guarantees of our test statistic ${\hat{𝒯}}_{\rho }$ and the corresponding test. To begin, Theorem 16 provides the asymptotic null distribution of ${\hat{𝒯}}_{\rho }$.

**Theorem 16** *Let* $\rho \in \left\{0,n^{-1}\right\}$. *Suppose Assumption 1*-*3 and 6 hold. Then under the null* (21), *the test statistic* ${\hat{𝒯}}_{\rho }$ *satisfies*

(24) $${\hat{𝒯}}_{\rho }\overset{w}{\to }\sum_{i}\lambda_{i}w_{i}$$

*where* $w_{i}$ *are i.i.d.* $\chi_{p}^{2}$ *random variables, and* $\lambda_{i}$ *are the eigenvalues of the following operator:*

$$E\left[\left(T_{Q_{*}(\mu )}^{Q}-I_{d}\right)⊗\left(T_{Q_{*}(\mu )}^{Q}-I_{d}\right)\right]$$

**Remark 17** *The proof of Theorem 16 relies on the uniform consistency established in Theorem 7, since* ${\hat{𝒯}}_{\rho }$ *involves estimated predictions* ${\hat{Q}}_{\rho }(\cdot )$ *at random covariates* ${\left\{X_{i}\right\}}_{i\in [n]}$. *In contrast, the uniform consistency is not needed in* Petersen et al. (2021) *because the Wasserstein space* $W_{2}(R)$ *is essentially flat (has zero sectional curvature), which allows for a closed*-*form expression for* ${\hat{𝒯}}_{n,1D}$. *However, the Bures*-Wasserstein manifold $\left({𝒮}_{d}^{++},W\right)$ *is positively curved when* d>1 (Ambrosio et al., 2005), *and no closed*-*form expression is available for* ${\hat{𝒯}}_{\rho }$. *Thus, we rely on uniform consistency to ensure the tightness of the Taylor approximation. For the proof, see* Appendix F.

Theorem 16 asserts that ${\hat{𝒯}}_{\rho }$ converges weakly to a weighted sum of $\chi_{p}^{2}\text{s}$ with weights determined by the eigenvalues of the covariance operator $E\left(T_{Q_{*}}^{Q}-I_{d}\right)⊗\left(T_{Q_{*}}^{Q}-I_{d}\right)$. To get a corresponding test, note that the asymptotic null distribution in Theorem 16 depends on unknown parameters, namely the eigenvalues $\lambda_{i}$, which must be approximated to formulate a rejection region. A natural approach would be to estimate the eigenvalues ${\hat{\lambda }}_{i}$ of the sample average $\frac{1}{n}\sum_{i=1}^{n}T_{{\hat{Q}}_{\rho }(\overset{‾}{X})}^{Q_{i}}⊗T_{{\hat{Q}}_{\rho }(\overset{‾}{X})}^{Q_{i}}$ and let ${\hat{q}}_{1-\alpha }$ be the 1−α quantile of $\sum_{i=1}{\hat{\lambda }}_{i}w_{i}$. Then we define our test $\Phi_{\alpha }$ for any α∈(0,1) by

(25) $$\Phi_{\rho ,\alpha }=1\left({\hat{𝒯}}_{\rho }>{\hat{q}}_{1-\alpha }\right)$$

With Theorem 16 established, Proposition 1 below shows that $\Phi_{\rho ,\alpha }$ has an asymptotic size of α under the null hypothesis. See Appendix G for the proof.

**Proposition 1** *Suppose Assumption 1*-*3 and 6 hold. Then under the null* (21),

$$P\left({\hat{𝒯}}_{\rho }>{\hat{q}}_{1-\alpha }\right)\to \alpha$$

as n→∞.

Finally, we turn to an analysis of the power of the test $\Phi_{\rho ,\alpha }$ under a sequence of contiguous alternatives. To this end, we denote by P the set of distributions of (X,Q) that satisfy Assumption 1 – 6,

$$P≔\left\{P\in {𝒫}_{2}\left(R^{p}\times {𝒮}_{d}^{+}\right):P\;\text{satisfies}\;\text{Assumption}\;1-6\right\}$$

For any P∈P, we measure the deviation of $Q^{*}(x)$ from being a constant function of x by $E{\;\text{dist}}^{2}\left(Q^{*}(X),Q^{*}(\mu )\right)$ where dist is chosen to be either the Wasserstein distance or the Frobenius distance. We define the corresponding alternatives under each distance as follows:

$$H_{1,n}:P\in P_{F}\left(a_{n}\right)≔\left\{\tilde{P}\in P:E_{\left(X,Q\right)\sim \tilde{P}}{\left\|Q^{*}\left(X\right)-Q^{*}\left(\mu \right)\right\|}_{\text{F}}^{2}\geq a_{n}^{2}\right\},\;{\tilde{H}}_{1,n}:P\in P_{W}\left(a_{n}\right)≔\left\{\tilde{P}\in P:E_{\left(X,Q\right)\sim \tilde{P}}W^{2}\left(Q^{*}\left(X\right),Q^{*}\left(\mu \right)\right)\geq a_{n}^{2}\right\}.$$

Theorem 18 shows that $\Phi_{\rho ,\alpha }$ is powerful against both $H_{1,n}$ and ${\tilde{H}}_{1,n}$ whenever $a_{n}≳n^{-\left(1/2-\alpha_{2}\right)}$ for some constant $\alpha_{2}>0$. The proof is provided in Appendix H.

**Theorem 18** *Let* $\rho \in \left\{0,n^{-1}\right\}$. *Consider a sequence of alternative hypotheses* $H_{1,n}$ *with* $a_{n}$ *being a sequence such that $a_{n}≳\frac{1}{n^{1/2-\alpha_{2}}}$ for some constant $\alpha_{2}>0$*. *Then the worst case power converges uniformly to* 1*, that is*

$$\underset{P\in P_{F}\left(a_{n}\right)}{\text{inf}}P\left({\hat{𝒯}}_{\rho }>{\hat{q}}_{1-\alpha }\right)\to 1$$

*as* n→∞. *The same result also holds for alternative hypotheses* ${\tilde{H}}_{1,n}$ *defined by the Wasserstein distance, that is*

$$\underset{P\in P_{W}\left(a_{n}\right)}{\text{inf}}P\left({\hat{𝒯}}_{\rho }>{\hat{q}}_{1-\alpha }\right)\to 1$$

*as* n→∞.

## 5 Algorithm and Numerical Experiments

In this section, we propose a Riemannian gradient descent algorithm for optimizing (15) in Section 5.1. We then present a series of numerical experiments in Section 5.2 to validate our theoretical results on the central limit theorem (Theorem 11), asymptotic null distribution (Theorem 16) and power (Theorem 18). Additionally, in Section 5.3, we conduct simulations under a setting where Q is not directly observed but is estimated from data, to assess the deviation from the setting with perfect observations.

### 5.1 Riemannian gradient descent algorithm

Motivated by the Bures-Wasserstein gradient descent algorithm (Chewi et al., 2020; Altschuler et al., 2021) for the vanilla Bures-Wasserstein barycenter, we propose a gradient descent algorithm to compute ${\hat{Q}}_{\rho }(x)$ in (15), which is given as Algorithm 1.

**Algorithm 1:** GD for Fréchet regression

1:	**Input:** covariates ${\left\{X_{i}\right\}}_{i=1}^{n}$, responses ${\left\{Q_{i}\right\}}_{i=1}^{n}$, $\rho \in \left\{0,n^{-1}\right\}$, covariate x, learning rate η, initialization $S_{0}$, maximum number of iterations T, threshold eps.
2:	Initialize $S\leftarrow S_{0}$.
3:	**for** t=1,…,T **do**
4:	Set
	(26) $$G\leftarrow I_{d}+\eta \cdot \frac{1}{n}\sum_{i=1}^{n}w_{n,\rho }\left(x,X_{i}\right)\left(T_{S}^{Q_{i}}-I_{d}\right)$$
5:	Set
	(27) S←GSG
6:	**if** $\|G{\|}_{\text{F}}<eps$ **then**
7:	**break**
8:	**Output:** S.

Algorithm 1 can be viewed as a Riemannian gradient descent algorithm (see Appendix A and Panaretos and Zemel (2016); Chewi et al. (2020); Altschuler et al. (2021)). Intuitively, $-\frac{1}{n}\sum_{i=1}^{n}w_{n,\rho }\left(x,X_{i}\right)\left(T_{S}^{{\overset{‾}{Q}}_{i}}-I_{d}\right)$ is the derivative of the objective function $F_{n,\rho }(x,S)$ in (15) in the tangent space (Ambrosio et al., 2005, Corollary 10.2.7) and (26) corresponds to one gradient step in the tangent space with step size η. Then (27) is mapping the gradient step in the tangent space back to ${𝒮}_{d}^{++}$ through the exponential map (Appendix A)

The algorithm terminates if the Frobenius norm of the gradient G falls below the threshold eps, indicating that the relative change in the gradient update is less than eps. For the initialization $S_{0}$, optimization over the Euclidean space typically starts near the origin (Chen et al., 2019; Ye and Du, 2021). However, since the space of symmetric positive definite (SPD) matrices, ${𝒮}_{d}^{++}$, is nonlinear, the natural counterpart of the origin in this space is the identity matrix $I_{d}$. Therefore, we initialize at $S_{0}=I_{d}$. In Appendix I, we use numerical simulations to compare this initialization with random initialization and initialization at the mean, and observe that it consistently performs at least as well as these alternatives. For the step size η, Altschuler et al. (2021) observed through numerical simulations that while the convergence rate of Euclidean gradient descent is highly sensitive to its step size, Riemannian gradient descent requires no tuning and works effectively with η=1 when computing the Bures-Wasserstein barycenter. In our simulations, we also find that η=1 performs at least as well as (and often better than) smaller step sizes. See Appendix I for details.

### 5.2 Simulation setup and results

To validate our theory and demonstrate the practical applicability of our inferential procedures, we conduct a series of numerical experiments with $\rho =n^{-1}$. We start with two illustrative examples that follow the Fréchet regression model. In Example 1, the covariance matrices share a common eigenspace and commute, which effectively reduces the Fréchet regression model to a linear regression model on the square roots. In contrast, Example 2 considers the case where the covariance matrices do not commute.

**Example 1** *Let* $\left\{X_{i}=\left(\begin{array}{lll} X_{i1} & \cdots & X_{ip} \end{array}\right),i\in [n]\right\}$ *be i.i.d. random covariates in* $R^{p}$ *with* $X_{i}\sim \text{Uniform}[-1,1{]}^{p}$. *The response matrices* $Q_{1},\ldots ,Q_{n}\in R^{d\times d}$ *are generated as*:

$$Q_{i}=UV_{i}f{\left(X_{i};\delta \right)}^{2}V_{i}U^{⊤}$$

*where* U *and* ${\left\{X_{i},V_{i}\right\}}_{i\in [n]}$ *are independent, and*:

- $f(\cdot ;\delta ):R^{p}\to R^{d\times d}$ *is a mapping from* $R^{p}$ *to diagonal matrices defined by*:
$$x=\left(\begin{array}{lll} x_{1} & \cdots & x_{p} \end{array}\right)\mapsto f\left(x;\delta \right)={\left(f(x;\delta {)}_{kl}\right)}_{k,l\in \left[d\right]},$$
  *where*
$$f(x;\delta {)}_{kk}=1.5+\frac{k}{2}+\delta \cdot \sum_{j=1}^{p}x_{j},$$
  *with* $\delta \in \left(-2p^{-1},2p^{-1}\right)$ *being a parameter that quantifies the deviation of the model from the null hypothesis* (21).
- $U\in {𝒪}_{d}$ *is a random orthogonal matrix following the Haar measure*.
- $V_{1},\ldots V_{n}\in R^{d}$ *are random diagonal matrices with i.i.d. diagonal entries* $V_{i,kk}\sim \text{Uniform}[-0.1,0.1]$.

*It can be verified that the pair* (X,Q) *satisfies the Fréchet regression model with the conditional expectation* $Q^{*}(\cdot )$ *satisfying*

$$Q^{*}\left(X_{i}\right)=Uf{\left(X_{i}\right)}^{2}U^{⊤}$$

**Example 2** *Let* $X=\left(\begin{array}{lll} X_{1} & \cdots & X_{p} \end{array}\right)$ *be a random covariate in $R^{p}$* with $X\sim \text{Uniform}[-1,1{]}^{p}$. *The response matrix* $Q\in R^{d\times d}$, *where* d *is an even number, is generated as*:

$$Q=UVg(X;\delta {)}^{2}VU^{⊤},$$

*where* U,V *and* X *are independent, and*:

- $g(\cdot ;\delta ):R^{p}\to R^{d\times d}$ is a mapping from $R^{p}$ to diagonal matrices defined by:
$$x=\left(\begin{array}{lll} x_{1} & \cdots & x_{p} \end{array}\right)\mapsto g(x;\delta )={\left(g(x;\delta {)}_{kl}\right)}_{k,l\in [d]},$$
  *where*
$$g(x;\delta {)}_{kk}=1.5+0.5\cdot \lceil k/2\rceil +\delta \cdot \sum_{j=1}^{p}x_{j},$$
  *with* ⌈⋅⌉ *denoting the ceiling function, and* $\delta \in \left(-2p^{-1},2p^{-1}\right)$ *being a parameter that quantifies the deviation of the model from the null hypothesis* (21).
- $U\in {𝒪}_{d}$ *is a random orthogonal matrix with a block*-*diagonal structure*
$$U=\text{diag}\left(U^{\left(1\right)},\ldots ,U^{\left(\lfloor d/2\rfloor \right)}\right)$$
  *where* $U^{\left(1\right)},\ldots ,U^{\left(\lfloor d/2\rfloor \right)}$ *are i.i.d. random* 2 × 2 *orthogonal matrices following the Haar measure*.
- $V\in R^{d}$ *is a diagonal matrix with i.i.d. diagonal entries* $V_{\text{ii}}\sim \text{Uniform}[-0.1,0.1]$.

*It can be verified that the pair* (X,Q) *satisfies the Fréchet regression model with*

$$Q^{*}(x)=g(x{)}^{2}$$

With Example 1 and 2 in hand, we proceed to check the validity of the central limit theorem for ${\hat{Q}}_{\rho }(x)$ as stated in Theorem 11. To this end, we generate random predictor-response pairs (X,Q) based on Example 1 with parameters d=5,p=5 and δ=0; and Example 2 with parameters d=6,p=5 and δ=0. For each trial, we generate n=200 samples of $\left(X_{i},Q_{i}\right)$ and obtain the Fréchet regression estimate ${\hat{Q}}_{\rho }(x)$ using Algorithm 1 with $\rho =n^{-1}$. We then compute normalized error

$$[\tilde{Q}(x){]}_{ij}=\frac{\sqrt{n}{\left[{\hat{Q}}_{\rho }(x)-Q^{*}(x)\right]}_{ij}}{\sqrt{{\hat{v}}_{x,ij}}}$$

where ${\hat{v}}_{x,ij}$ denotes the plug-in estimate for the asymptotic variance of the (i,j)-entry of $\sqrt{n}\left({\hat{Q}}_{\rho }(x)-Q^{*}(x)\right)$, following Theorem 11 and (20). Specifically, the covariance operator $\Xi_{x}$ in Theorem 11 is estimated as follows:

$${\hat{\Xi }}_{x}=\frac{1}{n}\sum_{i=1}^{n}V_{x,i}⊗V_{x,i}$$

where

$$V_{x,i}=V_{x,1,i}+V_{x,2,i}\;V_{x,1,i}=w_{n,\rho }\left(x,X_{i}\right)\left(T_{{\hat{Q}}_{\rho }(x)}^{Q_{i}}-I_{d}\right)\;V_{x,2,i}=-\left({\vec{x}}^{⊤}{\hat{\vec{\Sigma }}}_{\rho }^{-1}\left({\vec{X}}_{i}{\vec{X}}_{i}^{⊤}-\hat{\vec{\Sigma }}\right){\hat{\vec{\Sigma }}}_{\rho }^{-1}⊗I_{d}\right)\cdot \left(\frac{1}{n}\sum_{j=1}^{n}{\vec{X}}_{j}⊗\left(T_{{\hat{Q}}_{\rho }\left(x\right)}^{Q_{j}}-I_{d}\right)\right)$$

The estimated covariance ${\hat{v}}_{x,ij}$ can be extracted from ${\hat{\Xi }}_{x}$ by appropriately indexing its elements.

Figure 1 presents the Q-Q (quantile-quantile) plots of ${\tilde{Q}}_{ij}(x)$ against the standard normal distribution, based on 200 Monte Carlo trials at x=0. According to Theorem 11, ${\tilde{Q}}_{ij}(x)$ should asymptotically follow a standard normal distribution, which would be indicated by a Q-Q plot that exhibits a linear relationship with a slope of one and an intercept of zero. As shown in Figure 1, the empirical quantiles of ${\tilde{Q}}_{ij}(0)$ align closely with the theoretical quantiles of 𝒩(0,1), providing strong empirical support for the validity of Theorem 11.

We then turn our attention to Theorem 16, which characterizes the asymptotic null distribution of the test statistic ${\hat{𝒯}}_{\rho }$, and Theorem 18, which focuses on the power of the test. Figure 2 illustrates the results for Example 1 with parameters n=200,p∈{1,3,5},d∈{5,10}; Figure 3 presents results for Example 2 with parameters n=200,p∈{1,3,5},d∈{6,10}. The Q-Q plots demonstrate a linear fit with slope 1 and intercept 0. Additionally, the figures indicate that as p and d increase, the test statistic also increases. The power of the test quickly approaches 1 as the effect size δ grows. We compare the power of our test with that of the distance covariance test (dcov) (Székely et al., 2007) for testing independence, using the Python dcor package (Ramos-Carreño and Torrecilla, 2023). Given that the distance covariance test is nonparametric, our test is expected to outperform dcov in this context.

In Example 1 and 2 above, the covariate X is supported on the fixed compact domain $[-1,1{]}^{p}$. To illustrate the uniform convergence over a diverging domain $\|x\|≲\sqrt{\text{log}\;n}$ as stated in Theorem 7, we perform additional experiments with X~Uniform $[-\sqrt{\text{log}\;n},\sqrt{\text{log}\;n}{]}^{p}$ for n∈{100,200,400,800,1600}. The maximum in-sample estimation error is computed and is observed to scale approximately as $n^{-0.46}$, providing empirical support for the parametric rate of convergence up to logarithmic terms. For more details, see Appendix I.3.

### 5.3 Robustness of the results when covariance matrices are estimated

In this section, we examine the numerical performance of our results in a setting where Q is not directly observed but is instead estimated from the data. Specifically, consider the case where we have access only to ${\left\{\left(X_{i};Z_{i1},Z_{i2},\ldots ,Z_{i\tilde{n}}\right)\right\}}_{i\in [n]}$ with $Z_{i1},Z_{i2},\ldots ,Z_{i\tilde{n}}\overset{i.i.d.}{\sim }𝒩\left(0,Q_{i}\right)$. In this scenario, a natural plug-in approach for Fréchet estimation and testing is to estimate $Q_{i}$ using the sample covariance, defined as ${\overset{‾}{Q}}_{i}≔{\tilde{n}}^{-1}\sum_{j=1}^{\tilde{n}}Z_{i1}Z_{i1}^{⊤}$. This estimate ${\overset{‾}{Q}}_{i}$ is then substituted for $Q_{i}$ in downstream estimators. Specifically, we define the estimator ${\hat{Q}}_{\rho ,\tilde{n}}(x)$ and test statistic ${\hat{𝒯}}_{\rho ,\tilde{n}}$ with $\rho =n^{-1}$ as follows.

$${\hat{Q}}_{\rho ,\tilde{n}}(x)=\underset{S\in {𝒮}_{d}^{++}}{\text{argmin}}\frac{1}{n}\sum_{i=1}^{n}w_{n,\rho }\left(x,X_{i}\right)W^{2}\left(S,{\overset{‾}{Q}}_{i}\right)\;{\hat{𝒯}}_{\rho ,\tilde{n}}=\sum_{i=1}^{n}{\left\|{\hat{H}}_{\rho ,\tilde{n}}\cdot \left({\hat{Q}}_{\rho ,\tilde{n}}\left(X_{i}\right)-{\hat{Q}}_{\rho ,\tilde{n}}(\overset{‾}{X})\right)\right\|}_{\text{F}}^{2},\;\text{where}\;{\hat{H}}_{\rho ,\tilde{n}}=-\frac{1}{n}\sum_{i=1}^{n}dT_{{\hat{Q}}_{\rho ,\tilde{n}}(\overset{‾}{X})}^{{\overset{‾}{Q}}_{i}}$$

The asymptotic null distribution of ${\hat{𝒯}}_{\rho ,\tilde{n}}$ is computed as $\sum_{j}{\hat{\lambda }}_{\tilde{n},j}w_{i}$ where $w_{i}$ are i.i.d. $\chi_{p}^{2}$ random variables and ${\hat{\lambda }}_{\tilde{n},j}$ are the eigenvalues of the following estimated operator:

$$\frac{1}{n}\sum_{i=1}^{n}\left(T_{{\hat{Q}}_{\rho ,\tilde{n}}(\overset{‾}{X})}^{{\overset{‾}{Q}}_{i}}-I_{d}\right)⊗\left(T_{{\hat{Q}}_{\rho ,\tilde{n}}(\overset{‾}{X})}^{{\overset{‾}{Q}}_{i}}-I_{d}\right)$$

We denote by ${\hat{q}}_{1-\alpha ,\tilde{n}}$ the 1−α quantile of $\sum_{j}{\hat{\lambda }}_{\tilde{n},j}w_{i}$. This quantile is then used to construct a test:

$$\Phi_{\rho ,\tilde{n},\alpha }=1\left({\hat{𝒯}}_{\rho ,\tilde{n}}>{\hat{q}}_{1-\alpha ,\tilde{n}}\right)$$

Similarly, the covariance operator ${\hat{\Xi }}_{x,\tilde{n}}$ is computed as

(28) $${\hat{\Xi }}_{x,\tilde{n}}=\frac{1}{n}\sum_{i=1}^{n}V_{x,\tilde{n},i}⊗V_{x,\tilde{n},i}$$

where

$$V_{x,i}=V_{x,\tilde{n},1,i}+V_{x,\tilde{n},2,i}\;V_{x,\tilde{n},1,i}=w_{n,\rho }\left(x,X_{i}\right)\left(T_{{\hat{Q}}_{\rho ,\tilde{n}}(x)}^{{\overset{‾}{Q}}_{i}}-I_{d}\right)\;V_{x,\tilde{n},2,i}=-\left({\vec{x}}^{⊤}{\hat{\vec{\Sigma }}}_{\rho }^{-1}\left({\vec{X}}_{i}{\vec{X}}_{i}^{⊤}-\hat{\vec{\Sigma }}\right){\hat{\vec{\Sigma }}}_{\rho }^{-1}⊗I_{d}\right)\cdot \left(\frac{1}{n}\sum_{j=1}^{n}{\vec{X}}_{j}⊗\left(T_{{\hat{Q}}_{\rho ,\tilde{n}}(x)}^{{\overset{‾}{Q}}_{j}}-I_{d}\right)\right)$$

First, we examine the central limit theorem (Theorem 11) for ${\left(X_{i},Q_{i}\right)}_{i\in [n]}$ satisfying the conditions in Example 1 with parameters $\tilde{n}\in \{50,100,+\infty \},n=200,p=5,d=5$ and δ=0. Here $\tilde{n}=+\infty$ indicates $Q_{i}$ is directly observed. Using the estimated covariance operator ${\hat{\Xi }}_{x,\tilde{n}}$ defined in (28), we define the normalized error as:

$${\left[{\tilde{Q}}_{\rho ,\tilde{n}}(x)\right]}_{i,j}≔\frac{\sqrt{n}{\left[{\hat{Q}}_{\rho ,\tilde{n}}(x)-Q^{*}(x)\right]}_{ij}}{\sqrt{{\hat{v}}_{x,\tilde{n};ij}}}$$

where ${\hat{v}}_{x,\tilde{n};ij}$ is extracted from ${\hat{\Xi }}_{x,\tilde{n}}$ with appropriate indexing. Figure 4 shows the Q-Q plots of ${\left[{\tilde{Q}}_{n,\tilde{n}}(x)\right]}_{ij}$ against the standard normal distribution. Generally, as $\tilde{n}\to \infty$, the Q-Q plots for finite $\tilde{n}$ approach those of $\tilde{n}=\infty$, and can still be approximated by a straight line. This observation suggests that the central limit theorem for the entries still holds. Interestingly, the detailed behavior of the bias differs between diagonal and off-diagonal entries. For off-diagonal entries, Figure 4b indicates that $\left[{\hat{Q}}_{\rho ,\tilde{n}}(0)\right]ij$ is unbiased. In contrast, for diagonal entries, Figure 4a reveals that the Q-Q plots for finite $\tilde{n}$ are negatively shifted compared to that of $\tilde{n}=\infty (\text{blue})$, suggesting that ${\left[{\hat{Q}}_{\rho ,\tilde{n}}(0)\right]}_{ii}$ is negatively biased. In this paper, we focus on the theoretical properties of the Fréchet regression estimator (15) in the setting where covariance matrices are directly observed. We leave a detailed theoretical investigation of its refined behavior in the setting where covariance matrices are estimated for future work.

Figure 5 shows the Q-Q plot of ${\hat{𝒯}}_{\rho ,\tilde{n}}$ against $\sum_{j}{\hat{\lambda }}_{\tilde{n},j}w_{i}$ and the power of the test $\Phi_{\rho ,\tilde{n},\alpha }$ for $\left(X_{i},Q_{i}\right)$ satisfying the conditions of Example 1 with parameters $\tilde{n}\in \{50,100,+\infty \}$, n=200,p=5 and d=5. Notably, the Q-Q plots exhibit a linear relationship with slope 1 and intercept 0, indicating that ${\hat{𝒯}}_{\rho ,\tilde{n}}$ follows the asymptotic null distribution $\sum_{j}\lambda_{\tilde{n},j}w_{i}$, where $w_{i}$ are i.i.d. $\chi_{p}^{2}$ random variables and $\lambda_{\tilde{n},j}$ are the eigenvalues of the following operator:

$$E\left[\left(T_{Q^{*}(\mu )}^{\overset{‾}{Q}}-I_{d}\right)⊗\left(T_{Q^{*}(\mu )}^{\overset{‾}{Q}}-I_{d}\right)\right]$$

where $\overset{‾}{Q}=\text{Cov}Z$. This observation suggests that the plug-in method remains valid in this context. The power plot indicates that while the power of the test decreases when $Q_{i}$ is not directly observed, our test still demonstrates higher power compared to the distance covariance test (dcov) in this setting.

## 6 Application to Single-cell Gene Co-expression Networks

Aging is a complex process of accumulation of molecular, cellular, and organ damage, leading to loss of function and increased vulnerability to disease and death. Nutrient-sensing pathways, namely insulin/insulin-like growth factor signaling and target-of-rapamycin can substantially increase healthy life span of laboratory model organisms (Davinelli et al., 2012; de Lucia et al., 2020). These nutrient signaling pathways are conserved in various organisms. We are interested in understanding the co-expression structure of 61 genes in this KEGG nutrient-sensing pathways based on the recently published population scale single cell RNA-seq data of human peripheral blood mononuclear cells (PBMCs) from blood samples of over 982 healthy individuals with ages ranging from 20 to 90 (Yazar et al., 2022).

We focus our analysis on CD4+ naive and central memory T (CD4NC) cells, which is the most common cell type observed in the data. Age-associated changes in CD4 T-cell functionality have been linked to chronic inflammation and decreased immunity (Elyahu et al., 2019). There are a total of 51 genes that are expressed in this cell type. Even though the Fréchet regression still makes sense when the covariance matrix is potentially degenerate (see the remarks after Example 2), our theory relies on the strict positive definiteness. Hence, we retain only the genes that have nonzero variances at any age, resulting in a total of 37 genes, see Figure 6 for a concise overview of these covariance matrices for individuals at different ages, showing difference across different ages.

In genetics research, such covariance matrices represent individual-specific gene co-expression networks. We are interested in testing whether such networks are associated with ages by testing whether there is an age effect on the gene expression covariance matrices, i.e. ${ℋ}_{0}:Q^{*}(t)\equiv Q^{*}$ for some $Q^{*}$. The test we propose yields a p-value of 0.00019. When the analysis is performed separately for males and females, we obtain a p-value of 0.00034 for the male group and 0.00012 for the female group, suggesting a strong age effect on the gene expression covariance matrices.

## 7 Discussion

The present paper provides a framework for statistical inference in the context of Fréchet regression on the Bures-Wasserstein manifold, where covariance matrices serve as outcomes. Our contributions include establishing uniform convergence rates for estimating the conditional Fréchet mean and deriving the asymptotic distribution of the estimator. Leveraging these results, we further develop a hypothesis test to rigorously assess associations between Euclidean covariates and covariance matrix-valued outcomes. The finite-sample performance of our methods is validated through numerical simulations, and their practical utility is demonstrated in an application to gene co-expression analysis. Specifically, we test the association between age and co-expression patterns in the nutrient sensing pathway, revealing how these patterns evolve with age.

Beyond genomics, our framework has broad applicability in neuroimaging, where covariance matrices (or correlation matrices after standardization) of multiple brain regions are used to summarize as functional connectivity matrices. The proposed methods can be used to identify the factors that are associated with such functional connectivity matrices.

In this work, we focus on the theoretical analysis of the Fréchet regression estimator (15) under the assumption that outcome covariance matrices are directly observed. This setting aligns with prior frameworks in Petersen and Müller (2019) and Petersen et al. (2021). While our empirical results suggest the proposed hypothesis test remains valid when covariance matrices must first be estimated from data, formal theoretical guarantees for this setting remain an open problem. Additionally, relaxing the eigenvalue lower-bound assumption to accommodate singular covariance matrices would broaden the applicability of our framework, particularly in high-dimensional or sparse-data regimes.

## Supplementary Material

1

## Figures and Tables

**Figure 1:**
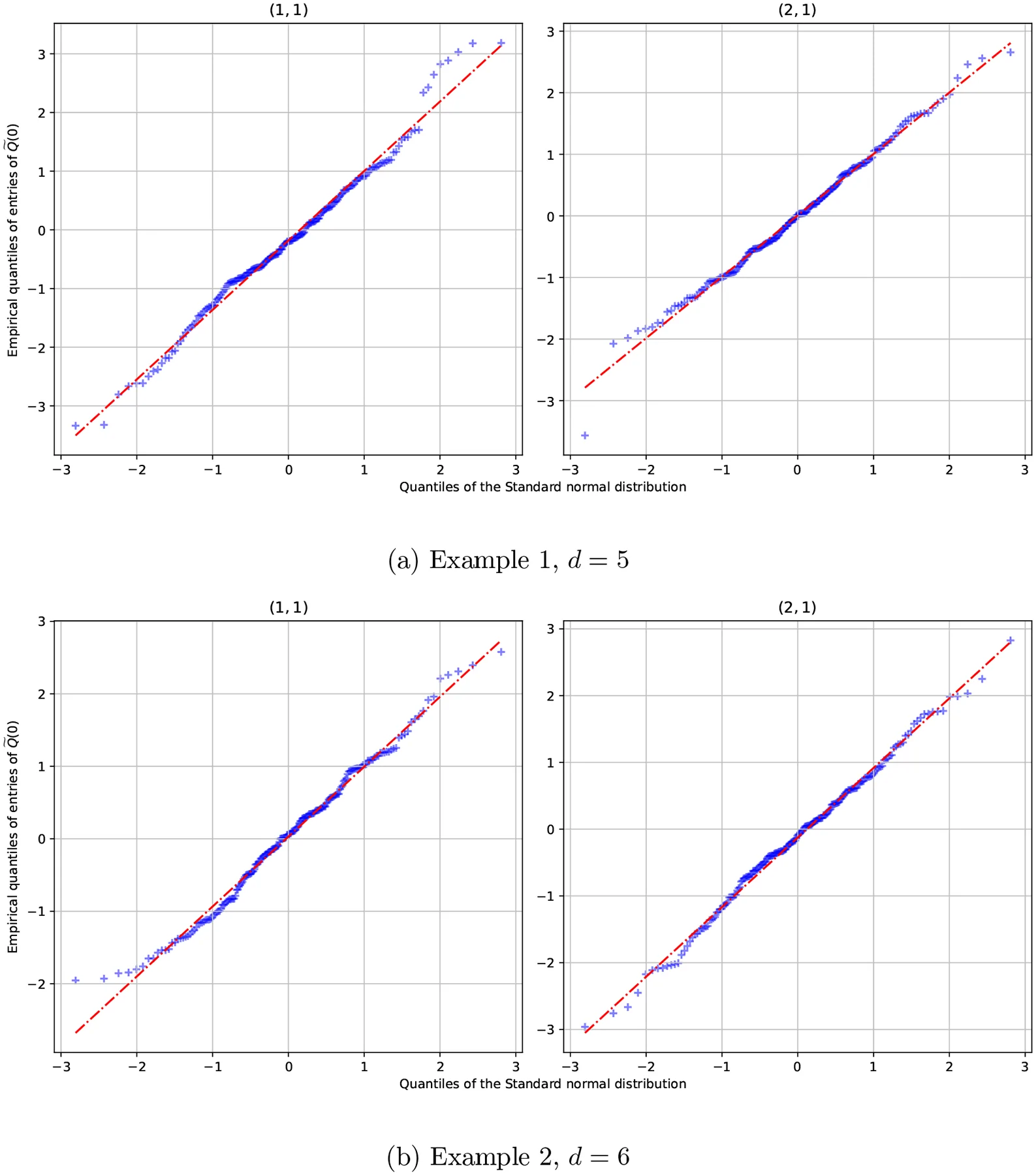
Q-Q plots of ${\tilde{Q}}_{11}(0)$ and ${\tilde{Q}}_{21}(0)$ with parameters p=5,δ=0,n=200. (a) Example 1, d=5; (b) Example 2, d=6.

**Figure 2:**
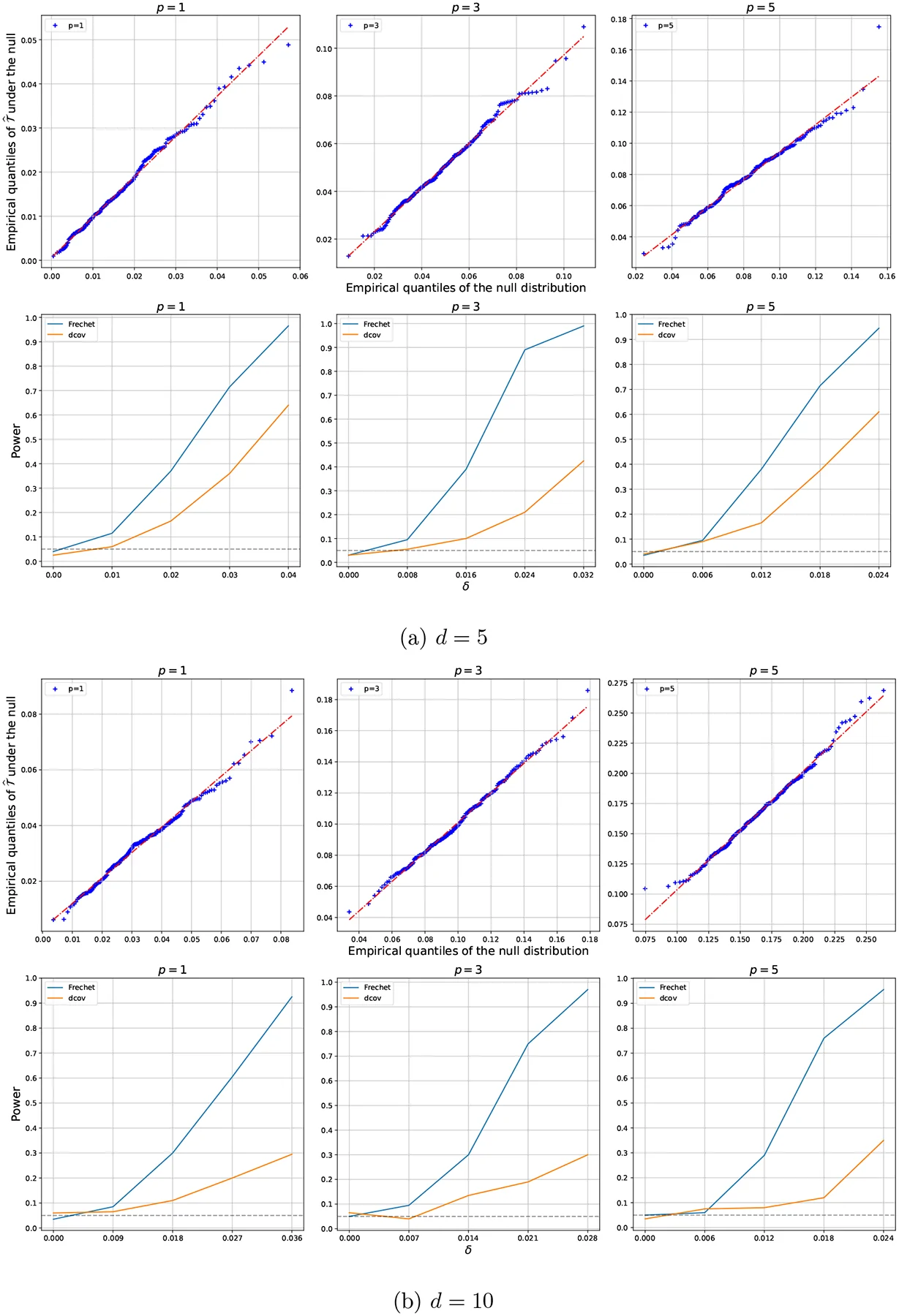
Q-Q plots of the test statistic ${\hat{𝒯}}_{\rho }$ against its asymptotic null distribution and power curves compared to dcov as a function of the effect size δ for Example 1, with parameters n=200,p∈{1,3,5}. (a) d=5; (b) d=10.

**Figure 3:**
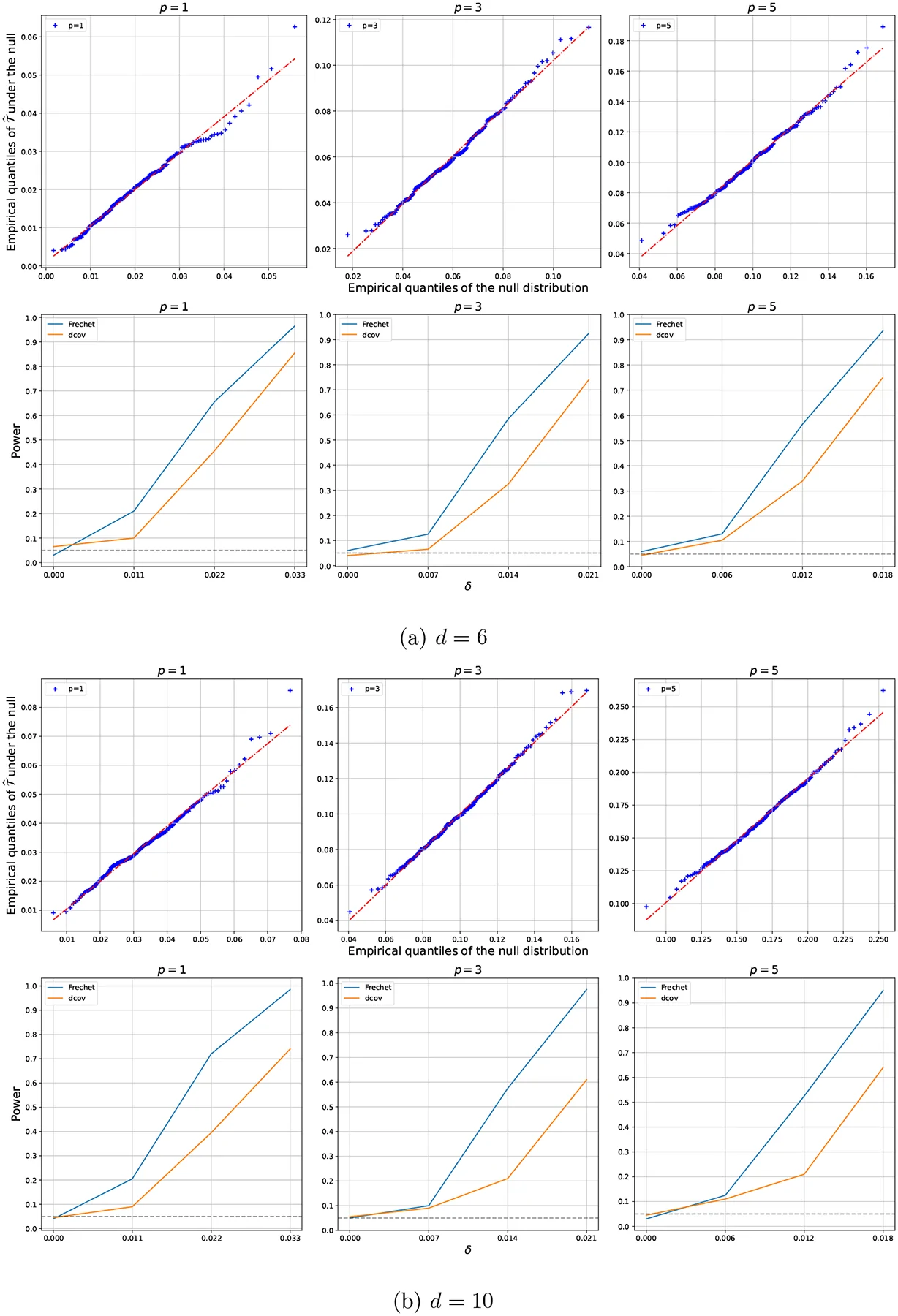
Q-Q plots of the test statistic ${\hat{𝒯}}_{\rho }$ against its asymptotic null distribution and power curves compared to dcov as a function of the effect size δ for Example 2, with parameters n=200,p∈{1,3,5}. (a) d=6; (b) d=10.

**Figure 4:**
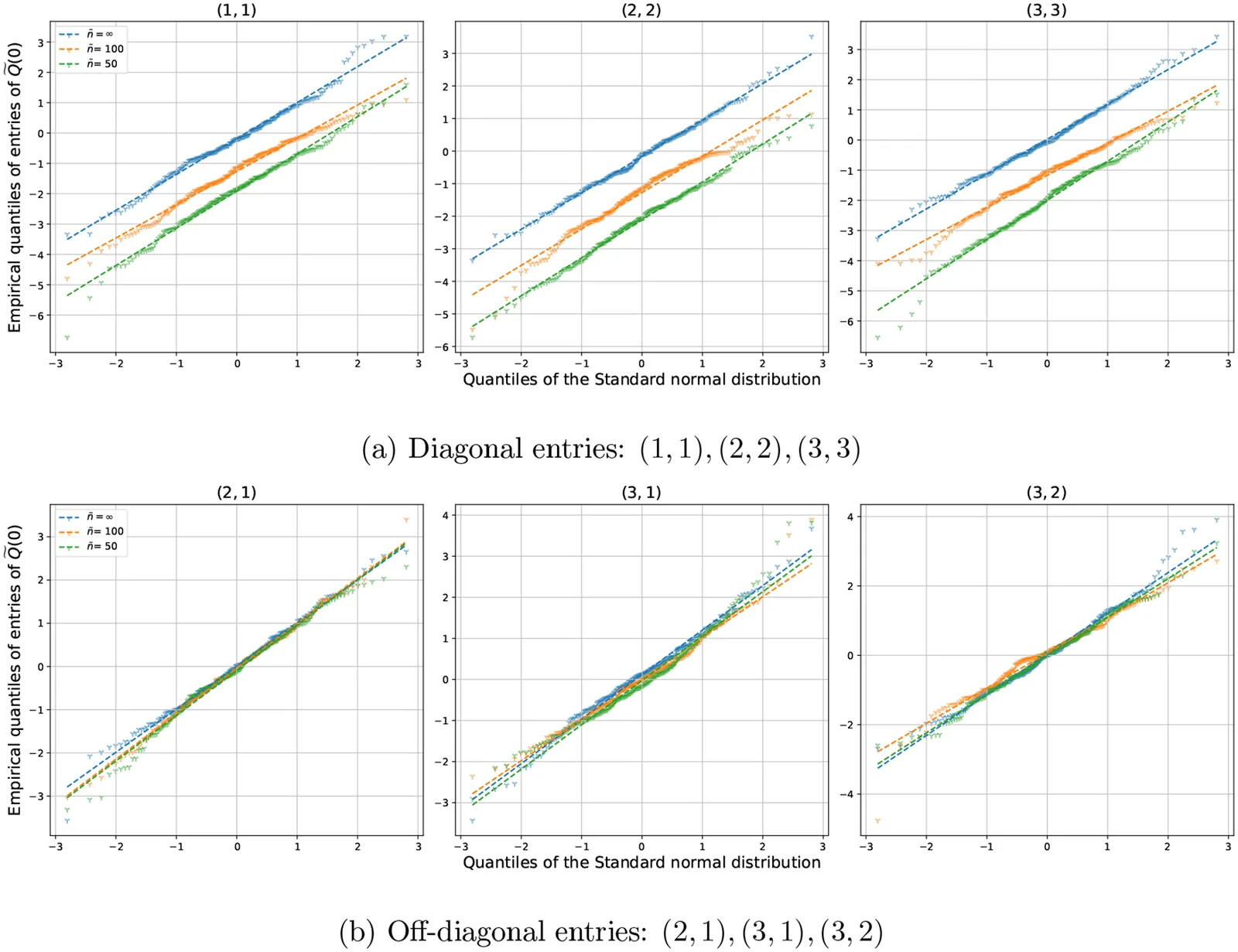
Q-Q plots of diagonal and off-diagonal entries of ${\tilde{Q}}_{\rho ,\tilde{n}}(0)$ with parameters d=5,n=200 and varying $\tilde{n}\in \{50,100,\infty \}$.

**Figure 5:**
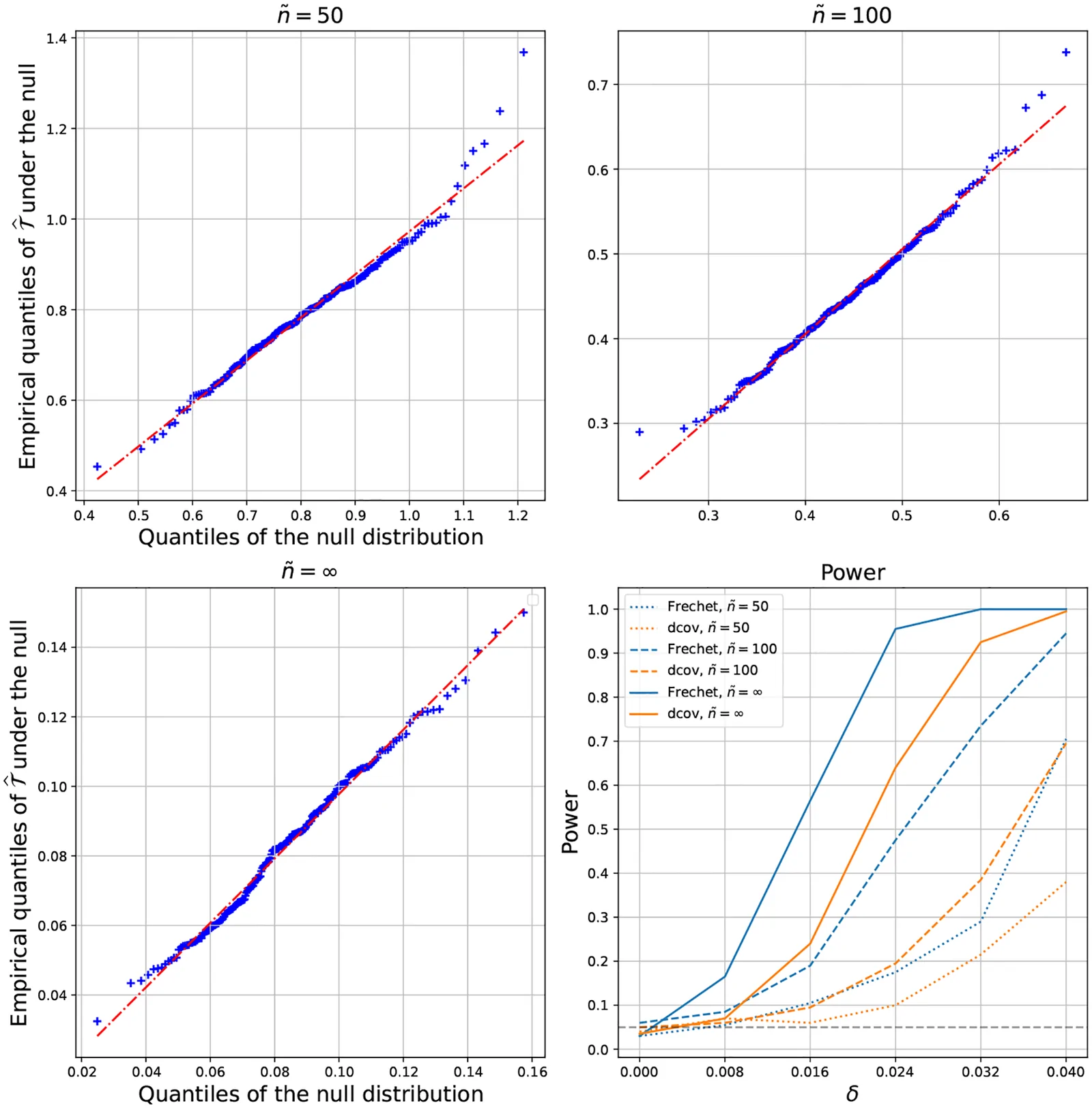
Q-Q plots of the test statistic ${\hat{𝒯}}_{\rho ,\tilde{n}}$ against the estimated plug-in null distribution $\sum_{j}{\hat{\lambda }}_{\tilde{n},j}w_{i}$ and power curves of $\Phi_{\rho ,\tilde{n},\alpha }$ compared to dcov as a function of the effect size δ.

**Figure 6:**
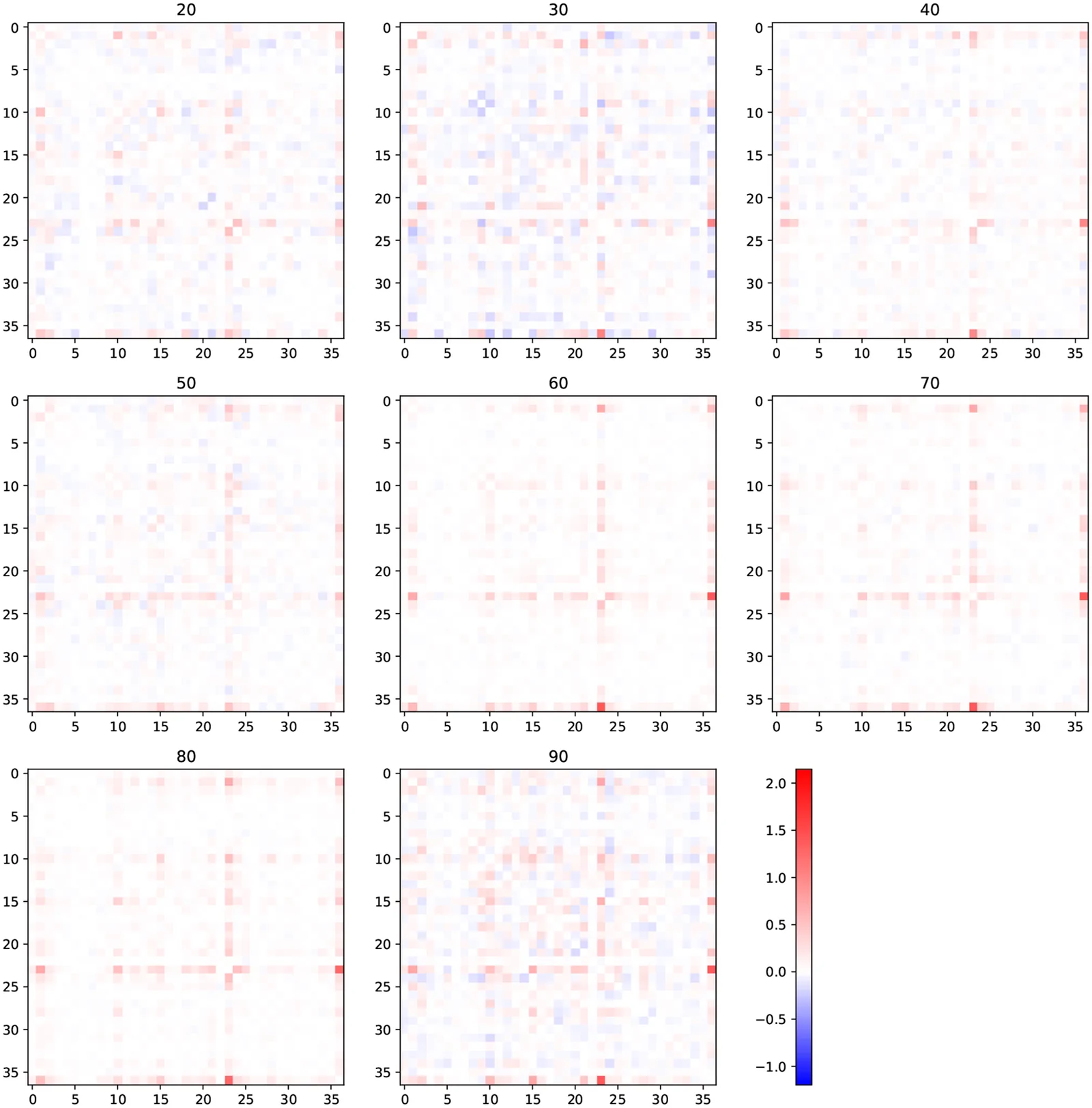
Heatmap of the gene expression covariance matrices with diagonal elements omitted for individual at age 20, 30,…, 90, respectively.

## References

[R1] Abadie A and Imbens GW. Large Sample Properties of Matching Estimators for Average Treatment Effects. Econometrica, 74(1):235–267, 2006.

[R2] Agueh M and Carlier G. Barycenters in the Wasserstein Space. SIAM Journal on Mathematical Analysis, 43(2):904–924, 2011.

[R3] Agueh M and Carlier G. Vers un théorème de la limite centrale dans l’espace de Wasserstein? Comptes Rendus. Mathématique, 355(7):812–818, 2017.

[R4] Altschuler J, Niles-Weed J, and Rigollet P. Near-linear time approximation algorithms for optimal transport via Sinkhorn iteration. Advances in neural information processing systems, 30, 2017.

[R5] Altschuler J, Chewi S, Gerber PR, and Stromme AJ. Averaging on the Bures-Wasserstein manifold: dimension-free convergence of gradient descent. Advances in neural information processing systems, 34:22132–22145, 2021.

[R6] Altschuler JM, Niles-Weed J, and Stromme AJ. Asymptotics for Semidiscrete Entropic Optimal Transport. SIAM Journal on Mathematical Analysis, 54(2):1718–1741, 2022.

[R7] Ambrosio L, Gigli N, and Savaré G. Gradient Flows In Metric Spaces and in the Space of Probability Measures. Birkhäuser-Verlag, 2005.

[R8] Arjovsky M, Chintala S, and Bottou L. Wasserstein Generative Adversarial Networks. In Proceedings of the 34th International Conference on Machine Learning, pages 214–223. PMLR, 2017.

[R9] Arnaudon M, Barbaresco F, and Yang L. Riemannian medians and means with applications to radar signal processing. IEEE Journal of Selected Topics in Signal Processing, 7(4):595–604, 2013.

[R10] Arsigny V, Fillard P, Pennec X, and Ayache N. Geometric means in a novel vector space structure on symmetric positive-definite matrices. SIAM Journal on Matrix Analysis and Applications, 29(1):328–347, 2007.

[R11] Barrio E. d. and Loubes J-M. Central limit theorems for empirical transportation cost in general dimension. The Annals of Probability, 47(2):926–951, 2019.

[R12] Bhatia R, Jain T, and Lim Y. On the Bures–Wasserstein distance between positive definite matrices. Expositiones Mathematicae, 37(2):165–191, 2019.

[R13] Bhattacharya R and Patrangenaru V. Large sample theory of intrinsic and extrinsic sample means on manifolds. The Annals of Statistics, 31(1):1–29, 2003.

[R14] Bhattacharya R and Patrangenaru V. Large sample theory of intrinsic and extrinsic sample means on manifolds—II. The Annals of Statistics, 33(3):1225–1259, 2005.

[R15] Bigot J, Gouet R, Klein T, and López A. Geodesic PCA in the Wasserstein space by convex PCA. Annales de l’Institut Henri Poincaré, Probabilités et Statistiques, 53(1):1–26, 2017.

[R16] Bunne C, Papaxanthos L, Krause A, and Cuturi M. Proximal Optimal Transport Modeling of Population Dynamics. In Proceedings of The 25th International Conference on Artificial Intelligence and Statistics, pages 6511–6528. PMLR, 2022.

[R17] Bunne C, Hsieh Y-P, Cuturi M, and Krause A. The Schrödinger Bridge between Gaussian Measures has a Closed Form. In Proceedings of The 26th International Conference on Artificial Intelligence and Statistics, pages 5802–5833. PMLR, 2023a.

[R18] Bunne C, Stark SG, Gut G, del Castillo JS, Levesque M, Lehmann K-V, Pelkmans L, Krause A, and Rätsch G. Learning single-cell perturbation responses using neural optimal transport. Nature Methods, 20(11):1759–1768, 2023b. 10.1038/s41592-023-01969-x PMC10630137 37770709

[R19] Bures D. An extension of Kakutani’s theorem on infinite product measures to the tensor product of semifinite *w**-algebras. Transactions of the American Mathematical Society, 135(0):199–212, 1969.

[R20] Cai TT, Zhang C-H, and Zhou HH. Optimal rates of convergence for covariance matrix estimation. The Annals of Statistics, 38(4):2118–2144, 2010.

[R21] Cai TT, Liu W, and Zhou HH. Estimating sparse precision matrix: Optimal rates of convergence and adaptive estimation. The Annals of Statistics, 44(2):455 – 488, 2016.

[R22] Carlsson M. Perturbation theory for the matrix square root and matrix modulus, 2018. arXiv:1810.01464.

[R23] Caseiro R, Martins P, Henriques JF, and Batista J. A nonparametric Riemannian framework on tensor field with application to foreground segmentation. Pattern Recognition, 45(11):3997–4017, 2012.

[R24] Chen Y and Müller H-G. Uniform convergence of local Fréchet regression with applications to locating extrema and time warping for metric space valued trajectories. The Annals of Statistics, 50(3):1573–1592, 2022.

[R25] Chen Y, Chi Y, Fan J, and Ma C. Gradient descent with random initialization: fast global convergence for nonconvex phase retrieval. Mathematical Programming, 176(1): 5–37, July 2019. ISSN 1436–4646. 10.1007/s10107-019-01363-6 PMC8025800 33833473

[R26] Chen Y, Lin Z, and Müller H-G. Wasserstein Regression. Journal of the American Statistical Association, 118(542):869–882, 2023. 10.1080/01621459.2021.1957900 PMC10259833 37309513

[R27] Chewi S, Maunu T, Rigollet P, and Stromme AJ. Gradient descent algorithms for Bures-Wasserstein barycenters. In Proceedings of Thirty Third Conference on Learning Theory, pages 1276–1304. PMLR, 2020.

[R28] Chiu TYM, Leonard T, and Tsui K-W. The Matrix-Logarithmic Covariance Model. Journal of the American Statistical Association, 91(433):198–210, 1996.

[R29] Cornea E, Zhu H, Kim P, and Ibrahim JG. Regression models on Riemannian symmetric spaces. Journal of the Royal Statistical Society Series B: Statistical Methodology, 79(2): 463–482, 2017. 10.1111/rssb.12169 PMC5433528 28529445

[R30] Cuturi M. Sinkhorn Distances: Lightspeed Computation of Optimal Transport. In Burges CJ, Bottou L, Welling M, Ghahramani Z, and Weinberger KQ, editors, Advances in Neural Information Processing Systems, volume 26, 2013.

[R31] Davinelli S, Willcox D, and Scapagnini G. Extending healthy ageing: nutrient sensitive pathway and centenarian population. Immunity & Ageing, 9:9, 2012. 10.1186/1742-4933-9-9 PMC3379947 22524452

[R32] de Lucia C, Murphy T, Steves C, and Lifestyle mediates the role of nutrient-sensing pathways in cognitive aging: cellular and epidemiological evidence. Communications Biology, 3:157, 2020. 10.1038/s42003-020-0844-1 PMC7118127 32242137

[R33] Deb N and Sen B. Multivariate Rank-Based Distribution-Free Nonparametric Testing Using Measure Transportation. Journal of the American Statistical Association, 118(541): 192–207, 2023.

[R34] del Barrio E, Sanz AG, Loubes J-M, and Niles-Weed J. An Improved Central Limit Theorem and Fast Convergence Rates for Entropic Transportation Costs. SIAM Journal on Mathematics of Data Science, 5(3):639–669, 2023.

[R35] Del Moral P and Niclas A. A Taylor expansion of the square root matrix function. Journal of Mathematical Analysis and Applications, 465(1):259–266, 2018.

[R36] Dryden IL, Koloydenko A, and Zhou D. Non-Euclidean statistics for covariance matrices, with applications to diffusion tensor imaging. The Annals of Applied Statistics, 3(3):1102–1123, 2009.

[R37] Dudley RM and Norvaiša R. Concrete Functional Calculus. Springer, New York, NY, 2011.

[R38] Elyahu Y, Hekselman I, Eizenberg-Magar I, Berner O, Strominger I, Schiller M, Mittal K, Nemirovsky A, Eremenko E, Vital A, Simonovsky E, Chalifa-Caspi V, Friedman N, Yeger-Lotem E, and Monsonego A. Aging promotes reorganization of the CD4 T cell landscape toward extreme regulatory and effector phenotypes. Science Advances, 5(8):eaaw8330, 2019. 10.1126/sciadv.aaw8330 PMC6703865 31457092

[R39] Fillard P, Arsigny V, Pennec X, Hayashi KM, Thompson PM, and Ayache N. Measuring brain variability by extrapolating sparse tensor fields measured on sulcal lines. Neuroimage, 34(2):639–650, 2007. 10.1016/j.neuroimage.2006.09.027 17113311

[R40] Friston KJ. Functional and effective connectivity: a review. Brain Connectivity, 1(1): 13–36, 2011. 10.1089/brain.2011.0008 22432952

[R41] Gonzalez-Sanz A, Loubes J-M, and Niles-Weed J. Weak limits of entropy regularized Optimal Transport; potentials, plans and divergences, 2022. arXiv:2207.07427.

[R42] Hallin M, Mordant G, and Segers J. Multivariate goodness-of-fit tests based on Wasserstein distance. Electronic Journal of Statistics, 15(1):1328–1371, 2021.

[R43] Hoff PD and Niu X. A covariance regression model. Statistica Sinica, pages 729–753, 2012.

[R44] Hsing T and Eubank R. Theoretical foundations of functional data analysis, with an introduction to linear operators, volume 997. John Wiley & Sons, 2015.

[R45] Hu W, Pan T, Kong D, and Shen W. Nonparametric matrix response regression with application to brain imaging data analysis. Biometrics, 77(4):1227–1240, 2021. 10.1111/biom.13362 32869275

[R46] Hütter J-C and Rigollet P. Minimax estimation of smooth optimal transport maps. The Annals of Statistics, 49(2):1166–1194, 2021.

[R47] Jin C, Netrapalli P, Ge R, Kakade SM, and Jordan MI. A Short Note on Concentration Inequalities for Random Vectors with SubGaussian Norm, 2019. arXiv:1902.03736.

[R48] Kim Y-H and Pass B. Wasserstein barycenters over Riemannian manifolds. Advances in Mathematics, 307:640–683, 2017.

[R49] Kong D, An B, Zhang J, and Zhu H. L2rm: Low-rank linear regression models for high-dimensional matrix responses. Journal of the American Statistical Association, 115(529): 403–424, 2020. 10.1080/01621459.2018.1555092 PMC7781207 33408427

[R50] Kroshnin A, Spokoiny V, and Suvorikova A. Statistical inference for Bures–Wasserstein barycenters. The Annals of Applied Probability, 31(3):1264–1298, 2021.

[R51] Le Gouic T and Loubes J-M. Existence and consistency of Wasserstein barycenters. Probability Theory and Related Fields, 168(3–4):901–917, 2017.

[R52] Le Gouic T, Paris Q, Rigollet P, and Stromme AJ. Fast convergence of empirical barycenters in Alexandrov spaces and the Wasserstein space. Journal of the European Mathematical Society, 25(6):2229–2250, 2022.

[R53] Lin Z. Riemannian Geometry of Symmetric Positive Definite Matrices via Cholesky Decomposition. SIAM Journal on Matrix Analysis and Applications, 40(4):1353–1370, 2019.

[R54] Lin Z, Müller H-G, and Park BU. Additive models for symmetric positive-definite matrices and Lie groups. Biometrika, 110(2):361–379, 2023.

[R55] Ma R, Sun ED, Donoho D, and Zou J. Principled and interpretable alignability testing and integration of single-cell data. Proceedings of the National Academy of Sciences, 121 (10):e2313719121, 2024. 10.1073/pnas.2313719121 PMC10927515 38416677

[R56] Manole T, Balakrishnan S, Niles-Weed J, and Wasserman L. Plugin Estimation of Smooth Optimal Transport Maps, 2022. arXiv:2107.12364.

[R57] Manole T, Balakrishnan S, Niles-Weed J, and Wasserman L. Central Limit Theorems for Smooth Optimal Transport Maps, 2023. arXiv:2312.12407.

[R58] Mena G and Niles-Weed J. Statistical bounds for entropic optimal transport: sample complexity and the central limit theorem. Advances in neural information processing systems, 32, 2019.

[R59] Ohta S-I. Barycenters in Alexandrov spaces of curvature bounded below. Advances in Geometry, 12(4):571–587, 2012.

[R60] Panaretos VM and Zemel Y. Amplitude and phase variation of point processes. The Annals of Statistics, 44(2):771–812, 2016.

[R61] Panaretos VM and Zemel Y. An Invitation to Statistics in Wasserstein Space. Springer Nature, 2020.

[R62] Petersen A and Müller H-G. Fréchet regression for random objects with Euclidean predictors. The Annals of Statistics, 47(2):691–719, 2019.

[R63] Petersen A, Liu X, and Divani AA. Wasserstein F-tests and confidence bands for the Fréchet regression of density response curves. The Annals of Statistics, 49(1):590–611, 2021.

[R64] Peyré G and Cuturi M. Computational Optimal Transport: With Applications to Data Science. Foundations and Trends^®^ in Machine Learning, 11(5–6):355–607, 2019.

[R65] Pooladian A-A and Niles-Weed J. Entropic estimation of optimal transport maps, May 2022. arXiv:2109.12004.

[R66] Pooladian A-A, Divol V, and Niles-Weed J. Minimax estimation of discontinuous optimal transport maps: The semi-discrete case. In International Conference on Machine Learning, pages 28128–28150. PMLR, 2023.

[R67] Ramos-Carreño C and Torrecilla JL. dcor: Distance correlation and energy statistics in Python. SoftwareX, 22, 2 2023. doi: 10.1016/j.softx.2023.101326.

[R68] Redko I, Habrard A, and Sebban M. Theoretical Analysis of Domain Adaptation with Optimal Transport. In Ceci M, Hollmén J, Todorovski L, Vens C, and Džeroski, editors, Machine Learning and Knowledge Discovery in Databases, pages 737–753, 2017.

[R69] Santambrogio F. Optimal Transport for Applied Mathematicians: Calculus of Variations, PDEs, and Modeling, volume 87. Springer, NY, 2015.

[R70] Schiebinger G, Shu J, Tabaka M, Cleary B, Subramanian V, Solomon A, Gould J, Liu S, Lin S, Berube P, Lee L, Chen J, Brumbaugh J, Rigollet P, Hochedlinger K, Jaenisch R, Regev A, and Lander ES. Optimal-Transport Analysis of Single-Cell Gene Expression Identifies Developmental Trajectories in Reprogramming. Cell, 176(4): 928–943, 2019. 10.1016/j.cell.2019.01.006 PMC6402800 30712874

[R71] Somnath VR, Pariset M, Hsieh Y-P, Martinez MR, Krause A, and Bunne C. Aligned Diffusion Schrödinger Bridges. In Proceedings of the Thirty-Ninth Conference on Uncertainty in Artificial Intelligence, volume 216, pages 1985–1995. PMLR, 2023.

[R72] Székely GJ, Rizzo ML, and Bakirov NK. Measuring and testing dependence by correlation of distances. The Annals of Statistics, 35(6):2769 – 2794, 2007.

[R73] Talagrand M. Isoperimetry and Integrability of the Sum of Independent Banach-Space Valued Random Variables. The Annals of Probability, 17(4):1546–1570, 1989.

[R74] Tony Cai WL and Luo X. A constrained l1 minimization approach to sparse precision matrix estimation. Journal of the American Statistical Association, 106(494):594–607, 2011.

[R75] van der Vaart AW. Asymptotic Statistics. Cambridge Series in Statistical and Probabilistic Mathematics. Cambridge University Press, 1998.

[R76] Van Der Vaart AW and Wellner JA. Weak Convergence and Empirical Processes. Springer Series in Statistics. Springer, New York, NY, 1996.

[R77] Vershynin R. High-Dimensional Probability: An Introduction with Applications in Data Science. Cambridge University Press, Cambridge, 2018.

[R78] Villani C. Topics in Optimal Transportation. American Mathematical Society, 2003.

[R79] Villani C. Optimal Transport: Old and New, volume 338. Springer, Berlin, Heidelberg, 2009.

[R80] Wainwright MJ. High-Dimensional Statistics: A Non-Asymptotic Viewpoint. Cambridge University Press, 2019.

[R81] Wasserman L. All of Nonparametric Statistics. Springer Texts in Statistics. Springer, New York, NY, 2006. ISBN 978-0-387-25145-5. doi: 10.1007/0-387-30623-4.

[R82] Weed J and Berthet Q. Estimation of smooth densities in Wasserstein distance. In conference on Learning Theory, pages 3118–3119. PMLR, 2019.

[R83] Wihler TP. On the Hölder continuity of matrix functions for normal matrices. Journal of inequalities in pure and applied mathematics, 10(10), 2009.

[R84] Yazar S, Alquicira-Hernandez J, Wing K, Senabouth A, Gordon MG, Andersen S, Lu Q, Rowson A, Taylor TRP, Clarke L, Maccora K, Chen C, Cook AL, Ye CJ, Fairfax KA, Hewitt AW, and Powell JE. Single-cell eqtl mapping identifies cell type–specific genetic control of autoimmune disease. Science, 376(6589):eabf3041, 2022. 10.1126/science.abf3041 35389779

[R85] Ye T and Du SS. Global convergence of gradient descent for asymmetric low-rank matrix factorization. In Advances in Neural Information Processing Systems, 2021.

[R86] Yokota T. Convex functions and barycenter on CAT (1)-spaces of small radii. Journal of the Mathematical Society of Japan, 68(3):1297–1323, 2016.

[R87] Yuan Y, Zhu H, Lin W, and Marron JS. Local polynomial regression for symmetric positive definite matrices. Journal of the Royal Statistical Society Series B: Statistical Methodology, 74(4):697–719, 2012. 10.1111/j.1467-9868.2011.01022.x PMC3448376 23008683

[R88] Zhao Y, Wang B, Mostofsky SH, Caffo BS, and Luo X. Covariate assisted principal regression for covariance matrix outcomes. Biostatistics, 22(3):629–645, 2021. 10.1093/biostatistics/kxz057 PMC8286560 31851318

[R89] Zou T, Lan W, Wang H, and Tsai C-L. Covariance Regression Analysis. Journal of the American Statistical Association, 112(517):266–281, 2017.

