# Supplementary material for: Wasserstein F-tests for Fréchet regression on Bures-Wasserstein manifolds
Source: J Mach Learn Res. Author manuscript; Available in PMC 2026 Oct 7. (PMC13641123)
Supplement: Supplementary file 1 [file NIHMS2193849-supplement-1.pdf]

## Appendix A. Background on optimal transport and functional calculus

In this section, we collect relevant background about optimal transport and functional calculus to make the paper more self-contained.

### A.1 Geometry of optimal transport

We begin with the geometry of optimal transport, and then specialize the general concepts to the Bures–Wasserstein manifold. For introductory expositions of optimal transport, we refer to Villani (2003); Santambrogio (2015); Panaretos and Zemel (2020). For a more comprehensive treatment, we refer to Ambrosio et al. (2005); Villani (2009).

Given $\mu_0, \mu_1 \in \mathcal{P}_{2,\mathrm{ac}}(\mathbb{R}^d)$, the constant-speed geodesic $(\mu_t)_{t\in[0,1]}$ connecting $\mu_0$ to $\mu_1$ is characterized by

$$\mu_t = \left[\mathrm{id} + t\left(T_{\mu_0}^{\mu_1} - \mathrm{id}\right)\right]_{\#} \mu_0, \qquad t \in [0,1]$$

Here, $\#$ denotes the pushforward operation defined by $T_{\#}\mu(E) = \mu(T^{-1}(E))$ for any Borel set $E \subset \mathbb{R}^d$. Then define the tangent vector of the geodesic $(\mu_t)_{t\in[0,1]}$ at $t = 0$ to be the mapping $T_{\mu_0}^{\mu_1} - \mathrm{id}$. The tangent space $T_{\mu_0}\mathcal{P}_{2,\mathrm{ac}}(\mathbb{R}^d)$ to $\mathcal{P}_{2,\mathrm{ac}}(\mathbb{R}^d)$ at $\mu_0$ is defined in Ambrosio et al., 2005, Thm 8.5.1 as

$$T_{\mu_0}\mathcal{P}_{2,\mathrm{ac}}(\mathbb{R}^d) := \overline{\left\{\lambda(T_{\mu_0}^{\nu} - \mathrm{id}) : \lambda > 0, \nu \in \mathcal{P}_{2,\mathrm{ac}}(\mathbb{R}^d)\right\}}^{L^2(\mu_0)}$$

Here the overline denotes closure with respect to the $L^2(\mu_0)$ measure.

Given two covariance matrices $Q, S \in \mathcal{S}_d^{++}$, the constant-speed geodesic connecting the corresponding centered Gaussians is given by

$$\left(I_d + t\left(T_Q^S - I_d\right)\right) Q \left(I_d + t\left(T_Q^S - I_d\right)\right), \quad t \in [0,1]$$

The tangent space $T_Q\mathcal{S}_d^{++}$ can be identified with the space $\mathcal{S}_d$ of symmetric $d \times d$ matrices. For any $\widetilde{S} \in T_Q\mathcal{S}_d^{++}$, its norm in the tangent space is given by

$$\left\|\widetilde{S}\right\|_Q := \left\langle \widetilde{S}, Q\widetilde{S}\right\rangle^{1/2}$$

### A.2 Functional calculus

To consider higher order differentials of $T_Q^S$ on the Bures-Wasserstein manifold, we give a brief review of some key concepts in functional calculus that are essential for the development, and direct readers to Dudley and Norvaiša (2011) for further details.

Let $Y$ and $Z$ be normed spaces with the norm on each denoted by $\|\cdot\|$, and let $U$ be an open subset of $Y$. Let $L(Y, Z)$ denote the space of all bounded linear operators from $Y$ into $Z$. A function $\phi : U \to Z$ is called (Fréchet) differentiable at $u \in U$ if there exists an $L(u) \in L(Y, Z)$ such that for each $y \in Y$ with $u + Y \in Y$,

$$\lim_{y\to 0} \frac{\|\phi(u + y) - \phi(u) - L(u)y\|}{\|y\|} = 0$$

The linear operator $L(u)$ is unique, is called the derivative of $\phi$ at $u$, and is denoted by $D\phi(u)$. If the function $\phi$ is differentiable at each $u \in U$, then we say that $\phi$ is differentiable on $U$.

To consider higher order derivatives of $\phi$, let $L^1(Y,Z) := L(Y,Z)$ with the usual operator norm, and let $L^{k+1}(Y,Z) := L(Y, L^k(Y,Z))$ with the operator norm recursively for $k = 1, 2, \ldots$. For $k \geq 2$, we say that $\phi$ is (Fréchet) differentiable of order $k$ at $u$ if $\phi$ has a $(k-1)$st derivative $D^{k-1}\phi(y)$ at each point $y$ of some neighborhood of $u$, and the mapping $D^{k-1}\phi$ is differentiable at $u$. Then $D^k\phi(u)$, the $k$th derivative of $\phi$ at $u$, is defined as the derivative of $D^{k-1}\phi$ at $u$. If $\phi$ is differentiable of order $k$ at $u$ for each $u \in U$ then we say that $\phi$ is differentiable of order $k$ on $U$. A mapping $\phi$ is called a $C^k$ function on $U \subset Y$ if the derivatives of $\phi$ through order $k$ all exist on $U$ and are continuous. A mapping $\phi$ is called a $C^\infty$ function on $U \subset X$ if it is a $C^k$ function for each $k$.

It would be convenient to introduce the notion of multilinear mappings in order study the properties of higher order derivatives $D^k\phi$. A function $A : Y^k \to Z$ is called $k$-linear if for each $j \in [k]$, $A(y_1, \ldots, y_k)$ is linear in $y_j$ for any fixed values of $y_i$, $i \neq j$. The function $A$ is called bounded if

$$\|A\| := \sup\left\{\|A(y_1, \ldots, y_k)\| : \|y_j\| \leq 1, j \in [k]\right\} < \infty \tag{29}$$

Let $\mathcal{M}_k(Y,Z)$ be the set of all bounded $k$-linear maps from $Y^k$ to $Z$ with norm define by (29).

The space $L^k(Y,Z)$ can be identified with $\mathcal{M}_k(Y,Z)$ through the natural isomorphism $\Phi_{(k)} : L^k(Y,Z) \to \mathcal{M}_k(Y,Z)$ defined by

$$\begin{aligned}\Phi_{(k)}(A)(y_1, \ldots, y_k) &:= A(y_1)(y_2)\cdots(y_k) \\ &:= \left[\cdots\left[A(y_1)\right](y_2)\cdots\right](y_k)\end{aligned}$$

Then the $k$th differential at $u$ is defined by $d^k\phi(u) := \Phi_{(k)}(D^k\phi(u))$.

The $k$th differential $d^k\phi(u)$ is symmetric in the sense that

$$d^k\phi(u)(y_{\sigma(1)}, \ldots, y_{\sigma(k)}) = d^k\phi(u)(y_1, \ldots, y_k)$$

for any permutation $\sigma$ of $[k]$. For simplicity, we also denote $d^k\phi(u)(y, \ldots, y)$ by $d^k\phi(u) \cdot y^{\otimes k}$.

Let $\mathcal{M}_{k,s}(Y,Z)$ denote the subspace of all symmetric elements of $\mathcal{M}_k(Y,Z)$. Then aside from the operator norm inherited from $\mathcal{M}_k(Y,Z)$, one can define another norm $|||\cdot|||$ on $\mathcal{M}_{k,s}(Y,Z)$ by

$$|||P||| := \sup\left\{\|P(y, y, \ldots, y)\| : \|y\| \leq 1\right\}$$

Properties of the high order differentials $d^kT_Q^S$ are investigated in Appendix B.1.

## Appendix B. Technical lemmas

In this section, we collect technical lemmas and relevant notations for the proof. First, properties of differentials of $T_Q^S$ are collected in Appendix B.1. Then various concentration results are given in Appendix B.2. Finally, properties of $F(\cdot,\cdot)$ and $Q^*(\cdot)$ are summarized in Appendix B.3.

### B.1 Differentials of optimal transport maps

Recall that the optimal transport map between two centered Gaussian distributions $\mathcal{N}(0,Q)$ and $\mathcal{N}(0,S)$ has the closed-form expression $T_Q^S = S^{1/2}(S^{1/2}QS^{1/2})^{-1/2}S^{1/2}$. In all relevant

analysis, we need to consider differentials of $T_Q^S$ when viewed as a function of $Q$ for fixed $S$, which we denote as $d^k T_Q^S$ for $k \geq 1$. It is shown in Kroshnin et al. (2021) that $dT_Q^S$ can be defined as follows. For any $H \in \mathcal{S}_d$,

$$dT_Q^S(H) := -S^{1/2}U^\top \Lambda^{-1/2} \delta \Lambda^{-1/2} U S^{1/2}$$

where $U^\top \Lambda U$ is an eigenvalue decomposition of $S^{1/2}QS^{1/2}$ with $UU^\top = U^\top U = I$ and $\delta = (\delta_{ij})_{i,j=1}^d$ with

$$\delta_{ij} = \begin{cases} \frac{\Delta_{ij}}{\sqrt{\lambda_i}+\sqrt{\lambda_j}} & i,j \leq \operatorname{rank}(S) \\ 0 & \text{otherwise} \end{cases}, \qquad \Delta = US^{1/2}HS^{1/2}U^\top$$

To consider higher order differentials of $T_Q^S$, we start with the map $Q \mapsto Q^{1/2}$ in Lemma 19, next move on to $Q \mapsto Q^{-1/2}$ in Lemma 20 which then leads to $T_Q^S$ in Lemma 21. Connections between $T_Q^S$ and $W^2(Q,S)$ are also investigated in Lemma 21.

**Lemma 19** *The square root functional* $\phi : Q \in \mathcal{S}_d^{++} \to \phi(Q) = Q^{1/2} \in \mathcal{S}_d^{++}$ *is Fréchet differentiable at any order on* $\mathcal{S}_d^{++}$*. Moreover, for any* $Q \in \mathcal{S}_d^{++}$ *and any* $n \geq 0$*, we have the estimates*

$$\left\|\left| d^{n+1}\phi(Q) \right|\right\| \leq C_{d,n} \lambda_{\min}(Q)^{-(n+1/2)}$$

*Here* $C_{d,n} = d^{n/2} \cdot n! \binom{2n}{n} \cdot 2^{-(2n+1)}$.

**Proof** See Del Moral and Niclas (2018) Theorem 1.1. ■

**Lemma 20** *The inverse square root functional* $\varphi : Q \in \mathcal{S}_d^{++} \mapsto \varphi(Q) = Q^{-1/2} \in \mathcal{S}_d^{++}$ *is Fréchet differentiable at any order on* $\mathcal{S}_d^{++}$*. Moreover, for any* $A \in \mathcal{S}_d^{++}, H \in \mathbb{S}^d$*, the following holds.*

$$\begin{aligned}
d\varphi(Q) \cdot H &= -Q^{-1/2} \left(d\phi(Q) \cdot H\right) Q^{-1/2} \\
d^2\varphi(Q) \cdot H^{\otimes 2} &= -Q^{-1/2} \left(d^2\phi(Q) \cdot H^{\otimes 2}\right) Q^{-1/2} + 2Q^{-1/2}(d\phi(Q) \cdot H)Q^{-1/2}(d\phi(Q) \cdot H)Q^{-1/2} \\
d^3\varphi(Q) \cdot H^{\otimes 3} &= -Q^{-1/2} \left(d^3\phi(Q) \cdot H^{\otimes 3}\right) Q^{-1/2} + 3Q^{-1/2} \left(d^2\phi(Q) \cdot H^{\otimes 2}\right) Q^{-1/2} \left(d\phi(Q) \cdot H\right) Q^{-1/2} \\
&\quad + 3Q^{-1/2} \left(d\phi(Q) \cdot H\right) Q^{-1/2} \left(d^2\phi(Q) \cdot H^{\otimes 2}\right) Q^{-1/2} \\
&\quad - 6Q^{-1/2} \left(d\phi(Q) \cdot H\right) Q^{-1/2} \left(d\phi(Q) \cdot H\right) Q^{-1/2} \left(d\phi(Q) \cdot H\right) Q^{-1/2}
\end{aligned}$$

*with*

$$\begin{aligned}
\left\|\left| d\varphi(Q) \right|\right\| &\leq C_{d,0} \lambda_{\min}(Q)^{-3/2} \\
\left\|\left| d^2\varphi(Q) \right|\right\| &\leq (C_{d,1} + 2C_{d,0}^2) \cdot (\lambda_{\min}(Q))^{-5/2} \\
\left\|\left| d^3\varphi(Q) \right|\right\| &\leq \left(C_{d,2} + 6C_{d,1}C_{d,0} + 6C_{d,0}^3\right) \lambda_{\min}(Q)^{-7/2}
\end{aligned}$$

*where* $C_{d,n}$ *is defined in Lemma 19.*

**Proof** By Lemma 19 we have for infinitesimal $H \in \mathcal{S}_d$ that

$$(Q+H)^{1/2} = Q^{1/2} + \underbrace{d\phi(Q)\cdot H}_{Z_1} + \underbrace{\frac{1}{2}d^2\phi(Q)\cdot H^{\otimes 2}}_{Z_2} + \underbrace{\frac{1}{6}d^3\phi(Q)\cdot H^{\otimes 3}}_{Z_3} + \cdots$$

Let $Z = Z_1 + Z_2 + Z_3 + Z_4$, and we obtain for infinitesimal $H \in \mathcal{S}_d$ that

$$\begin{aligned}
&(Q+H)^{-1/2}\\
&= \left(Q^{1/4}(I_d + Q^{-1/4}ZQ^{-1/4})Q^{1/4}\right)^{-1}\\
&= Q^{-1/4}(I_d + Q^{-1/4}ZQ^{-1/4})^{-1}Q^{-1/4}\\
&\overset{(i)}{=} Q^{-1/4}\left(I_d - Q^{-1/4}ZQ^{-1/4} + Q^{-1/4}ZQ^{-1/2}ZQ^{-1/4} - Q^{-1/4}ZQ^{-1/2}ZQ^{-1/2}ZQ^{-1/4} + \cdots\right)Q^{-1/4}\\
&= Q^{-1/2} - Q^{-1/2}ZQ^{-1/2} + Q^{-1/2}ZQ^{-1/2}ZQ^{-1/2} - Q^{-1/2}ZQ^{-1/2}ZQ^{-1/2}ZQ^{-1/2} + \cdots\\
&\overset{(ii)}{=} Q^{-1/2} - \underbrace{Q^{-1/2}Z_1Q^{-1/2}}_{\text{1st order in } H} - \underbrace{Q^{-1/2}Z_2Q^{-1/2} + Q^{-1/2}Z_1Q^{-1/2}Z_1Q^{-1/2}}_{\text{2nd order in } H}\\
&\underbrace{-Q^{-1/2}Z_3Q^{-1/2} + Q^{-1/2}Z_2Q^{-1/2}Z_1Q^{-1/2} + Q^{-1/2}Z_1Q^{-1/2}Z_2Q^{-1/2} - Q^{-1/2}Z_1Q^{-1/2}Z_1Q^{-1/2}Z_1Q^{-1/2}}_{\text{3rd order in } H}\\
&+ \text{higher order terms in } H
\end{aligned}$$

Here (i) follows from the von Neumann series expansion, and (ii) is obtained by arranging terms according to their orders in $H$. Then (20) follows, and we have the following estimate

$$\begin{aligned}
\left\|d^2\varphi(Q)\cdot H^{\otimes 2}\right\|_{\mathrm{F}} &\leq \left\|Q^{-1/2}\left(d^2\phi(Q)\cdot H^{\otimes 2}\right)Q^{-1/2}\right\|_{\mathrm{F}} + 2\left\|Q^{-1/2}(d\phi(Q)\cdot H)Q^{-1/2}(d\phi(Q)\cdot H)Q^{-1/2}\right\|_{\mathrm{F}}\\
&\leq \lambda_{\min}(Q)^{-1}\cdot \left|\!\left|\!\left|d^2\phi(Q)\right|\!\right|\!\right|\cdot\|H\|_{\mathrm{F}}^2 + 2\lambda_{\min}(Q)^{-3/2}\left|\!\left|\!\left|d\phi(Q)\right|\!\right|\!\right|^2\cdot\|H\|_{\mathrm{F}}^2\\
&\leq (C_{d,1} + 2C_{d,0}^2)\lambda_{\min}(Q)^{-5/2}\cdot\|H\|_{\mathrm{F}}^2
\end{aligned}$$

and similarly

$$\begin{aligned}
\left\|d^3\varphi(Q)\cdot H^{\otimes 3}\right\|_{F} &\leq \lambda_{\min}(Q)^{-1}\left|\!\left|\!\left|d^3\varphi(Q)\right|\!\right|\!\right|\cdot\|H\|_{\mathrm{F}}^3 + 6\lambda_{\min}(Q)^{-3/2}\left|\!\left|\!\left|d^2\varphi(Q)\right|\!\right|\!\right|\cdot\left|\!\left|\!\left|d\varphi(Q)\right|\!\right|\!\right|\cdot\|H\|_{\mathrm{F}}^3\\
&\quad + 6\lambda_{\min}(Q)^{-2}\left|\!\left|\!\left|d\varphi(Q)\right|\!\right|\!\right|^3\cdot\|H\|_{\mathrm{F}}^3\\
&\leq \left(C_{d,2} + 6C_{d,1}C_{d,0} + 6C_{d,0}^3\right)\lambda_{\min}(Q)^{-7/2}\cdot\|H\|_{\mathrm{F}}^3
\end{aligned}$$

Here the last inequality follows by applying Lemma 19. And we arrive at the desired result. ■

**Lemma 21** *The following properties hold for the 2-Wasserstein distance $W(Q,S)$ and the optimal transport map $T_Q^S$. For any $Q, Q_1, Q_2 \in \mathcal{S}_d^{++}$, $S \in \mathcal{S}_d^{+}$ and $X, Y \in \mathcal{S}_d$,*

***(i)*** *$W^2(Q,S)$ is upper bounded by*

$$W^2(Q,S) \leq 2d\left(\lambda_{\max}(Q) + \lambda_{\max}(S)\right)$$

***(ii)*** *$W^2(Q,S)$ is twice differentiable with*

$$d_Q W^2(Q,S)(X) = \left\langle I - T_Q^S, X \right\rangle$$
$$d_Q^2 W^2(Q,S)(X,Y) = -\left\langle X, dT_Q^S(Y) \right\rangle$$

*Moreover, the following quadratic approximation holds:*

$$\begin{aligned}&\frac{2}{\left(1+\lambda_{\max}^{1/2}(Q')\right)^2}\left\langle -dT_{Q_0}^S(Q_1-Q_0), Q_1-Q_0\right\rangle \\ \leq & W^2(Q_1,S) - W^2(Q_0,S) + \left\langle T_{Q_0}^S - I, Q_1 - Q_0\right\rangle \\ \leq & \frac{2}{\left(1+\lambda_{\min}^{1/2}(Q')\right)^2}\left\langle -dT_{Q_0}^S(Q_1-Q_0), Q_1-Q_0\right\rangle\end{aligned}$$

*with $Q' := Q_0^{-1/2} Q_1 Q_0^{-1/2}$.*

***(iii)*** *$dT_Q^S$ is self-adjoint, negative semi-definite and enjoys the following two-sided bound.*

$$\frac{\lambda_{\min}^{1/2}\left(S^{1/2}QS^{1/2}\right)}{2}\left\|Q^{-1/2}XQ^{-1/2}\right\|_F^2 \leq \left\langle -dT_Q^S(X), X\right\rangle \leq \frac{\lambda_{\max}^{1/2}\left(S^{1/2}QS^{1/2}\right)}{2}\left\|Q^{-1/2}XQ^{-1/2}\right\|_F^2$$

***(iv)*** *$W^2(Q_0,Q_1)$ can be upper and lower bounded by the Frobenius norm as follows*

$$\frac{1}{2}\frac{\lambda_{\max}(Q_0)\,\lambda_{\min}^{-2}(Q_0)}{1+\lambda_{\min}^{-1}(Q_0)\,\lambda_{\max}(Q_1)}\cdot\|Q_1-Q_0\|_{\mathrm{F}}^2 \leq W^2(Q_0,Q_1) \leq \frac{\lambda_{\max}(Q_0)\,\lambda_{\min}^{-2}(Q_0)}{1+\lambda_{\max}^{-1}(Q_0)\,\lambda_{\min}(Q_1)}\cdot\|Q_1-Q_0\|_{\mathrm{F}}^2$$

***(v)*** *$\left\|\left|d^k T_Q^S\right|\right\|$ can be upper bounded by*

$$\begin{aligned}\left\|\left|dT_Q^S\right|\right\| &\leq \frac{\lambda_{\min}(Q)^{-2}}{2}\cdot\left(\lambda_{\max}\left(S^{1/2}QS^{1/2}\right)\right)^{1/2} \\ \left\|\left|d^2T_Q^S\right|\right\| &\leq \lambda_{\max}(S)^3\left(C_{d,1}+2C_{d,0}^2\right)\cdot\left(\lambda_{\min}\left(S^{1/2}QS^{1/2}\right)\right)^{-5/2} \\ \left\|\left|d^3T_Q^S\right|\right\| &\leq \lambda_{\max}(S)^4\left(C_{d,2}+6C_{d,1}C_{d,0}+6C_{d,0}^3\right)\left(\lambda_{\min}\left(S^{1/2}QS^{1/2}\right)\right)^{-7/2}\end{aligned}$$

*Moreover, if $S,Q \in \mathcal{S}_d(M^{-1},M)$, then we have*

$$\begin{aligned}\left\|\left|dT_Q^S\right|\right\| &\leq \frac{1}{2}M^3 \\ \left\|\left|d^2T_Q^S\right|\right\| &\leq (C_{d,1}+2C_{d,0}^2)M^8 \\ \left\|\left|d^3T_Q^S\right|\right\| &\leq \left(C_{d,2}+6C_{d,1}C_{d,0}+6C_{d,0}^3\right)M^{11}\end{aligned}$$

**Proof**

**Proof of (i):** By the closed form expression for $W^2(Q,S)$, one has

$$\begin{aligned} W^2(Q,S) &= \operatorname{tr}\left[Q+S-2(S^{1/2}QS^{1/2})^{1/2}\right] \\ &\le \operatorname{tr}\left[Q+S+2(S^{1/2}QS^{1/2})^{1/2}\right] \\ &\le d\left(\lambda_{\max}(Q)+\lambda_{\max}(S)+2\lambda_{\max}(Q)^{1/2}\lambda_{\max}(S)^{1/2}\right) \\ &\le 2d\left(\lambda_{\max}(Q)+\lambda_{\max}(S)\right) \end{aligned}$$

**Proof of (ii), (iii):** see Kroshnin et al. (2021, Lemma A.2, A.3, A.4, A.6).

**Proof of (iv):** Set $S=Q_0$ in ((**ii**)), one can obtain

$$\begin{aligned} \frac{2}{\left(1+\lambda_{\max}^{1/2}(Q')\right)^2}\left\langle -dT_{Q_0}^{Q_0}(Q_1-Q_0),Q_1-Q_0\right\rangle &\le W^2(Q_1,Q_0) \\ &\le \frac{2}{\left(1+\lambda_{\min}^{1/2}(Q')\right)^2}\left\langle -dT_{Q_0}^{Q_0}(Q_1-Q_0),Q_1-Q_0\right\rangle \end{aligned} \tag{30}$$

where $Q'=Q_0^{-1/2}Q_1Q_0^{-1/2}$. Next, apply **(iii)** to get that for any $X\in\mathcal{S}_d$, one has

$$\frac{\lambda_{\min}(Q_0)}{2}\lambda_{\max}^{-2}(Q_0)\|X\|_{\mathrm{F}}^2 \le \left\langle -dT_{Q_0}^{Q_0}(X),X\right\rangle \le \frac{\lambda_{\max}(Q_0)}{2}\lambda_{\min}^{-2}(Q_0)\|X\|_{\mathrm{F}}^2 \tag{31}$$

Combine (30) and (31) to get that

$$\begin{aligned} W^2(Q_1,Q_0) &\le \frac{\lambda_{\max}(Q_0)\lambda_{\min}^{-2}(Q_0)}{\left(1+\lambda_{\min}^{1/2}(Q')\right)^2}\cdot\|Q_1-Q_0\|_{\mathrm{F}}^2 \\ &\le \frac{\lambda_{\max}(Q_0)\lambda_{\min}^{-2}(Q_0)}{1+\lambda_{\min}(Q')}\cdot\|Q_1-Q_0\|_{\mathrm{F}}^2 \\ &\le \frac{\lambda_{\max}(Q_0)\lambda_{\min}^{-2}(Q_0)}{1+\lambda_{\max}^{-1}(Q_0)\lambda_{\min}(Q_1)}\cdot\|Q_1-Q_0\|_{\mathrm{F}}^2 \end{aligned}$$

and similarly

$$\begin{aligned} W^2(Q_1,Q_0) &\ge \frac{\lambda_{\min}(Q_0)\lambda_{\max}^{-2}(Q_0)}{\left(1+\lambda_{\max}^{1/2}(Q')\right)^2}\cdot\|Q_1-Q_0\|_{\mathrm{F}}^2 \\ &\ge \frac{1}{2}\frac{\lambda_{\max}(Q_0)\lambda_{\min}^{-2}(Q_0)}{1+\lambda_{\max}(Q')}\cdot\|Q_1-Q_0\|_{\mathrm{F}}^2 \\ &\ge \frac{1}{2}\frac{\lambda_{\max}(Q_0)\lambda_{\min}^{-2}(Q_0)}{1+\lambda_{\min}^{-1}(Q_0)\lambda_{\max}(Q_1)}\cdot\|Q_1-Q_0\|_{\mathrm{F}}^2 \end{aligned}$$

**Proof of (v):** First, note that results for $dT_Q^S$ follows directly from **(iii)**.

Next since $T_Q^S = S^{1/2}(S^{1/2}QS^{1/2})^{-1/2}S^{1/2}$, applying Lemma 20 gives that for $H$ small enough, one has

$$\begin{aligned} d^2T_Q^S \cdot H^{\otimes 2} &= S^{1/2}\left[d^2\varphi(S^{1/2}QS^{1/2}) \cdot (S^{1/2}HS^{1/2})^{\otimes 2}\right]S^{1/2} \\ d^3T_Q^S \cdot H^{\otimes 3} &= S^{1/2}\left[d^3\varphi(S^{1/2}QS^{1/2}) \cdot (S^{1/2}HS^{1/2})^{\otimes 3}\right]S^{1/2} \end{aligned}$$

which implies

$$\begin{aligned} \left|\!\left|\!\left| d^2T_Q^S \right|\!\right|\!\right| &\leq \lambda_{\max}(S)^3 \cdot \left|\!\left|\!\left| d^2\varphi(S^{1/2}QS^{1/2}) \right|\!\right|\!\right| \\ &\leq \lambda_{\max}(S)^3 (C_{d,1} + 2C_{d,0}^2) \cdot \left(\lambda_{\min}(S^{1/2}QS^{1/2})\right)^{-5/2} \\ \left|\!\left|\!\left| d^3T_Q^S \right|\!\right|\!\right| &\leq \lambda_{\max}(S)^4 \left|\!\left|\!\left| d^3\varphi(S^{1/2}QS^{1/2}) \right|\!\right|\!\right| \\ &\leq \lambda_{\max}(S)^4 \left(C_{d,2} + 6C_{d,1}C_{d,0} + 6C_{d,0}^3\right) \lambda_{\min}(S^{1/2}QS^{1/2})^{-7/2} \end{aligned}$$

■

**Lemma 22** *Let $Y$ and $Z$ be normed spaces with the norm on each denoted by $\|\cdot\|$. For any $y \in Y$, $P \in \mathcal{M}_{k,s}(Y,Z)$, $k \geq 2$, let $Py$ denote a $(k-1)$-linear function defined by*

$$Py(y_1, \ldots, y_{k-1}) = P(y, y_1, \ldots, y_{k-1})$$

*Then $Py \in \mathcal{M}_{k-1,s}(Y,Z)$ and*

$$|\!|\!| Py |\!|\!| \leq \|P\|\,\|y\| \leq \frac{k^k}{k!} |\!|\!| P |\!|\!|\,\|y\| \tag{32}$$

**Proof** $Py \in \mathcal{M}_{k-1,s}(Y,Z)$ can be proved by definition. To prove (32), note that for any $\|\widetilde{y}\| \leq 1$, one has

$$\begin{aligned} \|Py(\widetilde{y}, \ldots, \widetilde{y})\| &\leq \|P\| \cdot \|y\| \cdot \|\widetilde{y}\|^{k-1} \\ &\leq \|P\| \cdot \|y\| \\ &\overset{(i)}{\leq} \frac{k^k}{k!} |\!|\!| P |\!|\!| \cdot \|y\| \end{aligned}$$

Here (i) follows from Dudley and Norvaiša (2011, Theorem 5.7).

■

### B.2 Concentration inequalities and uniform convergence

First, let us introduce some additional notation. Given a random variable $X$ we denote $\|X\|_{\psi_\alpha}$ for $\alpha > 0$ as follows.

$$\|X\|_{\psi_\alpha} := \inf\left\{\eta > 0 : \mathbb{E}\psi_\alpha\left(|X/\eta|\right) \le 1\right\}, \quad \text{where } \psi_\alpha(x) := \exp\left(x^\alpha\right) - 1 \quad \text{for } x \ge 0 \tag{33}$$

Note that by the definition and Markov's inequality, if $\|X\|_{\psi_\alpha} < \infty$, then

$$\mathbb{P}\left(|X| \ge t\right) \le 2\exp\left(-\frac{t^\alpha}{\|X\|_{\psi_\alpha}^\alpha}\right) \tag{34}$$

For $\alpha \ge 1$, $\|\cdot\|_{\psi_\alpha}$ is a norm (Vershynin, 2018), and for $\alpha < 1$, it is equivalent to a norm (Talagrand, 1989).

**Lemma 23** *Let $X$ be a random variable and $\alpha \ge 1$. Then the following properties are equivalent; the parameters $K_i > 0$ appearing in these properties differ from each other by at most an absolute constant factor depending on $\alpha$.*

1. *The $\psi_\alpha$-norm of $X$ satisfies $\|X\|_{\psi_\alpha} \le K_1$.*
2. *The tails of $X$ satisfy*

$$\mathbb{P}\left\{|X| \ge t\right\} \le 2\exp\left(-t^\alpha/K_2^\alpha\right) \quad \forall t \ge 0.$$

3. *The moments of $X$ satisfy*

$$\|X\|_p \le K_3 p^{1/\alpha}$$

**Proof** See Vershynin (2018, Exercise 2.7.3). ■

For a random vector $X \in \mathbb{R}^p$, denote

$$\|X\|_{\psi_\alpha} := \sup_{u \in \mathbb{R}^p, \|u\|=1} \left\|u^\top X\right\|_{\psi_\alpha}$$

**Lemma 24** *Let $C_1, c_1, \alpha, \beta, B > 0$. Then*

$$\min\left\{1, B^\beta \cdot C_1 \exp\left(-c_1 t^\alpha\right)\right\} \le C_1 \exp\left(-c_1 \frac{t^\alpha}{1 + \frac{\beta\log(1\vee B)}{\log(1\vee C_1)}}\right)$$

**Proof** Without loss of generality, assume $C_1, B \ge 1$. Denote

$$g(t) := C_1 \exp\left(-c_1 t^\alpha\right)$$
$$f(t) := C_1 \exp\left(-c_1 \frac{t^\alpha}{1 + \frac{\beta\log B}{\log C_1}}\right)$$

$f, g : (0, +\infty) \to \mathbb{R}$ satisfy the following properties

- $g, f$ are both positive and decreasing for $t \geq 0$.
- denote $t_0 = \left[\frac{\beta \log B + \log C_1}{c_1}\right]^{1/\alpha}$, then

$$1 \wedge g(t_0) = 1 \leq f(t_0)$$

- derivatives satisfy

$$\frac{d}{dt}\left[\log(g(t))\right] \leq \frac{d}{dt}\left[\log(f(t))\right], \quad \forall t \geq t_0$$

Therefore,

$$1 \wedge g(t) \leq f(t), \quad \forall t > 0$$

■

**Lemma 25** *Let $\alpha \geq 1$. Let $X, Q$ be random elements in $\mathbb{R}^d, \mathbb{R}^{d\times d}$ and let $\Phi_k$ be a random symmetric operator in $\mathcal{L}\left((\mathbb{R}^{d\times d})^{\times k}; \mathbb{R}^{d\times d}\right)$ for some $k \in \mathbb{N}_+$. Then*

*I. $\|X\|_{\psi_\alpha} \leq \|\|X\|_2\|_{\psi_\alpha} \leq c_0(2d+1)^{1/\alpha}\|X\|_{\psi_\alpha}$ for any $\alpha \geq 1$ where $c_0 > 0$ is some absolute constant independent of $d$ and $\alpha$.*

*II. If $Q \in \mathbb{R}^{d\times d}$ is symmetric, then for any $\|v\| = 1$,*

$$\left\|v^\top Q v\right\|_{\psi_\alpha} \leq \left\|\|Q\|_{\mathrm{op}}\right\|_{\psi_\alpha} \leq c_1\left(2d \cdot \frac{\log 3}{\log 2} + 1\right)^{1/\alpha} \sup_{\|v\|=1}\left\|v^\top Q v\right\|_{\psi_\alpha}$$

*where $c_1 > 0$ is some absolute constant independent of $d$ and $\alpha$.*

*III. For any $U \in \mathbb{R}^{d\times d}$ with unit Frobenius norm*

$$\|\langle U, Q\rangle\|_{\psi_\alpha} \leq \|\|Q\|_{\mathrm{F}}\|_{\psi_\alpha} \lesssim \sup_{U:\|U\|_{\mathrm{F}}=1}\|\langle U, Q\rangle\|_{\psi_\alpha}$$

*IV. For any $U \in \mathbb{R}^{d\times d}$ with unit Frobenius norm*

$$\left\|\left\|\Phi_k \cdot U^{\otimes k}\right\|_{\mathrm{F}}\right\|_{\psi_\alpha} \leq \|\|\!|\Phi_k|\!\|\|_{\psi_\alpha} \lesssim \sup_{U:\|U\|_{\mathrm{F}}=1}\left\|\left\|\Phi_k \cdot U^{\otimes k}\right\|_{\mathrm{F}}\right\|_{\psi_\alpha}$$

*The constants behind $\lesssim$ only depend on dimension $d$, $k$ and possible on $\alpha$.*

**Proof**
**I:** The first inequality $\|X\|_{\psi_\alpha} \leq \|\|X\|_2\|_{\psi_\alpha}$ follows since $\langle v, X\rangle \leq \|X\|$ for any $\|v\| = 1$.

For the second inequality, by Lemma 23, we can assume without loss of generality that $\|X\|_{\psi_\alpha}$ satisfies $K_2 = 1$, i.e.

$$\sup_{\|v\|=1} \mathbb{P}\left\{|\langle v, X\rangle| \geq t\right\} \leq 2\exp\left(-t^\alpha\right)$$

Following the 1/2-net argument in Lemma 1 in Jin et al. (2019), one can obtain

$$\begin{aligned}\mathbb{P}\left\{\|X\| \geq t\right\} &\leq 4^d \sup_{\|v\|=1} \mathbb{P}(\langle v, X\rangle \geq t/2) \\ &\leq \underbrace{2 \cdot 4^d \exp\left(-t^\alpha/2^\alpha\right)}_{=:g(t)}\end{aligned}$$

Lemma 24 implies that for any $t \geq 0$,

$$\mathbb{P}\left\{\|X\| \geq t\right\} \leq 2\exp\left(-\frac{t^\alpha}{(2d+1)\cdot 2^\alpha}\right) =: f(t)$$

Therefore, Lemma 23 implies that

$$\left\|\|X\|_2\right\|_{\psi_\alpha} \leq c_0(2d+1)^{1/\alpha}\left\|X\right\|_{\psi_\alpha}$$

where $c_0 > 0$ is some absolute constant independent of $d$ and $\alpha$.

**II:** We prove the second inequality here. By Lemma 23, we can assume without loss of generality that $\sup_{\|v\|=1}\left\|v^\top Qv\right\|_{\psi_\alpha}$ satisfies $K_2 = 1$, i.e.

$$\sup_{\|v\|=1} \mathbb{P}\left\{\left|v^\top Qv\right| \geq t\right\} \leq 2\exp\left(-t^\alpha\right)$$

Following the 1/4-net argument in Vershynin (2018, Exercise 4.4.3, Theorem 4.4.5.), one can obtain that for any $t \geq 0$,

$$\mathbb{R}\left\{\left\|\|Q\|_{\text{op}}\right\|_{\psi_\alpha} \geq t\right\} \leq 9^d \cdot 2\exp(-t^\alpha/2^\alpha)$$

Lemma 24 then implies that

$$\mathbb{R}\left\{\left\|\|Q\|_{\text{op}}\right\|_{\psi_\alpha} \geq t\right\} \leq 2\exp\left(-\frac{t^\alpha}{(2d\cdot\frac{\log 3}{\log 2}+1)\cdot 2^\alpha}\right), \quad \forall t \geq 0$$

Hence Lemma 23 implies that

$$\left\|\|Q\|_{\text{op}}\right\|_{\psi_\alpha} \leq c_1\left(2d\cdot\frac{\log 3}{\log 2}+1\right)^{1/\alpha}\sup_{\|v\|=1}\left\|v^\top Qv\right\|_{\psi_\alpha}$$

where $c_1 > 0$ is some absolute constant independent of $d$ and $\alpha$.

The proofs for statements **III** and **IV** follow a similar approach. ■

Next, we give some properties of the weights $w(x, X)$ and $w_{n,\rho}(x, X_i)$ that will be crucial to prove uniform concentration as well as a central limit theorem for the estimate $\widehat{Q}_\rho(x)$. Recall that by definition, $w(x, X) = 1 + (x-\mu)^\top\Sigma^{-1}(X-\mu)$ and $w_{n,\rho}(x) = 1 + (x - \overline{X})\widehat{\Sigma}_\rho^{-1}(X_i - \overline{X})$. For any vector $z \in \mathbb{R}^p$, denote $\vec{z} = (1, z^\top)^\top$ and

$$\vec{\Sigma} := \mathbb{E}\vec{X}\vec{X}^\top = \begin{pmatrix} 1 & \mu^\top \\ \mu & \Sigma + \mu\mu^\top\end{pmatrix}$$

$$\vec{\Sigma}_\rho := \begin{pmatrix} 1 & \mu^\top \\ \mu & \Sigma_\rho + \mu\mu^\top \end{pmatrix}, \quad \text{where } \Sigma_\rho = \Sigma + \rho I_p$$
$$\widehat{\vec{\Sigma}} := n^{-1}\sum_{i=1}^n \vec{X}_i\vec{X}_i^\top = \begin{pmatrix} 1 & \widehat{\mu}^\top \\ \widehat{\mu} & \widehat{\Sigma} + \widehat{\mu}\widehat{\mu}^\top \end{pmatrix}$$
$$\widehat{\vec{\Sigma}}_\rho := \begin{pmatrix} 1 & \widehat{\mu}^\top \\ \widehat{\mu} & \widehat{\Sigma}_\rho + \widehat{\mu}\widehat{\mu}^\top \end{pmatrix}$$

**Lemma 26** *Suppose $X, X_1, \ldots, X_n \overset{\text{i.i.d.}}{\sim} \mathbb{P} \in \mathcal{P}_2(\mathbb{R}^p)$. Let $\widehat{\vec{\Sigma}} = n^{-1}\sum_{i=1}^n \vec{X}_i\vec{X}_i^\top$. Then for any $x \in \mathbb{R}^p$,*

$$w(x, X) = \vec{x}^\top\vec{\Sigma}^{-1}\vec{X} \tag{35}$$
$$w_{n,\rho}(x, X_i) = \vec{x}^\top\widehat{\vec{\Sigma}}_\rho^{-1}\vec{X} \tag{36}$$

**Proof** For (35), by definition one has

$$\vec{\Sigma} = \begin{pmatrix} 1 & \mu^\top \\ \mu & \Sigma + \mu\mu^\top \end{pmatrix}$$

Computing the inverse of the block matrix $\vec{\Sigma}$ then gives

$$\vec{\Sigma}^{-1} = \begin{pmatrix} 1 + \mu^\top\Sigma^-\mu & -\mu^\top\Sigma^- \\ -\Sigma^-\mu & \Sigma^- \end{pmatrix}$$

Finally we arrive at (35) by computing $\vec{x}^\top\Lambda^{-1}\vec{X}$. (36) follows from similar arguments. ■

**Lemma 27** *Suppose Assumption 1 holds. Set*

$$W_{0n} := W_{0n}(x) = -\overline{X}^\top\widehat{\Sigma}_\rho^{-1}(x - \overline{X}) + \mu^\top\widehat{\Sigma}_\rho^{-1}(x - \mu)$$
$$W_{1n} := W_{1n}(x) = \Sigma^{-1}(x - \mu) - \widehat{\Sigma}_\rho^{-1}(x - \overline{X})$$

*one has*

$$w_{n,\rho}(x, X_i) - w(x, X_i) = W_{0n} + W_{1n}^\top X_i \tag{37}$$

*Moreover, for any $\rho \in \{0, n^{-1}\}$, $L \geq 1$ and $\tau \geq 0$, there exists a constant $C > 0$ independent of $n$ such that*

$$\mathbb{P}\left\{\sup_{x \in B_\mu(L)} |W_{0n}(x)| > \frac{L}{\sqrt{n}}C(1+\tau)\log n\right\} \lesssim n^{-(1+\tau)} \tag{38}$$
$$\mathbb{P}\left\{\sup_{x \in B_\mu(L)} \|W_{1n}(x)\| > \frac{L}{\sqrt{n}}C(1+\tau)\log n\right\} \lesssim n^{-(1+\tau)} \tag{39}$$

**Proof** Under Assumption 1, we can assume without loss of generality that $\mu = 0$ and $\Sigma = I_p$. Then

$$\begin{aligned} w_{n,\rho}(x, X_i) &= 1 + (x - \overline{X})^\top \widehat{\Sigma}_\rho^{-1}(X_i - \overline{X}) \\ w(x, X) &= 1 + x^\top X_i \end{aligned}$$

*Proof of (37):* Direct computation gives

$$\begin{aligned} w_{n,\rho}(x, X_i) - w(x, X_i) &= (x - \overline{X})^\top \widehat{\Sigma}_\rho^{-1}(X_i - \overline{X}) - x^\top X_i \\ &= \underbrace{-x^\top \widehat{\Sigma}_\rho^{-1}\overline{X} + \overline{X}^\top \widehat{\Sigma}_\rho^{-1}\overline{X}}_{=W_{0n}(x)} + \Bigg(\underbrace{(\widehat{\Sigma}_\rho^{-1} - I_p)x + \widehat{\Sigma}_\rho^{-1}\overline{X}}_{=W_{1n}(x)}\Bigg)^\top X_i \end{aligned}$$

*Proof of (38), (39):* For $W_{0n}(x)$:

$$\begin{aligned} \mathbb{P}\left\{\sup_{x \in B_\mu(L)} |W_{0n}(x)| > t\right\} &\le \mathbb{P}\left(\left\{\left\|\widehat{\Sigma}_\rho^{-1}\right\|_{\text{op}} \le 2\right\} \cap \left\{L\left\|\overline{X}\right\| + \left\|\overline{X}\right\|^2 \ge t/2\right\}\right) \\ &\quad + \mathbb{P}\left(\left\{2 < \left\|\widehat{\Sigma}_\rho^{-1}\right\|_{\text{op}} \le n\right\} \cap \left\{L\left\|\overline{X}\right\| + \left\|\overline{X}\right\|^2 \ge t/n\right\}\right) \\ &\quad + \mathbb{P}\left\{\left\|\widehat{\Sigma}_\rho^{-1}\right\|_{\text{op}} > n\right\} \\ &\le \underbrace{\mathbb{P}\left(\left\{L\left\|\overline{X}\right\| + \left\|\overline{X}\right\|^2 \ge t/2\right\}\right)}_{(\mathbf{I})} \\ &\quad + \underbrace{\mathbb{P}\left(\left\{2 < \left\|\widehat{\Sigma}_\rho^{-1}\right\| \le n\right\}\right) \wedge \mathbb{P}\left(\left\{L\left\|\overline{X}\right\| + \left\|\overline{X}\right\|^2 \ge t/n\right\}\right)}_{(\mathbf{II})} \\ &\quad + \underbrace{\mathbb{P}\left\{\left\|\widehat{\Sigma}_\rho^{-1}\right\| > n\right\}}_{(\mathbf{III})} \end{aligned}$$

Note that for any $s \ge 0$, one has

$$\begin{aligned} \mathbb{P}\left\{L\left\|\overline{X}\right\| + \left\|\overline{X}\right\|^2 \ge s\right\} &\le \mathbb{P}\left\{L\left\|\overline{X}\right\| \ge \frac{\sqrt{n}L}{\sqrt{n}L + 1}s\right\} + \mathbb{P}\left\{\left\|\overline{X}\right\|^2 \ge \frac{1}{\sqrt{n}L + 1}s\right\} \\ &= \mathbb{P}\left\{\left\|\overline{X}\right\| \ge \frac{\sqrt{n}}{\sqrt{n}L + 1}s\right\} + \mathbb{P}\left\{\left\|\overline{X}\right\|^2 \ge \frac{1}{\sqrt{n}L + 1}s\right\} \\ &\lesssim \exp(-cns^2/L^2) + \exp(-c\sqrt{n}s/L) \end{aligned}$$

- If $\rho = 0$, then

$$\begin{aligned} (\mathbf{II}) + (\mathbf{III}) &\le \mathbb{P}\left(\left\|\widehat{\Sigma}_\rho^{-1}\right\|_{\text{op}} > 2\right) \\ &\le \mathbb{P}\left(\left\|\widehat{\Sigma}_\rho - I_p\right\|_{\text{op}} > 1/2\right) \end{aligned}$$

$$\lesssim \exp(-cn)$$

Hence

$$\mathbb{P}\left\{\sup_{x\in B_\mu(L)} |W_{0n}(x)| > t\right\} \lesssim \exp(-cnt^2/L^2) + \exp(-c\sqrt{n}t/L) + \exp(-cn)$$
$$\lesssim \begin{cases} \exp(-c\sqrt{n}t/L) + \exp(-cn), & t \geq L/\sqrt{n} \\ \exp(-cnt^2/L^2) + \exp(-cn), & t < L/\sqrt{n} \end{cases}$$

or equivalently for any $s > 0$,

$$\mathbb{P}\left\{\sup_{x\in B_\mu(L)} |W_{0n}(x)| > \frac{L}{\sqrt{n}}s\right\} \lesssim \begin{cases} \exp(-cs^2) & s < 1 \\ \exp(-cs), & 1 \leq s \leq n \\ \exp(-cn), & s > n \end{cases}$$

- If $\rho = 1/n$, then $\mathbf{III} = 0$. As a result, for any $s \geq 1$,

$$\mathbb{P}\left\{\sup_{x\in B_\mu(L)} |W_{0n}(x)| > s\frac{L}{\sqrt{n}}\right\}$$
$$\lesssim \exp(-cs) + \exp(-cn) \wedge \begin{cases} \exp(-cs^2/n^2), & 1 \leq s \leq n \\ \exp(-cs/n), & s > n \end{cases}$$
$$= \exp(-cs) + \begin{cases} \exp(-cn), & 1 \leq s \leq n^2 \\ \exp(-cs/n), & s > n^2 \end{cases}$$
$$\lesssim \begin{cases} \exp(-cs), & 1 \leq s \leq n \\ \exp(-cn), & n < s \leq n^2 \\ \exp(-cs/n), & s > n^2 \end{cases}$$

*For $W_{1n}(x)$:* note that $\widehat{\Sigma}_\rho^{-1} - I_p = -\widehat{\Sigma}_\rho^{-1}\left(\widehat{\Sigma}_\rho - I_p\right)$, then one has

$$\mathbb{P}\left\{\sup_{x\in B_\mu(L)} \|W_{1n}(x)\| > t\right\}$$
$$\leq \mathbb{P}\left\{\left\|\widehat{\Sigma}_\rho^{-1}\right\|_{\mathrm{op}}\left\|\widehat{\Sigma}_\rho - I_p\right\|_{\mathrm{op}} L + \left\|\widehat{\Sigma}_\rho^{-1}\right\|_{\mathrm{op}}\|\overline{X}\| \geq t\right\}$$
$$\leq \mathbb{P}\left\{\left\|\widehat{\Sigma}_\rho^{-1}\right\|_{\mathrm{op}}\left\|\widehat{\Sigma}_\rho - I_p\right\|_{\mathrm{op}} L \geq \frac{L}{L+1}t\right\} + \mathbb{P}\left\{\left\|\widehat{\Sigma}_\rho^{-1}\right\|_{\mathrm{op}}\|\overline{X}\| \geq \frac{1}{L+1}t\right\}$$

Hence for $L \geq 1$, one has

$$\mathbb{P}\left\{\sup_{x\in B_\mu(L)} \|W_{1n}(x)\| > t\right\}$$
$$\leq \mathbb{P}\left\{\left\|\widehat{\Sigma}_\rho^{-1}\right\|_{\mathrm{op}}\left\|\widehat{\Sigma}_\rho - I_p\right\|_{\mathrm{op}} \geq \frac{t}{2L}\right\} + \mathbb{P}\left\{\left\|\widehat{\Sigma}_\rho^{-1}\right\|_{\mathrm{op}}\|\overline{X}\| \geq \frac{t}{2L}\right\}$$

$$
\begin{aligned}
&\le \mathbb{P}\left(\left\{\left\|\widehat{\Sigma}_\rho^{-1}\right\|_{\mathrm{op}} \le 2\right\} \cap \left\{\left\|\widehat{\Sigma}_\rho - I_p\right\|_{\mathrm{op}} \ge t/(4L)\right\}\right) \\
&+ \mathbb{P}\left(\left\{2 < \left\|\widehat{\Sigma}_\rho^{-1}\right\|_{\mathrm{op}} \le n\right\} \cap \left\{\left\|\widehat{\Sigma}_\rho - I_p\right\|_{\mathrm{op}} \ge t/(2nL)\right\}\right) \\
&+ \mathbb{P}\left(\left\{\left\|\widehat{\Sigma}_\rho^{-1}\right\|_{\mathrm{op}} > n\right\}\right) \\
&+ \mathbb{P}\left(\left\{\left\|\widehat{\Sigma}_\rho^{-1}\right\|_{\mathrm{op}} \le 2\right\} \cap \left\{\|\overline{X}\| \ge t/(4L)\right\}\right) \\
&+ \mathbb{P}\left(\left\{2 < \left\|\widehat{\Sigma}_\rho^{-1}\right\|_{\mathrm{op}} \le n\right\} \cap \left\{\|\overline{X}\| \ge t/(2nL)\right\}\right) \\
&+ \mathbb{P}\left(\left\{\left\|\widehat{\Sigma}_\rho^{-1}\right\|_{\mathrm{op}} > n\right\}\right) \\
&\lesssim \underbrace{\mathbb{P}\left\{\left\|\widehat{\Sigma}_\rho - I_p\right\|_{\mathrm{op}} \ge \frac{t}{4L}\right\} + \mathbb{P}\left\{\|\overline{X}\| \ge \frac{t}{4L}\right\}}_{\textbf{(i)}} \\
&+ \underbrace{\mathbb{P}\left\{2 < \left\|\widehat{\Sigma}_\rho^{-1}\right\|_{\mathrm{op}} \le n\right\} \wedge \left[\mathbb{P}\left\{\left\|\widehat{\Sigma}_\rho - I_p\right\|_{\mathrm{op}} \ge t/(2nL)\right\} + \mathbb{P}\left\{\|\overline{X}\| \ge t/(2nL)\right\}\right]}_{\textbf{(ii)}} \\
&+ \underbrace{\mathbb{P}\left(\left\{\left\|\widehat{\Sigma}_\rho^{-1}\right\|_{\mathrm{op}} > n\right\}\right)}_{\textbf{(iii)}}
\end{aligned}
$$

- If $\rho = 0$, then

$$
\textbf{(ii)} + \textbf{(iii)} \le \mathbb{P}\left\{\left\|\widehat{\Sigma}_\rho^{-1}\right\|_{\mathrm{op}} > 2\right\} \lesssim \exp(-cn)
$$

  As a result, for any $L \ge 1$,

$$
\begin{aligned}
\mathbb{P}\left\{\sup_{x \in B_\mu(L)} \|W_{1n}(x)\| > t\right\} &\lesssim \exp(-cnt/L) \vee \exp(-cnt^2/L^2) + \exp(-cnt^2/L^2) + \exp(-cn) \\
&= \begin{cases} \exp(-cnt^2/L^2), & t < L \\ \exp(-cn), & t \ge L \end{cases}
\end{aligned}
$$

  Therefore, for any $s > 0$, $L \ge 1$,

$$
\mathbb{P}\left\{\sup_{x \in B_\mu(L)} \|W_{1n}(x)\| > \frac{L}{\sqrt{n}} s\right\} \lesssim \begin{cases} \exp(-cs^2), & s < \sqrt{n} \\ \exp(-cn), & s > \sqrt{n} \end{cases}
$$

- If $\rho = n^{-1}$, then $\textbf{(iii)} = 0$. As a result, for any $t = sL/\sqrt{n}$ and $L \ge 1$,

$$
\mathbb{P}\left\{\sup_{x \in B_\mu(L)} \|W_{1n}(x)\| > \frac{L}{\sqrt{n}} s\right\}
$$

$$\lesssim \begin{cases} \exp(-cs^2), & 0 < s < \sqrt{n} \\ \exp(-cn), & \sqrt{n} \le s < n^{3/2} \\ \exp(-cs/\sqrt{n}), & s \ge n^{3/2} \end{cases}$$

Combining results for $W_{0n}(x)$ and $W_{1n}(x)$, one can obtain that for any $\rho \in \left\{0, n^{-1}\right\}$, $L \ge 1$, $\tau \ge 0$ and $1 \le s \le \sqrt{n}$,

$$\mathbb{P}\left\{\sup_{x \in B_\mu(L)} |W_{0n}(x)| > \frac{L}{\sqrt{n}} s\right\} \vee \mathbb{P}\left\{\sup_{x \in B_\mu(L)} \|W_{1n}(x)\| > \frac{L}{\sqrt{n}} s\right\} \lesssim \exp(-cs)$$

By taking $s = O\left((1+\tau)\log n\right)$, the proof is then complete. ∎

**Lemma 28** *Let $\{(X_i, Q_i)\}_{i=1}^n$ be i.i.d. samples satisfying Assumption 1-2. Let $(\mathcal{V}, \|\cdot\|)$ be a normed vector space and $\psi : \mathcal{S}_d^{++} \times \Theta \to \mathcal{V}$ is a mapping parametrized by $\theta \in \Theta$. Suppose there exists an event $\mathcal{E}_0$ under which*

- $\|X_i - \mu\| \le L_n$ *for $i = 1, ..., n$.*
- $\|\psi(Q_i, \theta)\| \le K_n$ *for $i = 1, ..., n$ uniformly for $\theta \in \Theta$.*

*Denote*

$$G(L, \Theta; n) := \sup_{x \in B_\mu(L)} \sup_{\theta \in \Theta} \left\| \frac{1}{n} \sum_{i=1}^n \left[w_{n,\rho}(x, X_i) - w(x, X_i)\right] \psi(Q_i; \theta) \right\|$$

*Then for any $\rho \in \left\{0, n^{-1}\right\}$, $L \ge 1$ and any $\tau \ge 0$, there exists constant $C$ indpendent of $n$ such that*

$$\mathbb{P}\left\{G(L, \Theta; n) \ge C K_n (L_n + 1) \frac{L}{\sqrt{n}} (1+\tau)\log n\right\} \le O\left(n^{-(1+\tau)}\right) + \mathbb{P}(\mathcal{E}_0^c)$$

**Proof** Given $s \ge 1$, define event $\mathcal{E}_1(s)$

$$\mathcal{E}_1(s) := \sup_{x \in B_\mu(L)} \sup_{\theta \in \Theta} \left\| \frac{1}{n} \sum_{i=1}^n \left[w_{n,\rho}(x, X_i) - w(x, X_i)\right] \psi(Q_i; \theta) \right\| \ge 2K_n(L_n + 1) \cdot \frac{L}{\sqrt{n}} s$$

Then

$$\mathbb{P}\left(\mathcal{E}_1(s)\right) \le \mathbb{P}\left(\mathcal{E}_1(s) \cap \mathcal{E}_0\right) + \mathbb{P}\left(\mathcal{E}_0^c\right)$$

By (37) in Lemma 27, one can obtain for any $x$ and $\theta \in \Theta$,

$$\begin{aligned} &\left\| \frac{1}{n} \sum_{i=1}^n w_{n,\rho}(x, X_i)\psi(Q_i; \theta) - \frac{1}{n} \sum_{i=1}^n w(x, X_i)\psi(Q_i; \theta) \right\| \\ =& \left\| W_{0n}(x) \frac{1}{n} \sum_{i=1}^n \psi(Q_i; \theta) + \frac{1}{n} \sum_{i=1}^n W_{1n}(x)^\top X_i \psi(Q_i; \theta) \right\| \end{aligned}$$

$$\leq |W_{0n}(x)| \cdot \frac{1}{n}\sum_{i=1}^{n} \|\psi(Q_i;\theta)\| + \|W_{1n}(x)\|_2 \frac{1}{n}\sum_{i=1}^{n} \|X_i\|_2 \|\psi(Q_i;\theta)\|$$

Hence

$$\begin{aligned}
\mathbb{P}(\mathcal{E}_1(s)\cap\mathcal{E}_0) &\leq \mathbb{P}\left\{\sup_{x\in B_\mu(L)} [|W_{0n}(x)|\cdot K_n + \|W_{1n}(x)\|_2 \cdot L_n K_n] \geq 2K_n(L_n+1)\cdot\frac{L}{\sqrt{n}}s\right\} \\
&= \mathbb{P}\left\{\sup_{x\in B_\mu(L)} [|W_{0n}(x)| + \|W_{1n}(x)\|_2] \geq 2\cdot\frac{L}{\sqrt{n}}s\right\} \\
&\leq \mathbb{P}\left\{\sup_{x\in B_\mu(L)} |W_{0n}(x)| \geq \frac{L}{\sqrt{n}}s\right\} + \mathbb{P}\left\{\sup_{x\in B_\mu(L)} \|W_{1n}(x)\|_2 \geq \frac{L}{\sqrt{n}}s\right\}
\end{aligned}$$

Applying (38) and (38) in Lemma 27 gives the desired result. ■

We will need uniform upper bounds for various quantities of the form

$$\sup_{\theta\in\Theta} f(Z_1^n;\theta)$$

where $Z_1^n$ denotes $(Z_1,...,Z_n)$. To this end, we decompose the above quantity as follows.

$$\underbrace{\sup_{\theta\in\Theta} f(Z_1^n;\theta) - \mathbb{E}\sup_{\theta\in\Theta} f(Z_1^n;\theta)}_{\text{perturbation}} + \underbrace{\mathbb{E}\sup_{\theta\in\Theta} f(Z_1^n;\theta)}_{\text{expectation}}$$

The expectation term is bounded in Lemma 29 below with a chaining argument, while the perturbation term is shown to concentrate in Lemma 31 by exploiting its bounded difference property. The proof of Lemma 29 is deferred to Appendix B.2.1.

**Lemma 29** *Given a function class*

$$\mathcal{F}(\Theta) = \{f(\cdot;\theta) : \mathbb{R}^p \to \mathbb{R} : \theta\in\Theta\}$$

*where $f$ is continuous jointly in $(z,\theta)$ and $(\Theta, d)$ is a separable metric space with finite diameter $D := \sup_{\theta_1,\theta_2\in\Theta} d(\theta_1,\theta_2) \gtrsim 1$. Suppose a random vector $Z\in\mathbb{R}^p$ satisfies the following inequality*

$$\|f(Z;\theta_1) - f(Z;\theta_2)\|_{\psi_2} \leq \tau\left(d(\theta_1,\theta_2)\right), \quad \forall\theta_1,\theta_2\in\Theta \tag{40}$$

*for some increasing function $\tau : \mathbb{R}^+\to\mathbb{R}^+$ that satisfies $\tau(0) = 0, \tau(+\infty) = +\infty$. Then*

- *the following inequality holds*

$$\mathbb{E}\sup_{\theta\in\Theta} |f(Z;\theta)| \leq C\int_0^{\tau(D)} \sqrt{\log N(\tau^{-1}(t);\Theta)}\cdot dt + \sup_{\theta\in\Theta}\mathbb{E}\,|f(Z;\theta)| \tag{41}$$

  *where $C > 0$ is a fixed absolute constant and $N(\epsilon;\Theta)$ is the $\epsilon$-covering number of $\Theta$.*

- *Specifically, if* $\tau$ *has the form*
$$\tau(\epsilon) = \frac{K}{\sqrt{n}} \left(\epsilon \vee \epsilon^{\alpha_0}\right)$$
*for some constant* $\alpha_0 > 0$*, then for any* $D \gtrsim 1$*, one has*
$$\int_0^{\tau(D)} \sqrt{\log N(\tau^{-1}(t);\Theta)} \cdot dt \lesssim \frac{KD^{1\vee\alpha_0}}{\sqrt{n}} \sqrt{\log^+ D} \tag{42}$$

- *Specifically, if* $Z_1, \dots, Z_n \in \mathbb{R}^p$ *are i.i.d. and* $f : (\mathbb{R}^p)^k \times \Theta \to \mathbb{R}$ *is equal to*
$$f(Z_1^n;\theta) = \left\| \frac{1}{n}\sum_{i=1}^n \psi(Z_i;\theta) - \mathbb{E}\frac{1}{n}\sum_{i=1}^n \psi(Z_i;\theta) \right\|, \quad Z_1^n := (Z_1, \dots, Z_n)$$
*where* $\psi(z;\theta) \in \mathcal{M}_{k,s}(\mathbb{R}^m;\mathbb{R}^m)$ *is a symmetric k-linear operator for any* $z \in \mathbb{R}^p, \theta \in \Theta$*. Then the following inequality*
$$\left\| f(Z_1^n;\theta) - f(Z_1^n;\widetilde{\theta}) \right\|_{\psi_2} \lesssim \frac{1}{\sqrt{n}} \left\| \left\| \psi(Z_1;\theta) - \psi(Z_1;\widetilde{\theta}) \right\| \right\|_{\psi_2} \tag{43}$$
*holds.*

**Remark 30** *(43) will be applied in three ways as follows.*

- $m = 1, k = 1$*:* $\psi(z;\theta) \in \mathbb{R}$ *and* $\|\psi(z;\theta)\|$ *reduces to the absolute value of* $\psi(z;\theta)$*. Examples include* $\psi(X, Q; x, S) = w(x, X)W^2(Q, S)$ *in Lemma 38*
- $m = d, k = 1$*:* $\psi(z;\theta) \in \mathcal{L}(\mathbb{R}^m;\mathbb{R}^m)$ *can be viewed as a matrix. Moreover,* $\|\psi(z;\theta)\|$ *reduces to the matrix operator norm. Examples include* $\psi(X, Q; x) = w(x, X)(T_{Q^*(x)}^Q - I_d)$ *in Lemma 40.*
- $m = d \times d, k = 1$*: this is a special case of the previous one by identifying* $d \times d$ *matrices as a vector in* $\mathbb{R}^{d^2}$*. Examples include* $\psi(X, Q; x) = -w(x, X)dT_{Q^*(x)}^Q$ *in Lemma 40.*
- $m = d \times d, k = 2$*: Examples include* $\psi(X, Q; x, S) = w(x, X)d^2T_S^Q$ *in Lemma 40.*

**Lemma 31** *Let* $\psi : \mathbb{R}^p \times \Theta \to \mathbb{R}^q$ *be a class of functions indexed by* $\theta \in \Theta$*. Suppose* $\psi$ *is uniformly bounded in the sense that there exists a finite constant* $K$ *such that* $\|\psi(x;\theta)\|_2 \le K$ *for any* $x \in \mathbb{R}^p$ *and* $\theta \in \Theta$*. For any fixed* $y_0 \in \mathcal{W}$*, define* $f : (\mathbb{R}^p)^n \times \Theta \to \mathbb{R}^q$ *as*
$$f(x_1^n) := \sup_{\theta\in\Theta} \left\| \frac{1}{n}\sum_{i=1}^n \psi(x_i;\theta) - y_0 \right\|_2$$
*Then* $f$ *satisfies the bounded difference property with parameter* $2K/n$*, i.e. for any* $x_1^n, \widetilde{x}_1^n \in (\mathbb{R}^p)^n$ *such that* $\sum_{i=1}^n \mathbb{I}(x_i \neq \widetilde{x}_i) \le 1$*,*
$$\|f(x_1^n) - f(\widetilde{x}_1^n)\|_2 \le \frac{2K}{n}, \tag{44}$$
*Moreover, suppose* $X_1, \dots, X_n$ *are i.i.d. random element in* $\mathbb{R}^p$*, then*
$$\|f(X_1^n) - \mathbb{E}f(X_1^n)\|_{\psi_2} \lesssim \frac{2K}{\sqrt{n}} \tag{45}$$

**Proof** Without loss of generality, assume $x_1 \neq \widetilde{x}_1$ and $x_j = \widetilde{x}_j$ for $j = 2, \ldots, n$. Then one has

$$
\begin{aligned}
&\left\| \frac{1}{n} \sum_{i=1}^{n} \psi(x_i;\theta) - y_0 \right\|_2 - \sup_{\widetilde{\theta}\in\Theta} \left\| \frac{1}{n} \sum_{i=1}^{n} \psi(\widetilde{x}_i;\widetilde{\theta}) - y_0 \right\|_2 \\
\leq &\left\| \frac{1}{n} \sum_{i=1}^{n} \psi(x_i;\theta) - y_0 \right\|_2 - \left\| \frac{1}{n} \sum_{i=1}^{n} \psi(\widetilde{x}_i;\theta) - y_0 \right\|_2 \\
\leq &\left\| \frac{1}{n} \sum_{i=1}^{n} \psi(x_i;\theta) - y_0 - \frac{1}{n} \sum_{i=1}^{n} \psi(\widetilde{x}_i;\theta) + y_0 \right\|_2 \\
= &\left\| \frac{1}{n} \psi(x_1;\theta) - \psi(\widetilde{x}_1;\theta) \right\|_2 \\
\leq &\frac{2K}{n}
\end{aligned}
$$

Taking supremum over $\theta$ then gives

$$
f(x_1^n) - f(\widetilde{x}_1^n) \leq \frac{2K}{n}
$$

The other direction can be obtained with the role $x_1^n$ and $\widetilde{x}_1^n$ reversed. Therefore we get (44). By Corollary 2.21 in Wainwright (2019), we get (45). The proof is then complete. ∎

Finally, even though Theorem 7 shows fast convergence $\widehat{Q}_\rho(x) \to Q^*(x)$, one needs $\widehat{Q}_\rho(\overline{X}) \to Q^*(\mu)$ when considering power in Theorem 18, and this is stated in Lemma 32 below. A crucial observation here is that $\widehat{Q}_\rho(\overline{X})$ and $Q^*(\mu)$ are the empirical and population Fréchet mean respectively. The proof is built upon Le Gouic et al. (2022) and Altschuler et al. (2021).

**Lemma 32** *Assume Assumption 1 and 2 hold. Then there exists an event $\widetilde{E}$ with probability at least $1 - n^{-100}$ under which the following holds*

$$
W^2\left(\widehat{Q}_\rho(\overline{X}), Q^*(\mu)\right) \lesssim \frac{\operatorname{polylog}(n)}{\sqrt{n}} \tag{46}
$$

*As a result, under $\widetilde{E}$, one has*

$$
\left\| \widehat{Q}_\rho(\overline{X}) - Q^*(\mu) \right\|_{\mathrm{F}} \lesssim \frac{\operatorname{polylog}(n)}{\sqrt{n}} \tag{47}
$$

**Proof** Note that $\widehat{Q}_\rho(\overline{X})$ and $Q^*(\mu)$ are equal to the empirical and population barycenter respectively. For simplicity, we write $Q^*$ for $Q^*(\mu)$.

**Proof of (46):** From (3.8)-(3.9) in Le Gouic et al. (2022), the variance inequality in Chewi et al. (2020, Theorem 6) and results in Altschuler et al. (2021), one can obtain the following

$$W^2\left(\widehat{Q}_\rho(\overline{X}), Q^*\right) \leq C_b \left\| \frac{1}{n}\sum_{i=1}^n \left(T_{Q^*}^{Q_i} - I_d\right)\right\|_{Q^*} \tag{48}$$

where $C_b > 0$ is a constant independent of $n$ and $\|A\|_{Q^*(\mu)} := \langle A, Q^* A\rangle$. Moreover, Lemma 33 implies that $c_1^{-1} I_d \preceq Q^* \preceq c_1 I_d$; see Assumption 2 for the definition of $c_1$. As a result, one can obtain

$$\begin{aligned}\left\| \frac{1}{n}\sum_{i=1}^n \left(T_{Q^*}^{Q_i} - I_d\right)\right\|_{Q^*} &\leq \lambda_{\max}\left(Q^*\right)\cdot \left\| \frac{1}{n}\sum_{i=1}^n \left(T_{Q^*}^{Q_i} - I_d\right)\right\|_{\mathrm{F}} \\ &\leq c_\Lambda \left\| \frac{1}{n}\sum_{i=1}^n \left(T_{Q^*}^{Q_i} - I_d\right)\right\|_{\mathrm{F}} \end{aligned}\tag{49}$$

Note that by the optimality condition for barycenter, one has $\mathbb{E}\left(T_{Q^*}^{Q} - I_d\right) = 0$. Then one can apply Lemma 25 to get that

$$\begin{aligned}\left\|\left\| \frac{1}{n}\sum_{i=1}^n \left(T_{Q^*}^{Q_i} - I_d\right)\right\|_{\mathrm{F}}\right\|_{\psi_2} &\lesssim \sup_{\|U\|_{\mathrm{F}}\leq 1} \left\| \left\langle U, \frac{1}{n}\sum_{i=1}^n \left(T_{Q^*}^{Q_i} - I_d\right)\right\rangle\right\|_{\psi_2} \\ &\lesssim \sup_{\|U\|_{\mathrm{F}}\leq 1} \frac{1}{\sqrt{n}} \left\| \left\langle U, T_{Q^*}^{Q} - I_d\right\rangle\right\|_{\psi_2} \\ &\leq \frac{1}{\sqrt{n}} \left\|\left\| T_{Q^*}^{Q} - I_d\right\|_{\mathrm{F}}\right\|_{\psi_2}\end{aligned}\tag{50}$$

Recall that $T_S^Q = S^{-1/2}\left(S^{1/2} Q S^{1/2}\right)^{1/2} S^{-1/2}$, then one has

$$\begin{aligned}\left\|\left\| T_{Q^*}^{Q} - I_d\right\|_{\mathrm{F}}\right\|_{\psi_2} &\overset{(i)}{\lesssim} \left\|\left\| T_{Q^*}^{Q} - I_d\right\|_{\mathrm{op}}\right\|_{\psi_2} \\ &\leq \left\| 1 + c_\Lambda^{3/2} \lambda_{\max}\left(Q\right)^{1/2}\right\|_{\psi_2} \\ &\overset{(ii)}{\leq} \left\| 1 + c_\Lambda^{3/2} c_\Lambda^{1/2} \left\|X-\mu\right\|^{1/2}\right\|_{\psi_2} \\ &\leq \left\| 1 + c_\Lambda^{3/2} c_\Lambda^{1/2} \left(1 \vee \left\|X-\mu\right\|\right)\right\|_{\psi_2} \\ &\leq \left\| 1 + c_\Lambda^{2} \left(1 + \left\|X-\mu\right\|\right)\right\|_{\psi_2} \\ &\lesssim 1\end{aligned}\tag{51}$$

Here (i) follows since dimension $d$ is fixed and absorbed into the constant factor independent of $n$, (ii) is a result of Assumption 2.

Finally, combining (48) (49) (50) and (51) gives

$$\left\| W^2\left(\widehat{Q}_\rho(\overline{X}), Q^*(\mu)\right)\right\|_{\psi_2} \lesssim \frac{1}{\sqrt{n}} \tag{52}$$

which implies (46).

**Proof of (47):** Apply ((**iv**)) in Lemma 21 to get that under the event $\widetilde{E}$,

$$\begin{aligned}
\left\|\widehat{Q}_\rho(\overline{X}) - Q^*\right\|_{\mathrm{F}} &\leq W^2\left(\widehat{Q}_\rho(\overline{X}), Q^*\right) \cdot \frac{1 + \lambda_{\min}^{-1}\left(Q^*\right)\lambda_{\max}\left(\widehat{Q}_\rho(\overline{X})\right)}{\lambda_{\max}\left(Q^*\right)\cdot\lambda_{\min}^{-2}\left(Q^*\right)} \\
&\lesssim W^2\left(\widehat{Q}_\rho(\overline{X}), Q^*\right)\cdot\left(1 + \lambda_{\max}\left(\widehat{Q}_\rho(\overline{X})\right)\right) \\
&\leq C\frac{\sqrt{\log n}}{\sqrt{n}}\cdot\left(1 + \lambda_{\max}\left(\widehat{Q}_\rho(\overline{X})\right)\right)
\end{aligned} \tag{53}$$

for constant $C > 0$ large enough. Note that this implies

$$\begin{aligned}
\lambda_{\max}\left(\widehat{Q}_\rho(\overline{X})\right) &\leq \lambda_{\max}\left(Q^*\right) + \left\|\widehat{Q}_\rho(\overline{X}) - Q^*\right\|_{\mathrm{F}} \\
&\leq c_\Lambda + C\frac{\sqrt{\log n}}{\sqrt{n}}\cdot\left(1 + \lambda_{\max}\left(\widehat{Q}_\rho(\overline{X})\right)\right)
\end{aligned}$$

Solving for $\lambda_{\max}\left(\widehat{Q}_\rho(\overline{X})\right)$ gives

$$\begin{aligned}
\lambda_{\max}\left(\widehat{Q}_\rho(\overline{X})\right) &\leq \frac{c_\Lambda + C\frac{\sqrt{\log n}}{\sqrt{n}}}{1 - C\frac{\sqrt{\log n}}{\sqrt{n}}} \\
&\lesssim 1 \qquad \text{for } n \text{ large enough}
\end{aligned} \tag{54}$$

Finally, plugging (54) back into (53) gives (47).

■

### B.2.1 Proof of Lemma 29

**Proof of (41):** Since $f(z, \theta)$ is continuous in $\theta$ and $\Theta$ is separable, we have for any countable, dense subset $\mathbb{U} \subset \Theta$, the following equality

$$\mathbb{E}\sup_{\theta\in\Theta}|f(Z,\theta)| = \mathbb{E}\sup_{S\in\mathbb{U}}|f(Z,\theta)|$$

holds. By the monotone convergence theorem, it suffices to assume that $\mathbb{U}$ is finite and get an upper bound that is independent of the cardinality of $\mathbb{U}$.

For each $k \in \mathbb{Z}$, let $\mathbb{U}_k \subset \mathbb{U}$ be a minimal $\epsilon_k$-covering set of $\mathbb{U}$ where $\epsilon_k$ is defined by

$$\epsilon_k = \tau^{-1}(2^{-k}) \tag{55}$$

Let $N(\epsilon_k, \mathbb{U})$ be the cardinality of the minimal $\epsilon_k$-covering set $\mathbb{U}_k$ of $\mathbb{U}$. Since $\mathbb{U}$ is a subset of $\Theta$, $N(\epsilon_k, \mathbb{U})$ can be upper bounded by

$$\log N(\epsilon_k; \mathbb{U}) = \log|\mathbb{U}_k| \leq \log N(\epsilon_k; \Theta)$$

Since $\mathbb{U}$ is finite, there is a largest $\eta \in \mathbb{Z}$ and a smallest integer $H \in \mathbb{Z}$ such that

$$\mathbb{U}_\eta = \{\theta_0\} \text{ for some } \theta_0 \in \mathbb{U}, \qquad \mathbb{U}_H = \mathbb{U}$$

For each $k \in \mathbb{Z}$, define the mapping $\pi_k : \Theta \to \mathbb{U}_k$ via

$$\pi_k(\theta) = \operatorname*{argmin}_{\widetilde{\theta} \in \mathbb{U}_k} d(\widetilde{\theta}, \theta)$$

so that $\pi_k(S)$ is the best approximation of $\theta \in \Theta$ from the set $\mathbb{U}_k$.

For any $\theta \in \mathbb{U}$, apply the triangle inequality to see that

$$\begin{aligned}
\mathbb{E}\left|\max_{\theta\in\mathbb{U}} f(Z,\theta)\right| &\leq \mathbb{E}\left|\max_{\theta\in\mathbb{U}} \left(f(Z,\theta) - f(Z,\theta_0)\right)\right| + \mathbb{E}\left|f(Z;\theta_0)\right| \\
&\leq \mathbb{E}\max_{\theta\in\mathbb{U}} \left|f(Z,\theta) - f(Z,\theta_0)\right| + \mathbb{E}\left|f(Z;\theta_0)\right| \\
&\overset{(i)}{=} \mathbb{E}\max_{\theta\in\mathbb{U}} \left|\sum_{k=\eta+1}^{H} \left(f(Z,\pi_k(\theta)) - f(Z,\pi_{k-1}(\theta))\right)\right| + \mathbb{E}\left|f(Z;\theta_0)\right| \\
&\leq \mathbb{E}\max_{\theta\in\mathbb{U}} \sum_{k=\eta+1}^{H} \left|f(Z,\pi_k(\theta)) - f(Z,\pi_{k-1}(\theta))\right| + \mathbb{E}\left|f(Z;\theta_0)\right| \\
&\leq \sum_{k=\eta+1}^{H} \mathbb{E}\max_{\theta\in\mathbb{U}} \left|f(Z,\pi_k(\theta)) - f(Z,\pi_{k-1}(\theta))\right| + \mathbb{E}\left|f(Z;\theta_0)\right|
\end{aligned} \tag{56}$$

Here (i) is a consequence of decomposing $f(Z,\theta) - f(Z,\theta_0)$ as a telescoping sum

$$f(Z,\theta) - f(Z,\theta_0) = \sum_{k=\eta+1}^{H} \left(f(Z,\pi_k(\theta)) - f(Z,\pi_{k-1}(\theta))\right)$$

For any fixed $\theta \in \Theta$, one has

$$\begin{aligned}
\left\|f(\cdot,\pi_k(\theta)) - f(\cdot,\pi_{k-1}(\theta))\right\|_{\psi_2} &\leq \left\|f(\cdot,\pi_k(\theta)) - f(\cdot,\theta)\right\|_{\psi_2} + \left\|f(\cdot,\theta) - f(\cdot,\pi_{k-1}(\theta))\right\|_{\psi_2} \\
&\overset{(I)}{\leq} \tau(\epsilon_k) + \tau(\epsilon_{k-1}) \\
&\leq 2\tau(\epsilon_{k-1}) \\
&\overset{(II)}{=} 2 \cdot 2^{-(k-1)}
\end{aligned}$$

Here (I) is a result of (40), and (II) follows from (55). Then, one can obtain

$$\begin{aligned}
\mathbb{E}\max_{\theta\in\mathbb{U}} \left|f(Z,\pi_k(\theta)) - f(Z,\pi_{k-1}(\theta))\right| &\overset{(i)}{\lesssim} \sqrt{|\mathbb{U}_{k-1}| \cdot |\mathbb{U}_k|} \cdot 2 \cdot 2^{-(k-1)} \\
&\lesssim \sqrt{\log N(\epsilon_k;\Theta)} \cdot 2^{-(k-1)}
\end{aligned} \tag{57}$$

Here (i) follows from the properties of the maximum of finitely many sub-Gaussian random variables and the fact that the maximum is taken over at most $|\mathbb{U}_{k-1}| \cdot |\mathbb{U}_k| \leq N(\epsilon_k;\Theta)^2$

random variables. Therefore,

$$\begin{aligned}\sum_{k=\eta+1}^{H}\mathbb{E}\max_{\theta\in\mathbb{U}}|f(Z,\pi_k(\theta))-f(Z,\pi_{k-1}(\theta))| &\lesssim \sum_{k=\eta+1}^{H}\sqrt{\log N(\epsilon_k;\Theta)}\cdot 2^{-(k-1)}\\ &= \sum_{k=\eta+1}^{H}\sqrt{\log N(\tau^{-1}(2^{-k});\Theta)}\cdot 2^{-(k-1)}\\ &\lesssim \int_0^{\infty}\sqrt{\log N(\tau^{-1}(t);\Theta)}\cdot dt\\ &\overset{(I)}{=} \int_0^{\tau(D)}\sqrt{\log N(\tau^{-1}(t);\Theta)}\cdot dt\end{aligned}\tag{58}$$

Here (I) follows by noticing that $\log N(\tau^{-1}(t);\Theta)=0$ for any $t\geq\tau(D)$.

Combine (56) and (58) to see that

$$\mathbb{E}\sup_{\theta\in\Theta}|f(Z;\theta)|\leq C\int_0^{\tau(D)}\sqrt{\log N(\tau^{-1}(t);\Theta)}\cdot dt+\sup_{\theta\in\Theta}\mathbb{E}\,|f(Z;\theta)|$$

for some constant $C>0$.

**Proof of (42):** Consider two cases $\alpha_0\in(0,1]$ and $\alpha_0>1$.

**Case I $\alpha_0\in(0,1]$:** Note that since $\alpha_0\in(0,1]$, one has

$$\begin{aligned}\tau^{-1}(t) &= \left(\frac{\sqrt{n}t}{K}\right)\wedge\left(\frac{\sqrt{n}t}{K}\right)^{1/\alpha_0}\\ &= \begin{cases}\frac{\sqrt{n}t}{K} & \sqrt{n}t\geq K\\ \left(\frac{\sqrt{n}t}{K}\right)^{1/\alpha_0} & \sqrt{n}t\leq K\end{cases}\end{aligned}\tag{59}$$

As a result, one has

$$\begin{aligned}&\int_0^{\tau(D)}\sqrt{\log N(\tau^{-1}(t);\Theta)}\cdot dt\\ &\overset{(i)}{=}\frac{1}{\sqrt{n}}\int_0^{\sqrt{n}\tau(D)}\sqrt{\log N\left(\tau^{-1}\left(\frac{t}{\sqrt{n}}\right);\Theta\right)}\cdot dt\\ &\overset{(ii)}{=}\frac{1}{\sqrt{n}}\int_0^{K}\sqrt{\log N\left((t/K)^{1/\alpha_0};\Theta\right)}\cdot dt+\frac{1}{\sqrt{n}}\int_K^{\sqrt{n}\tau(D)}\sqrt{\log N\left(t/K;\Theta\right)}\cdot dt\\ &\lesssim\frac{1}{\sqrt{n}}\int_0^{K}\sqrt{\log^+\left(\frac{D}{(t/K)^{1/\alpha_0}}\right)}\cdot dt+\frac{1}{\sqrt{n}}\int_K^{\sqrt{n}\tau(D)}\sqrt{\log^+\left(\frac{D}{(t/K)}\right)}\cdot dt\\ &\overset{(iii)}{=}\frac{K}{\sqrt{n}}\underbrace{\int_0^{1}\sqrt{\log^+\left(\frac{D}{t^{1/\alpha_0}}\right)}\cdot dt}_{a_1}+\frac{K}{\sqrt{n}}\underbrace{\int_1^{\sqrt{n}\tau(D)/K}\sqrt{\log^+\left(\frac{D}{t}\right)}\cdot dt}_{a_2}\end{aligned}\tag{60}$$

Here (i), (iii) follows from a change of variables, and (ii) follows from (59).

- $a_1$: one has

$$\begin{aligned} a_1 &\overset{(i)}{\leq} \int_0^1 \sqrt{\log^+\left(\frac{1}{t^{1/\alpha_0}}\right)} dt + \int_0^1 \sqrt{\log^+ D} dt \\ &\overset{(ii)}{\lesssim} \sqrt{\log^+ D} \end{aligned} \tag{61}$$

Here (i) follows from the inequality $\sqrt{s+t} \leq \sqrt{s} + \sqrt{t}$ for $s, t \geq 0$, and (ii) follows from the assumption that $D \gtrsim 1$.

- $a_2$: for $D \gtrsim 1$,

$$\begin{aligned} a_2 &\overset{(i)}{\leq} \int_1^{\sqrt{n}\tau(D)/K} \sqrt{\log^+ D} dt \\ &\overset{(ii)}{\leq} \frac{\sqrt{n}\tau(D)}{K} \cdot \sqrt{\log^+ D} \\ &= (D \vee D^{\alpha_0}) \sqrt{\log^+ D} \\ &\overset{(iii)}{\lesssim} D \sqrt{\log^+ D} \end{aligned} \tag{62}$$

Here (i) a consequence of the fact that $D/t \leq D$ for $t \geq 1$, (ii) results from substituting the definition of $\tau(\cdot)$ and (iii) follows from the assumption $\alpha_0 \in (0, 1]$.

Combine (60), (61) and (62) to see that

$$\int_0^{\tau(D)} \sqrt{\log N(\tau^{-1}(t); \Theta)} \cdot dt \lesssim \frac{K}{\sqrt{n}} D \sqrt{\log^+ D}$$

which proves (42) for $\alpha_0 \in (0, 1]$.

**Case II $\alpha_0 > 1$:** for $\alpha_0 > 1$, one has

$$\begin{aligned} \tau^{-1}(t) &= \left(\frac{\sqrt{n}t}{K}\right) \wedge \left(\frac{\sqrt{n}t}{K}\right)^{1/\alpha_0} \\ &= \begin{cases} \frac{\sqrt{n}t}{K} & \sqrt{n}t \leq K \\ \left(\frac{\sqrt{n}t}{K}\right)^{1/\alpha_0} & \sqrt{n}t > K \end{cases} \end{aligned} \tag{63}$$

As a result, one can obtain

$$
\begin{aligned}
&\int_0^{\tau(D)} \sqrt{\log N(\tau^{-1}(t);\Theta)}\cdot dt \\
\overset{(i)}{=}\ & \frac{1}{\sqrt{n}}\int_0^{\sqrt{n}\tau(D)} \sqrt{\log N\left(\tau^{-1}\left(\frac{t}{\sqrt{n}}\right);\Theta\right)}\cdot dt \\
\overset{(ii)}{=}\ & \frac{1}{\sqrt{n}}\int_0^{K} \sqrt{\log N\left(t/K;\Theta\right)}\cdot dt + \frac{1}{\sqrt{n}}\int_K^{\sqrt{n}\tau(D)} \sqrt{\log N\left((t/K)^{1/\alpha_0};\Theta\right)}\cdot dt \\
\lesssim\ & \frac{1}{\sqrt{n}}\int_0^{K} \sqrt{\log^+\left(\frac{D}{t/K}\right)}\cdot dt + \frac{1}{\sqrt{n}}\int_K^{\sqrt{n}\tau(D)} \sqrt{\log^+\left(\frac{D}{(t/K)^{1/\alpha_0}}\right)}\cdot dt \\
\overset{(iii)}{=}\ & \frac{K}{\sqrt{n}}\underbrace{\int_0^{1} \sqrt{\log^+\left(\frac{D}{t}\right)}\cdot dt}_{a_3} + \frac{K}{\sqrt{n}}\underbrace{\int_1^{\sqrt{n}\tau(D)/K} \sqrt{\log^+\left(\frac{D}{t^{1/\alpha_0}}\right)}\cdot dt}_{a_4}
\end{aligned}
\tag{64}
$$

Here (i), (iii) follows from a change of variables, and (ii) follows from (63).

- $a_3$: one has

$$
\begin{aligned}
a_3 &\overset{(i)}{\leq} \int_0^1 \sqrt{\log^+\left(\frac{1}{t}\right)}dt + \int_0^1 \sqrt{\log^+ D}dt \\
&\overset{(ii)}{\lesssim} \sqrt{\log^+ D}
\end{aligned}
\tag{65}
$$

  Here (i) follows from the inequality $\sqrt{s+t} \leq \sqrt{s} + \sqrt{t}$ for $s, t \geq 0$, and (ii) follows from the assumption that $D \gtrsim 1$.

- $a_4$: for $D \gtrsim 1$,

$$
\begin{aligned}
a_4 &\overset{(i)}{\leq} \int_1^{\sqrt{n}\tau(D)/K} \sqrt{\log^+ D}dt \\
&\overset{(ii)}{\leq} \frac{\sqrt{n}\tau(D)}{K}\cdot\sqrt{\log^+ D} \\
&= (D \vee D^{\alpha_0})\sqrt{\log^+ D} \\
&\overset{(iii)}{\lesssim} D^{\alpha_0}\sqrt{\log^+ D}
\end{aligned}
\tag{66}
$$

  Here (i) a consequence of the fact that $D/\left(t^{1/\alpha_0}\right) \leq D$ for $t \geq 1$, (ii) results from substituting the definition of $\tau(\cdot)$ and (iii) follows from the assumption $\alpha_0 > 1$.

Combine (64), (65) and (66) to see that

$$
\int_0^{\tau(D)} \sqrt{\log N(\tau^{-1}(t);\Theta)}\cdot dt \lesssim \frac{K}{\sqrt{n}} D^{\alpha_0}\sqrt{\log^+ D}
$$

which proves (42) for $\alpha_0 > 1$.

**Proof of (43):** Let $\Psi_n(\theta) := \frac{1}{n}\sum_{i=1}^n \psi(Z_i;\theta)$, then one has

$$
\begin{aligned}
&\left\| f(Z_1^n;\theta) - f(Z_1^n;\widetilde{\theta}) \right\|_{\psi_2} \\
&= \left\| \left\| \Psi_n(\theta) - \mathbb{E}\Psi_n(\theta) \right\| - \left\| \Psi_n(\widetilde{\theta}) - \mathbb{E}\Psi_n(\widetilde{\theta}) \right\| \right\|_{\psi_2} \\
&\le \left\| \left\| \Psi_n(\theta) - \mathbb{E}\Psi_n(\theta) - \Psi_n(\widetilde{\theta}) + \mathbb{E}\Psi_n(\widetilde{\theta}) \right\| \right\|_{\psi_2} \\
&\overset{(i)}{\lesssim} \sup_{u,v\in\mathbb{S}^{m-1}} \left\| \left\langle u, \left[\widetilde{\Psi}_n(\theta) - \mathbb{E}\widetilde{\Psi}_n(\theta)\right] - \left[\widetilde{\Psi}_n(\widetilde{\theta}) + \widetilde{\Psi}_n(\widetilde{\theta})\right] \cdot v^{\otimes k} \right\rangle \right\|_{\psi_2} \\
&\overset{(ii)}{\lesssim} \frac{1}{\sqrt{n}} \sup_{u,v\in\mathbb{S}^{m-1}} \left\| \left\langle u, \left[\psi(Z;\theta) - \psi(Z;\widetilde{\theta})\right] - \mathbb{E}\left[\psi(Z;\theta) - \psi(Z;\widetilde{\theta})\right] \cdot v^{\otimes k} \right\rangle \right\|_{\psi_2} \\
&\overset{(iii)}{\lesssim} \frac{1}{\sqrt{n}} \sup_{u,v\in\mathbb{S}^{m-1}} \left\| \left\langle u, \left[\psi(Z;\theta) - \psi(Z;\widetilde{\theta})\right] \cdot v^{\otimes k} \right\rangle \right\|_{\psi_2} \\
&\le \frac{1}{\sqrt{n}} \sup_{v\in\mathbb{S}^{m-1}} \left\| \left\| \left[\psi(Z;\theta) - \psi(Z;\widetilde{\theta})\right] \cdot v^{\otimes k} \right\|_{\mathrm{F}} \right\|_{\psi_2} \\
&\overset{(iv)}{\le} \frac{1}{\sqrt{n}} \sup_{v\in\mathbb{S}^{m-1}} \left\| \left\| \psi(Z;\theta) - \psi(Z;\widetilde{\theta}) \right\| \cdot \|v\|_{\mathrm{F}}^k \right\|_{\psi_2} \\
&\le \frac{1}{\sqrt{n}} \left\| \left\| \psi(Z;\theta) - \psi(Z;\widetilde{\theta}) \right\| \right\|_{\psi_2}
\end{aligned}
$$

Here (i) is a result of Lemma 25, (ii) arises due to independence, (iii) follows from Lemma 2.6.8 in Vershynin (2018), and (iv) is derived from the definition of $\|\cdot\|$. The proof is then complete.

### B.3 Properties of $F$ and $Q^*$

This section collects properties of $F(\cdot,\cdot)$ and $Q^*(\cdot)$ that are needed later in the proof.

**Lemma 33** *Assume Assumption 1 and 2 holds. Then the following inequalities*

$$\gamma_\Lambda(\|x-\mu\|)^{-1} I_d \preceq Q^*(x) \preceq \gamma_\Lambda(\|x-\mu\|) I_d, \qquad \forall x \in \operatorname{supp} X \tag{67}$$

*Moreover, there exist constant $C_0^*, C_1^* > 0$ which only depend on $c_\Sigma, C_{\psi_2}$ from Assumption 1 and $c_\Lambda, C_1$ (from Assumption 2) such that*

$$Q^*(x) \preceq \left[C_0^* + C_1^* \|x-\mu\|\right]^2 I_d \tag{68}$$

**Proof** Without loss of generality, assume $X$ have expectation $\mu = 0$ and covariance $\Sigma = I_p$. Denote $\lambda_1(x) = \lambda_{\max}(Q^*(x))$ and $\lambda_d(x) = \lambda_{\min}(Q^*(x))$
*Proof of (67):* By the optimality condition of $Q^*(x) = \operatorname{argmin}_S \mathbb{E}\left[W^2(S,Q) \middle| X = x\right]$. By the differential properties of $W^2$ in Lemma 21, one can obtain

$$I_d = \mathbb{E}\left[T_{Q^*(x)}^{Q} \middle| X = x\right]$$

Recall that $T_{Q^*(x)}^{Q} = Q^*(x)^{-1/2}\left(Q^*(x)^{1/2}QQ^*(x)^{1/2}\right)^{1/2}Q^*(x)^{-1/2}$, multiplying both sides of the equation with $Q^*(x)^{1/2}$ then gives

$$Q^*(x) = \mathbb{E}\left[\left(Q^*(x)^{1/2}QQ^*(x)^{1/2}\right)^{1/2}|X = x\right]$$

Then one can obtain

$$\begin{aligned}\lambda_1(x) &= \lambda_{\max}\left(\mathbb{E}\left[\left(Q^*(x)^{1/2}QQ^*(x)^{1/2}\right)^{1/2} \mid X = x\right]\right)\\ &\leq \mathbb{E}\left[\lambda_{\max}\left(\left(Q^*(x)^{1/2}QQ^*(x)^{1/2}\right)^{1/2}\right) \mid X = x\right]\\ &\leq \gamma_\Lambda(\|x-\mu\|)^{1/2}\lambda_1(x)^{1/2}\end{aligned}$$

Here the second line follows from the convexity of the largest eigenvalue function $\lambda_{\max}(\cdot)$ over PSD matrices. The last inequality follows from Assumption 2. Therefore, one can readily obtain $\lambda_1(x) \leq \gamma_\Lambda(\|x-\mu\|)$. The lower bound $\lambda_d(x) \geq \gamma_\Lambda(\|x-\mu\|)^{-1}$ can be derived similarly by the concavity of the function $\lambda_{\min}(\cdot)$.

*Proof of (68):* By the optimality condition of $Q^*(x) = \operatorname{argmin}_S \mathbb{E}w(x,X)W^2(S,Q)$ , one has

$$0 = \mathbb{E}w(x,X)\left(T_{Q^*(x)}^{Q} - I_d\right)$$

Note that $\mathbb{E}w(x,X) = 1$ for any $x$, hence one has

$$I_d = \mathbb{E}w(x,X)T_{Q^*(x)}^{Q}$$

Recall that $T_{Q^*(x)}^{Q} = Q^*(x)^{-1/2}\left(Q^*(x)^{1/2}QQ^*(x)^{1/2}\right)^{1/2}Q^*(x)^{-1/2}$, multiplying both sides of the equation with $Q^*(x)^{1/2}$ then gives

$$Q^*(x) = \mathbb{E}w(x,X)\left(Q^*(x)^{1/2}Q_iQ^*(x)^{1/2}\right)^{1/2}$$

Taking the largest eigenvalue on both sides, one has

$$\begin{aligned}\lambda_1(x) &\leq \mathbb{E}\left|w(x,X)\right| \cdot \left\|\left(Q^*(x)^{1/2}QQ^*(x)^{1/2}\right)^{1/2}\right\|_{\text{op}}\\ &\leq \mathbb{E}\left|w(x,X)\right| \cdot \lambda_{\max}(Q)^{1/2}\lambda_1^{1/2}(x)\end{aligned}$$

which implies

$$\lambda_1(x) \leq \left[\mathbb{E}\left(1 + \|x\| \cdot \|X\|\right)\lambda_{\max}(Q)^{1/2}\right]^2$$

Under Assumption 1 and Assumption 2, we have $\lambda_{\max}(Q_i) \leq \gamma_\Lambda(\|X_i\|)$, which implies that

$$\begin{aligned}\lambda_1(x) &\leq \left[\mathbb{E}\gamma_\Lambda(\|X\|)^{1/2} + \|x\|\,\mathbb{E}\,\|X\|\,\gamma_\Lambda(\|X\|)^{1/2}\right]^2\\ &\leq \left[C_0^* + C_1^*\,\|x\|\right]^2\end{aligned}$$

where $C_0^*, C_1^* > 0$ are finite constants only depend on $c_\Sigma, C_{\psi_2}$ from Assumption 1 and $c_\Lambda, C_\Lambda$ (from Assumption 2). ∎

**Lemma 34** *Assume Assumption 1 and 2 hold. If $X$ and $Q$ are independent, then the following holds.*

- $Q^*(x) \equiv Q^*(\mu)$
- *Assumption 5 holds for $C_\lambda = 0$ and $c_\lambda$ large enough.*
- *Assumption 4 holds for $\delta_F = 1$, $\alpha_F = 2$, $C_F = 1$ and some constant $c_F$ large enough.*

**Proof** Independence between $X$ and $Q$ implies that

$$
\begin{aligned}
F(x,S) &= \mathbb{E}w(x,X)W^2(S,Q) \\
&= \mathbb{E}w(x,X)\cdot\mathbb{E}W^2(S,Q) \\
&= \mathbb{E}W^2(S,Q)
\end{aligned}
$$

The existence and uniqueness of the minimizer of $F(x,\cdot)$ follow from Panaretos and Zemel (2020, Proposition 3.2.3, Proposition 3.2.7). Moreover, since $\mathbb{E}W^2(S,Q)$ does not depend on $x$, we have $Q^*(x) \equiv Q^*(\mu)$. We denote $Q^* := Q^*(\mu)$ from now on. Note that Lemma 33 implies that $Q^* \in \mathcal{S}_d(c_\Lambda^{-1}, c_\Lambda)$

**Verification of Assumption 5:** Again by independence, one has

$$
\mathbb{E}\left[-w(x,X)dT_{Q^*}^{Q}\right] = \mathbb{E}\left[-dT_{Q^*}^{Q}\right]
$$

Therefore, one can obtain

$$
\begin{aligned}
\lambda_{\min}\left(\mathbb{E}\left[-w(x,X)dT_{Q^*}^{Q}\right]\right) &= \lambda_{\min}\left(\mathbb{E}\left[-dT_{Q^*}^{Q}\right]\right) \\
&\overset{(i)}{\geq} \mathbb{E}\frac{\lambda_{\min}^{1/2}(Q^{1/2}Q^*Q^{1/2})}{2}\cdot\lambda_{\min}^2(Q^{*-1}) \\
&\gtrsim \mathbb{E}\lambda_{\min}^{1/2}(Q) \\
&\overset{(ii)}{\gtrsim} \mathbb{E}\left[\gamma_\Lambda(\|X-\mu\|)\right]^{-1/2} \\
&\gtrsim \mathbb{E}\frac{1}{1\vee\|X-\mu\|^{C_\Lambda/2}} \\
&\overset{(iii)}{\geq} c_{\text{eig}}
\end{aligned}
$$

for some constant $c_{\text{eig}}$ small enough. Here (i) follows form Lemma 21, (ii) is a result of Assumption 2 and (iii) is due to Assumption 1. The proof is then complete.

**Verification of Assumption 4:** In order to verify (13). Let us denote $Q' = Q^{*-1/2} S Q^{*-1/2}$. Then ((**ii**)) and ((**iii**)) in Lemma 21 imply the following lower bound.

$$
\begin{aligned}
& F(x,S) - F(x,Q^*) \\
=& \mathbb{E}\left[W^2(S,Q) - W^2(Q^*,Q)\right] \\
\geq& \mathbb{E}\left\langle I_d - T_{Q^*}^{Q}, S - Q^* \right\rangle + \mathbb{E}\frac{2}{\left(1+\lambda_{\max}^{1/2}(Q'(x))\right)^2}\left\langle -dT_{Q^*}^{Q}(S-Q^*), S-Q^*\right\rangle \\
\overset{(i)}{\geq}& \mathbb{E}\frac{2}{\left(1+\lambda_{\max}^{1/2}(Q'(x))\right)^2}\cdot\frac{\lambda_{\min}^{1/2}\left(Q^{1/2}Q^*Q^{1/2}\right)}{2}\cdot\left\|Q^{*-1/2}(S-Q^*)Q^{*-1/2}\right\|_{\mathrm{F}}^2
\end{aligned}
\tag{69}
$$

Here (i) follows from the fact that $\mathbb{E}(T_{Q^*}^{Q} - I_d) = 0$ which is a consequence of the optimality condition and independence.

Triangle inequality implies that

$$
\left\{S \in \mathcal{S}_d^{++} : \delta \leq \|S - Q^*\|_{\mathrm{F}} \leq \Delta\right\} \subset \left\{S \in \mathcal{S}_d^{++} : \|S\|_{\mathrm{op}} \leq c_\Lambda + \Delta\right\}
$$

which combined with (69) implies for any $\Delta \geq \delta$,

$$
\begin{aligned}
& \inf_{\delta \leq \|S-Q^*\|_{\mathrm{F}} \leq \Delta} F(x,S) - F(x,Q^*) \\
\geq& \inf_{\delta \leq \|S-Q^*\|_{\mathrm{F}} \leq \Delta} \mathbb{E}\frac{2}{\left(1+\lambda_{\max}^{1/2}(Q')\right)^2}\cdot\frac{\lambda_{\min}^{1/2}\left(Q^{1/2}Q^*Q^{1/2}\right)}{2}\cdot\left\|Q^{*-1/2}(S-Q^*)Q^{*-1/2}\right\|_{\mathrm{F}}^2 \\
\overset{(i)}{\gtrsim}& \inf_{\delta \leq \|S-Q^*\|_{\mathrm{F}} \leq \Delta} \frac{1}{\left(1+\Delta^{1/2}\right)^2}\cdot\|S-Q^*\|_{\mathrm{F}}^2 \\
\overset{(ii)}{\gtrsim}& \inf_{\delta \leq \|S-Q^*\|_{\mathrm{F}} \leq \Delta} \frac{1}{1+\Delta}\cdot\|S-Q^*\|_{\mathrm{op}}^2 \\
\geq& c_{\mathrm{sep}}\frac{1}{\Delta}\delta^2
\end{aligned}
$$

where $c_{\mathrm{sep}} > 0$ is a constant independent of $n$ that is small enough. Here (i) follows from the inequalities that $\lambda_{\max}(Q') \lesssim \lambda_{\max}(S) \lesssim \Delta$, $\lambda_{\min}\left(Q^{1/2}Q^*Q^{1/2}\right) \gtrsim \lambda_{\min}(Q)$ and $\mathbb{E}\lambda_{\min}(Q)^{1/2} \gtrsim 1$; (ii) holds by choosing $\delta_F = 1$ so that $\Delta \geq \delta_F = 1$.

Combining results above, Assumption 4 holds with with $\delta_F = 1$, $\alpha_F = 2$, $C_F = 1$ and $c_F = 1/c_{\mathrm{sep}}$. The proof is then complete.

■

Recall the definition of $\gamma_\Lambda(\cdot)$ from Assumption 2 and the definition of $\alpha_F, \delta_F, C_F$ from Assumption 4. Denote $M_L := \gamma_\Lambda(L)$ for any $L > 0$. With these notations in place, we now present the continuity theorem for $Q^*(\cdot)$ as follows.

**Lemma 35 (Hölder continuity of $Q^*(\cdot)$)** *Suppose Assumption 1-5 hold. Then for any $L \geq e$ that satisfies $M_L > \delta_F$, any $x, \widetilde{x} \in B_\mu(L)$,*

$$\|Q^*(x) - Q^*(\widetilde{x})\| \leq C_H \max\left\{ \left(L^{C_F} M_L^{C_F+1}\right)^{1/\alpha_F} \|x - \widetilde{x}\|^{1/\alpha_F}, L^{C_F} M_L^{C_F+1} \|x - \widetilde{x}\| \right\} \quad (70)$$

*holds as long as the constant $C_H$ is large enough. Here $C_H$ is independent of $n$ but depends on $d$.*

**Proof** Note that $M_L \geq 1$ by the properties of $\gamma_\Lambda$ in Assumption 2. The proof is divided into 3 steps.

1. First, we should that $F(x, S)$ is Lipschitz in $x$ for any fixed $S \in \mathcal{S}_d(M_L^{-1}, M_L)$.
2. Next, the Lipschitzness in step 1 and Assumption 4 imply the local Hölder continuity of $Q^*(x)$ with respect to $x$.
3. Then, an argument based on linear interpolation between $x$ and $\widetilde{x}$ implies Lipschitz continuity for $x, \widetilde{x}$ separated far apart.

Combining the 3 step above finally gives (70).

To start with, note that Lemma 33 implies

$$Q^*(x) \in \mathcal{S}_d(M_L^{-1}, M_L), \quad \text{for any } x \in B(\mu, L)$$

Recall that $F(x, S) = \mathbb{E}\left[w(x, X) W^2(S, Q)\right]$.

**1. $F$ is Lipschitz in $x$:** for any $x, \widetilde{x} \in B_\mu(L)$, one can obtain

$$\begin{aligned}
|F(x, S) - F(\widetilde{x}, S)| &= \left|\mathbb{E}(x - \widetilde{x})\Sigma^{-1}(X - \mu) W^2(S, Q)\right| \\
&\lesssim \|x - \widetilde{x}\| \cdot \mathbb{E}\left\|(X - \mu) W^2(S, Q)\right\| \\
&\overset{(i)}{\lesssim} \|x - \widetilde{x}\| \cdot \mathbb{E}\|X - \mu\| \left(\lambda_{\max}(S) + \lambda_{\max}(Q)\right) \\
&= \|x - \widetilde{x}\| \cdot \left[\mathbb{E}\|X - \mu\| \lambda_{\max}(S) + \mathbb{E}\|X - \mu\| \lambda_{\max}(Q)\right] \\
&\overset{(ii)}{\lesssim} \|x - \widetilde{x}\| \left(\lambda_{\max}(S) + 1\right)
\end{aligned} \quad (71)$$

Here (i) follows from (**(i)**) in Lemma 21, and (ii) follows from the concentration of $X$ (Assumption 1) and boundedness of $Q$ given $X$ (Assumption 2).

**2. Local Hölder continuity:** for any $x, \widetilde{x} \in B_\mu(L)$, one has

$$\begin{aligned}
0 \leq & F(x, Q^*(\widetilde{x})) - F(x, Q^*(x)) \\
&= \left(F(x, Q^*(\widetilde{x})) - F(\widetilde{x}, Q^*(\widetilde{x}))\right) + \underbrace{\left(F(\widetilde{x}, Q^*(\widetilde{x})) - F(\widetilde{x}, Q^*(x))\right)}_{\leq 0} + \left(F(\widetilde{x}, Q^*(x)) - F(x, Q^*(x))\right) \\
&\leq |F(x, Q^*(\widetilde{x})) - F(\widetilde{x}, Q^*(\widetilde{x}))| + |F(\widetilde{x}, Q^*(x)) - F(x, Q^*(x))| \\
&\overset{(i)}{\lesssim} \|x - \widetilde{x}\| \cdot \left(\lambda_{\max}(Q^*(x)) + \lambda_{\max}(Q^*(\widetilde{x})) + 2\right) \\
&\overset{(ii)}{\leq} C M_L \|x - \widetilde{x}\|
\end{aligned} \quad (72)$$

provided that constant $C > 0$ is large enough. Here (i) follows from (71) and (ii) follows since $Q^*(x), Q^*(\widetilde{x}) \in \mathcal{S}_d(M_L^{-1}, M_L)$ for $x, \widetilde{x} \in B_\mu(L)$ by Lemma 33.

Note also that for any $x, \widetilde{x} \in B_\mu(L)$, one has $\|Q^*(x) - Q^*(\widetilde{x})\|_F \le \sqrt{d}M_L$. Recall the definition of $\delta_F, \alpha_F, \gamma_2(\cdot,\cdot)$ from Assumption 4. Then for any $x, \widetilde{x} \in B_\mu(L)$ that satisfy

$$\|x - \widetilde{x}\| \le \frac{\delta_F^{\alpha_F}}{CM_L\gamma_2(L, \sqrt{d}M_L)} =: t_0 \tag{73}$$

one can obtain

$$\|Q^*(x) - Q^*(\widetilde{x})\|_F \le \left(CM_L\gamma_2(L, \sqrt{d}M_L)\right)^{1/\alpha_F} \cdot \|x - \widetilde{x}\|^{1/\alpha_F} \tag{74}$$

To see this, note that first we have $\|Q^*(x) - Q^*(\widetilde{x})\|_F < \delta_F$ since otherwise Assumption 4 then implies that

$$F(x, Q^*(\widetilde{x})) - F(x, Q^*(x)) > \frac{\delta_F^{\alpha_F}}{\gamma_2(L, \sqrt{d}M_L)} \overset{(i)}{\ge} CM_L \|x - \widetilde{x}\|$$

Here $(i)$ results from (73). This is a contradiction with (72). Next applying Assumption 4 with $\delta = \|Q^*(x) - Q^*(\widetilde{x})\|_F$ and $\Delta = \sqrt{d}M_L$ gives

$$F(x, Q^*(\widetilde{x})) - F(x, Q^*(x)) \ge \frac{\|Q^*(x) - Q^*(\widetilde{x})\|^{\alpha_F}}{\gamma_2(L, \sqrt{d}M_L)}$$

which combined with (71) implies (74).

**3.** $x, \widetilde{x}$ **far apart:** for any $x, \widetilde{x} \in B_\mu(L)$ such that $\|x - \widetilde{x}\| > t_0$, let $K := \lceil \|x - \widetilde{x}\| / t_0 \rceil$ and

$$x_k := \left(1 - \frac{k}{K}\right) x + \frac{k}{K}\widetilde{x}, \quad k = 0, \ldots, K$$

Then $\|x_{k+1} - x_k\| \le t_0$, and one can apply (74) to see that

$$\begin{aligned}
\|Q^*(x) - Q^*(\widetilde{x})\| &\le \sum_{k=0}^{K-1} \|Q^*(x_{k+1}) - Q^*(x_k)\| \\
&\le \sum_{k=0}^{K-1} \left(CM_L\gamma_2(L, \sqrt{d}M_L)\right)^{1/\alpha_F} \cdot \|x_{k+1} - x_k\|^{1/\alpha_F} \\
&\le \sum_{k=0}^{K-1} \left(CM_L\gamma_2(L, \sqrt{d}M_L)\right)^{1/\alpha_F} \cdot t_0^{1/\alpha_F} \\
&= K\delta_F \\
&\le 2\frac{\|x - \widetilde{x}\|}{t_0}\delta_F \\
&\lesssim M_L\gamma_2(L, \sqrt{d}M_L) \cdot \|x - \widetilde{x}\|
\end{aligned} \tag{75}$$

Here the last line follows since $\delta_F$ is a constant defined in Assumption 4.

Finally, combine (74) and (75) to see that for any $x, \widetilde{x} \in B_\mu(L)$, one has

$$\|Q^*(x) - Q^*(\widetilde{x})\| \le C_H \cdot \max\left\{(M_L\gamma_2(L, M_L))^{1/\alpha_F} \|x - \widetilde{x}\|^{1/\alpha_F}, M_L\gamma_2(L, M_L) \|x - \widetilde{x}\|\right\}$$

for some constant $C_H$ independent of $n$ (but depends on $d$). ■

## Appendix C. Proof of Theorem 7 and Corollary 10

### C.1 Proof outline for uniform convergence

We start by introducing some notations. Define

$$\begin{aligned}
\widehat{F}_\rho(x, S) &= \frac{1}{n}\sum_{i=1}^n w_{n,\rho}(x, X_i) W^2(S, Q_i) \\
\widehat{A}_\rho &:= \frac{1}{n}\sum_{i=1}^n w_{n,\rho}(x, X_i)\left(T_{Q^*(x)}^{Q_i} - I_d\right) \\
\widehat{\Phi}_\rho(x) &:= \frac{1}{n}\sum_{i=1}^n w_{n,\rho}(x, X_i) dT_{Q^*(x)}^{Q_i} \\
\widehat{\Psi}_\rho(x, S) &:= \frac{1}{n}\sum_{i=1}^n w_{n,\rho}(x, X_i) d^2 T_S^{Q_i}
\end{aligned} \tag{76}$$

and

$$\begin{aligned}
\widetilde{F}_n(x, S) &:= \frac{1}{n}\sum_{i=1}^n w(x, X_i) W^2(S, Q_i) \\
\widetilde{A}_n(x) &:= \frac{1}{n}\sum_{i=1}^n w(x, X_i)\left(T_{Q^*(x)}^{Q_i} - I_d\right) \\
\widetilde{\Phi}_n(x) &:= \frac{1}{n}\sum_{i=1}^n w(x, X_i) dT_{Q^*(x)}^{Q_i} \\
\widetilde{\Psi}_n(x, S) &:= \frac{1}{n}\sum_{i=1}^n w(x, X_i) d^2 T_S^{Q_i}
\end{aligned} \tag{77}$$

For any $t \ge 1$, define the event $E_0(t)$ as

$$E_0(t) := \left\{\|X_i - \mu\| \le t, Q_i \in \mathcal{S}_d(M_t^{-1}, M_t), i \in [n]\right\}$$

where $M_t := \gamma_\Lambda(t)$ and $\gamma_\Lambda(\cdot)$ is defined in Assumption 2. Specifically, let $L_\tau = \sqrt{(1+\tau)\log n}$ for $\tau \ge 0$ and define

$$E_0 = E_0\left(C_{\psi_2} L_\tau\right), \tag{78}$$

Under Assumption 1 and 2, we have $\mathbb{P}(E_0^c) \le 2n^{-(1+\tau)}$.

We now give an outline of the proof strategy for our uniform convergence theory, namely, Theorems 7 and Corollary 10.

- Lemma 36 establishes a tail probability bound for the largest eigenvalue of $\widehat{Q}_\rho(x)$.
- Lemma 37 provides uniform concentration guarantees for $\widehat{F}_\rho$.
- Lemma 38 demonstrates uniform consistency of $\widehat{Q}_\rho(x)$, albeit with a potentially slow rate;
- Lemma 40-42 derive upper bounds for quantities associated with $\widehat{A}_\rho(x)$, $\widehat{\Phi}_\rho(x)$ and $\widehat{\Psi}_\rho(x; S)$.
- Lemma 43 arrives at the non-asymptotic $\sqrt{n}$-rate uniform convergence in the Frobenius norm with high probability.
- Lemma 44 combines Lemma 36 and 43 to obtain a $\sqrt{n}$-rate moment bound for the uniform Frobenius error.
- Lemma 45 generalizes the preceding moment result to the Wasserstein distance.

Theorem 7 and Corollary 10 then follow immediately by combining Lemmas 36-45.

**Lemma 36** *Suppose Assumption 1-2 hold. If $n \geq 3$ and $1 \leq L \leq \sqrt{n}$, then there exist constants $C_b, c_b, c_e > 0$ (independent of $n$) such that for $t_0 = C_b L$, the following hold:*

- ***Case** $\rho = 0$**:** for all $t \geq t_0$, the tail probability satisfies*

$$\mathbb{P}\left(\sup_{x \in B_\mu(L)} \widehat{\lambda}_1(x) \geq t^2\right) \lesssim h_0(t) + \exp(-c_e n)$$

- ***Case** $\rho = n^{-1}$**:** for all $t \geq t_0$, the tail probability satisfies*

$$\mathbb{P}\left(\sup_{x \in B_\mu(L)} \widehat{\lambda}_1(x) \geq t^2\right) \lesssim \begin{cases} h_0(t) + \exp(-c_e n), & t \in [t_0, n t_0] \\ h_0(t) + \exp(-c_e n) \wedge h_0(t/n), & t \in (n t_0, +\infty) \end{cases}$$

*where in the above two cases, $h_0 : \mathbb{R}_+ \to \mathbb{R}_+$ is defined as*

$$h_0(t) := \exp\left(-\sqrt{\frac{t}{c_b L}}\right) + n \cdot \exp\left[-\left(\frac{t}{c_b L}\right)^{2/(C_\Lambda + 2)}\right]$$

*As a result, denote*

$$\widetilde{E}_0 := \left\{\sup_{x \in B_\mu(L)} \lambda_{\max}\left(\widehat{Q}_\rho(x)\right) < \widetilde{M}_L\right\} \tag{79}$$

$$\text{where} \quad \widetilde{M}_L := (c_b \vee C_b)^2 \, L^2 L_\tau^{4 + (4 \vee 2C_1)}$$

*then*

$$\mathbb{P}(\widetilde{E}_0^c) \leq C_{\lambda,\tau} n^{-\tau} \tag{80}$$

*for some constant $C_{\lambda,\tau}$ independent of $n$.*

**Proof** See Appendix C.2. ■

Denote $F(x, S) = \mathbb{E}\widetilde{F}_n(x, S)$.

**Lemma 37** *Instate the notations and assumptions in Theorem 7 and Lemma 36. Then for* $\rho \in \{0, n^{-1}\}$ *and any* $\tau > 0$*,*

$$\sup_{\substack{x \in B_\mu(L) \\ S \in \mathcal{S}_d(0, \widetilde{M}_L)}} \left| \widehat{F}_\rho(x, S) - F(x, S) \right| \leq C_{F,\tau} \frac{L\widetilde{M}_L^3}{\sqrt{n}}$$

*with probability greater than* $1 - c_{F,\tau} n^{-\tau}$ *for constants* $C_{F,\tau}, c_{F,\tau}$ *independent of* $n$*.*

**Proof** See Appendix C.3. ■

**Lemma 38** *Instate the notations and assumptions in Theorem 7 and Lemma 37. Let* $1 \leq L = O(\sqrt{\log n})$*. Then for* $\rho \in \{0, n^{-1}\}$ *and any* $\tau > 0$*, there exists an event* $\widetilde{E}_1 \subset \widetilde{E}_0$ *with probability greater than* $1 - c_{\delta,\tau} n^{-\tau}$ *under which*

$$\sup_{x \in B_\mu(L)} \left\| \widehat{Q}_\rho(x) - Q^*(x) \right\|_{\mathrm{F}} \leq C_{\delta,\tau} \frac{\operatorname{polylog}(n)}{n^{1/(2\alpha_F)}} \tag{81}$$

*for constants* $C_{\delta,\tau}, c_{\delta,\tau} > 0$ *independent of* $n$*. As a result, under* $\widetilde{E}_1$*,*

$$\widehat{Q}_\rho(x) \in \mathcal{S}_d\left((C_{\text{slow}} M_L)^{-1}, C_{\text{slow}} M_L\right), \quad \forall x \in B_\mu(L) \tag{82}$$

*for some constant* $C_{\text{slow}} > 0$ *independent of* $n$*.*

**Proof** See Appendix C.4. ■

**Remark 39** *We remind readers that in Lemma 38,* $\alpha_F \geq 1$ *is defined in Assumption 4. Therefore, Lemma 38 might only lead to a uniform convergence rate slower than* $\sqrt{n}$*. For example, it is shown in Lemma 34 that* $\alpha_F = 2$ *when* $X$ *and* $Q$ *are independent, which results by (81) in a uniform convergence rate of* $n^{1/4}$*. Such a slow rate is not enough to derive the asymptotic null distribution of our test statistic in Theorem 16. Therefore, we apply a finer analysis in the following part of the proof to further boost the uniform convergence rate to* $n^{1/2}$*.*

**Lemma 40** *Instate the notations and assumptions in Theorem 7 and Lemma 38. Then there exists an event* $E_{2,1} \subset \widetilde{E}_1$ *with probability greater than* $1 - c_{\tau,1} n^{-\tau}$ *under which*

$$\sup_{x \in B_\mu(L)} \left\| \widetilde{A}_n(x) \right\|_{\mathrm{F}} \leq C_{\tau,1} \frac{\operatorname{polylog}(n)}{\sqrt{n}} \tag{83}$$

$$\sup_{x \in B_\mu(L)} \left\| \widetilde{\Phi}_n(x) - \mathbb{E}\widetilde{\Phi}_n(x) \right\| \leq C_{\tau,1} \frac{\operatorname{polylog}(n)}{\sqrt{n}} \tag{84}$$

$$\sup_{\substack{x\in B_\mu(L)\\ S\in\mathcal{S}_d\left((C_{\rm slow}M_L)^{-1},C_{\rm slow}M_L\right)}} \left\|\left|\widetilde{\Psi}_n(x;S)-\mathbb{E}\widetilde{\Psi}_n(x;S)\right|\right\| \le C_{\tau,1}\frac{\mathrm{polylog}\,(n)}{\sqrt{n}} \tag{85}$$

$$\sup_{\substack{x\in B_\mu(L)\\ S\in\mathcal{S}_d\left((C_{\rm slow}M_L)^{-1},C_{\rm slow}M_L\right)}} \left\|\left|\mathbb{E}\widetilde{\Psi}_n(x;S)\right|\right\| \le C_{\tau,1}\mathrm{polylog}\,(n) \tag{86}$$

*for constants $C_{\tau,1}, c_{\tau,1}$ independent of $n$.*

**Proof** See Appendix C.5. ■

**Lemma 41** *Instate the notations and assumptions in Theorem 7 and Lemma 38. Then there exists event $E_{2,2}\subset\widetilde{E}_1$ with probability greater than $1-c_{\tau,2}n^{-\tau}$ under which*

$$\sup_{x\in B_\mu(L)}\left\|\widehat{A}_\rho(x)-\widetilde{A}_n(x)\right\|_{\rm F} \le C_{\tau,2}\frac{\mathrm{polylog}\,(n)}{\sqrt{n}} \tag{87}$$

$$\sup_{x\in B_\mu(L)}\left\|\left|\widehat{\Phi}_\rho(x)-\widetilde{\Phi}_n(x)\right|\right\| \le C_{\tau,2}\frac{\mathrm{polylog}\,(n)}{\sqrt{n}} \tag{88}$$

$$\sup_{\substack{x\in B_\mu(L)\\ S\in\mathcal{S}_d\left((C_{\rm slow}M_L)^{-1},C_{\rm slow}M_L\right)}} \left\|\left|\widehat{\Psi}_\rho(x;S)-\widetilde{\Psi}_n(x;S)\right|\right\| \le C_{\tau,2}\frac{\mathrm{polylog}\,(n)}{\sqrt{n}} \tag{89}$$

*for constants $C_{\tau,2}, c_{\tau,2}$ independent of $n$.*

**Proof** See Appendix C.6. ■

With Lemma 40 and 41 in hand, we define event $\widetilde{E}_2 := E_{2,1}\cap E_{2,2}$ under which $\widehat{A}_\rho(x)$, $\widehat{\Psi}_\rho(x;S)$ and $\widehat{\Phi}_\rho^{-1}(x)$ are uniformly bounded. This is summarized in Lemma 42.

**Lemma 42** *Instate the notations and assumptions in Theorem 7, Lemma 38, 40 and 41. Then $\mathbb{P}(\widetilde{E}_2^c)\le\widetilde{c}_{\tau,2}n^{-\tau}$ and under $\widetilde{E}_2$ the following hold:*

$$\sup_{x\in B_\mu(L)}\left\|\widehat{A}_\rho(x)\right\|_{\rm F} \le \widetilde{C}_{\tau,2}\frac{\mathrm{polylog}\,(n)}{\sqrt{n}} \tag{90}$$

$$\sup_{\substack{x\in B_\mu(L)\\ S\in\mathcal{S}_d\left((C_{\rm slow}M_{\widetilde{L}})^{-1},C_{\rm slow}M_{\widetilde{L}}\right)}} \left\|\left|\widehat{\Psi}_\rho(x,S)\right|\right\| \le \widetilde{C}_{\tau,2}\mathrm{polylog}\,(n) \tag{91}$$

$$\inf_{x\in B_{\mu(L)}}\lambda_{\min}\left(-\widehat{\Phi}_\rho(x)\right) \ge \frac{1}{\widetilde{C}_{\tau,2}\mathrm{polylog}\,(n)} \tag{92}$$

*As a result, under $\widetilde{E}_2$ the operator $-\widehat{\Phi}_\rho$ is invertible and*

$$\sup_{x\in B_\mu(L)}\left\|\left|-\widehat{\Phi}_\rho^{-1}(x)\right|\right\| \le \widetilde{C}_{\tau,2}\mathrm{polylog}\,(n) \tag{93}$$

**Proof** See Appendix C.7. ■

**Lemma 43** *Instate the notations and assumptions in Theorem 7, Lemma 36 - 41. then under $\widetilde{E}_2$,*

$$\sup_{x\in B_\mu(L)} \left\|\widehat{Q}_\rho(x) - Q^*(x)\right\|_{\mathrm{F}} \le C_{\mathrm{fast},\tau} \frac{\mathrm{polylog}\,(n)}{\sqrt{n}}$$

*for constant $C_{\mathrm{fast},\tau}$ independent of $n$.*

**Proof** See Appendix C.8. ■

**Lemma 44** *Let $\rho = 1/n$. Instate the notations and assumptions in Theorem 7, Lemma 36 - 43. Then*

$$\mathbb{E} \sup_{x\in B_\mu(L)} \left\|\widehat{Q}_\rho(x) - Q^*(x)\right\|_{\mathrm{F}} \le C_{E,F} \frac{\mathrm{polylog}\,(n)}{\sqrt{n}}$$

*for some constant $C_{E,F}$ independent of $n$.*

**Proof** See Appendix C.9. ■

**Lemma 45** *Let $\rho = 1/n$. Instate the notations and assumptions in Theorem 7, Lemma 36 - 43. Then*

$$\mathbb{E} \sup_{x\in B_\mu(L)} W\left(\widehat{Q}_\rho(x), Q^*(x)\right) \le C_{E,W} \frac{\mathrm{polylog}\,(n)}{\sqrt{n}}$$

*for some constant $C_{E,W}$ independent of $n$.*

**Proof** See Appendix C.10. ■

### C.2 Proof of Lemma 36

Without loss of generality, assume $\mu = 0$, $\Sigma = I_p$, $C_{\psi_2} = 1$ and $c_\Lambda = 1$ (defined in Assumption 2). Denote $\widehat{\lambda}_1(x) = \lambda_{\max}\left(\widehat{Q}_\rho(x)\right)$.

By the optimality condition for $\widehat{Q}_\rho(x)$, one has

$$0 = \frac{1}{n}\sum_{i=1}^n w_{n,\rho}(x, X_i)\left(T_{\widehat{Q}_\rho(x)}^{Q_i} - I_d\right)$$

Note that $n^{-1}\sum_{i=1}^n w_{n,\rho}(x, X_i) = 1$ for any $x$, hence one has

$$I_d = \frac{1}{n}\sum_{i=1}^n w_{n,\rho}(x, X_i) T_{\widehat{Q}_\rho(x)}^{Q_i}$$

Recall that $T^Q_{\widehat{Q}_\rho(x)} = \widehat{Q}_\rho(x)^{-1/2} \left(\widehat{Q}_\rho(x)^{1/2} Q \widehat{Q}_\rho(x)^{1/2}\right)^{1/2} \widehat{Q}_\rho(x)^{-1/2}$, multiplying both sides of the equation with $\widehat{Q}_\rho(x)$ then gives

$$\widehat{Q}_\rho(x) = \frac{1}{n}\sum_{i=1}^n w_{n,\rho}(x, X_i) \left(\widehat{Q}_\rho(x)^{1/2} Q_i \widehat{Q}_\rho(x)^{1/2}\right)^{1/2}$$

Taking the largest eigenvalue on both sides, one has

$$\begin{aligned}
\widehat{\lambda}_1(x) &\leq \frac{1}{n}\sum_{i=1}^n |w_{n,\rho}(x, X_i)| \cdot \left\| \left(\widehat{Q}_\rho(x)^{1/2} Q_i \widehat{Q}_\rho(x)^{1/2}\right)^{1/2} \right\|_{\mathrm{op}} \\
&\leq \frac{1}{n}\sum_{i=1}^n |w_{n,\rho}(x, X_i)| \cdot \lambda_{\max}(Q_i)^{1/2} \widehat{\lambda}_1^{1/2}(x)
\end{aligned}$$

which implies

$$\begin{aligned}
\widehat{\lambda}_1(x) &\leq \left[\frac{1}{n}\sum_{i=1}^n \left|1 + (x - \overline{X})^\top \widehat{\Sigma}_\rho^{-1}(X_i - \overline{X})\right| \cdot \lambda_{\max}(Q_i)^{1/2}\right]^2 \\
&\leq \left[\frac{1}{n}\sum_{i=1}^n \left(1 + \|x - \overline{X}\| \cdot \left\|\widehat{\Sigma}_\rho^{-1}\right\|_{\mathrm{op}} \cdot \|X_i - \overline{X}\|\right) \lambda_{\max}(Q_i)^{1/2}\right]^2
\end{aligned}$$

Hence

$$\begin{aligned}
&\mathbb{P}\left(\sup_{x \in B_\mu(L)} \widehat{\lambda}_1(x) \geq t^2\right) \\
\leq &\mathbb{P}\left(\left\{\left\|\widehat{\Sigma}_\rho^{-1}\right\|_{\mathrm{op}} \leq 2\right\} \cap \left\{\sup_{x \in B_\mu(L)} \frac{1}{n}\sum_{i=1}^n \left(1 + \|x - \overline{X}\| \cdot \|X_i - \overline{X}\|\right) \lambda_{\max}(Q_i)^{1/2} \geq t/2\right\}\right) \\
&+ \mathbb{P}\left(\left\{2 < \left\|\widehat{\Sigma}_\rho^{-1}\right\|_{\mathrm{op}} \leq n\right\} \cap \left\{\sup_{x \in B_\mu(L)} \frac{1}{n}\sum_{i=1}^n \left(1 + \|x - \overline{X}\| \cdot \|X_i - \overline{X}\|\right) \lambda_{\max}(Q_i)^{1/2} \geq t/n\right\}\right) \\
&+ \mathbb{P}\left(\left\|\widehat{\Sigma}_\rho^{-1}\right\|_{\mathrm{op}} > n\right) \\
\leq &\underbrace{\mathbb{P}\left(\sup_{x \in B_\mu(L)} \frac{1}{n}\sum_{i=1}^n \left(1 + \|x - \overline{X}\| \cdot \|X_i - \overline{X}\|\right) \lambda_{\max}(Q_i)^{1/2} \geq t/2\right)}_{\textbf{(I)}} \\
&+ \underbrace{\mathbb{P}\left(2 < \left\|\widehat{\Sigma}_\rho^{-1}\right\|_{\mathrm{op}} \leq n\right) \wedge \mathbb{P}\left(\sup_{x \in B_\mu(L)} \frac{1}{n}\sum_{i=1}^n \left(1 + \|x - \overline{X}\| \cdot \|X_i - \overline{X}\|\right) \lambda_{\max}(Q_i)^{1/2} \geq t/n\right)}_{\textbf{(II)}} \\
&+ \underbrace{\mathbb{P}\left(\left\|\widehat{\Sigma}_\rho^{-1}\right\|_{\mathrm{op}} > n\right)}_{\textbf{(III)}}
\end{aligned}$$

**Claim 1** *Suppose Assumption 1-2 hold with $\mu = 0$, $\Sigma = I_p$, $C_{\psi_2} = 1$ and $c_\Lambda = 1$. If $n \geq 3$, $1 \leq L \leq \sqrt{n}$. then for $\rho \in \{0, n^{-1}\}$ and any $t \geq 6L$,*

$$\mathbb{P}\left\{\left\|\widehat{\Sigma}_\rho^{-1}\right\|_{\text{op}} > 2\right\} \lesssim \exp(-c_e n)$$
$$\mathbb{P}\left\{\sup_{x \in B_\mu(L)} \frac{1}{n}\sum_{i=1}^n \left(1 + \|x - \overline{X}\| \cdot \|X_i - \overline{X}\|\right) \lambda_{\max}(Q_i)^{1/2} \geq t\right\} \lesssim h_0(t)$$

*where*

$$h_0(t) = \exp\left(-\sqrt{\frac{t}{c_b L}}\right) + n \cdot \exp\left[-\left(\frac{t}{c_b L}\right)^{2/(C_\Lambda+2)}\right]$$

*for some constants $c_b, c_e > 0$ independent of $n$.*

See Appendix C.2.1 for the proof.

Combining the above results, one can obtain that

- If $\rho = 0$, then

$$(\textbf{II}) + (\textbf{III}) \leq \mathbb{P}\left(\left\|\widehat{\Sigma}_\rho^{-1}\right\|_{\text{op}} > 2\right)$$

  As a result, for any $t \geq 6L$, the tail probability satisfies

$$\mathbb{P}\left(\sup_{x \in B_\mu(L)} \widehat{\lambda}_1(x) \geq t^2\right) \lesssim h_0(t) + \exp(-c_e n)$$

- If $\rho = n^{-1}$, then $(\textbf{III}) = 0$. As a results, the tail probability satisfies

$$\mathbb{P}\left(\sup_{x \in B_\mu(L)} \widehat{\lambda}_1(x) \geq t^2\right) \lesssim \begin{cases} h_0(t) + \exp(-c_e n), & t \in [t_0, nt_0] \\ h_0(t) + \exp(-c_e n) \wedge h_0(t/n), & t \in (nt_0, +\infty) \end{cases}$$

  with $t_0 = 6L$.

Finally, set

$$\tilde{t}_L = (C_b \vee c_b)\, L_\tau^{2+(2\vee 2C_1)} L$$

Note that $\tilde{t}_L \geq t_0$, hence

$$\begin{aligned}\mathbb{P}\left(\sup_{x \in B_\mu(L)} \widehat{\lambda}_1(x) \geq \tilde{t}_L^2\right) &\lesssim h_0(\tilde{t}_L) + \exp(-c_e n) \\ &\lesssim n^{-(1+\tau)} + \exp(-c_e n) \\ &\leq C_\tau n^{-(1+\tau)}\end{aligned}$$

for some constant $C_\tau$ independent of $n$ (but depends on $\tau$).

This finishes the proof of Lemma 36.

#### C.2.1 Proof of Claim 1

The first inequality follows by noticing that $\widehat{\Sigma}_\rho = n^{-1}\sum_{i=1}^n X_iX_i^\top - \overline{X}\,\overline{X}^\top + \rho I_p$ with $\rho \in \{0, 1/n\}$. Applying the triangle inequality, one can obtain that for $n \geq 3$,

$$\begin{aligned}
\mathbb{P}\left\{\left\|\widehat{\Sigma}_\rho^{-1}\right\|_{\mathrm{op}} > 2\right\} &\leq \mathbb{P}\left\{\left\|\widehat{\Sigma}_\rho - I_p\right\|_{\mathrm{op}} > 1/2\right\} \\
&\leq \mathbb{P}\left(\left\|n^{-1}\sum_{i=1}^n X_iX_i^\top - I_p\right\|_{\mathrm{op}} > 1/12\right) + \mathbb{P}\left(\left\|\overline{X}\,\overline{X}^\top\right\|_{\mathrm{op}} > 1/12\right)
\end{aligned}$$

Note that the last line holds for both $\rho \in \left\{0, n^{-1}\right\}$. Under the sub-Gaussian assumption in Assumption 1, one can obtain that

$$\begin{aligned}
&\mathbb{P}\left\{\left\|n^{-1}\sum_{i=1}^n X_iX_i^\top - I_p\right\|_{\mathrm{op}} > 1/12\right\} \lesssim \exp(-cn) \\
&\mathbb{P}\left\{\left\|\overline{X}\,\overline{X}^\top\right\|_{\mathrm{op}} > 1/12\right\} \lesssim \exp(-cn)
\end{aligned}$$

Now we move on to the second inequality. Note that

$$\begin{aligned}
&\frac{1}{n}\sum_{i=1}^n \left(1 + \left\|x - \overline{X}\right\| \cdot \left\|X_i - \overline{X}\right\|\right)\lambda_{\max}(Q_i)^{1/2} \\
\leq &\frac{1}{n}\sum_{i=1}^n \left(1 + \|x\| \cdot \|X_i\|\right)\lambda_{\max}(Q_i)^{1/2} + \frac{1}{n}\sum_{i=1}^n \left\|\overline{X}\right\| \cdot \|X_i\|\,\lambda_{\max}(Q_i)^{1/2} \\
+ &\frac{1}{n}\sum_{i=1}^n \|x\| \cdot \left\|\overline{X}\right\|\lambda_{\max}(Q_i)^{1/2} + \frac{1}{n}\sum_{i=1}^n \left\|\overline{X}\right\|^2\lambda_{\max}(Q_i)^{1/2} \\
= &\left(1 + \|x\|\left\|\overline{X}\right\| + \left\|\overline{X}\right\|^2\right) \cdot \frac{1}{n}\sum_{i=1}^n \lambda_{\max}(Q_i)^{1/2} + \left(\left\|\overline{X}\right\| + \|x\|\right) \cdot \frac{1}{n}\sum_{i=1}^n \|X_i\|\,\lambda_{\max}(Q_i)^{1/2}
\end{aligned}$$

Therefore, for any $L \geq 1$ and $t > 0$, one has

$$\begin{aligned}
&\mathbb{P}\left\{\sup_{x\in B_\mu(L)} \frac{1}{n}\sum_{i=1}^n \left(1 + \left\|x - \overline{X}\right\| \cdot \left\|X_i - \overline{X}\right\|\right)\lambda_{\max}(Q_i)^{1/2} \geq t\right\} \\
\leq &\mathbb{P}\left\{\left(1 + L\left\|\overline{X}\right\| + \left\|\overline{X}\right\|^2\right) \cdot \frac{1}{n}\sum_{i=1}^n \lambda_{\max}(Q_i)^{1/2} \geq \frac{1}{1+L}t\right\} \\
+ &\mathbb{P}\left\{\left(\left\|\overline{X}\right\| + \|x\|\right) \cdot \frac{1}{n}\sum_{i=1}^n \|X_i\|\,\lambda_{\max}(Q_i)^{1/2} \geq \frac{L}{1+L}t\right\} \\
\leq &\underbrace{\mathbb{P}\left\{\left(1 + L\left\|\overline{X}\right\| + \left\|\overline{X}\right\|^2\right) \cdot \frac{1}{n}\sum_{i=1}^n \lambda_{\max}(Q_i)^{1/2} \geq \frac{t}{2L}\right\}}_{(\mathbf{A})}
\end{aligned}$$

$$+\underbrace{\mathbb{P}\left\{\left(\left\|\overline{X}\right\|+L\right)\cdot\frac{1}{n}\sum_{i=1}^{n}\left\|X_i\right\|\lambda_{\max}(Q_i)^{1/2}\geq\frac{t}{2}\right\}}_{(\mathbf{B})}$$

**Analysis for (A):** for any $1 \leq L \leq \sqrt{n}$, one can obtain that

$$\begin{aligned}
(\mathbf{A}) &\leq \mathbb{P}\left\{\frac{1}{n}\sum_{i=1}^{n}\lambda_{\max}(Q_i)^{1/2}\geq\frac{1}{1+L/\sqrt{n}+1/n}\cdot\frac{t}{2L}\right\} \\
&\quad+\mathbb{P}\left\{L\left\|\overline{X}\right\|\frac{1}{n}\sum_{i=1}^{n}\lambda_{\max}(Q_i)^{1/2}\geq\frac{L/\sqrt{n}}{1+L/\sqrt{n}+1/n}\cdot\frac{t}{2L}\right\} \\
&\quad+\mathbb{P}\left\{\left\|\overline{X}\right\|^2\frac{1}{n}\sum_{i=1}^{n}\lambda_{\max}(Q_i)^{1/2}\geq\frac{1/n}{1+L/\sqrt{n}+1/n}\cdot\frac{t}{2L}\right\} \\
&\leq \mathbb{P}\left\{\frac{1}{n}\sum_{i=1}^{n}\lambda_{\max}(Q_i)^{1/2}\geq\frac{1}{3}\cdot\frac{t}{2L}\right\}+\underbrace{\mathbb{P}\left\{\left\|\overline{X}\right\|\frac{1}{n}\sum_{i=1}^{n}\lambda_{\max}(Q_i)^{1/2}\geq\frac{1/\sqrt{n}}{3}\cdot\frac{t}{2L}\right\}}_{\mathbf{A_1}} \\
&\quad+\underbrace{\mathbb{P}\left\{\left\|\overline{X}\right\|^2\frac{1}{n}\sum_{i=1}^{n}\lambda_{\max}(Q_i)^{1/2}\geq\frac{1/n}{3}\cdot\frac{t}{2L}\right\}}_{\mathbf{A_2}}
\end{aligned}$$

- $\mathbf{A_1}$:

$$\mathbf{A_1}\leq\mathbb{P}\left\{\left\|\overline{X}\right\|\geq\frac{1}{\sqrt{n}}\cdot\sqrt{\frac{t}{6L}}\right\}+\mathbb{P}\left\{\frac{1}{n}\sum_{i=1}^{n}\lambda_{\max}(Q_i)^{1/2}\geq\sqrt{\frac{t}{6L}}\right\}$$

- $\mathbf{A_2}$:

$$\mathbf{A_2}\leq\mathbb{P}\left\{\left\|\overline{X}\right\|^2\geq\frac{1}{n}\cdot\sqrt{\frac{t}{6L}}\right\}+\mathbb{P}\left\{\frac{1}{n}\sum_{i=1}^{n}\lambda_{\max}(Q_i)^{1/2}\geq\sqrt{\frac{t}{6L}}\right\}$$

Hence for $t \geq 6L$,

$$\begin{aligned}
(\mathbf{A}) &\lesssim \mathbb{P}\left\{\frac{1}{n}\sum_{i=1}^{n}\lambda_{\max}(Q_i)^{1/2}\geq\sqrt{\frac{t}{6L}}\right\}+\mathbb{P}\left\{\left\|\overline{X}\right\|^2\geq\frac{1}{n}\cdot\sqrt{\frac{t}{6L}}\right\} \\
&\lesssim \mathbb{P}\left\{\frac{1}{n}\sum_{i=1}^{n}\lambda_{\max}(Q_i)^{1/2}\geq\sqrt{\frac{t}{6L}}\right\}+\exp\left(-c\sqrt{\frac{t}{6L}}\right)
\end{aligned}$$

**Analysis for (B):**

$$(\mathbf{B})\leq\mathbb{P}\left\{L\cdot\frac{1}{n}\sum_{i=1}^{n}\left\|X_i\right\|\lambda_{\max}(Q_i)^{1/2}\geq\frac{\sqrt{n}L}{\sqrt{n}L+1}\frac{t}{2}\right\}$$

$$
\begin{aligned}
&+ \mathbb{P}\left\{\left\|\overline{X}\right\| \cdot \frac{1}{n}\sum_{i=1}^n \|X_i\|\,\lambda_{\max}(Q_i)^{1/2} \geq \frac{1}{\sqrt{n}L+1}\frac{t}{2}\right\} \\
&\leq \mathbb{P}\left\{\frac{1}{n}\sum_{i=1}^n \|X_i\|\,\lambda_{\max}(Q_i)^{1/2} \geq \frac{t}{4L}\right\} + \mathbb{P}\left\{\left\|\overline{X}\right\| \cdot \frac{1}{n}\sum_{i=1}^n \|X_i\|\,\lambda_{\max}(Q_i)^{1/2} \geq \frac{t}{4\sqrt{n}L}\right\} \\
&\leq \mathbb{P}\left\{\frac{1}{n}\sum_{i=1}^n \|X_i\|\,\lambda_{\max}(Q_i)^{1/2} \geq \frac{t}{4L}\right\} \\
&+ \mathbb{P}\left\{\left\|\overline{X}\right\| \geq \sqrt{\frac{t}{4nL}}\right\} + \mathbb{P}\left\{\frac{1}{n}\sum_{i=1}^n \|X_i\|\,\lambda_{\max}(Q_i)^{1/2} \geq \sqrt{\frac{t}{4L}}\right\}
\end{aligned}
$$

Hence for $t \geq 4L$,

$$
\begin{aligned}
(\mathbf{B}) &\lesssim \mathbb{P}\left\{\left\|\overline{X}\right\| \geq \sqrt{\frac{t}{4nL}}\right\} + \mathbb{P}\left\{\frac{1}{n}\sum_{i=1}^n \|X_i\|\,\lambda_{\max}(Q_i)^{1/2} \geq \sqrt{\frac{t}{4L}}\right\} \\
&\lesssim \exp(-ct/L) + \mathbb{P}\left\{\frac{1}{n}\sum_{i=1}^n \|X_i\|\,\lambda_{\max}(Q_i)^{1/2} \geq \sqrt{\frac{t}{4L}}\right\}
\end{aligned}
$$

Combining results for $(\mathbf{A})$ and $(\mathbf{B})$, one can obtain that for $t \geq 6L$,

$$
\begin{aligned}
&(\mathbf{A}) + (\mathbf{B}) \\
&\lesssim \exp\left(-c\sqrt{\frac{t}{6L}}\right) + \exp(-ct/L) + \mathbb{P}\left\{\frac{1}{n}\sum_{i=1}^n \lambda_{\max}(Q_i)^{1/2} \geq \sqrt{\frac{t}{6L}}\right\} \\
&+ \mathbb{P}\left\{\frac{1}{n}\sum_{i=1}^n \|X_i\|\,\lambda_{\max}(Q_i)^{1/2} \geq \sqrt{\frac{t}{4L}}\right\} \\
&\lesssim \exp\left(-c\sqrt{\frac{t}{6L}}\right) + \mathbb{P}\left\{\frac{1}{n}\sum_{i=1}^n \lambda_{\max}^{1/2}(Q_i) \geq \sqrt{\frac{t}{6L}}\right\} + \mathbb{P}\left\{\frac{1}{n}\sum_{i=1}^n \|X_i\|\,\lambda_{\max}^{1/2}(Q_i) \geq \sqrt{\frac{t}{4L}}\right\}
\end{aligned}
$$

Note that for any $s \geq 1$,

$$
\begin{aligned}
\mathbb{P}\left\{\frac{1}{n}\sum_{i=1}^n \lambda_{\max}(Q_i)^{1/2} \geq s\right\} &\leq n \cdot \mathbb{P}\left\{\lambda_{\max}(Q_i)^{1/2} \geq s\right\} \\
&\leq n \cdot \mathbb{P}\left\{(1 \vee \|X_i\|)^{C_\Lambda/2} \geq s\right\} \\
&= n \cdot \mathbb{P}\left\{\|X_i\| \geq s^{2/C_\Lambda}\right\} \\
&\lesssim n \cdot \exp\left(-cs^{4/C_\Lambda}\right)
\end{aligned}
$$

and

$$
\mathbb{P}\left\{\frac{1}{n}\sum_{i=1}^n \|X_i\|\,\lambda_{\max}(Q_i)^{1/2} \geq s\right\} \leq n \cdot \mathbb{P}\left\{\|X_i\|\,\lambda_{\max}(Q_i)^{1/2} \geq s\right\}
$$

$$
\begin{aligned}
&\leq n \cdot \mathbb{P}\left\{\|X_i\| \left(1 \vee \|X_i\|\right)^{C_\Lambda/2} \geq s\right\} \\
&= n \cdot \mathbb{P}\left\{\|X_i\|^{1+C_\Lambda/2} \geq s\right\} \\
&\lesssim n \cdot \exp\left(-cs^{4/(C_\Lambda+2)}\right)
\end{aligned}
$$

Therefore, for any $t \geq 6L$,

$$
\begin{aligned}
(\mathbf{A}) + (\mathbf{B}) &\lesssim \exp\left(-c\sqrt{\frac{t}{L}}\right) + n \cdot \exp\left[-c\left(t/L\right)^{2/C_\Lambda}\right] + n \cdot \exp\left[-c\left(t/L\right)^{2/(C_\Lambda+2)}\right] \\
&\lesssim \exp\left(-c\sqrt{\frac{t}{L}}\right) + n \cdot \exp\left[-c\left(t/L\right)^{2/(C_\Lambda+2)}\right]
\end{aligned}
$$

The proof of Claim 1 is complete by adjusting constant $c$ here.

### C.3 Proof of Lemma 37

Without loss of generality, assume $\mu = 0$, $\Sigma = I_p$, $C_{\psi_2} = 1$. Recall notation $\mathcal{S}_d(a,b) := \{A \in \mathcal{S}_d : aI_d \prec A \prec bI_d\}$. Denote parameter $\theta = (x, S)$ and parameter space $\Theta = B_\mu(L) \times \mathcal{S}_d(0, \widetilde{M}_L)$. Let $F(\theta)$ denote $F(x,S)$ and similarly for other functions of $(x,S)$. By triangle inequality, one can obtain that

$$
\sup_{\theta \in \Theta}\left|\widehat{F}_\rho(\theta) - F(\theta)\right| \leq \underbrace{\sup_{\theta \in \Theta}\left|\widehat{F}_\rho(\theta) - \widetilde{F}_n(\theta)\right|}_{\zeta_1} + \underbrace{\sup_{\theta \in \Theta}\left|\widetilde{F}_n(\theta) - F(\theta)\right|}_{\zeta_2} \tag{94}
$$

For any $t_1, t_2 > 0$, define $C_{\sup}(t_1,t_2) := \sup\left\{W^2(Q,S) : Q \in \mathcal{S}_d(0,t_1), S \in \mathcal{S}_d(0,t_2)\right\}$. By Lemma 21, one has

$$
C_{\sup}(t_1,t_2) \lesssim t_1 + t_2 \tag{95}
$$

Here dimension $d$ is viewed as a fixed constant and absorbed into $\lesssim$.

- $\zeta_1$: under event $E_0$ (defined in (78)), one has
  - $\|X_i - \mu\| \leq L_\tau = \sqrt{(1+\tau)\log n}$ for $i = 1, ..., n$.
  - $W^2(Q_i, S) \lesssim M_{L_\tau} \vee \widetilde{M}_L$ for any $S \in \mathcal{S}_d(0, \widetilde{M}_L)$ and $i \in [n]$.

  Lemma 28 then implies that

  $$
  \zeta_1 \lesssim \left(M_{L_\tau} \vee \widetilde{M}_L\right)\frac{LL_\tau^3}{\sqrt{n}} \tag{96}
  $$

  with probability greater than $1 - O\left(n^{-(1+\tau)}\right)$.

- $\zeta_2$ can be bounded by truncation and the uniform concentration in Lemma 29. Let $\widetilde{L} \geq 1$ be a parameter to be specified later, and let $M_{\widetilde{L}} := \gamma_\Lambda(\widetilde{L})$. Then Assumption 2 implies that a.s.

  $$
  X \in B_\mu(\widetilde{L}) \implies Q \in \mathcal{S}_d\left(M_{\widetilde{L}}^{-1}, M_{\widetilde{L}}\right) \tag{97}
  $$

Let $\widetilde{X}$ be a truncated form of $X$ defined as

$$\widetilde{X} := \widetilde{X}(\widetilde{L}) = X\mathbf{1}(\|X\| \leq \widetilde{L}) \tag{98}$$

Let $\widetilde{X}_> := X - \widetilde{X}$. By definition, one has $X = \widetilde{X} + \widetilde{X}_>$ , $\left\|\widetilde{X}\right\| \leq \widetilde{L}$ and

$$\widetilde{X}_> = \begin{cases} 0, & \|X\| \leq \widetilde{L} \\ X, & \|X\| > \widetilde{L} \end{cases} \tag{99}$$

As a result, one can obtain the following decomposition.

$$\begin{aligned}
& w(x,X)W^2(S,Q) \\
= & \left(1 + x^\top X\right) W^2(S,Q) \\
= & \left(1 + x^\top \widetilde{X}\right) W^2(S,Q) + x^\top \widetilde{X}_> W^2(S,Q) \\
= & \left(1 + x^\top \widetilde{X}\right) W^2(S,Q) \mathbf{1}\left(Q \in \mathcal{S}\left(M_{\widetilde{L}}^{-1}, M_{\widetilde{L}}\right)\right) \\
& + \left(1 + x^\top \widetilde{X}\right) W^2(S,Q) \mathbf{1}\left(Q \notin \mathcal{S}\left(M_{\widetilde{L}}^{-1}, M_{\widetilde{L}}\right)\right) + x^\top \widetilde{X}_> W^2(S,Q) \\
\overset{(i)}{=} & \left(1 + x^\top \widetilde{X}\right) W^2(S,Q) \mathbf{1}\left(Q \in \mathcal{S}\left(M_{\widetilde{L}}^{-1}, M_{\widetilde{L}}\right)\right) \\
& + W^2(S,Q) \mathbf{1}\left(Q \notin \mathcal{S}\left(M_{\widetilde{L}}^{-1}, M_{\widetilde{L}}\right)\right) + x^\top \widetilde{X}_> W^2(S,Q)
\end{aligned}$$

Here (i) follows from (97). Indeed, if $Q \notin \mathcal{S}\left(M_{\widetilde{L}}^{-1}, M_{\widetilde{L}}\right)$, then as a result of (97), one has $\|X\| > \widetilde{L}$ which then implies $\widetilde{X} = 0$.

As a consequence, one can then obtain following decomposition of $\widetilde{F}_n(\theta) - F(\theta)$.

$$\begin{aligned}
\widetilde{F}_n(\theta) - F(\theta) = & \alpha_1(\theta) + \alpha_2(\theta) + \alpha_3(\theta), \quad \text{where} \\
\alpha_1(\theta) := & \frac{1}{n} \sum_{i=1}^n \left(1 + x^\top \widetilde{X}_i\right) W^2(S,Q_i) \mathbf{1}\left(Q_i \in \mathcal{S}\left(M_{\widetilde{L}}^{-1}, M_{\widetilde{L}}\right)\right) \\
& - \mathbb{E}\left(1 + x^\top \widetilde{X}\right) W^2(S,Q) \mathbf{1}\left(Q \in \mathcal{S}\left(M_{\widetilde{L}}^{-1}, M_{\widetilde{L}}\right)\right) \\
\alpha_2(\theta) := & \frac{1}{n} \sum_{i=1}^n x^\top \widetilde{X}_{i,>} W^2(S,Q_i) - \mathbb{E} x^\top \widetilde{X}_> W^2(S,Q) \\
\alpha_3(\theta) := & \frac{1}{n} \sum_{i=1}^n W^2(S,Q_i) \mathbf{1}\left(Q_i \notin \mathcal{S}\left(M_{\widetilde{L}}^{-1}, M_{\widetilde{L}}\right)\right) \\
& - \mathbb{E} W^2(S,Q) \mathbf{1}\left(Q \notin \mathcal{S}\left(M_{\widetilde{L}}^{-1}, M_{\widetilde{L}}\right)\right)
\end{aligned}$$

Then triangle inequality gives

$$\zeta_2 \leq \sup_{\theta \in \Theta} |\alpha_1(\theta)| + \sup_{\theta \in \Theta} |\alpha_2(\theta)| + \sup_{\theta \in \Theta} |\alpha_3(\theta)| \tag{100}$$

– $\alpha_2(\theta), \alpha_3(\theta)$: $\sup |\alpha_2(\theta)|$ and $\sup |\alpha_3(\theta)|$ can be upper bounded as in Claim 2. The proof is deferred to Appendix C.3.1.

**Claim 2** *There exists constant $\widetilde{C}$ independent of $n$ such that by taking $\widetilde{L} = \widetilde{C}C_{\psi_2}\sqrt{(1+\tau)\log n}$, the following inequality*

$$\sup_{\theta\in\Theta} |\alpha_2(\theta)| \vee \sup_{\theta\in\Theta} |\alpha_3(\theta)| = O\left(\frac{\widetilde{M_L}\widetilde{L} + \widetilde{L}^{1+C_\Lambda}}{n^{1+\tau}} \cdot L\right)$$

*holds with probability greater than $1 - O\left(n^{-\tau}\right)$.*

– $\alpha_1(\theta)$: Apply triangle inequality to see that

$$\sup_{\theta\in\Theta} |\alpha_1(\theta)| = \underbrace{\sup_{\theta\in\Theta} |\alpha_1(\theta)| - \mathbb{E}\sup_{\theta\in\Theta} |\alpha_1(\theta)|}_{b_1} + \underbrace{\mathbb{E}\sup_{\theta\in\Theta} |\alpha_1(\theta)|}_{b_2} \tag{101}$$

After truncation, $\alpha_1(\theta)$ is uniformly bounded. Hence $b_1$ can be upper bounded by exploiting the bounded difference property (Lemma 31). For $b_2$, we first show that $W^2(Q,S)$ is only Hölder continuous in $S$ which then implies Hölder (rather than Lipschitz) continuity of the corresponding sub-Gaussian norm. By generalizing Dudley's integral inequality via chaining in Lemma 29, we arrive at an upper bound for $b_2$. The results are summarized below in in Claim 3 whose proof is deferred to Appendix C.3.2.

**Claim 3** *Instate the notations and assumptions in Lemma 38 and Claim 2.*

$$\sup_{\theta\in\Theta} |\alpha_1(\theta)| = O\left(\frac{L\widetilde{M}_L^2}{\sqrt{n}}\sqrt{\log \widetilde{M}_L}\right)$$

*with probability at least $1 - O\left(n^{-(1+\tau)}\right)$.*

– combine Claim 2, Claim 3 and (100) to see that

$$\zeta_2 = O\left(\frac{L\widetilde{M}_L^3}{\sqrt{n}}\right) \tag{102}$$

with probability at least $1 - O\left(n^{-\tau}\right)$.

- Combining results for $\zeta_1$ and $\zeta_2$, one has

$$\begin{aligned}\sup_{\theta\in\Theta}\left|\widehat{F}_\rho(\theta) - F(\theta)\right| &\le \zeta_1 + \zeta_2 \\ &= O\left(\frac{L\widetilde{M}_L^3}{\sqrt{n}}\right)\end{aligned}$$

with probability greater than $1 - O\left(n^{-\tau}\right)$.

The proof of Lemma 37 is now complete.

### C.3.1 Proof of Claim 2

Without loss of generality, assume $\|X\|_{\psi_2} \le \|\|X\|\|_{\psi_2} \le 1$.
**Proof for** $\alpha_2(\theta)$: a crude upper bound suffices here. By triangle inequality, one has

$$\begin{aligned}
&\sup_{\theta\in\Theta} |\alpha_2(\theta)| \\
\le& \sup_{\theta\in\Theta} \left|\mathbb{E}x^\top \widetilde{X}_> W^2(S,Q)\right| + \sup_{\theta\in\Theta} \left|\frac{1}{n}\sum_{i=1}^n x^\top \widetilde{X}_{i,>} W^2(S,Q_i)\right| \\
\le& \sup_{\theta\in\Theta} \|x\| \cdot \mathbb{E}\left\|\widetilde{X}_>\right\| \cdot W^2(S,Q) + \sup_{\theta\in\Theta} \frac{1}{n}\sum_{i=1}^n \|x\| \cdot \left\|\widetilde{X}_{i,>}\right\| \cdot W^2(S,Q_i)
\end{aligned}$$

Therefore, for any $t > 0$, one has

$$\begin{aligned}
\mathbb{P}\left\{\sup_{\theta\in\Theta} |\alpha_2(\theta)| \ge t\right\} \le& \underbrace{\mathbb{P}\left\{\sup_{\theta\in\Theta} \|x\| \cdot \mathbb{E}\left\|\widetilde{X}_>\right\| \cdot W^2(S,Q) \ge t\right\}}_{=:\mathbf{I}(t)} \\
&+ \underbrace{\mathbb{P}\left\{\sup_{\theta\in\Theta} \frac{1}{n}\sum_{i=1}^n \|x\| \cdot \left\|\widetilde{X}_{i,>}\right\| \cdot W^2(S,Q_i) > 0\right\}}_{=:\mathbf{II}(t)}
\end{aligned}$$

**I(t):** Lemma 21 and Assumption 2 imply that

$$\sup_{\theta\in\Theta} W^2(S,Q) \lesssim \widetilde{M}_L + \gamma_\Lambda(\|X\|)$$

which further gives

$$\begin{aligned}
(\mathbf{I}) \le& \mathbb{P}\left\{\mathbb{E}\left\|\widetilde{X}_>\right\| \left(\widetilde{M}_L + \|X\|^{C_\Lambda}\right) \ge t/(Lc)\right\} \\
\le& \mathbb{P}\left\{\mathbb{E}\left\|\widetilde{X}_>\right\| \ge \frac{t}{2cL\widetilde{M}_L}\right\} + \mathbb{P}\left\{\mathbb{E}\left\|\widetilde{X}_>\right\| \|X\|^{C_\Lambda} \ge t/(2Lc)\right\}
\end{aligned}$$

By the definition of $\widetilde{X}_>$, one can obtain that $\left\|\widetilde{X}_>\right\| \|X\|^{C_\Lambda} = \left\|\widetilde{X}_>\right\|^{1+C_\Lambda}$ and for any $s \ge 0$,

$$\mathbb{P}\left\{\left\|\widetilde{X}_>\right\| \ge \widetilde{L} + s\right\} \le 2\exp\left(-\widetilde{L}^2\right)\exp\left(-s^2\right)$$

Therefore, one can obtain that for any $\widetilde{L} \ge 1$, one has

$$\begin{aligned}
\mathbb{E}\left\|\widetilde{X}_>\right\| \le& 2\exp(-\widetilde{L}^2)\int_0^\infty (\widetilde{L}+s)\exp(-s^2) \\
\lesssim& \exp(-\widetilde{L}^2)\widetilde{L}
\end{aligned}$$

and

$$\mathbb{E}\left\|\widetilde{X}_>\right\|^{1+C_\Lambda} \le 2\exp(-\widetilde{L}^2)\int_0^\infty (\widetilde{L}+s)^{1+C_\Lambda}\exp(-s^2)ds$$

$$\lesssim \exp(-\widetilde{L}^2)\widetilde{L}^{1+C_\Lambda}$$

Therefore, by taking $\widetilde{L} = O(L_\tau)$, there exists constant $C_I > 0$ such that

$$\mathbf{I}(\mathbf{t}) = 0, \quad \forall t \geq C_I n^{-(1+\tau)} \cdot \left(\widetilde{M}_L + M_{\widetilde{L}}\right) L\widetilde{L}$$

**II:** Note that $\left\|\widetilde{X}_{i,>}\right\| > 0$ if and only only if $\|X_i\| > \widetilde{L}$. Hence

$$\begin{aligned}(\mathbf{II}) &\leq n \cdot \mathbb{P}\left\{\left\|\|X_i\| > \widetilde{L}\right\|\right\} \\ &\lesssim n \cdot \exp(-\widetilde{L}^2) \\ &\lesssim n^{-\tau}\end{aligned}$$

**Combining (I), (II),** there exists a constant $\widetilde{C}_2 > 0$ such that for any $s \geq 0$,

$$\mathbb{P}\left\{\sup_{\theta\in\Theta} |\alpha_2(\theta)| \geq n^{-(1+\tau)}\widetilde{C}_2 L\widetilde{L}\left(\widetilde{M}_L + \widetilde{L}^{C_\Lambda}\right)\right\} \lesssim n^{-\tau}$$

**Proof for** $\alpha_3(\theta)$**:**

$$\begin{aligned}\alpha_3(\theta) := \frac{1}{n}\sum_{i=1}^n W^2(S, Q_i)\mathbf{1}\left(Q_i \notin \mathcal{S}\left(M_{\widetilde{L}}^{-1}, M_{\widetilde{L}}\right)\right) \\ - \mathbb{E}W^2(S, Q)\mathbf{1}\left(Q \notin \mathcal{S}\left(M_{\widetilde{L}}^{-1}, M_{\widetilde{L}}\right)\right)\end{aligned}$$

Following a similar argument to the one above, there exists constant $\widetilde{C}_3 > 0$ such that

$$\mathbb{P}\left\{\sup_{\theta\in\Theta} |\alpha_3(\theta)| \geq n^{-(1+\tau)}\widetilde{C}_3 L(\widetilde{M}_L + \widetilde{L}^{C_\Lambda})\right\} \lesssim n^{-\tau}$$

Finally, combining above results on $\alpha_2(\theta)$ and $\alpha_3(\theta)$ proves Claim 2.

### C.3.2 Proof of Claim 3

Recall that we assume without loss of generality that $C_{\psi_2} = 1$, $\mu = 0$, $\Sigma = I_p$, $\widetilde{L} = L_\tau$, $M_{\widetilde{L}} = \gamma_\Lambda(\widetilde{L})$ and $\widetilde{M}_L$ is defined in Lemma 36 as

$$\widetilde{M}_L \asymp L^2 L_\tau^{4+(4\vee 2C_1)} \gtrsim c_\Lambda(1 \vee \widetilde{L})^{C_\Lambda} = M_{\widetilde{L}}$$

Denote $Z = (X, Q)$ and define $\widetilde{X}$ as in (98). Define

$$f(Z;\theta) := \left(1 + (x-\mu)^\top \Sigma^{-1}(\widetilde{X} - \mu)\right) W^2(S, Q)\mathbb{1}\left(Q \in \mathcal{S}\left(M_{\widetilde{L}}^{-1}, M_{\widetilde{L}}\right)\right)$$

Then one has $\alpha_1(\theta) = n^{-1}\sum_{i=1}^n f(Z_i;\theta) - \mathbb{E}f(Z;\theta)$.

**Analysis of $b_1$:** due to truncation, $f$ is uniformly bounded. To see this, note that recall the definition of $\Theta$,

$$\theta \in \Theta \iff x \in B_\mu(L), S \in \mathcal{S}_d(0, \widetilde{M}_L)$$

Then for any $\theta \in \Theta$, one has

$$\begin{aligned}
\sup_{\theta\in\Theta}\sup_{Z} |f(Z;\theta)| &= \sup_{\theta\in\Theta}\sup_{Z} \left|(1 + x^\top \widetilde{X})W^2(Q,S)\right| \mathbb{1}\left(Q \in \mathcal{S}_d\left(M_{\widetilde{L}}^{-1}, M_{\widetilde{L}}\right)\right) \\
&\le \sup_{\theta\in\Theta}\sup_{Z} \left(1 + \|x-\mu\| \cdot \left\|\widetilde{X} - \mu\right\|\right) \cdot \left|W^2(Q,S)\right| \mathbb{1}\left(Q \in \mathcal{S}_d\left(M_{\widetilde{L}}^{-1}, M_{\widetilde{L}}\right)\right) \\
&\lesssim \left(1 + L\widetilde{L}\right) \cdot (M_{\widetilde{L}} + \widetilde{M}_L) \\
&\lesssim L\widetilde{L}\widetilde{M}_L
\end{aligned} \tag{103}$$

By Lemma 31, one has $\|b_1(x)\|_{\psi_2} \lesssim \frac{L\widetilde{L}\widetilde{M}_L}{\sqrt{n}}$ which implies

$$b_1 = O\left(\frac{L\widetilde{L}\widetilde{M}_L L_\tau}{\sqrt{n}}\right) \tag{104}$$

with probability at least $1 - O\left(n^{-(1+\tau)}\right)$ for some absolute constant $C > 0$.

**Analysis of $b_2$:** to apply Lemma 29, we follow the steps below.

- first we define a metric $d$ on $\Theta$ by

$$d(\theta, \widetilde{\theta}) = \max\left\{\|x - \widetilde{x}\|_2, \left\|S - \widetilde{S}\right\|_{\mathrm{F}}\right\}$$

  for any $\theta = (x, S)$ and $\widetilde{S} = (\widetilde{x}, \widetilde{S})$. Since $\mathcal{S}_d(0, \widetilde{M}_L)$ is a subset of $\left\{S \in \mathbb{R}^{d\times d} : |S_{ij}| \le \widetilde{M}_L\right\}$ (by noticing that for any $S \in \mathcal{S}^+_{\widetilde{M}_L}$, one has $0 \le S_{ii} \le \widetilde{M}_L$ and $|S_{ij}| \le \sqrt{S_{ii}S_{jj}} \le \widetilde{M}_L$), the diameter $D$ and metric entropy of $\Theta$ can be upper bounded by

$$D \le \max\left\{\mathrm{diam}(B_\mu(\widetilde{L})), \mathrm{diam}(\mathcal{S}_d(\widetilde{M}_L^{-1}, \widetilde{M}_L))\right\} \lesssim L \vee \widetilde{M}_L \overset{(i)}{\lesssim} \widetilde{M}_L \tag{105}$$

  and

$$\begin{aligned}
\log N(\epsilon; \Theta) &\le \log\left(N(\epsilon; B_\mu(\widetilde{L})) \cdot N(\epsilon; \mathcal{S}^+_{\widetilde{M}_L})\right) \\
&\lesssim \log^+\left(\frac{L \vee \widetilde{M}_L}{\epsilon}\right) \\
&\overset{(ii)}{\lesssim} \log^+\left(\frac{\widetilde{M}_L}{\epsilon}\right)
\end{aligned} \tag{106}$$

  Here both (i) and (ii) arise due to $\widetilde{M}_L \gtrsim L$.

- Next, let us consider the sub-Gaussian norm of $\alpha_1(\theta) - \alpha_1(\widetilde{\theta})$ as (40) in Lemma 29. By (43) in Lemma 29, one has

$$\begin{aligned}
\left\|\alpha_1(\theta) - \alpha_1(\widetilde{\theta})\right\|_{\psi_2} &\lesssim \frac{1}{\sqrt{n}} \left\|\left\|f(Z;\theta) - f(Z;\widetilde{\theta})\right\|\right\|_{\psi_2} \\
&= \frac{1}{\sqrt{n}} \left\|\left\|f(Z;x,S) - f(Z;\widetilde{x},\widetilde{S})\right\|\right\|_{\psi_2} \\
&\leq \frac{1}{\sqrt{n}} \underbrace{\left\|\left\|f(Z;x,S) - f(Z;x,\widetilde{S})\right\|\right\|_{\psi_2}}_{\beta_1} + \frac{1}{\sqrt{n}} \underbrace{\left\|\left\|f(Z;x,\widetilde{S}) - f(Z;\widetilde{x},\widetilde{S})\right\|\right\|_{\psi_2}}_{\beta_2}
\end{aligned} \tag{107}$$

  - $\beta_1$: Let $g(Q;S) := (Q^{1/2}SQ^{1/2})^{1/2}$. Then for any $Q \in \mathcal{S}_d(M_{\widetilde{L}}^{-1}, M_{\widetilde{L}})$,

$$\begin{aligned}
\left\|g(Q;S) - g(Q;\widetilde{S})\right\|_{\mathrm{F}} &= \left\|(Q^{1/2}SQ^{1/2})^{1/2} - (Q^{1/2}\widetilde{S}Q^{1/2})^{1/2}\right\|_{\mathrm{F}} \\
&\lesssim \left\|Q^{1/2}SQ^{1/2} - Q^{1/2}\widetilde{S}Q^{1/2}\right\|_{\mathrm{F}}^{1/2} \\
&\leq \left\|S - \widetilde{S}\right\|_{\mathrm{F}}^{1/2} \|Q\|_{\mathrm{op}}^{1/2} \\
&\leq M_{\widetilde{L}}^{1/2} \left\|S - \widetilde{S}\right\|_{F}^{1/2}
\end{aligned}$$

    Here in the second inequality, we exploit the following the Hölder continuity of matrix square root in Wihler (2009); Carlsson (2018).

$$\left\|A^{1/2} - B^{1/2}\right\|_F \leq C_d \|A - B\|_F^{1/2}$$

    where $C_d$ is a constant only depending on dimension $d$. Hence for any $\theta, \widetilde{\theta} \in \Theta$,

$$\begin{aligned}
\beta_1 &= \left\|\left\|f(X,Q;x,S) - f(X,Q;x,\widetilde{S})\right\|\right\|_{\psi_2} \\
&\leq \left\|\left|1 + x^\top \widetilde{X}\right|\right\|_{\psi_2} \cdot \left\|\left(g(Q;S) - g(Q;\widetilde{S})\right) \cdot \mathbb{1}\left(Q \in \mathcal{S}_d\left(M_{\widetilde{L}}^{-1}, M_{\widetilde{L}}\right)\right)\right\|_{\mathrm{F}} \\
&\leq \left(1 + \|x\| \cdot \left\|\left\|\widetilde{X}\right\|_2\right\|_{\psi_2}\right) \cdot M_{\widetilde{L}}^{1/2} \left\|S - \widetilde{S}\right\|_{\mathrm{F}}^{1/2} \\
&\lesssim L \cdot M_{\widetilde{L}}^{1/2} \left\|S - \widetilde{S}\right\|_{\mathrm{F}}^{1/2}
\end{aligned} \tag{108}$$

  - $\beta_2$: for any $\theta, \widetilde{\theta} \in \Theta$, one has

$$\begin{aligned}
\beta_2 &= \left\|(x - \widetilde{x})^\top \widetilde{X} W^2(Q,S) \mathbb{1}\left(Q \in \mathcal{S}_d\left(M_{\widetilde{L}}^{-1}, M_{\widetilde{L}}\right)\right)\right\|_{\psi_2} \\
&\overset{(i)}{\lesssim} \|x - \widetilde{x}\| \left\|\widetilde{X}\right\|_{\psi_2} \cdot \left(M_{\widetilde{L}} + \widetilde{M}_L\right) \\
&\lesssim \widetilde{M}_L \|x - \widetilde{x}\|_2
\end{aligned} \tag{109}$$

    Here (i) follows from (95).

– As a result of (108) and (109), one can obtain

$$\begin{aligned}\left\|f(Z;\theta)-f(Z;\widetilde{\theta})\right\|_{\psi_2} &\le \beta_1+\beta_2\\ &\lesssim LM_{\widetilde{L}}^{1/2}d(\theta,\widetilde{\theta})^{1/2}+\widetilde{M}_L d(\theta,\widetilde{\theta})\\ &\lesssim L\widetilde{M}_L\left(d(\theta,\widetilde{\theta})^{1/2}\vee d(\theta,\widetilde{\theta})\right) \qquad (110)\end{aligned}$$

Combining (107) and (110) then gives

$$\left\|\alpha_1(\theta)-\alpha_1(\widetilde{\theta})\right\|_{\psi_2}\lesssim \frac{L\widetilde{M}_L}{\sqrt{n}}\left(d(\theta,\widetilde{\theta})^{1/2}\vee d(\theta,\widetilde{\theta})\right)$$

Hence $\tau(\epsilon)$ in Lemma 29 can be chosen as

$$\tau(\epsilon)=\frac{K}{\sqrt{n}}\left(\epsilon^{1/2}\vee\epsilon\right),\quad K=C_f L\widetilde{M}_L \qquad (111)$$

- by Lemma 29, one has

$$b_2:=\mathbb{E}\sup_{\theta\in\Theta}\left|\alpha_1(Z;\theta)\right|\lesssim \underbrace{\frac{KD}{\sqrt{n}}\sqrt{\log^+ D}}_{\zeta_1}+\underbrace{\sup_{\theta\in\Theta}\mathbb{E}\left|\alpha_1(Z;\theta)\right|}_{\zeta_2}$$

– $\zeta_1$: by (42) in Lemma 29 and (105), one can obtain

$$\zeta_1\lesssim\frac{L\widetilde{M}_L^2}{\sqrt{n}}\sqrt{\log\widetilde{M}_L} \qquad (112)$$

– $\zeta_2$: For any $\theta\in\Theta$, we have

$$\begin{aligned}\|f(Z;\theta)\|_{\psi_2} &\le \||f(Z;\theta)|\|_{\psi_2}\\ &\le \left\|\left|1+(x-\mu)^\top\Sigma^{-1}(\widetilde{X}-\mu)\right|\right\|_{\psi_2}\\ &\quad\cdot\sup_{S\in\mathcal{S}_d(0,\widetilde{M}_L)}\left[W^2(Q,S)\mathbb{1}\left(Q\in\mathcal{S}_d\left(M_{\widetilde{L}}^{-1},M_{\widetilde{L}}\right)\right)\right]\\ &\lesssim\left(1+\|x-\mu\|\left\|\left\|\widetilde{X}-\mu\right\|\right\|_{\psi_2}\right)\cdot\left(M_{\widetilde{L}}+\widetilde{M}_L\right)\\ &\lesssim L\widetilde{M}_L\end{aligned}$$

Therefore for any $\theta\in\Theta$, one has $\|\alpha_1(\theta)\|_{\psi_2}\lesssim L\widetilde{M}_L/\sqrt{n}$ and

$$\mathbb{E}\left|\alpha_1(Z;\theta)\right|\lesssim\|\alpha_1(\theta)\|_{\psi_2}\lesssim\frac{L\widetilde{M}_L}{\sqrt{n}}$$

Take supremum over $\theta\in\Theta$ to see that

$$\zeta_2\lesssim\frac{L\widetilde{M}_L}{\sqrt{n}} \qquad (113)$$

– As a result of (112) and (113), one can obtain

$$b_2 \lesssim \frac{L\widetilde{M}_L^2}{\sqrt{n}}\sqrt{\log \widetilde{M}_L} \tag{114}$$

Finally, combine (104) and (114) to see that

$$\sup_{\theta\in\Theta}|\alpha_1(\theta)| \le b_1 + b_2 \lesssim C\frac{L\widetilde{M}_L^2}{\sqrt{n}}\sqrt{\log \widetilde{M}_L} \tag{115}$$

with probability at least $1 - O\left(n^{-(1+\tau)}\right)$. The proof of Claim 3 is then complete.

### C.4 Proof of Lemma 38

Without loss of generality, assume $\mu = 0$, $\Sigma = I_p$ and $C_{\psi_2} = 1$ in Assumption 1 and Assumption 2. To prove the uniform convergence of $\widehat{Q}_\rho$ in (81), observe that for any $\delta_n, \epsilon_n > 0$, one has

$$\begin{aligned}
&\left\{\sup_{x\in B_\mu(L)}\left\|\widehat{Q}_\rho(x) - Q^*(x)\right\|_{\mathrm{F}} \le \delta_n\right\}\\
&\overset{(i)}{\supset}\left\{\inf_{S:\|S-Q^*(x)\|_{\mathrm{F}}\le\delta_n}\widehat{F}_\rho(x,S) < \inf_{S:\|S-Q^*(x)\|_{\mathrm{F}}\ge\delta_n}\widehat{F}_\rho(x,S), \forall x\in B_\mu(L)\right\}\\
&\overset{(ii)}{\supset}\left\{\widehat{F}_\rho(x,Q^*(x)) < \inf_{S:\|S-Q^*(x)\|_{\mathrm{F}}\ge\delta_n}\widehat{F}_\rho(x,S), \forall x\in B_\mu(L)\right\}\cap\left\{\widehat{Q}_\rho(x)\preceq\widetilde{M}_L I_d, \forall x\in B_\mu(L)\right\}\\
&\overset{(iii)}{\supset}\left\{\widehat{F}_\rho(x,Q^*(x)) < \inf_{\substack{S:\|S-Q^*(x)\|_{\mathrm{F}}\ge\delta_n\\ S\preceq\widetilde{M}_L I_d}}\widehat{F}_\rho(x,S), \forall x\in B_\mu(L)\right\}\cap\left\{\widehat{Q}_\rho(x)\preceq\widetilde{M}_L I_d, \forall x\in B_\mu(L)\right\}\\
&\supset\underbrace{\left\{\sup_{\substack{x\in B_\mu(L)\\ S\preceq\widetilde{M}_L I_d}}\left|\widehat{F}_\rho(x,S) - F(x,S)\right| \le \epsilon_n\right\}}_{=:\mathcal{E}_1}\\
&\quad\cap\underbrace{\left\{F(x,Q^*(x)) \le \inf_{\substack{S:\|S-Q^*(x)\|_{\mathrm{F}}\ge\delta_n\\ S\preceq\widetilde{M}_L I_d}}F(x,S) - 3\epsilon_n, \forall x\in B_\mu(L)\right\}}_{=:\mathcal{E}_2}\\
&\quad\cap\underbrace{\left\{\widehat{Q}_\rho(x)\preceq\widetilde{M}_L I_d, \forall x\in B_\mu(L)\right\}}_{=\widetilde{E}_0\text{ in Lemma 36}}
\end{aligned}\tag{116}$$

Here (i) and (iii) follow since $\widehat{Q}_\rho(x)$ is the minimizer of $F_{n,\rho}(x,\cdot)$; (ii) is obtained by setting $S = Q^*(x)$.

With (116) in place, it suffices to choose appropriate $\delta_n, \epsilon_n$ such that $\mathcal{E}_1\cap\mathcal{E}_2$ occurs with high probability and define $E_2 = \mathcal{E}_1\cap\mathcal{E}_2\cap\widetilde{E}_0$. To this end, we first find the uniform

convergence rate $\epsilon_n$ so that $\mathbb{P}(\mathcal{E}_1^c) \lesssim n^{-\tau}$. Next, $\delta_n$ is set based on $\epsilon_n$ and properties of $F$ so that $\mathbb{P}(\mathcal{E}_2^c) \lesssim n^{-\tau}$. For $\widetilde{E}_0$, we already showed in (80) that $\mathbb{P}(\widetilde{E}_0^c) \lesssim n^{-\tau}$. Combining results on $\mathcal{E}_1, \mathcal{E}_2$ and $\widetilde{E}_0$, the proof is then complete.

**Analysis of $\mathcal{E}_1$:** Let $\epsilon_0 = \delta_F^{\alpha_F} / \gamma_F(L, \sqrt{d}\widetilde{M}_L)$ ($\delta_F$ and $\gamma_F(\cdot,\cdot)$ are defined in Assumption 4) and set

$$\epsilon_n := C_{F,\tau} \frac{L\widetilde{M}_L^3}{\sqrt{n}} \wedge \frac{\epsilon_0}{3}$$

Note that for $L = O\left(\sqrt{\log n}\right)$, $C_{F,\tau}\frac{L\widetilde{M}_L^3}{\sqrt{n}} \le \epsilon_0/3$ for large $n$. Therefore, Lemma 37 implies that for any $\tau \ge 0$,

$$\mathbb{P}\left\{\mathcal{E}_1\right\} \ge 1 - c_{\delta,\tau} n^{-\tau}$$

for some constant $c_{\delta,\tau} > 0$ independent of $n$.

**Analysis of $\mathcal{E}_2$:** Note that for any $x \in B_\mu(L)$ and $S \preceq \widetilde{M}_L I_d$, one has

$$\|S - Q^*(x)\|_{\mathrm{F}} \le \sqrt{d} \max\left\{\|S\|_{\mathrm{op}}, \|Q^*(x)\|_{\mathrm{op}}\right\} \le \sqrt{d}\widetilde{M}_L$$

Hence one has

$$\inf_{\substack{S: \|S - Q^*(x)\|_{\mathrm{F}} \ge \delta_n \\ S \preceq \widetilde{M}_L I_d}} F(x,S) - F(x, Q^*(x)) \ge \inf_{S: \delta_n \le \|S - Q^*(x)\|_{\mathrm{F}} \le \sqrt{d}\widetilde{M}_L} F(x,S) - F(x,Q^*(x))$$

Set

$$\delta_n := \left[3\epsilon_n \gamma_F(L, \sqrt{d}\widetilde{M}_L)\right]^{1/\alpha_F}$$

By definition, $\delta_n \le \delta_F$. Therefore, Assumption 4 implies that $\mathbb{P}(\mathcal{E}_2) = 1$ for $n$ large enough.

Combining the above results on $\mathcal{E}_1, \mathcal{E}_2$ and $\widetilde{E}_0$, one can obtain

$$\mathbb{P}\left(\sup_{x \in B_\mu(L)} \left\|\widehat{Q}_\rho(x) - Q^*(x)\right\|_{\mathrm{F}} \le C_{\delta,\tau}\left[\frac{L\widetilde{M}_L^3 \gamma_F(L, \sqrt{d}\widetilde{M}_L)}{\sqrt{n}}\right]^{1/\alpha_F}\right) \ge 1 - c_{\delta,\tau} n^{-\tau}$$

for constants $C_{\delta,\tau}, c_{\delta,\tau}$ independent of $n$. Note that by definition, $L, \widetilde{M}_L, \gamma_F(L, \sqrt{d}\widetilde{M}_L) \lesssim \mathrm{polylog}\,(n)$, one can then arrive at (81).

Finally, Eq. (82) follows by noticing that $\delta_F \wedge \delta_n = o\,(M_L)$.

### C.5 Proof of Lemma 40

Without loss of generality, assume $\mu = 0$, $\Sigma = I_p$, and $C_{\psi_2} = 1$ in Assumption 1-Assumption 2. If $X$ is unbounded, then one can apply the truncation as (98) and follow similar arguments as in Lemma 38. Hence we can assume without loss of generality that $\|X - \mu\| \le \widetilde{L}$ almost surely with $\widetilde{L} := C_{\psi_2} L_\tau = C_{\psi_2}\sqrt{(1+\tau)\log n}$. Then we have a.s.

$$\begin{aligned} &\|X - \mu\| \le \widetilde{L} \\ &Q, Q^*(X) \in \mathcal{S}_d(M_{\widetilde{L}}^{-1}, M_{\widetilde{L}}) \\ &\mathbb{1}(E_0) = 1 \end{aligned} \tag{117}$$

where we remind readers that $E_0 := \left\{\|X_i - \mu\| \le \widetilde{L}, Q_i \in \mathcal{S}_d(M_{\widetilde{L}}^{-1}, M_{\widetilde{L}}), i \in [n]\right\}$ is defined in (78).

**Proof of (83):** one can obtain the following decomposition.

$$\sup_{x\in B_\mu(L)} \left\|\widetilde{A}_n(x)\right\|_F \le \underbrace{\sup_{x\in B_\mu(L)} \left\|\widetilde{A}_n(x)\right\|_F - \mathbb{E}\sup_{x\in B_\mu(L)} \left\|\widetilde{A}_n(x)\right\|_F}_{a_1} + \underbrace{\mathbb{E}\sup_{x\in B_\mu(L)} \left\|\widetilde{A}_n(x)\right\|_F}_{a_2} \tag{118}$$

Let $\varphi(Z;x) = w(x,X)\left(T_{Q^*(x)}^{Q} - I_d\right)$.
*Analysis of* $a_1$*:* By (117), one has almost surely that for any $x \in B_\mu(L)$,

$$\begin{aligned}\|\varphi(Z;x)\|_{\mathrm{F}} &\lesssim (1 + \|x-\mu\|\cdot\|X-\mu\|)\cdot \sup_{\substack{Q\in\mathcal{S}_d(M_{\widetilde{L}}^{-1}, M_{\widetilde{L}})\\ S\in\mathcal{S}_d((M_L)^{-1}, M_L)}} \left\|T_S^Q - I_d\right\|_{\mathrm{F}} \\ &\overset{(i)}{\lesssim} L\widetilde{L}M_{\widetilde{L}}^{1/2}M_L^{3/2}\end{aligned}$$

Here (i) follows by noticing that

$$\sup_{\substack{Q\in\mathcal{S}_d(M_{\widetilde{L}}^{-1}, M_{\widetilde{L}})\\ S\in\mathcal{S}_d((M_L)^{-1}, M_L)}} \left\|T_S^Q\right\|_{\mathrm{op}} = \sup_{\substack{Q\in\mathcal{S}_d(M_{\widetilde{L}}^{-1}, M_{\widetilde{L}})\\ S\in\mathcal{S}_d((M_L)^{-1}, M_L)}} \left\|S^{-1/2}(S^{1/2}QS^{1/2})^{1/2}S^{-1/2}\right\|_{\mathrm{op}} \le M_{\widetilde{L}}^{1/2}M_L^{3/2}$$

Lemma 31 then implies that $\|a_1\|_{\psi_2} \lesssim \frac{L\widetilde{L}M_{\widetilde{L}}^{1/2}M_L^{3/2}}{\sqrt{n}}$. Therefore, one can obtain

$$a_1 \lesssim \frac{L\widetilde{L}M_{\widetilde{L}}^{1/2}M_L^{3/2}L_\tau}{\sqrt{n}} \tag{119}$$

with probability at least $1 - O(n^{-(1+\tau)})$.
*Analysis of* $a_2$*:* to apply Lemma 29, we follow the steps below.

- First, we consider the sub-Gaussian norm of $\left\|\widetilde{A}_n(x)\right\|_{\mathrm{F}} - \left\|\widetilde{A}_n(\widetilde{x})\right\|_{\mathrm{F}}$ as in (40) in Lemma 29. For any $x, \widetilde{x} \in B_\mu(L)$, one has

$$\left\|\left\|\widetilde{A}_n(x)\right\|_{\mathrm{F}} - \left\|\widetilde{A}_n(\widetilde{x})\right\|_{\mathrm{F}}\right\|_{\psi_2} \le \left\|\left\|\widetilde{A}_n(x) - \widetilde{A}_n(\widetilde{x})\right\|_{\mathrm{F}}\right\|_{\psi_2} \tag{120}$$

  Note that

$$\widetilde{A}_n(x) - \widetilde{A}_n(\widetilde{x}) = \frac{1}{n}\sum_{i=1}^n \left[w(x,X_i)\left(T_{Q^*(x)}^{Q_i} - I_d\right) - w(\widetilde{x},X_i)\left(T_{Q^*(\widetilde{x})}^{Q_i} - I_d\right)\right]$$

Since $\mathbb{E}\widetilde{A}_n(x) = 0$ by the optimality condition (147), one has

$$\begin{aligned}\left\|\left\|\widetilde{A}_n(x) - \widetilde{A}_n(\widetilde{x})\right\|_{\mathrm{F}}\right\|_{\psi_2} &\overset{(i)}{\lesssim} \frac{1}{\sqrt{n}} \left\|\left\|w(x,X)\left(T^Q_{Q^*(x)} - I_d\right) - w(\widetilde{x},X)\left(T^Q_{Q^*(\widetilde{x})} - I_d\right)\right\|_{\mathrm{F}}\right\|_{\psi_2} \\ &\le \frac{1}{\sqrt{n}} \underbrace{\left\|\left\|w(x,X)\left(T^Q_{Q^*(x)} - I_d\right) - w(x,X)\left(T^Q_{Q^*(\widetilde{x})} - I_d\right)\right\|_{\mathrm{F}}\right\|_{\psi_2}}_{b_1} \\ &\quad + \frac{1}{\sqrt{n}} \underbrace{\left\|\left\|w(x,X)\left(T^Q_{Q^*(\widetilde{x})} - I_d\right) - w(\widetilde{x},X)\left(T^Q_{Q^*(\widetilde{x})} - I_d\right)\right\|_{\mathrm{F}}\right\|_{\psi_2}}_{b_2} \end{aligned} \tag{121}$$

Here (i) follows from (43) in Lemma 29.

- $b_1$: For any $x, \widetilde{x} \in B_\mu(L)$, one has $Q^*(x), Q^*(\widetilde{x}) \in \mathcal{S}_d(M_L^{-1}, M_L)$. Then one can obtain

$$\begin{aligned} b_1 &= \left\|\left\|w(x,X)\left(T^Q_{Q^*(x)} - T^Q_{Q^*(\widetilde{x})}\right)\right\|_{\mathrm{F}}\right\|_{\psi_2} \\ &\lesssim \left\| |1 + \|x-\mu\| \cdot \|X-\mu\|| \cdot \left\|T^Q_{Q^*(x)} - T^Q_{Q^*(\widetilde{x})}\right\|_{\mathrm{F}}\right\|_{\psi_2} \\ &\lesssim \||1 + \|x-\mu\| \cdot \|X-\mu\||\|_{\psi_2} \cdot \sup_{Q \in \mathcal{S}(M_{\widetilde{L}}^{-1}, M_{\widetilde{L}})} \left\|T^Q_{Q^*(x)} - T^Q_{Q^*(\widetilde{x})}\right\|_{\mathrm{F}} \\ &\lesssim L \cdot \sup_{\substack{Q \in \mathcal{S}_d(M_{\widetilde{L}}^{-1}, M_{\widetilde{L}}) \\ x, \widetilde{x} \in B_\mu(L)}} \left\|T^Q_{Q^*(x)} - T^Q_{Q^*(\widetilde{x})}\right\|_{\mathrm{F}} \\ &\overset{(i)}{=} L \cdot \sup_{\substack{Q \in \mathcal{S}_d(M_{\widetilde{L}}^{-1}, M_{\widetilde{L}}) \\ Q' \in \mathcal{S}_d(M_L^{-1}, M_L)}} \left\|dT^Q_{Q'} \cdot (Q^*(x) - Q^*(\widetilde{x}))\right\|_{\mathrm{F}} \\ &\overset{(ii)}{\le} L \cdot \|Q^*(x) - Q^*(\widetilde{x})\|_{\mathrm{F}} \cdot \sup_{\substack{Q \in \mathcal{S}_d(M_{\widetilde{L}}^{-1}, M_{\widetilde{L}}) \\ S \in \mathcal{S}_d(M_L^{-1}, M_L)}} \left\|\left|dT^Q_S\right|\right\| \\ &\overset{(iii)}{\lesssim} L \cdot \mathrm{poly}\left(M_L, M_{\widetilde{L}}\right) \cdot \|Q^*(x) - Q^*(\widetilde{x})\|_{\mathrm{F}} \end{aligned} \tag{122}$$

  Here (i) is a consequence of the mean value theorem (Dudley and Norvaiša, 2011, Theorem 5.3) for some $Q'$ that lies between $Q^*(x)$ and $Q^*(\widetilde{x})$, (ii) arises from Lemma 22, and (iii) follows from Lemma 21.

- $b_2$: one can obtain that

$$\begin{aligned} b_2 &\le \left\|\left|(x-\widetilde{x})\Sigma^{-1}(X-\mu)\right|\right\|_{\psi_2} \sup_{\substack{Q \in \mathcal{S}_d(M_{\widetilde{L}}^{-1}, M_{\widetilde{L}}) \\ S \in \mathcal{S}_d(M_L^{-1}, M_L)}} \left\|T^Q_S - I_d\right\|_{\mathrm{F}} \\ &\overset{(i)}{\lesssim} \|x-\widetilde{x}\| \, \|\|X-\mu\|\|_{\psi_2} \mathrm{poly}\left(M_L, M_{\widetilde{L}}\right) \\ &\lesssim \|x-\widetilde{x}\| \, \mathrm{poly}\left(M_L, M_{\widetilde{L}}\right) \end{aligned} \tag{123}$$

almost surely. Here (i) arises from Lemma 25 and the bounds on $T_S^Q$ in Lemma 21.

Combining (120), (121), (122) and (123) gives that for any $x, \widetilde{x} \in B_\mu(L)$,

$$\begin{aligned}
&\left\| \left\| \widetilde{A}_n(x) \right\|_{\mathrm{F}} - \left\| \widetilde{A}_n(\widetilde{x}) \right\|_{\mathrm{F}} \right\|_{\psi_2} \\
&\lesssim \frac{1}{\sqrt{n}} \cdot L\mathrm{poly}\left(M_L, M_{\widetilde{L}}\right) \left(\|x - \widetilde{x}\| + \|Q^*(x) - Q^*(\widetilde{x})\|_F\right) \\
&\overset{(i)}{\lesssim} \frac{1}{\sqrt{n}} \cdot L\mathrm{poly}\left(M_L, M_{\widetilde{L}}\right) \left(\|x - \widetilde{x}\| + M_L \gamma_F(L, M_L) \left(\|x - \widetilde{x}\|_2 \vee \|x - \widetilde{x}\|^{1/\alpha_F}\right)\right) \\
&\overset{(ii)}{\leq} \frac{C}{\sqrt{n}} \cdot L^{1+C_F} M_L^{4+C_F} \left(\|x - \widetilde{x}\|_2 \vee \|x - \widetilde{x}\|^{1/\alpha_F}\right)
\end{aligned} \tag{124}$$

Here (i) follows from Lemma 35 and the fact that $M\gamma_F(L, M) \geq 1$.

- With the Hölder continuity (124) in place, we can apply Lemma 29 with $\tau(\epsilon)$ chosen as

$$\tau(\epsilon) = \frac{K}{\sqrt{n}} \left(\epsilon^{1/\alpha_F} \vee \epsilon\right), \quad K = CL^{1+C_F} M_L^{4+C_F}$$

which gives

$$\begin{aligned}
a_2 &\lesssim \frac{KL^{1 \vee \alpha_F^{-1}}}{\sqrt{n}} \sqrt{\log^+ L} + \sup_{x \in B_\mu(L)} \mathbb{E} \left\| \widetilde{A}_n(x) \right\|_{\mathrm{F}} \\
&\overset{(i)}{=} \underbrace{\frac{KL}{\sqrt{n}} \sqrt{\log^+ L}}_{\zeta_1} + \underbrace{\sup_{x \in B_\mu(L)} \mathbb{E} \left\| \widetilde{A}_n(x) \right\|_{\mathrm{F}}}_{\zeta_2}
\end{aligned}$$

Here (i) follows since $\alpha_F \geq 1$ as defined in Assumption 4.

  - $\zeta_1$: direct calculation gives that

$$\zeta_1 \lesssim \frac{L^{2+C_F} M_L^{4+C_F}}{\sqrt{n}} \sqrt{\log^+ L} \tag{125}$$

– $\zeta_2$: for any $U \in \mathbb{R}^{d\times d}$ with unit Frobenius norm, one has

$$\begin{aligned}
\sup_{x\in B_\mu(L)} \left\| \left\| \widetilde{A}_n(x) \right\|_{\mathrm{F}} \right\|_{\psi_2} &\overset{(i)}{\lesssim} \sup_{x\in B_\mu(L)} \sup_{\|U\|_{\mathrm{F}}=1} \left\| \left\langle U, \widetilde{A}_n(x) \right\rangle \right\|_{\psi_2} \\
&\overset{(ii)}{\lesssim} \sup_{x\in B_\mu(L)} \sup_{\|U\|_{\mathrm{F}}=1} \left\| \left\langle U, \frac{1}{n}\sum_{i=1}^n \varphi(Z_i;x) - \mathbb{E}\varphi(Z;x) \right\rangle \right\|_{\psi_2} \\
&\lesssim \sup_{x\in B_\mu(L)} \sup_{\|U\|_{\mathrm{F}}=1} \frac{1}{\sqrt{n}} \left\| \langle U, \varphi(Z;x)\rangle \right\|_{\psi_2} \\
&\le \sup_{x\in B_\mu(L)} \frac{1}{\sqrt{n}} \left\| \|\varphi(Z;x)\|_{\mathrm{F}} \right\|_{\psi_2} \\
&\le \frac{1}{\sqrt{n}} \sup_{x\in B_\mu(L)} \| |w(x,X)| \|_{\psi_2} \cdot \sup_{\substack{Q\in\mathcal{S}_d(M_{\widetilde{L}}^{-1}, M_{\widetilde{L}}) \\ S\in\mathcal{S}_d(M_L^{-1}, M_L)}} \left\| T_S^Q - I_d \right\|_{\mathrm{F}} \\
&\lesssim \frac{1}{\sqrt{n}} L \cdot \mathrm{poly}\left(M_L, M_{\widetilde{L}}\right)
\end{aligned}$$

Here (i) results from Lemma 25, (ii) follows from independence. Therefore,

$$\zeta_2 \lesssim \frac{L\cdot \mathrm{poly}\left(M_L, M_{\widetilde{L}}\right)}{\sqrt{n}} \tag{126}$$

- As a result of (125) and (126), one can obtain

$$a_2 \lesssim \frac{\mathrm{poly}\left(L, M_L, M_{\widetilde{L}}\right)}{\sqrt{n}} \sqrt{\log^+ L} \tag{127}$$

Finally, combining (118), (119) and (127) gives (83).

**Proof of (84):** one can obtain the following decomposition.

$$\begin{aligned}
\sup_{x\in B_\mu(L)} \left\| \widetilde{\Phi}_n(x) - \mathbb{E}\widetilde{\Phi}_n(x) \right\| \le{}& \underbrace{\sup_{x\in B_\mu(L)} \left\| \widetilde{\Phi}_n(x) - \mathbb{E}\widetilde{\Phi}_n(x) \right\| - \mathbb{E} \sup_{x\in B_\mu(L)} \left\| \widetilde{\Phi}_n(x) - \mathbb{E}\widetilde{\Phi}_n(x) \right\|}_{a_3} \\
&+ \underbrace{\mathbb{E} \sup_{x\in B_\mu(L)} \left\| \widetilde{\Phi}_n(x) - \mathbb{E}\widetilde{\Phi}_n(x) \right\|}_{a_4}
\end{aligned} \tag{128}$$

Let $Z = (X, Q)$. Define $\phi(Z;x) = w(x,X) dT^Q_{Q^*(x)}$.
*Analysis of $a_3$:* By (117), one has almost surely that for any $x \in B_\mu(L)$,

$$\begin{aligned}
\|\phi(Z;x)\| &\lesssim L\widetilde{L} \cdot \sup_{\substack{Q\in\mathcal{S}_d(M_{\widetilde{L}}^{-1}, M_{\widetilde{L}}) \\ S\in\mathcal{S}_d(M_L^{-1}, M_L)}} \left\| dT_S^Q \right\| \\
&\lesssim L\widetilde{L}\mathrm{poly}\left(M_L, M_{\widetilde{L}}\right)
\end{aligned}$$

By Lemma 31, one can then obtain

$$\|a_3\|_{\psi_2} \lesssim \frac{L\widetilde{L}\mathrm{poly}\left(M_L, M_{\widetilde{L}}\right)}{\sqrt{n}} \tag{129}$$

*Analysis of $a_4$:* to apply Lemma 29, we follow the steps below.

- First, let us consider the sub-Gaussian norm of $\left\|\left\|\widetilde{\Phi}_n(x) - \mathbb{E}\widetilde{\Phi}_n(x)\right\|\right\| - \left\|\left\|\widetilde{\Phi}_n(\widetilde{x}) - \mathbb{E}\widetilde{\Phi}_n(\widetilde{x})\right\|\right\|$. For any $x, \widetilde{x} \in B_\mu(L)$, by (43) in Lemma 29, one can obtain

$$\left\|\left\|\left\|\widetilde{\Phi}_n(x) - \mathbb{E}\widetilde{\Phi}_n(x)\right\|\right\| - \left\|\left\|\widetilde{\Phi}_n(\widetilde{x}) - \mathbb{E}\widetilde{\Phi}_n(\widetilde{x})\right\|\right\|\right\|_{\psi_2} \lesssim \frac{1}{\sqrt{n}} \left\|\left\|\phi(Z;x) - \phi(Z;\widetilde{x})\right\|\right\|_{\psi_2} \tag{130}$$

  Moreover,

$$\begin{aligned} &\left\|\left\|\phi(Z;x) - \phi(Z;\widetilde{x})\right\|\right\|_{\psi_2} \\ &= \left\|\left\|w(x,X)dT^Q_{Q^*(x)} - w(\widetilde{x},X)dT^Q_{Q^*(\widetilde{x})}\right\|\right\|_{\psi_2} \\ &\le \underbrace{\left\|\left\|w(x,X)dT^Q_{Q^*(x)} - w(x,X)dT^Q_{Q^*(\widetilde{x})}\right\|\right\|_{\psi_2}}_{b_3} \\ &+ \underbrace{\left\|\left\|w(x,X)dT^Q_{Q^*(\widetilde{x})} - w(\widetilde{x},X)dT^Q_{Q^*(\widetilde{x})}\right\|\right\|_{\psi_2}}_{b_4} \end{aligned} \tag{131}$$

  - $b_3$: we have for any $x, \widetilde{x} \in B_\mu(L)$,

$$\begin{aligned} b_3 &\overset{(i)}{=} \left\|\left\|w(x,X)d^2T^Q_{Q'} \cdot (Q^*(x) - Q^*(\widetilde{x}))\right\|\right\|_{\psi_2} \\ &\lesssim L\left\|X-\mu\right\|_{\psi_2} \cdot \left\|Q^*(x) - Q^*(\widetilde{x})\right\|_{\mathrm{F}} \cdot \sup_{\substack{Q\in\mathcal{S}_d(M_{\widetilde{L}}^{-1}, M_{\widetilde{L}}) \\ S\in\mathcal{S}_d(M_L^{-1}, M_L)}} \left\|\left\|d^2T^Q_S\right\|\right\| \\ &\overset{(ii)}{\lesssim} L \cdot \mathrm{poly}\left(M_L, M_{\widetilde{L}}\right)\left\|Q^*(x) - Q^*(\widetilde{x})\right\|_{\mathrm{F}} \end{aligned} \tag{132}$$

    Here (i) is a consequence of the mean value theorem (Dudley and Norvaiša, 2011, Theorem 5.3) for some $Q'$ that lies between $Q^*(x)$ and $Q^*(\widetilde{x})$ and (ii) follows from Lemma 21.

  - $b_4$: for any $x, \widetilde{x} \in B_\mu(L)$,

$$\begin{aligned} b_4 &\le \left\||w(x,X) - w(\widetilde{x},X)|\right\|_{\psi_2} \sup_{\substack{Q\in\mathcal{S}_d(M_{\widetilde{L}}^{-1}, M_{\widetilde{L}}) \\ S\in\mathcal{S}_d(M_L^{-1}, M_L)}} \left\|\left\|dT^Q_S\right\|\right\| \\ &\lesssim \mathrm{poly}\left(M_L, M_{\widetilde{L}}\right)\left\|x - \widetilde{x}\right\|_2 \end{aligned} \tag{133}$$

Combining (130), (131), (132) and (133), one can obtain that

$$\begin{aligned}
&\left\| \left\| \widetilde{\Phi}_n(x) - \mathbb{E}\widetilde{\Phi}_n(x) \right\| - \left\| \widetilde{\Phi}_n(\widetilde{x}) - \mathbb{E}\widetilde{\Phi}_n(\widetilde{x}) \right\| \right\|_{\psi_2} \\
&\lesssim \frac{1}{\sqrt{n}} \cdot L\mathrm{poly}\left(M_L, M_{\widetilde{L}}\right) \left(\|x - \widetilde{x}\| + \|Q^*(x) - Q^*(\widetilde{x})\|_{\mathrm{F}}\right) \\
&\lesssim \frac{1}{\sqrt{n}} \cdot L\mathrm{poly}\left(M_L, M_{\widetilde{L}}\right) \left(\|x - \widetilde{x}\| \vee \|x - \widetilde{x}\|^{1/\alpha_F}\right)
\end{aligned}$$

- With the Hölder continuity above, we can apply Lemma 29 with $\tau(\epsilon)$ chosen as

$$\tau(\epsilon) = \frac{K}{\sqrt{n}} \left(\epsilon^{1/\alpha_F} \vee \epsilon\right), \quad K = \mathrm{poly}\left(L, M_L\right)$$

which gives

$$a_4 \lesssim \underbrace{\frac{KL}{\sqrt{n}} \sqrt{\log^+ L}}_{\zeta_3} + \underbrace{\sup_{x \in B_\mu(L)} \mathbb{E} \left\| \widetilde{\Phi}_n(x) \right\|}_{\zeta_4}$$

  - $\zeta_3$: direct calculation gives

$$\zeta_3 \lesssim \frac{\mathrm{poly}\left(L, M_L\right)}{\sqrt{n}} \tag{134}$$

  - $\zeta_4$: for any $U, V \in \mathbb{R}^{d \times d}$ with unit Frobenius norm, one has

$$\begin{aligned}
\sup_{x \in B_\mu(L)} \left\| \left\| \widetilde{\Phi}_n(x) \right\| \right\|_{\psi_2} &\overset{(i)}{\lesssim} \sup_{x \in B_\mu(L)} \sup_{\|U\|_{\mathrm{F}} = \|V\|_{\mathrm{F}} = 1} \left\| \left\langle V, \widetilde{\Phi}_n(x) \cdot U \right\rangle \right\|_{\psi_2} \\
&\overset{(ii)}{\lesssim} \sup_{x \in B_\mu(L)} \sup_{\|U\|_{\mathrm{F}} = \|V\|_{\mathrm{F}} = 1} \frac{1}{\sqrt{n}} \left\| \langle V, [\phi(Z; x) - E\phi(Z; x)] \cdot U \rangle \right\|_{\psi_2} \\
&\lesssim \sup_{x \in B_\mu(L)} \sup_{\|U\|_{\mathrm{F}} = \|V\|_{\mathrm{F}} = 1} \frac{1}{\sqrt{n}} \left\| \langle V, \phi(Z; x) \cdot U \rangle \right\|_{\psi_2} \\
&\le \sup_{x \in B_\mu(L)} \frac{1}{\sqrt{n}} \left\| \|\phi(Z; x)\| \right\|_{\psi_2} \\
&\le \frac{1}{\sqrt{n}} \sup_{x \in B_\mu(L)} \left\| |w(x, X)| \right\|_{\psi_2} \cdot \sup_{\substack{Q \in \mathcal{S}_d(M_{\widetilde{L}}^{-1}, M_{\widetilde{L}}) \\ S \in \mathcal{S}_d(M_L^{-1}, M_L)}} \left\| dT_S^Q \right\| \\
&\overset{(iii)}{\lesssim} \frac{1}{\sqrt{n}} L\mathrm{poly}\left(M_L, M_{\widetilde{L}}\right)
\end{aligned}$$

    Here (i) results from Lemma 25, (ii) follows from independence and (iii) is due to Lemma 21. Hence

$$\zeta_4 \lesssim \frac{L\mathrm{poly}\left(M_L, M_{\widetilde{L}}\right)}{\sqrt{n}} \tag{135}$$

- As a result of (134) and (135), one can obtain

$$a_4 \lesssim \frac{\mathrm{poly}\left(L, M_L, M_{\widetilde{L}}\right)}{\sqrt{n}} \tag{136}$$

Finally, combining (128), (129) and (136) gives (84).

**Proof of (85):** Denote $\Theta = B_\mu(L) \times \mathcal{S}_d((C_{\text{slow}} M_{\widetilde{L}})^{-1}, C_{\text{slow}} M_{\widetilde{L}})$ and $\theta = (x, S) \in \Theta$.

$$\begin{aligned}\sup_{\theta\in\Theta}\left\|\widetilde{\Psi}_n(\theta) - \mathbb{E}\widetilde{\Psi}_n(\theta)\right\| \leq& \underbrace{\sup_{\theta\in\Theta}\left\|\widetilde{\Psi}_n(\theta) - \mathbb{E}\widetilde{\Psi}_n(\theta)\right\| - \mathbb{E}\sup_{\theta\in\Theta}\left\|\widetilde{\Psi}_n(\theta) - \mathbb{E}\widetilde{\Psi}_n(\theta)\right\|}_{a_5} \\ &+ \underbrace{\mathbb{E}\sup_{\theta\in\Theta}\left\|\widetilde{\Psi}_n(\theta) - \mathbb{E}\widetilde{\Psi}_n(\theta)\right\|}_{a_6}\end{aligned} \tag{137}$$

Let $Z = (X, Q)$. Define $\psi(Z;\theta) = w(x, X) d^2 T_S^Q$ and $\overline{\psi}(Z;\theta) = \psi(Z;\theta) - \mathbb{E}\psi(Z;\theta)$
*Analysis of* $a_5$*:* By (117), one has almost surely that

$$\begin{aligned}\sup_{Z,\theta}\|\psi(Z;\theta)\| &\leq \sup_{\substack{x\in B_\mu(L)\\ X\in B_\mu(\widetilde{L})}} |w(x,X)| \cdot \sup_{\substack{Q\in\mathcal{S}_d(M_{\widetilde{L}}^{-1}, M_{\widetilde{L}})\\ S\in\mathcal{S}_d((C_{\text{slow}}M_L)^{-1}, C_{\text{slow}}M_L)}} \left\|d^2 T_S^Q\right\| \\ &\lesssim L\widetilde{L}\cdot \text{poly}\left(M_L, M_{\widetilde{L}}\right)\end{aligned}$$

Lemma 31 then implies that , $\|a_5\|_{\psi_2} \lesssim \frac{L\widetilde{L}\cdot\text{poly}\left(M_{\widetilde{L}}M_L\right)}{\sqrt{n}}$. Therefore, one can obtain

$$a_5 \lesssim L\widetilde{L}\cdot\text{poly}\left(M_L M_{\widetilde{L}}\right)\frac{L_\tau}{\sqrt{n}} \tag{138}$$

with probability at least $1 - O(n^{-(1+\tau)})$.
*Analysis of* $a_6$*:* to apply Lemma 29, we follow the steps below.

- First, let us consider the sub-Gaussian norm of $\left\|\widetilde{\Psi}_n(\theta) - \mathbb{E}\widetilde{\Psi}_n(\theta)\right\| - \left\|\widetilde{\Psi}_n(\widetilde{\theta}) - \mathbb{E}\widetilde{\Psi}_n(\widetilde{\theta})\right\|$. For any $\theta, \widetilde{\theta} \in \Theta$, one has

$$\left\|\left\|\widetilde{\Psi}_n(\theta) - \mathbb{E}\widetilde{\Psi}_n(\theta)\right\| - \left\|\widetilde{\Psi}_n(\widetilde{\theta}) - \mathbb{E}\widetilde{\Psi}_n(\widetilde{\theta})\right\|\right\|_{\psi_2} \overset{(i)}{\lesssim} \frac{1}{\sqrt{n}}\left\|\left\|\psi(Z;\theta) - \psi(Z;\widetilde{\theta})\right\|\right\|_{\psi_2} \tag{139}$$

  Here (i) follows from Lemma 29. Moreover, one has

$$\begin{aligned}&\left\|\left\|\psi(Z;\theta) - \psi(Z;\widetilde{\theta})\right\|\right\|_{\psi_2} \\ =& \left\|\left\|w(x,X)d^2T_S^Q - w(\widetilde{x},X)d^2T_{\widetilde{S}}^Q\right\|\right\|_{\psi_2} \\ \leq& \underbrace{\left\|\left\|w(x,X)d^2T_S^Q - w(x,X)d^2T_{\widetilde{S}}^Q\right\|\right\|_{\psi_2}}_{b_5} + \underbrace{\left\|\left\|w(x,X)d^2T_{\widetilde{S}}^Q - w(\widetilde{x},X)d^2T_{\widetilde{S}}^Q\right\|\right\|_{\psi_2}}_{b_6}\end{aligned} \tag{140}$$

  - $b_5$: for any $\theta, \widetilde{\theta} \in \Theta$, one can obtain

$$b_5 \overset{(i)}{=} \left\|\left\|w(x,X)d^3T_{Q'}^Q \cdot (S - \widetilde{S})\right\|\right\|_{\psi_2}$$

$$
\begin{aligned}
&\leq \left\| \| w(x,X) \| \right\|_{\psi_2} \cdot \left\| S - \widetilde{S} \right\|_{\mathrm{F}} \cdot \sup_{\substack{Q \in \mathcal{S}_d(M_{\widetilde{L}}^{-1}, M_{\widetilde{L}}) \\ S \in \mathcal{S}_d((C_{\mathrm{slow}} M_L)^{-1}, C_{\mathrm{slow}} M_L)}} \left\| d^3 T_S^Q \right\| \\
&\overset{(ii)}{\lesssim} L \cdot \mathrm{poly}\left(M_L, M_{\widetilde{L}}\right) \left\| S - \widetilde{S} \right\|_{\mathrm{F}} \qquad (141)
\end{aligned}
$$

Here (i) is a consequence of the mean value theorem (Dudley and Norvaiša, 2011, Theorem 5.3) for some $Q'$ that lies between $Q^*(x)$ and $Q^*(\widetilde{x})$ and (ii) follows from Lemma 21.

- $b_6$: for any $\theta, \widetilde{\theta} \in \Theta$, one has

$$
\begin{aligned}
b_6 &\leq \left\| \| w(x,X) - w(\widetilde{x},X) \| \right\|_{\psi_2} \sup_{\substack{Q \in \mathcal{S}_d(M_{\widetilde{L}}^{-1}, M_{\widetilde{L}}) \\ S \in \mathcal{S}_d((C_{\mathrm{slow}} M_L)^{-1}, C_{\mathrm{slow}} M_L)}} \left\| d^2 T_S^Q \right\| \\
&\lesssim \mathrm{poly}\left(M_L, M_{\widetilde{L}}\right) \| x - \widetilde{x} \| \qquad (142)
\end{aligned}
$$

Combining (130), (140), (141) and (142), one can obtain

$$
\left\| \left\| \widetilde{\Psi}_n(\theta) - \mathbb{E}\widetilde{\Psi}_n(\theta) \right\| - \left\| \widetilde{\Psi}_n(\widetilde{\theta}) - \mathbb{E}\widetilde{\Psi}_n(\widetilde{\theta}) \right\| \right\|_{\psi_2} \lesssim \frac{L\mathrm{poly}\left(M_L, M_{\widetilde{L}}\right)}{\sqrt{n}} \cdot d(\theta, \widetilde{\theta})
$$

- With the Lipschitz continuity above, we can apply Lemma 29 with $\tau(\epsilon)$ chosen as

$$
\tau(\epsilon) = \frac{K}{\sqrt{n}} \epsilon, \quad K = C \cdot L\mathrm{poly}\left(M_L, M_{\widetilde{L}}\right)
$$

which gives

$$
a_6 \lesssim \underbrace{\frac{K M_{\widetilde{L}}}{\sqrt{n}} \sqrt{\log^+ M_{\widetilde{L}}}}_{\zeta_5} + \underbrace{\sup_{\theta \in \Theta} \mathbb{E} \left\| \widetilde{\Psi}_n(\theta) \right\|}_{\zeta_6}
$$

- $\zeta_5$: direct calculation gives

$$
\zeta_5 \lesssim \frac{L\mathrm{poly}\left(M_L M_{\widetilde{L}}\right)}{\sqrt{n}} \qquad (143)
$$

- $\zeta_6$: for any $U, V \in \mathbb{R}^{d \times d}$ with unit Frobenius norm, one has

$$
\begin{aligned}
\left\| \left\langle V, \widetilde{\Psi}_n(\theta) \cdot U \right\rangle \right\|_{\psi_2} &\lesssim \frac{1}{\sqrt{n}} \left\| \langle V, [\psi(Z;\theta) - \mathbb{E}\psi(Z;\theta)] \cdot U \rangle \right\|_{\psi_2} \\
&\lesssim \frac{1}{\sqrt{n}} \left\| \langle V, \psi(Z;\theta) \cdot U \rangle \right\|_{\psi_2} \\
&\leq \frac{1}{\sqrt{n}} \left\| \| \psi(Z;\theta) \| \right\|_{\psi_2} \\
&\leq \frac{1}{\sqrt{n}} \left\| \| w(x,X) \| \right\|_{\psi_2} \cdot \sup_{\substack{Q \in \mathcal{S}_d(M_{\widetilde{L}}^{-1}, M_{\widetilde{L}}) \\ S \in \mathcal{S}_d((C_{\mathrm{slow}} M_L)^{-1}, C_{\mathrm{slow}} M_L)}} \left\| d^2 T_S^Q \right\| \\
&\lesssim \frac{1}{\sqrt{n}} L \cdot \mathrm{poly}\left(M_L M_{\widetilde{L}}\right)
\end{aligned}
$$

which combined with Lemma 25 implies

$$\zeta_6 \lesssim \frac{L\text{poly}\left(M_L M_{\widetilde{L}}\right)}{\sqrt{n}} \tag{144}$$

- As a result of (143) and (144), one can obtain

$$a_6 \lesssim \frac{L\text{poly}\left(M_L M_{\widetilde{L}}\right)}{\sqrt{n}} \tag{145}$$

Finally, combining (137), (138) and (145) gives (85).

**Proof of (86):** by definition, one has

$$\mathbb{E}\widetilde{\Psi}_n(x;S) = \mathbb{E}_{(X,Q)}\left[w(x,X)d^2T_S^Q\right]$$

Therefore, by the truncation assumption (117), one can obtain

$$\begin{aligned}
&\sup_{\substack{x\in B_\mu(L)\\ S\in\mathcal{S}_d\left((C_{\text{slow}}M_{\widetilde{L}})^{-1},C_{\text{slow}}M_{\widetilde{L}}\right)}} \left\|\left\|\mathbb{E}\widetilde{\Psi}_n(x;S)\right\|\right\| \\
\leq &\left[\sup_{x\in B_\mu(L)} \mathbb{E}\left|w(x,X)\right|\right]\cdot\left[\sup_{\substack{Q\in\mathcal{S}_d(M_{\widetilde{L}}^{-1},M_{\widetilde{L}})\\ S\in\mathcal{S}_d\left((C_{\text{slow}}M_L)^{-1},C_{\text{slow}}M_L\right)}} \left\|\left\|d^2T_S^Q\right\|\right\|\right] \\
\overset{(i)}{\lesssim} &L\cdot\text{poly}\left(M_L M_{\widetilde{L}}\right)
\end{aligned}$$

Here (i) follows from Lemma 21.

Finally, note that by definition, $\widetilde{L} \asymp \sqrt{\log n}$ and $M_{\widetilde{L}} = \text{poly}\left(\widetilde{L}\right)$, one can obtain (86).

### C.6 Proof of Lemma 41

As argued at the beginning of Appendix C.5, we can assume without loss of generality that $\|X-\mu\| \leq \widetilde{L}$ almost surely with $\widetilde{L}$ defined in (78). As a result, we have almost surely that

$$\begin{aligned}
\|X-\mu\| &\leq \widetilde{L} \\
Q, Q^*(X) &\in \mathcal{S}_d(M_{\widetilde{L}}^{-1}, M_{\widetilde{L}}) \\
\mathbb{1}(E_0) &= 1
\end{aligned} \tag{146}$$

where $E_0 := \left\{\|X_i-\mu\| \leq L, Q_i \in \mathcal{S}_d(M_{\widetilde{L}}^{-1}, M_{\widetilde{L}}), i\in[n]\right\}$.

With the boundedness condition (146) in place, Lemma 21 implies the following upper bounds

$$\sup_{\substack{Q\in\mathcal{S}_d(M_{\widetilde{L}}^{-1},M_{\widetilde{L}})\\ S\in\mathcal{S}_d((C_{\mathrm{slow}}M_L)^{-1},C_{\mathrm{slow}}M_L)}} \left\|T_S^Q - I_d\right\|_{\mathrm{F}} \le \mathrm{poly}\left(M_L, M_{\widetilde{L}}\right)$$
$$\sup_{\substack{Q\in\mathcal{S}_d(M_{\widetilde{L}}^{-1},M_{\widetilde{L}})\\ S\in\mathcal{S}_d((C_{\mathrm{slow}}M_L)^{-1},C_{\mathrm{slow}}M_L)}} \left\|\left|dT_S^Q\right|\right\| \le \mathrm{poly}\left(M_L, M_{\widetilde{L}}\right)$$
$$\sup_{\substack{Q\in\mathcal{S}_d(M_{\widetilde{L}}^{-1},M_{\widetilde{L}})\\ S\in\mathcal{S}_d((C_{\mathrm{slow}}M_L)^{-1},C_{\mathrm{slow}}M_L)}} \left\|\left|d^2T_S^Q\right|\right\| \le \mathrm{poly}\left(M_L, M_{\widetilde{L}}\right)$$

almost surely. Applying Lemma 28 then gives the desired results.

### C.7 Proof of Lemma 42

**Proof of (90):** apply the triangle inequality to see that

$$\begin{aligned}\sup_{x\in B_\mu(L)} \left\|\widehat{A}_\rho(x)\right\|_{\mathrm{F}} &\le \sup_{x\in B_\mu(L)} \left\|\widehat{A}_\rho(x) - \widetilde{A}_n(x)\right\|_{\mathrm{F}} + \sup_{x\in B_\mu(L)} \left\|\widetilde{A}_n(x)\right\|_{\mathrm{F}} \\ &\overset{(i)}{\le} \frac{\mathrm{polylog}\,(n)}{\sqrt{n}}\end{aligned}$$

Here (i) follows from Lemma 40 and Lemma 41

**Proof of (91):** apply the triangle inequality to see that

$$\begin{aligned}&\sup_{\substack{x\in B_\mu(L)\\ S\in\mathcal{S}_d((2M_L)^{-1},2M_L)}} \left\|\left|\widehat{\Psi}_\rho(x,S)\right|\right\| \\ &\le \sup_{\substack{x\in B_\mu(L)\\ S\in\mathcal{S}_d((2M_L)^{-1},2M_L)}} \left\|\left|\widehat{\Psi}_\rho(x,S) - \widetilde{\Psi}_n(x,S)\right|\right\| + \sup_{\substack{x\in B_\mu(L)\\ S\in\mathcal{S}_d((2M_L)^{-1},2M_L)}} \left\|\left|\widetilde{\Psi}_n(x,S) - \mathbb{E}\widetilde{\Psi}_n(x,S)\right|\right\| \\ &\quad + \sup_{\substack{x\in B_\mu(L)\\ S\in\mathcal{S}_d((2M_L)^{-1},2M_L)}} \left\|\left|\mathbb{E}\widetilde{\Psi}_n(x,S)\right|\right\| \\ &\overset{(i)}{\le} \frac{\mathrm{polylog}\,(n)}{\sqrt{n}} + \frac{\mathrm{polylog}\,(n)}{\sqrt{n}} + \mathrm{polylog}\,(n) \\ &\le \mathrm{polylog}\,(n)\end{aligned}$$

Here (i) follows from Lemma 40 and Lemma 41.

**Proof of (92), (93):** By the remark after Assumption 5 and the eigenvalue stability inequality, one can obtain

$$\begin{aligned}
&\inf_{x\in B_{\mu(L)}} \lambda_{\min}\left(-\widehat{\Phi}_\rho(x)\right)\\
\geq &\inf_{x\in B_{\mu(L)}} \lambda_{\min}\left(-\mathbb{E}\widetilde{\Phi}_n(x)\right) - \sup_{x\in B_{\mu(L)}} \left|\left|\left|\widetilde{\Phi}_n(x) - \mathbb{E}\widetilde{\Phi}_n(x)\right|\right|\right| - \sup_{x\in B_{\mu(L)}} \left|\left|\left|\widetilde{\Phi}_n(x) - \widehat{\Phi}_\rho(x)\right|\right|\right|\\
\overset{(i)}{\geq} &\frac{1}{\text{polylog}\,(n)} - \frac{\text{polylog}\,(n)}{\sqrt{n}} - \frac{\text{polylog}\,(n)}{\sqrt{n}}\\
\geq &\frac{1}{\text{polylog}\,(n)}
\end{aligned}$$

Here (i) follows by noticing that $\mathbb{E}(-\widetilde{\Phi}_n(x)) = \mathbb{E}\left(-w(x,X)dT^{Q}_{Q^*(x)}\right)$, Assumption 5 and applying Lemma 40, Lemma 41.

By the remark after Assumption 5 and (92), one then has

$$\left|\left|\left|-\widehat{\Phi}_\rho^{-1}(x)\right|\right|\right| = \lambda_{\max}\left(-\widehat{\Phi}_\rho^{-1}(x)\right) \leq \frac{1}{\lambda_{\min}\left(-\widehat{\Phi}_\rho(x)\right)}$$

Then (93) follows from (92).

### C.8 Proof of Lemma 43

Recall definitions of $Q^*(x)$ and $\widehat{Q}_\rho(x)$ that

$$\begin{aligned}
Q^*(x) &= \operatorname*{argmin}_{S\in\mathcal{S}_d^{++}} \mathbb{E}\left[w(x,X)W^2(S,Q)\right]\\
\widehat{Q}_\rho(x) &= \operatorname*{argmin}_{S\in\mathcal{S}_d^{++}} \frac{1}{n}\sum_{i=1}^n w_{n,\rho}(x,X_i)W^2(S,Q_i)
\end{aligned}$$

First, the differential properties of $W^2$ (Lemma 21) imply the following optimality conditions for $Q^*(x)$ and $\widehat{Q}_\rho(x)$.

$$\mathbb{E}w(x,X)\left(T^{Q}_{Q^*(x)} - I_d\right) = 0 \tag{147}$$

$$\frac{1}{n}\sum_{i=1}^n w_{n,\rho}(x,X_i)\left(T^{Q_i}_{\widehat{Q}_\rho(x)} - I_d\right) = 0 \tag{148}$$

Next, one can apply Lemma 21 again to get the 2nd order Taylor expansion of (148) around $Q^*(x)$ as follows.

$$\begin{aligned}
0 = &\frac{1}{n}\sum_{i=1}^n w_{n,\rho}(x,X_i)\left(T^{Q_i}_{Q^*(x)} - I_d\right) + \frac{1}{n}\sum_{i=1}^n w_{n,\rho}(x,X_i)dT^{Q_i}_{Q^*(x)}\cdot\left(\widehat{Q}_\rho(x) - Q^*(x)\right)\\
&+ \frac{1}{2n}\sum_{i=1}^n w_{n,\rho}(x,X_i)d^2T^{Q_i}_{\widetilde{Q}_n(x)}\cdot\left(\widehat{Q}_\rho(x) - Q^*(x)\right)^{\otimes 2}
\end{aligned}$$

where $\widetilde{Q}_n(x)$ lies between $Q^*(x)$ and $\widehat{Q}_\rho(x)$. Rearranging then gives

$$\widehat{Q}_\rho(x) - Q^*(x) = \left( \underbrace{-\frac{1}{n}\sum_{i=1}^n w_{n,\rho}(x, X_i) dT^{Q_i}_{Q^*(x)}}_{=\widehat{\Phi}_\rho(x)} \right)^{-1} \cdot \underbrace{\frac{1}{n}\sum_{i=1}^n w_{n,\rho}(x, X_i)\left(T^{Q_i}_{Q^*(x)} - I_d\right)}_{=\widehat{A}_\rho(x)}$$
$$+ \left(-\frac{1}{n}\sum_{i=1}^n w_{n,\rho}(x, X_i) dT^{Q_i}_{Q^*(x)}\right)^{-1} \cdot \frac{1}{2} \cdot \underbrace{\frac{1}{n}\sum_{i=1}^n w_{n,\rho}(x, X_i) d^2 T^{Q_i}_{\widetilde{Q}_n(x)}}_{=\widehat{\Psi}_\rho\left(x;\widetilde{Q}_{n,\rho}(x)\right)} \cdot (\widehat{Q}_\rho(x) - Q^*(x))^{\otimes 2} \tag{149}$$

Taking Frobenius norm on both sides of (149) gives the following quadratic inequality for $\left\|\widehat{Q}_\rho(x) - Q^*(x)\right\|_{\mathrm{F}}$.

$$\begin{aligned}&\left\|\widehat{Q}_\rho(x) - Q^*(x)\right\|_{\mathrm{F}} \\ &\leq \left\|\left|-\widehat{\Phi}_\rho(x)^{-1}\right\|\right| \cdot \left\|\widehat{A}_\rho(x)\right\|_{\mathrm{F}} + \frac{1}{2}\left\|\left|-\widehat{\Phi}_\rho(x)^{-1}\right\|\right| \cdot \left\|\left|\widehat{\Psi}_\rho(x, \widetilde{Q}(x))\right\|\right| \cdot \left\|\widehat{Q}_\rho(x) - Q^*(x)\right\|_{\mathrm{F}}^2\end{aligned} \tag{150}$$

With Lemma 42 in place, one can then derive from (150) that the following quadratic inequality holds uniformly for any $x \in B_\mu(L)$ under $\widetilde{E}_2$.

$$\left\|\widehat{Q}_\rho(x) - Q^*(x)\right\|_{\mathrm{F}} \leq a_0 + a_2\left\|\widehat{Q}_\rho(x) - Q^*(x)\right\|_{\mathrm{F}}^2$$

Here $a_0, a_2 > 0$ are uniform for any $x \in B_\mu(L)$ and satisfies

$$\begin{aligned}a_0 &= \widetilde{C}'_{\tau,1}\frac{\mathrm{polylog}\,(n)}{\sqrt{n}} \\ a_2 &= \widetilde{C}'_{\tau,2}\mathrm{polylog}\,(n)\,\mathrm{polylog}\,(n)\end{aligned}$$

for constants $\widetilde{C}'_{\tau,1}, \widetilde{C}'_{\tau,2} > 0$ independent of $n$.

Therefore, taking the supremum over $x$ gives

$$\sup_{x\in B_\mu(L)}\left\|\widehat{Q}_\rho(x) - Q^*(x)\right\|_{\mathrm{F}} \leq a_0 + a_2 \sup_{x\in B_\mu(L)}\left\|\widehat{Q}_\rho(x) - Q^*(x)\right\|_{\mathrm{F}}^2$$

Solving the above quadratic inequality for $\sup_{x\in B_\mu(L)}\left\|\widehat{Q}_\rho(x) - Q^*(x)\right\|_{\mathrm{F}}$ then gives

$$\sup_{x\in B_\mu(L)}\left\|\widehat{Q}_\rho(x) - Q^*(x)\right\|_{\mathrm{F}} \in \left[0, \frac{1-\sqrt{1-4a_0a_2}}{2a_2}\right] \cup \left[\frac{1+\sqrt{1-4a_0a_2}}{2a_2}, +\infty\right)$$

Since $\widetilde{E}_2 \subset \widetilde{E}_1$ and the slow rate of convergence (Lemma 38) implies that only the smaller branch should be retained. Therefore, one has

$$\begin{aligned}\sup_{x \in B_\mu(L)} \left\| \widehat{Q}_\rho(x) - Q^*(x) \right\|_{\mathrm{F}} &\leq \frac{1 - \sqrt{1 - 4a_0 a_2}}{2a_2} \\ &= \frac{4a_0 a_2}{2a_2(1 + \sqrt{1 - 4a_0 a_2})} \\ &\leq C_{\mathrm{fast},\tau} \frac{\cdot \mathrm{polylog}\,(n)}{\sqrt{n}}\end{aligned}$$

for some constant $C_{\mathrm{fast},\tau}$ independent of $n$.

The proof of Lemma 43 is then complete.

### C.9 Proof of Lemma 44

Fix $\tau = 10$. Denote

$$\begin{aligned}\Delta_{n,\rho} &= \sup_{x \in B_\mu(L)} \left\| \widehat{Q}_{n,\rho}(x) - Q^*(x) \right\|_{\mathrm{F}} \\ \widetilde{E}_\rho &= \left\{ \sup_{x \in B_\mu(L)} \left\| \widehat{Q}_{n,\rho}(x) \right\|_{\mathrm{op}} \leq n^2 \widetilde{M}_L \right\},\end{aligned}$$

where $\widetilde{M}_L = (C_B \vee c_b)^2 L^2 L_\tau^{4+(4\vee 2C_1)} \geq t_0^2$ is defined in Lemma 36. By definition, one has $\widetilde{E}_2 \subset \widetilde{E}_0 \subset \widetilde{E}_\rho$. Hence one can obtain the following decomposition

$$\begin{aligned}\mathbb{E}\Delta_{n,\rho} &= \mathbb{E}\Delta_{n,\rho} \mathbf{1}\left\{\widetilde{E}_2\right\} + \mathbb{E}\Delta_{n,\rho} \mathbf{1}\left\{\widetilde{E}_2^c \cap \widetilde{E}_\rho\right\} + \mathbb{E}\Delta_{n,\rho} \mathbf{1}\left\{\widetilde{E}_\rho^c\right\} \\ &\leq C_{\mathrm{fast},\tau} \frac{\mathrm{polylog}\,(n)}{\sqrt{n}} + + \mathbb{E}\Delta_{n,\rho} \mathbf{1}\left\{\widetilde{E}_2^c \cap \widetilde{E}_\rho\right\} + \mathbb{E}\Delta_{n,\rho} \mathbf{1}\left\{\widetilde{E}_\rho^c\right\}\end{aligned}$$

- Under event $\widetilde{E}_2^c \cap \widetilde{E}_\rho$, one has

$$\begin{aligned}\Delta_{n,\rho} &\lesssim \sup_{x \in B_\mu(L)} \left\| \widehat{Q}_\rho(x) - Q^*(x) \right\|_{\mathrm{op}} \\ &\leq \sup_{x \in B_\mu(L)} \left[ \left\| \widehat{Q}_\rho(x) \right\|_{\mathrm{op}} \vee \left\| Q^*(x) \right\|_{\mathrm{op}} \right] \\ &\overset{(i)}{\lesssim} n^2 \widetilde{M}_L\end{aligned}$$

  Here (i) follows from Lemma 33, 36 and the definition of $\widetilde{E}_\rho$. Hence

$$\begin{aligned}\mathbb{E}\Delta_{n,\rho} \mathbf{1}\left\{\widetilde{E}_2^c \cap \widetilde{E}_\rho\right\} &\lesssim n^2 \widetilde{M}_L \mathbb{P}(\widetilde{E}_2^c) \\ &\lesssim \frac{\mathrm{polylog}\,(n)}{n^{\tau-2}}\end{aligned}$$

- Under event $\widetilde{E}_\rho^c$, one has

$$\begin{aligned}\Delta_{n,\rho} &\lesssim \sup_{x\in B_\mu(L)} \left[\left\|\widehat{Q}_\rho(x)\right\|_{\mathrm{op}} + \|Q^*(x)\|_{\mathrm{op}}\right] \\ &\lesssim \sup_{x\in B_\mu(L)} \left\|\widehat{Q}_\rho(x)\right\|_{\mathrm{op}}\end{aligned}$$

Lemma 36 implies that under $\widetilde{E}_\rho^c$, for any $t \geq n^2\widetilde{M}_L$,

$$\begin{aligned}&\mathbb{P}\left\{\sup_{x\in B_\mu(L)} \left\|\widehat{Q}_\rho(x)\right\|_{\mathrm{op}} \geq t\right\} \\ &\lesssim h_0(\sqrt{t}/n) \\ &\lesssim n\cdot\exp\left[-\left(\frac{\sqrt{t}}{c_b L n}\right)^{2/(C_\Lambda\vee 2+2)}\right] \\ &= n\cdot\exp\left[-\left(\frac{t}{c_b^2 L^2 n^2}\right)^{1/(C_\Lambda\vee 2+2)}\right]\end{aligned}$$

Hence

$$\begin{aligned}\mathbb{E}\Delta_{n,\rho}\mathbf{1}\left\{\widetilde{E}_\rho^c\right\} &\lesssim \mathbb{E}\sup_{x\in B_\mu(L)} \left\|\widehat{Q}_\rho(x)\right\|_{\mathrm{op}} \mathbf{1}\left\{\sup_{x\in B_\mu(L)} \left\|\widehat{Q}_\rho(x)\right\|_{\mathrm{op}} > n^2\widetilde{M}_L\right\} \\ &\lesssim \int_{n^2\widetilde{M}_L}^\infty nt\exp\left[-\left(\frac{t}{c_b^2L^2n^2}\right)^{1/(C_\Lambda\vee 2+2)}\right]dt \\ &= \int_{\widetilde{M}_L}^\infty n^5 t\exp\left[-\left(\frac{t}{c_b^2L^2}\right)^{1/(C_\Lambda\vee 2+2)}\right]dt \\ &\overset{(i)}{\lesssim} \int_{\widetilde{M}_L}^\infty n^5 t\left\{\exp\left[-\left(\frac{\widetilde{M}_L}{c_b^2L^2}\right)^{1/(C_\Lambda\vee 2+2)} - \left(\frac{t}{c_b^2L^2}\right)^{1/(C_\Lambda\vee 2+2)}\right]\right\}dt \\ &\lesssim \int_{\widetilde{M}_L}^\infty n^5 t\left\{n^{-(1+\tau)}\exp\left[-\left(\frac{t}{c_b^2L^2}\right)^{1/(C_\Lambda\vee 2+2)}\right]\right\}dt \\ &\lesssim n^{4-\tau}L^4\int_{\widetilde{M}_L/(c_bL^2)}^\infty t\exp\left[-t^{1/(C_\Lambda\vee 2+2)}\right]dt \\ &\lesssim n^{4-\tau}L^4\end{aligned}$$

Here (i) follows from the inequality

$$(a+b)^\alpha \geq (a^\alpha + b^\alpha)/2^{1-\alpha}, \quad a,b\geq 0, 0<\alpha\leq 1$$

Finally, it follows by combining the results above that for $\tau \geq 9/2$,

$$\mathbb{E}\Delta_{n,\rho} \leq \frac{\mathrm{polylog}\,(n)}{\sqrt{n}}$$

This finishes the proof of Lemma 44.

### C.10 Proof of Lemma 45

Let $\tau > 9/2$. Lemma 21 **(iv)** implies that under $\widetilde{E}_2$, one also has

$$\sup_{x \in B_\mu(L)} W\left(\widehat{Q}_\rho(x), Q^*(x)\right) \leq \frac{\operatorname{polylog}(n)}{\sqrt{n}}$$

The proof then follows similar steps to those in Lemma 44. Specifically, denote

$$\widetilde{\Delta}_{n,\rho} = \sup_{x \in B_\mu(L)} W\left(\widehat{Q}_\rho(x), Q^*(x)\right)$$

Then one can obtain

$$\begin{aligned} \mathbb{E}\widetilde{\Delta}_{n,\rho} &= \mathbb{E}\widetilde{\Delta}_{n,\rho}\mathbf{1}\left\{\widetilde{E}_2\right\} + \mathbb{E}\widetilde{\Delta}_{n,\rho}\mathbf{1}\left\{\widetilde{E}_2^c \cap \widetilde{E}_\rho\right\} + \mathbb{E}\widetilde{\Delta}_{n,\rho}\mathbf{1}\left\{\widetilde{E}_\rho^c\right\} \\ &\leq \frac{\operatorname{polylog}(n)}{\sqrt{n}} + + \mathbb{E}\widetilde{\Delta}_{n,\rho}\mathbf{1}\left\{\widetilde{E}_2^c \cap \widetilde{E}_\rho\right\} + \mathbb{E}\widetilde{\Delta}_{n,\rho}\mathbf{1}\left\{\widetilde{E}_\rho^c\right\} \end{aligned}$$

- Under event $\widetilde{E}_2^c \cap \widetilde{E}_\rho$, Lemma 21 **(i)** implies that

$$\begin{aligned} \widetilde{\Delta}_{n,\rho} &\lesssim \sup_{x \in B_\mu(L)} \left[\left\|\widehat{Q}_\rho(x)\right\|_{\mathrm{op}} \vee \left\|Q^*(x)\right\|_{\mathrm{op}}\right] \\ &\overset{(i)}{\lesssim} n^2 \widetilde{M}_L \end{aligned}$$

  Here (i) follows from Lemma 33, 36 and the definition of $\widetilde{E}_\rho$. Hence

$$\begin{aligned} \mathbb{E}\widetilde{\Delta}_{n,\rho}\mathbf{1}\left\{\widetilde{E}_2^c \cap \widetilde{E}_\rho\right\} &\leq n^2 \widetilde{M}_L \mathbb{P}(\widetilde{E}_2^c) \\ &\leq \frac{\operatorname{polylog}(n)}{n^{\tau-2}} \end{aligned}$$

- Under event $\widetilde{E}_\rho^c$, one can similarly obtain that

$$\widetilde{\Delta}_{n,\rho} \lesssim \sup_{x \in B_\mu(L)} \left\|\widehat{Q}_\rho(x)\right\|_{\mathrm{op}}$$

  and

$$\begin{aligned} \mathbb{E}\widetilde{\Delta}_{n,\rho}\mathbf{1}\left\{\widetilde{E}_\rho^c\right\} &\lesssim \mathbb{E} \sup_{x \in B_\mu(L)} \left\|\widehat{Q}_\rho(x)\right\|_{\mathrm{op}} \mathbf{1}\left\{\sup_{x \in B_\mu(L)} \left\|\widehat{Q}_\rho(x)\right\|_{\mathrm{op}} > n^2 \widetilde{M}_L\right\} \\ &\lesssim n^{-(\tau-4)} \end{aligned}$$

Finally, one can arrive at the conclusion that for $\tau \geq 9/2$,

$$\mathbb{E}\widetilde{\Delta}_{n,\rho} \leq \frac{\operatorname{polylog}(n)}{\sqrt{n}}$$

This finishes the proof of Lemma 45.

## Appendix D. Proof of Theorem 11

To simplify notation, we fix $x$ and write $Q^*$ for $Q^*(x)$, $\widehat{Q}_{n,\rho}$ for $\widehat{Q}_{n,\rho}(x)$ when there is no ambiguity.

Following the same argument as (149) in Appendix C, one can obtain

$$\mathbb{E}_{(X,Q)\sim\mathbb{P}}\ w(x,X)\left(T_{Q^*}^{Q} - I_d\right) = 0 \tag{151}$$

$$\begin{aligned}\sqrt{n}\left(\widehat{Q}_{n,\rho}(x) - Q^*(x)\right) &= \Bigg(\underbrace{-\frac{1}{n}\sum_{i=1}^{n} w_{n,\rho}(x,X_i)dT_{Q^*(x)}^{Q_i}}_{=\widehat{\Phi}_\rho(x)}\Bigg)^{-1} \cdot \underbrace{\frac{1}{\sqrt{n}}\sum_{i=1}^{n} w_{n,\rho}(x,X_i)\left(T_{Q^*(x)}^{Q_i} - I_d\right)}_{=:a_n(x)} \\ &+ \left(-\frac{1}{n}\sum_{i=1}^{n} w_{n,\rho}(x,X_i)dT_{Q^*(x)}^{Q_i}\right)^{-1} \cdot \underbrace{\frac{\sqrt{n}}{2}\cdot\frac{1}{n}\sum_{i=1}^{n} w_{n,\rho}(x,X_i)d^2T_{\widetilde{Q}_n(x)}^{Q_i}\cdot(\widehat{Q}_{n,\rho}(x) - Q^*(x))^{\otimes 2}}_{=:b_n(x)}\end{aligned} \tag{152}$$

**Analysis of $\widehat{\Phi}_\rho(x)$:** Lemma 40 and 41 together imply that for any fixed $x$,

$$\widehat{\Phi}_\rho(x) \xrightarrow{p} \mathbb{E}\left[-w(x,X)dT_{Q^*(x)}^{Q}\right]$$

Then by Assumption 5, one has

$$\left[\widehat{\Phi}_\rho(x)\right]^{-1} \xrightarrow{p} \left[\mathbb{E}\left(-w(x,X)dT_{Q^*(x)}^{Q}\right)\right]^{-1} \tag{153}$$

**Analysis of $b_n(x)$:** Theorem 7, Lemma 40 and 41 together imply that for any fixed $x$, with probability at least $1 - O(n^{-100})$,

$$\begin{aligned}|b_n(x)| &\le \frac{\sqrt{n}}{2}\cdot\left\|\left|\frac{1}{n}\sum_{i=1}^{n} w_{n,\rho}(x,X_i)d^2T_{\widetilde{Q}_n(x)}^{Q_i}\right|\right\|\cdot\left\|\widehat{Q}_{n,\rho}(x) - Q^*(x)\right\|_{\mathrm{F}}^2 \\ &\le \frac{\sqrt{n}}{2}\cdot\mathrm{polylog}\,(n)\cdot\frac{\mathrm{polylog}\,(n)}{n} \\ &= \frac{\mathrm{polylog}\,(n)}{\sqrt{n}}\end{aligned} \tag{154}$$

**Analysis of $a_n(x)$:** by Lemma 26, one has $w(x,X) = \vec{x}^\top \vec{\Sigma}^{-1}\vec{X}$ and $w_{n,\rho}(x,X) = \vec{x}^\top \widehat{\vec{\Sigma}}_\rho^{-1}\vec{X}$. Hence one can obtain

$$\begin{aligned}
a_n(x) &= \frac{1}{\sqrt{n}}\sum_{i=1}^n \vec{x}^\top \widehat{\vec{\Sigma}}_\rho^{-1}\vec{X}_i\left(T_{Q^*}^{Q_i} - I_d\right) \\
&= \left(\vec{x}^\top \widehat{\vec{\Sigma}}_\rho^{-1} \otimes I_d\right)\cdot \frac{1}{\sqrt{n}}\sum_{i=1}^n \vec{X}_i \otimes \left(T_{Q^*}^{Q_i} - I_d\right) \\
&= \underbrace{\left(\vec{x}^\top \vec{\Sigma}^{-1} \otimes I_d\right)\cdot \frac{1}{\sqrt{n}}\sum_{i=1}^n \vec{X}_i \otimes \left(T_{Q^*}^{Q_i} - I_d\right)}_{=:a_{n,1}(x)} \\
&\quad + \underbrace{\sqrt{n}\left(\vec{x}^\top(\widehat{\vec{\Sigma}}_\rho^{-1} - \vec{\Sigma}^{-1}) \otimes I_d\right)\cdot \mathbb{E}\vec{X} \otimes \left(T_{Q^*}^{Q} - I_d\right)}_{=:a_{n,2}(x)} \\
&\quad + \underbrace{\sqrt{n}\left(\vec{x}^\top(\widehat{\vec{\Sigma}}_\rho^{-1} - \vec{\Sigma}^{-1}) \otimes I_d\right)\cdot \left[\frac{1}{n}\sum_{i=1}^n \vec{X}_i \otimes \left(T_{Q^*}^{Q_i} - I_d\right) - \mathbb{E}\vec{X} \otimes \left(T_{Q^*}^{Q} - I_d\right)\right]}_{=:a_{n,3}(x)}
\end{aligned} \tag{155}$$

- $a_{n,1}(x)$: by (151) and Lemma 26, one can obtain

$$\mathbb{E}a_{n,1}(x) = 0 \tag{156}$$

To see this, it suffices to show $\mathbb{E}\left(\vec{x}^\top \vec{\Sigma}^{-1} \otimes I_d\right)\cdot\left(\vec{X} \otimes (T_{Q^*}^{Q} - I_d)\right) = 0$. Direct computation shows that the LHS of the equality is equal to

$$\mathbb{E}\vec{x}^\top \vec{\Sigma}^{-1}\vec{X}\left(T_{Q^*}^{Q} - I_d\right) = \mathbb{E}w(x,X)(T_{Q^*}^{Q} - I_d) = 0$$

Here the equality follows from the optimality condition (151).

- $a_{n,2}(x)$: using the formula $A^{-1} - B^{-1} = A^{-1}(B-A)B^{-1}$, one can obtain

$$\begin{aligned}
a_{n,2}(x) &= \sqrt{n}\left(\vec{x}^\top \widehat{\vec{\Sigma}}_\rho^{-1}(\vec{\Sigma} - \widehat{\vec{\Sigma}}_\rho^{-1})\vec{\Sigma}^{-1} \otimes I_d\right)\cdot \mathbb{E}\vec{X} \otimes \left(T_{Q^*}^{Q} - I_d\right) \\
&= \sqrt{n}\left(\vec{x}^\top \vec{\Sigma}^{-1}(\vec{\Sigma} - \widehat{\vec{\Sigma}}_\rho)\vec{\Sigma}^{-1} \otimes I_d\right)\cdot \mathbb{E}\vec{X} \otimes \left(T_{Q^*}^{Q} - I_d\right) \\
&\quad - \sqrt{n}\left(\vec{x}^\top(\vec{\Sigma}^{-1} - \widehat{\vec{\Sigma}}_\rho^{-1})(\vec{\Sigma} - \widehat{\vec{\Sigma}}_\rho^{-1})\vec{\Sigma}^{-1} \otimes I_d\right)\cdot \mathbb{E}\vec{X} \otimes \left(T_{Q^*}^{Q} - I_d\right) \\
&= -\frac{1}{\sqrt{n}}\left(\sum_{i=1}^n \vec{x}^\top \vec{\Sigma}^{-1}(\vec{X}_i\vec{X}_i^\top - \vec{\Sigma})\vec{\Sigma}^{-1} \otimes I_d\right)\cdot\left(\mathbb{E}\vec{X} \otimes (T_{Q^*}^{Q} - I_d)\right) + O_p\left(\frac{1}{\sqrt{n}}\right)
\end{aligned} \tag{157}$$

Here the last line follows from the fact that $\widehat{\vec{\Sigma}}_\rho - \vec{\Sigma} = O_p(n^{-1/2})$ which is itself due to the sub-Gaussianity of $X$.

- $a_{n,3}(x)$: note that $\left[\frac{1}{n}\sum_{i=1}^n \vec{X}_i \otimes \left(T_{Q^*}^{Q_i} - I_d\right) - \mathbb{E}\vec{X} \otimes \left(T_{Q^*}^{Q} - I_d\right)\right] = O_p(n^{-1/2})$ again by the sub-Gaussianity of $X$ and Assumption 2, one can arrive at

$$a_{n,3}(x) = O_p(n^{-1/2}) \tag{158}$$

Combining (155), (156), (157), (158) and the functional central limit theorem (Hsing and Eubank, 2015, Theorem 7.7.6), one can obtain

$$\begin{aligned} a_n(x) = \frac{1}{\sqrt{n}}\sum_{i=1}^n \Big[ &\left(\vec{x}^\top \vec{\Sigma}^{-1} \otimes I_d\right) \cdot \left(\vec{X}_i \otimes (T_{Q^*}^{Q_i} - I_d)\right) - \\ &\left(\vec{x}^\top \vec{\Sigma}^{-1}(\vec{X}_i\vec{X}_i^\top - \vec{\Sigma}) \otimes I_d\right) \cdot \left(\mathbb{E}\vec{X} \otimes (T_{Q^*}^{Q} - I_d)\right) \Big] + o_p(1) \\ \xrightarrow{w} Z_x & \end{aligned} \tag{159}$$

where $Z_x \sim \mathcal{N}(0, \Xi_x)$ is a Gaussian random matrix with mean 0 and covariance $\Xi_x$. Here $\Xi_x := \mathbb{E}V_x \otimes V_x$ with

$$V_x = w(x, X)\left(T_{Q^*}^{Q} - I_d\right) - \left(\vec{x}^\top \vec{\Sigma}^{-1}(\vec{X}\vec{X}^\top - \vec{\Sigma}) \otimes I_d\right) \cdot \left(\mathbb{E}\vec{X} \otimes (T_{Q^*}^{Q} - I_d)\right)$$

Finally, (152), (153), (154) and (159) together imply

$$\sqrt{n}\left(\widehat{Q}_{n,\rho} - Q^*\right) \xrightarrow{w} \left(-\mathbb{E}w(x, X) dT_{Q^*}^{Q}\right)^{-1} \cdot Z_x$$

which completes the proof.

## Appendix E. Proof of Corollary 14

The optimality condition for $Q^*(x)$ implies that

$$\mathbb{E}w(x, X)\left(T_{Q^*(x)}^{Q} - I_d\right) = 0$$

Note that independence between $X$ and $Q$ implies that

$$\begin{aligned} \mathbb{E}w(x, X)\left(T_{Q^*(x)}^{Q} - I_d\right) &= \mathbb{E}w(x, X)\mathbb{E}\left(T_{Q^*(x)}^{Q} - I_d\right) \\ &\overset{(i)}{=} \mathbb{E}\left(T_{Q^*(x)}^{Q} - I_d\right) \end{aligned}$$

Here (i) follows from the fact that $\mathbb{E}w(x, X) = 1$. Therefore, one has

$$\mathbb{E}\left(T_{Q^*(x)}^{Q} - I_d\right) = 0$$

By independence, the above equality then implies

$$\mathbb{E}\vec{X} \otimes (T_{Q^*}^{Q} - I_d) = 0$$

Hence one has $V_{x,2} = 0$, the proof is then complete.

## Appendix F. Proof of Theorem 16

First, we demonstrate in Lemma 46 below uniform fast convergence under Assumption 1, 2, 5, 6 and the null hypothesis. In order to apply Theorem 7, it suffices to verify Assumption 4 and Assumption 5. Note that Assumption 6 and the null hypothesis implies the independence between $X$ and $Q$, then both Assumption 4 and 5 are consequences of Lemma 34.

**Lemma 46** *Instate the assumptions in Theorem 16. Then with probability at least* $1 - O(n^{-100})$*, one has*

$$\sup_{x \in B_\mu(L_n)} \left\| \widehat{Q}_\rho(x) - Q^*(x) \right\|_{\mathrm{F}} \leq \frac{\operatorname{polylog}(n)}{\sqrt{n}}$$

$$\sup_{1 \leq i \leq n} \left\| \widehat{Q}_\rho(X_i) - Q^*(X_i) \right\|_{\mathrm{F}} \leq \frac{\operatorname{polylog}(n)}{\sqrt{n}}$$

With Lemma 46 in place, we consider the Taylor expansion of the optimality condition for $\widehat{Q}_\rho(x)$. Recall that the optimality condition for $\widehat{Q}_\rho(x)$ gives

$$\frac{1}{n} \sum_{i=1}^{n} w_{n,\rho}(x, X_i) \left( T^{Q_i}_{\widehat{Q}_\rho(x)} - I_d \right) = 0$$

Then one can apply Lemma 21 to get the following 2nd order Taylor expansion around $Q^*(x)$.

$$0 = \frac{1}{n} \sum_{i=1}^{n} w_{n,\rho}(x, X_i) \left( T^{Q_i}_{Q^*(x)} - I_d \right) + \frac{1}{n} \sum_{i=1}^{n} w_{n,\rho}(x, X_i) dT^{Q_i}_{Q^*(x)} \cdot \left( \widehat{Q}_\rho(x) - Q^*(x) \right) + R_2(x) \tag{160}$$

where $R_2(x)$ is the 2nd order remainder term with some $\widetilde{Q}_n(x)$ lying between $Q^*(x)$ and $\widehat{Q}_\rho(x)$ defined as follows.

$$R_2(x) := \frac{1}{2n} \sum_{i=1}^{n} w_{n,\rho}(x, X_i) d^2 T^{Q_i}_{\widetilde{Q}_n(x)} \cdot \left( \widehat{Q}_\rho(x) - Q^*(x) \right)^{\otimes 2}$$

Under the null hypothesis (21), one has $Q^*(x) \equiv Q^*$ and (160) reduces to

$$0 = \frac{1}{n} \sum_{i=1}^{n} w_{n,\rho}(x, X_i) \left( T^{Q_i}_{Q^*} - I_d \right) + \frac{1}{n} \sum_{i=1}^{n} w_{n,\rho}(x, X_i) dT^{Q_i}_{Q^*} \cdot \left( \widehat{Q}_\rho(x) - Q^*(x) \right) + R_2(x) \tag{161}$$

Setting $x = \overline{X}$ in (161) then gives

$$0 = \frac{1}{n} \sum_{i=1}^{n} \left( T^{Q_i}_{Q^*} - I_d \right) + \frac{1}{n} \sum_{i=1}^{n} dT^{Q_i}_{Q^*} \cdot \left( \widehat{Q}_\rho(x) - Q^*(x) \right) + R_2(x) \tag{162}$$

Take difference between (161) and (162) and rearrange, one can obtain

$$\begin{aligned}
&\underbrace{\left(-\frac{1}{n}\sum_{i=1}^{n} dT_{Q^*}^{Q_i}\right)\cdot(\widehat{Q}_\rho(x)-\widehat{Q}_\rho(\overline{X}))}_{=:\tau(x)} \\
=&\underbrace{\frac{1}{n}\sum_{i=1}^{n}(w_{n,\rho}(x,X_i)-1)(T_{Q^*}^{Q_i}-I_d)}_{=:\alpha_0(x)}+\underbrace{\frac{1}{n}\sum_{i=1}^{n}(w_{n,\rho}(x,X_i)-1)dT_{Q^*}^{Q_i}\cdot(\widehat{Q}_\rho(x)-\widehat{Q}_\rho(\overline{X}))}_{=:\alpha_1(x)} \\
&+\underbrace{R_2(x)-R_2(\overline{X})}_{=:\alpha_2(x)}
\end{aligned} \tag{163}$$

Before delving further into the proof, we pause to introduce more notations. Define

$$\widehat{\tau}(x)=\left(-\frac{1}{n}\sum_{i=1}^{n} dT_{\widehat{Q}_\rho(\overline{X})}^{Q_i}\right)\cdot(\widehat{Q}_\rho(x)-\widehat{Q}_\rho(\overline{X}))$$

Then by definition, the test statistic $\widehat{\mathcal{T}}_\rho=\sum_{i=1}^n\|\widehat{\tau}(X_i)\|_{\mathrm{F}}^2$, and we have the following decomposition of $\tau(x)$.

$$\begin{aligned}
\widehat{\tau}(x)&=\tau(x)+\widehat{\tau}(x)-\tau(x)\\
&=\tau(x)+\underbrace{\left(\frac{1}{n}\sum_{i=1}^{n} dT_{Q^*}^{Q_i}-\frac{1}{n}\sum_{i=1}^{n} dT_{\widehat{Q}_\rho(\overline{X})}^{Q_i}\right)\cdot(\widehat{Q}_\rho(x)-\widehat{Q}_\rho(\overline{X}))}_{=:\alpha_3(x)}\\
&=\alpha_0(x)+\underbrace{\alpha_1(x)+\alpha_2(x)+\alpha_3(x)}_{=:R(x)}
\end{aligned}$$

We also define counterparts of $\alpha_0,...,\alpha_2$ and $R_2$ by replacing $w_{n,\rho}(\cdot,\cdot)$ with $w(\cdot,\cdot)$ as follows.

$$\begin{aligned}
\widetilde{\alpha}_0(x)&:=\frac{1}{n}\sum_{i=1}^{n}(w(x,X_i)-1)(T_{Q^*}^{Q_i}-I_d)\\
\widetilde{\alpha}_1(x)&:=\frac{1}{n}\sum_{i=1}^{n}(w(x,X_i)-1)dT_{Q^*}^{Q_i}\cdot(\widehat{Q}_\rho(x)-\widehat{Q}_\rho(\overline{X}))\\
\widetilde{\alpha}_2(x)&:=\widetilde{R}_2(x)-\widetilde{R}_2(\overline{X})
\end{aligned}$$

where

$$\widetilde{R}_2(x)=\frac{1}{2n}\sum_{i=1}^{n} w(x,X_i)d^2T_{\widetilde{Q}_n(x)}^{Q_i}\cdot(\widehat{Q}_\rho(x)-Q^*)^{\otimes 2}$$

With the above notation in place, one can obtain

$$\widehat{\mathcal{T}}_\rho=\sum_{k=1}^{n}\|\alpha_0(X_k)\|_{\mathrm{F}}^2+\underbrace{2\sum_{k=1}^{n}\langle\alpha_0(X_k),R(X_k)\rangle+\sum_{k=1}^{n}\|R(X_k)\|_{\mathrm{F}}^2}_{\mathrm{Rem}_n} \tag{164}$$

The proof is then divided into three steps.

- First, we give upper bounds for $\widetilde{\alpha}_i$ as well as $\widetilde{\alpha}_i - \alpha_i$ and their consequences.
- Next, we show that the remainder term $\mathrm{Rem}_n$ is negligible.
- Then, we demonstrate that $\sum_i \|\alpha_0(X_i)\|_{\mathrm{F}}^2$ converges weakly to the desired asymptotic null distribution (24).

**Analysis of $\widetilde{\alpha}$ and $\widetilde{\alpha} - \alpha$:** We give uniform upper bounds for $\widetilde{\alpha}_i(x) - \alpha_i(x)$ for $i = 0, 1, 2$ in Lemma 47 as uniform upper bounds for $\widetilde{\alpha}_i(x)$ and $\alpha_3(x)$ in Lemma 48; see Appendix F.1, F.2 for the proof.

**Lemma 47** *Instate the notations and assumptions in Theorem 16.*

$$\sup_{x \in B_\mu(L)} \|\alpha_0(x) - \widetilde{\alpha}_0(x)\|_F \leq \frac{\mathrm{polylog}\,(n)}{\sqrt{n}} \tag{165}$$

$$\sup_{x \in B_\mu(L)} \|\alpha_1(x) - \widetilde{\alpha}_1(x)\|_F \leq \frac{\mathrm{polylog}\,(n)}{n} \tag{166}$$

$$\sup_{x \in B_\mu(L)} \|\alpha_2(x) - \widetilde{\alpha}_2(x)\|_F \leq \frac{\mathrm{polylog}\,(n)}{n^{3/2}} \tag{167}$$

*with probability at least* $1 - O\left(n^{-99}\right)$.

**Lemma 48** *Instate the notations and assumptions in Theorem 16.*

$$\sup_{x \in B_\mu(L)} \|\widetilde{\alpha}_0(x)\|_F \leq \frac{\mathrm{polylog}\,(n)}{\sqrt{n}} \tag{168}$$

$$\sup_{x \in B_\mu(L)} \|\widetilde{\alpha}_1(x)\|_F \leq \frac{\mathrm{polylog}\,(n)}{n} \tag{169}$$

$$\sup_{x \in B_\mu(L)} \|\widetilde{\alpha}_2(x)\|_F \leq \frac{\mathrm{polylog}\,(n)}{n} \tag{170}$$

$$\sup_{x \in B_\mu(L)} \|\alpha_3(x)\|_F \leq \frac{\mathrm{polylog}\,(n)}{n} \tag{171}$$

*with probability at least* $1 - O\left(n^{-99}\right)$.

With the above lemmas in place, one can readily obtain that with probability at least $1 - O(n^{-99})$,

$$\sup_{i \in [n]} \|\alpha_0(X_i)\|_{\mathrm{F}} \leq \sup_{i \in [n]} \|\widetilde{\alpha}_0(X_i) - \alpha_0(X_i)\|_{\mathrm{F}} + \sup_{i \in [n]} \|\widetilde{\alpha}_0(X_i)\|_{\mathrm{F}} \leq \frac{\mathrm{polylog}\,(n)}{\sqrt{n}} \tag{172}$$

$$\sup_{i \in [n]} \|\alpha_1(X_i)\|_{\mathrm{F}} \leq \sup_{i \in [n]} \|\widetilde{\alpha}_1(X_i) - \alpha_1(X_i)\|_{\mathrm{F}} + \sup_{i \in [n]} \|\widetilde{\alpha}_1(X_i)\|_{\mathrm{F}} \leq \frac{\mathrm{polylog}\,(n)}{n} \tag{173}$$

$$\sup_{i \in [n]} \|\alpha_2(X_i)\|_{\mathrm{F}} \leq \sup_{i \in [n]} \|\widetilde{\alpha}_2(X_i) - \alpha_2(X_i)\|_{\mathrm{F}} + \sup_{i \in [n]} \|\widetilde{\alpha}_2(X_i)\|_{\mathrm{F}} \leq \frac{\mathrm{polylog}\,(n)}{n} \tag{174}$$

which then implies

$$\sup_{i\in[n]} \|R(X_i)\|_{\mathrm{F}} \leq \sum_{k=1}^{3} \sup_{i\in[n]} \|\alpha_k(X_i)\|_{\mathrm{F}} \leq \frac{\operatorname{polylog}(n)}{n} \tag{175}$$

**Negligibility of $\mathrm{Rem}_n$:** We consider two terms $\sum_{k=1}^{n} \langle \alpha_0(X_k), R(X_k)\rangle$ and $\sum_{k=1}^{n} \|R(X_k)\|_{\mathrm{F}}^2$ separately.

*Analysis of $\sum_{k=1}^{n} \langle \alpha_0(X_k), R(X_k)\rangle$:* By (172) and (175), one has with probability at least $1 - O(n^{-99})$,

$$\begin{aligned}\sum_{k=1}^{n} \langle \alpha_0(X_k), R(X_k)\rangle &\leq \sum_{k=1}^{n} \|\alpha_0(X_k)\|_{\mathrm{F}} \cdot \|R(X_k)\|_{\mathrm{F}} \\ &\leq \sum_{k=1}^{n} \frac{\operatorname{polylog}(n)}{n^{3/2}} \\ &= \frac{\operatorname{polylog}(n)}{\sqrt{n}}\end{aligned}$$

*Analysis of $\sum_{k=1}^{n} \|R(X_k)\|_{\mathrm{F}}^2$:* Similarly, by (175), one has with probability at least $1 - O(n^{-99})$,

$$\sum_{k=1}^{n} \|R(X_k)\|_{\mathrm{F}}^2 \leq \frac{\operatorname{polylog}(n)}{n}$$

Therefore, the above results imply that with probability at least $1 - O(n^{-99})$,

$$\mathrm{Rem}_n \leq \frac{\operatorname{polylog}(n)}{\sqrt{n}} \tag{176}$$

**Analysis of $\alpha_0$:** To consider the main term $\sum_{i=1}^{n} \|\alpha_0(X_i)\|_{\mathrm{F}}^2$, one has

$$\begin{aligned}&\sum_{k=1}^{n} \|\alpha_0(X_k)\|_{\mathrm{F}}^2 \\ =&\frac{1}{n^2} \sum_{k=1}^{n} \left\langle \sum_{i=1}^{n} (X_k - \overline{X})^\top \widehat{\Sigma}_\rho^{-1} (X_i - \overline{X})(T_{Q^*}^{Q_i} - I_d), \sum_{j=1}^{n} (X_k - \overline{X})^\top \widehat{\Sigma}_\rho^{-1} (X_j - \overline{X})(T_{Q^*}^{Q_j} - I_d) \right\rangle \\ =&\frac{1}{n^2} \sum_{i,j=1}^{n} \sum_{k=1}^{n} (X_i - \overline{X})^\top \widehat{\Sigma}_\rho^{-1} (X_k - \overline{X})(X_k - \overline{X})^\top \widehat{\Sigma}_\rho^{-1} (X_j - \overline{X}) \left\langle T_{Q^*}^{Q_i} - I_d, T_{Q^*}^{Q_j} - I_d \right\rangle \\ =&\frac{1}{n} \sum_{i,j=1}^{n} (X_i - \overline{X})^\top \widehat{\Sigma}_\rho^{-1} (X_j - \overline{X}) \left\langle T_{Q^*}^{Q_i} - I_d, T_{Q^*}^{Q_j} - I_d \right\rangle \\ =&\left\| \frac{1}{\sqrt{n}} \sum_{i=1}^{n} \widehat{\Sigma}_\rho^{-1/2} (X_i - \overline{X}) \otimes (T_{Q^*}^{Q_i} - I_d) \right\|_{\mathrm{F}}^2\end{aligned}$$

Note that

$$\begin{aligned}
&\frac{1}{\sqrt{n}}\sum_{i=1}^{n}\widehat{\Sigma}_{\rho}^{-1/2}(X_i-\overline{X})\otimes(T_{Q^*}^{Q_i}-I_d)\\
&=\left[(\widehat{\Sigma}_{\rho}^{-1/2}\Sigma^{1/2})\otimes I_d\right]\cdot\left[\frac{1}{\sqrt{n}}\sum_{i=1}^{n}\Sigma^{-1/2}(X_i-\overline{X})\otimes(T_{Q^*}^{Q_i}-I_d)\right]\\
&=(1+o_p(1))\frac{1}{\sqrt{n}}\sum_{i=1}^{n}\Sigma^{-1/2}(X_i-\overline{X})\otimes(T_{Q^*}^{Q_i}-I_d) \qquad (177)
\end{aligned}$$

Also, one can obtain

$$\begin{aligned}
&\left(\frac{1}{\sqrt{n}}\sum_{i=1}^{n}\Sigma^{-1/2}(X_i-\overline{X})\otimes(T_{Q^*}^{Q_i}-I_d)\right)-\left(\frac{1}{\sqrt{n}}\sum_{i=1}^{n}\Sigma^{-1/2}(X_i-\mu)\otimes(T_{Q^*}^{Q_i}-I_d)\right)\\
=&\frac{1}{\sqrt{n}}\sum_{i=1}^{n}\Sigma^{-1/2}(\mu-\overline{X})\otimes(T_{Q^*}^{Q_i}-I_d)\\
=&\Sigma^{-1/2}(\mu-\overline{X})\otimes\left[\frac{1}{\sqrt{n}}\sum_{i=1}^{n}\left(T_{Q^*}^{Q_i}-I_d\right)\right]\\
=&o_p(1) \qquad (178)
\end{aligned}$$

Here the last line follows since $\overline{X}-\mu=o_p(1)$ and $\frac{1}{\sqrt{n}}\sum_{i=1}^{n}(T_{Q^*}^{Q_i}-I_d)$ is asymptotically normal with zero mean. The zero mean is justified in the following claim whose proof is deferred to Appendix F.3.

**Claim 4** *Under the null hypothesis (21) and Assumption 4, 6, $X$ and $Q$ are independent and one has*

$$\mathbb{E}(X-\mu)\otimes\left(T_{Q^*}^{Q}-I_d\right)=0 \qquad (179)$$

$$\mathbb{E}\left(T_{Q^*}^{Q}-I_d\right)=0 \qquad (180)$$

Claim 4 also implies that $\mathbb{E}\Sigma^{-1/2}(X-\mu)\otimes(T_{Q^*}^{Q_i}-I_d)=0$. Therefore, by the functional central limit theorem (Hsing and Eubank, 2015, Theorem 7.7.6), one can obtain

$$\frac{1}{\sqrt{n}}\sum_{i=1}^{n}\Sigma^{-1/2}(X_i-\mu)\otimes(T_{Q^*}^{Q_i}-I_d)\xrightarrow{w}\mathcal{N}\left(0,I_p\otimes\mathbb{E}\left[(T_{Q^*}^{Q}-I_d)\otimes(T_{Q^*}^{Q}-I_d)\right]\right) \qquad (181)$$

Combining (177), (178) and (181), we arrive at

$$\frac{1}{\sqrt{n}}\sum_{i=1}^{n}\widehat{\Sigma}_{\rho}^{-1/2}(X_i-\overline{X})\otimes(T_{Q^*}^{Q_i}-I_d)\xrightarrow{w}\mathcal{N}\left(0,I_p\otimes\mathbb{E}\left[(T_{Q^*}^{Q}-I_d)\otimes(T_{Q^*}^{Q}-I_d)\right]\right)$$

which then implies that

$$\sum_{k=1}^{n}\|\alpha_0(X_k)\|^2\xrightarrow{w}\sum_i\lambda_i w_i \qquad (182)$$

where $w_i$ are i.i.d. $\chi_p^2$ random variables and $\lambda_i$ are the eigenvalues of $\mathbb{E}\left[(T_{Q_*}^{Q} - I_d) \otimes (T_{Q_*}^{Q} - I_d)\right]$.

Finally, taking (164) (176) and (182) collectively yields

$$\widehat{\mathcal{T}}_\rho \xrightarrow{w} \sum_i \lambda_i w_i$$

### F.1 Proof of Lemma 47

Apply Lemma 46 and triangle inequality to see that under the null (21), with probability at least $1 - O(n^{-100})$, one has

$$\begin{aligned}
&\sup_{x \in B_\mu(L)} \left\| \widehat{Q}_\rho(x) - Q^* \right\|_{\mathrm{F}} \le \frac{\text{polylog}\,(n)}{\sqrt{n}} \\
&\sup_{x \in B_\mu(L)} \left\| \widehat{Q}_\rho(x) - \widehat{Q}_\rho(\overline{X}) \right\|_{\mathrm{F}} \le \sup_{x \in B_\mu(L)} \left\| \widehat{Q}_\rho(x) - Q^* \right\|_{\mathrm{F}} + \sup_{x \in B_\mu(L)} \left\| \widehat{Q}_\rho(\overline{X}) - Q^* \right\|_{\mathrm{F}} \le \frac{\text{polylog}\,(n)}{\sqrt{n}}
\end{aligned} \tag{183}$$

**Proof of (165):** note that

$$\begin{aligned}
\alpha_0(x) - \widetilde{\alpha}_0(x) &= \frac{1}{n} \sum_{i=1}^n \left(w_{n,\rho}(x, X_i) - w(x, X_i)\right) (T_{Q^*}^{Q_i} - I_d) \\
&= A_n(x) - \widetilde{A}_n(x)
\end{aligned}$$

Hence (165) follows from Lemma 41.

**Proof of (166):** one can obtain

$$\begin{aligned}
\|\alpha_1(x) - \widetilde{\alpha}_1(x)\|_{\mathrm{F}} &= \left\| \frac{1}{n} \sum_{i=1}^n (w_{n,\rho}(x, X_i) - w(x, X_i)) dT_{Q^*}^{Q_i} \cdot (\widehat{Q}_\rho(x) - \widehat{Q}_\rho(\overline{X})) \right\|_{\mathrm{F}} \\
&\le \left\|\left\| \frac{1}{n} \sum_{i=1}^n (w_{n,\rho}(x, X_i) - w(x, X_i)) dT_{Q^*}^{Q_i} \right\|\right\| \cdot \left\| \widehat{Q}_\rho(x) - \widehat{Q}_\rho(\overline{X}) \right\|_{\mathrm{F}} \\
&= \left\|\left\| \Phi_n(x) - \widetilde{\Phi}_n(x) \right\|\right\| \cdot \left\| \widehat{Q}_\rho(x) - \widehat{Q}_\rho(\overline{X}) \right\|_{\mathrm{F}}
\end{aligned}$$

Hence (166) follows from Lemma 41 and (183).

**Proof of (167):** one can obtain

$$\begin{aligned}
\|\alpha_2(x) - \widetilde{\alpha}_2(x)\|_{\mathrm{F}} &= \left\| \frac{1}{n} \sum_{i=1}^n (w_{n,\rho}(x, X_i) - w(x, X_i)) d^2 T_{\widetilde{Q}_n(x)}^{Q_i} \cdot (\widehat{Q}_\rho(x) - Q^*)^{\otimes 2} \right\|_{\mathrm{F}} \\
&\le \left\|\left\| \frac{1}{n} \sum_{i=1}^n (w_{n,\rho}(x, X_i) - w(x, X_i)) d^2 T_{\widetilde{Q}_n(x)}^{Q_i} \right\|\right\| \cdot \left\| \widehat{Q}_\rho(x) - \widehat{Q}_\rho(\overline{X}) \right\|_{\mathrm{F}}^2 \\
&= \left\|\left\| \Psi_n(x, \widetilde{Q}_n(x)) - \widetilde{\Psi}_n(x, \widetilde{Q}_n(x)) \right\|\right\| \cdot \left\| \widehat{Q}_\rho(x) - \widehat{Q}_\rho(\overline{X}) \right\|_{\mathrm{F}}^2
\end{aligned}$$

Hence (167) follows from Lemma 41 and (183).

### F.2 Proof of Lemma 48

Apply Lemma 46 and triangle inequality to see that under the null (21), with probability at least $1 - O(n^{-100})$, one has

$$\sup_{x \in B_\mu(L)} \left\| \widehat{Q}_\rho(x) - Q^* \right\|_{\mathrm{F}} \leq \frac{\operatorname{polylog}(n)}{\sqrt{n}}$$

$$\sup_{x \in B_\mu(L)} \left\| \widehat{Q}_\rho(x) - \widehat{Q}_\rho(\overline{X}) \right\|_{\mathrm{F}} \leq \sup_{x \in B_\mu(L)} \left\| \widehat{Q}_\rho(x) - Q^* \right\|_{\mathrm{F}} + \sup_{x \in B_\mu(L)} \left\| \widehat{Q}_\rho(\overline{X}) - Q^* \right\|_{\mathrm{F}} \leq \frac{\operatorname{polylog}(n)}{\sqrt{n}} \tag{184}$$

**Proof of (168):** Recall the definition of $\widetilde{A}_n(x)$ in (77), one can see that (168) follows from (83) in Lemma 40 with a slight and straightforward modification to accommodate the $w(x, X) - 1$ term here. For brevity, we omit the proof.

**Proof of (169):**

$$\|\widetilde{\alpha}_1\|_{\mathrm{F}} \leq \left\|\left\| \frac{1}{n} \sum_{i=1}^{n} (w(x, X_i) - 1) dT_{Q^*}^{Q_i} \right\|\right\| \cdot \left\| \widehat{Q}_\rho(x) - \widehat{Q}_\rho(\overline{X}) \right\|_{\mathrm{F}} \tag{185}$$

Recall the definition of $\widetilde{\Phi}_n(x)$ in (77), with a slight modification of the proof of (84) in Lemma 40, one can obtain

$$\sup_{x \in B_\mu(L)} \left\|\left\| \frac{1}{n} \sum_{i=1}^{n} (w(x, X_i) - 1) dT_{Q^*}^{Q_i} \right\|\right\| \leq \frac{\operatorname{polylog}(n)}{\sqrt{n}} \tag{186}$$

For brevity, the proof is omitted.

Combining (184), (185) and (186) gives (169).

**Proof of (170):** Apply triangle inequality to see that

$$\begin{aligned} \sup_{x \in B_\mu(L)} \|\widetilde{\alpha}_2(x)\|_{\mathrm{F}} &\leq \left\| \widetilde{R}_2(\overline{X}) \right\|_{\mathrm{F}} + \sup_{x \in B_\mu(L)} \left\| \widetilde{R}_2(x) \right\|_{\mathrm{F}} \\ &\leq 2 \sup_{x \in B_\mu(L)} \left\| \widetilde{R}_2(x) \right\|_{\mathrm{F}} \end{aligned}$$

Moreover, one can obtain

$$\begin{aligned} \sup_{x \in B_\mu(L)} \left\| \widetilde{R}_2(x) \right\|_{\mathrm{F}} &\lesssim \sup_{x \in B_\mu(L)} \left\|\left\| \frac{1}{n} \sum_{i=1}^{n} w(x, X_i) d^2 T_{\widetilde{Q}_n(x)}^{Q_i} \right\|\right\| \cdot \left\| \widehat{Q}_\rho(x) - Q^* \right\|_{\mathrm{F}}^2 \\ &\leq \sup_{\substack{x \in B_\mu(L) \\ S \in \mathcal{S}_d((2M_L)^{-1}, 2M_L)}} \left\|\left\| \widetilde{\Psi}_n(x, S) \right\|\right\| \cdot \sup_{x \in B_\mu(L)} \left\| \widehat{Q}_\rho(x) - Q^* \right\|_{\mathrm{F}}^2 \\ &\overset{(i)}{\leq} \frac{\operatorname{polylog}(n)}{n} \end{aligned}$$

Here (i) follows from (85), (86) in Lemma 40 as well as (184).

**Proof of (171):** one can obtain

$$\sup_{x\in B_\mu(L)} \|\alpha_3(x)\|_{\mathrm{F}} = \sup_{x\in B_\mu(L)} \left\| \left( \frac{1}{n}\sum_{i=1}^n dT_{Q^*}^{Q_i} - \frac{1}{n}\sum_{i=1}^n dT_{\widehat{Q}_\rho(\overline{X})}^{Q_i} \right) \cdot (\widehat{Q}_\rho(x) - \widehat{Q}_\rho(\overline{X})) \right\|_{\mathrm{F}}$$
$$\leq \left\|\left| \frac{1}{n}\sum_{i=1}^n dT_{Q^*}^{Q_i} - \frac{1}{n}\sum_{i=1}^n dT_{\widehat{Q}_\rho(\overline{X})}^{Q_i} \right\|\right| \cdot \sup_{x\in B_\mu(L)} \left\| \widehat{Q}_\rho(x) - \widehat{Q}_\rho(\overline{X}) \right\|_{\mathrm{F}} \tag{187}$$

Moreover, one has

$$\left\|\left| \frac{1}{n}\sum_{i=1}^n dT_{Q^*}^{Q_i} - \frac{1}{n}\sum_{i=1}^n dT_{\widehat{Q}_\rho(\overline{X})}^{Q_i} \right\|\right| \overset{(i)}{\leq} \frac{1}{n}\sum_{i=1}^n \left\|\left| dT_{Q^*}^{Q_i} - dT_{\widehat{Q}_\rho(\overline{X})}^{Q_i} \right\|\right|$$
$$\overset{(ii)}{\leq} \frac{1}{n}\sum_{i=1}^n \left\|\left| d^2T_{Q'_{i,n}}^{Q_i} \cdot (\widehat{Q}_\rho(\overline{X}) - Q^*) \right\|\right|$$
$$\overset{(iii)}{\leq} \frac{1}{n}\sum_{i=1}^n \left\|\left| d^2T_{Q'_{i,n}}^{Q_i} \right\|\right| \cdot \left\| \widehat{Q}_\rho(\overline{X}) - Q^* \right\|_{\mathrm{F}} \tag{188}$$

Here (i) follows from triangle inequality, (ii) from the mean value theorem (Dudley and Norvaiša, 2011, Theorem 5.3) for some $Q'_{i,n}$ that lies on the segment between $Q^*$ and $\widehat{Q}_\rho(\overline{X})$, and (iii) arises from Lemma 22. Therefore, with a slight modification of the proof of (86) in Lemma 40, one can obtain with probability at least $1 - O(n^{-100})$,

$$\frac{1}{n}\sum_{i=1}^n \left\|\left| d^2T_{Q'_{i,n}}^{Q_i} \right\|\right| \leq \mathrm{polylog}\,(n) \tag{189}$$

For brevity, the proof is omitted.

Finally, combining (187), (188) and (189) gives

$$\sup_{x\in B_\mu(L)} \|\alpha_3(x)\|_{\mathrm{F}} \lesssim \left[ \frac{1}{n}\sum_{i=1}^n \left\|\left| d^2T_{Q'_{i,n}}^{Q_i} \right\|\right| \right] \cdot \left[ \sup_{x\in B_\mu(L)} \left\| \widehat{Q}_\rho(x) - Q^*(x) \right\|_{\mathrm{F}} \right]^2$$
$$\leq \frac{\mathrm{polylog}\,(n)}{n}$$

which finishes the proof for (171).

### F.3 Proof of Claim 4

The independence follows directly from Assumption 6 and the null hypothesis (21).

**Proof of (179):** by independence, we have

$$\mathbb{E}(X-\mu) \otimes \left( T_{Q^*}^{Q} - I_d \right) = [\mathbb{E}(X-\mu)] \otimes \left[ \mathbb{E}\left( T_{Q^*}^{Q} - I_d \right) \right]$$
$$= 0$$

**Proof of (180):** The optimality condition of $Q^*(x)$ gives that

$$\mathbb{E}w(x,X)\left(T^{Q}_{Q^*(x)} - I_d\right) = 0$$

By independence, one then has

$$\begin{aligned}\mathbb{E}\left(T^{Q}_{Q^*(x)} - I_d\right) &\overset{(i)}{=} [\mathbb{E}w(x,X)]\cdot\left[\mathbb{E}w(x,X)\left(T^{Q}_{Q^*(x)} - I_d\right)\right]\\ &= \mathbb{E}w(x,X)\left(T^{Q}_{Q^*(x)} - I_d\right)\\ &= 0\end{aligned}$$

Here (i) follows from the fact that $\mathbb{E}w(x,X) \equiv 1$.

## Appendix G. Proof of Proposition 1

Note that under the null (21), it holds that $Q^*(\overline{X}) = Q^*$. Then Theorem 7 implies that with probability at least $1 - O(n^{-100})$,

$$\left\|\widehat{Q}_\rho(\overline{X}) - Q^*\right\|_{\mathrm{F}} \le \frac{\mathrm{polylog}\,(n)}{\sqrt{n}}$$

Therefore, one has with probability at least $1 - O(n^{-100})$,

$$\widehat{Q}_\rho(\overline{X}) \in \mathcal{S}_d((2c_1)^{-1}, 2c_1) \tag{190}$$

for $n$ large enough. Here we recall that $c_1 \ge 1$ is a constant defined in Assumption 2.

One can apply the triangle inequality to see that

$$\begin{aligned}&\left\|\frac{1}{n}\sum_{i=1}^n\left(T^{Q_i}_{\widehat{Q}_\rho(\overline{X})} - I_d\right)\otimes\left(T^{Q_i}_{\widehat{Q}_n(\overline{X})} - I_d\right) - \mathbb{E}\left(T^{Q}_{Q^*} - I_d\right)\otimes\left(T^{Q}_{Q^*} - I_d\right)\right\|_{\mathrm{F}}\\ &\le \left\|\frac{1}{n}\sum_{i=1}^n\left(T^{Q_i}_{\widehat{Q}_\rho(\overline{X})} - I_d\right)\otimes\left(T^{Q_i}_{\widehat{Q}_n(\overline{X})} - I_d\right) - \mathbb{E}\left(T^{Q}_{\widehat{Q}_\rho(\overline{X})} - I_d\right)\otimes\left(T^{Q}_{\widehat{Q}_\rho(\overline{X})} - I_d\right)\right\|_{\mathrm{F}}\\ &\quad + \left\|\mathbb{E}\left(T^{Q}_{\widehat{Q}_\rho(\overline{X})} - I_d\right)\otimes\left(T^{Q}_{\widehat{Q}_\rho(\overline{X})} - I_d\right) - \mathbb{E}\left(T^{Q}_{Q^*} - I_d\right)\otimes\left(T^{Q}_{Q^*} - I_d\right)\right\|_{\mathrm{F}}\\ &\overset{(i)}{\le} \underbrace{\sup_{S\in\mathcal{S}_d((2c_1)^{-1},2c_1)}\left\|\frac{1}{n}\sum_{i=1}^n\left(T^{Q_i}_{S} - I_d\right)\otimes\left(T^{Q_i}_{S} - I_d\right) - \mathbb{E}\left(T^{Q}_{S} - I_d\right)\otimes\left(T^{Q}_{S} - I_d\right)\right\|_{\mathrm{F}}}_{\zeta_1}\\ &\quad + \underbrace{\sup_{S:\|S-Q^*\|_{\mathrm{F}}\le\mathrm{polylog}(n)/\sqrt{n}}\left\|\mathbb{E}\left(T^{Q}_{S} - I_d\right)\otimes\left(T^{Q}_{S} - I_d\right) - \mathbb{E}\left(T^{Q}_{Q^*} - I_d\right)\otimes\left(T^{Q}_{Q^*} - I_d\right)\right\|_{\mathrm{F}}}_{\zeta_2}\end{aligned} \tag{191}$$

Here (i) follows from (190).

Upper bounds for $\zeta_1, \zeta_2$ in (191) are summarized in the lemma below whose proof is deferred to Appendix G.1. Note that Lemma 49 do not assume the null hypothesis so that it can be reused later for the proof of the power (Theorem 18).

**Lemma 49** *Suppose Assumption 1-6 hold. Then with probability at least* $1 - O(n^{-100})$,

$$\zeta_1 \le \frac{\mathrm{polylog}\,(n)}{\sqrt{n}} \tag{192}$$

$$\zeta_2 \le \frac{\mathrm{polylog}\,(n)}{\sqrt{n}} \tag{193}$$

Lemma 49 combined with (191) then implies

$$\left\| \frac{1}{n} \sum_{i=1}^{n} \left( T^{Q_i}_{\widehat{Q}_\rho(\overline{X})} - I_d \right) \otimes \left( T^{Q_i}_{\widehat{Q}_n(\overline{X})} - I_d \right) - \mathbb{E} \left( T^{Q}_{Q^*} - I_d \right) \otimes \left( T^{Q}_{Q^*} - I_d \right) \right\|_{\mathrm{F}} \le \frac{\mathrm{polylog}\,(n)}{\sqrt{n}} \tag{194}$$

As a result, if $(\lambda_i)_{i \in [d^2]}$ are sorted in order, then one has $\widehat{\lambda}_i \to \lambda_i$ uniformly for $i \in [d^2]$ in probability which further implies that

$$\sum_{i=1}^{d^2} \widehat{\lambda}_i w_i \xrightarrow{p} \sum_{i=1}^{d^2} \lambda_i w_i$$

Then the continuous mapping theorem implies that $\widehat{q}_{1-\alpha} \to q_{1-\alpha}$ in probability. Then one can obtain

$$\mathbb{P}\left( \widehat{\mathcal{T}}_\rho > \widehat{q}_{1-\alpha} \right) \le \mathbb{P}\left( \widehat{\mathcal{T}}_\rho > q_{1-\alpha} - \epsilon \right) + \mathbb{P}\left( |\widehat{q}_{1-\alpha} - q_{1-\alpha}| > \epsilon \right)$$

Taking the limit as $n \to \infty$, followed by letting $\epsilon \to 0$ to get that

$$\limsup_{n \to \infty} \mathbb{P}\left( \widehat{\mathcal{T}}_\rho > \widehat{q}_{1-\alpha} \right) \le \alpha \tag{195}$$

A similar lower bound shows

$$\liminf_{n \to \infty} \mathbb{P}\left( \widehat{\mathcal{T}}_\rho > \widehat{q}_{\alpha} \right) \ge \alpha \tag{196}$$

Finally, combining (195) and (196) completes the proof.

### G.1 Proof of Lemma 49

**Proof of (192):** the proof is similar to Lemma 40, and is hence omitted for brevity.

**Proof of (193):** First, recall differential properties (Lemma 21) that

$$\begin{aligned} \left\| T^Q_S \right\|_{\mathrm{op}} &\le \lambda_{\max}(Q) \cdot \lambda_{\min}(Q)^{-1/2} \cdot \lambda_{\min}(S)^{-1/2} \\ \left\| dT^Q_S \right\| &\le \frac{1}{2} \lambda_{\max}(Q)^{1/2} \cdot \lambda_{\min}(S)^{-2} \cdot \lambda_{\max}(S)^{1/2} \end{aligned} \tag{197}$$

Also, denote $\phi(Q, S) := \left( T^Q_S - I_d \right) \otimes \left( T^Q_S - I_d \right)$.

With these results in place, one can obtain

$$\begin{aligned} \|\phi(Q,S) - \phi(Q,Q^*)\|_{\mathrm{F}} &\le \left\| \left( T^Q_S - I_d \right) \otimes \left( T^Q_S - T^Q_{Q^*} \right) \right\|_{\mathrm{F}} + \left\| \left( T^Q_S - T^Q_{Q^*} \right) \otimes \left( T^Q_{Q^*} - I_d \right) \right\|_{\mathrm{F}} \\ &\le \left\| T^Q_S - I_d \right\|_{\mathrm{F}} \cdot \left\| T^Q_S - T^Q_{Q^*} \right\|_{\mathrm{F}} + \left\| T^Q_S - T^Q_{Q^*} \right\|_{\mathrm{F}} \cdot \left\| T^Q_{Q^*} - I_d \right\|_{\mathrm{F}} \\ &\le \left\| T^Q_S - I_d \right\|_{\mathrm{F}} \cdot \left\| dT^Q_{S'} \right\| \cdot \|S - Q^*\|_{\mathrm{F}} + \left\| T^Q_{Q^*} - I_d \right\|_{\mathrm{F}} \cdot \left\| dT^Q_{S'} \right\| \cdot \|S - Q^*\|_{\mathrm{F}} \end{aligned}$$

where $S'$ lies between $Q^*$ and $S$. Note that Assumption 2 and the condition $\|S - Q^*\|_{\mathrm{F}} \leq \mathrm{polylog}\,(n)/\sqrt{n}$ implies that

$$S, Q^* \in \mathcal{S}_d((2c_1)^{-1}, 2c_1)$$

Then for any $S \in \mathcal{S}_d((2c_1)^{-1}, 2c_1)$, one has

$$\begin{aligned}\mathbb{E}\left\|T_S^Q - I_d\right\|_{\mathrm{F}} \cdot \left\|\left|dT_{S'}^Q\right|\right\| &\overset{(i)}{\lesssim} \mathbb{E}\left(\|X-\mu\|^{3C_1/2} + 1\right) \cdot \|X-\mu\|^{C_1/2} \\ &\overset{(ii)}{\lesssim} 1\end{aligned}$$

Here $C_1$ is defined in Assumption 2, (i) follows from (197) and (ii) is a result of the sub-Gaussianity of $X$. Similarly, one has

$$\mathbb{E}\left\|T_{Q^*}^Q - I_d\right\|_{\mathrm{F}} \cdot \left\|\left|dT_{S'}^Q\right|\right\| \lesssim 1$$

Combining results above, one can obtain

$$\begin{aligned}\zeta_2 &\leq \sup_{S:\|S-Q^*\|_{\mathrm{F}} \leq \mathrm{polylog}(n)/\sqrt{n}} \mathbb{E}_Q \|\phi(Q,S) - \phi(Q,Q^*)\|_{\mathrm{F}} \\ &\lesssim \sup_{S:\|S-Q^*\|_{\mathrm{F}} \leq \mathrm{polylog}(n)/\sqrt{n}} 1 \cdot \|S - Q^*\|_{\mathrm{F}} \\ &\leq \frac{\mathrm{polylog}\,(n)}{\sqrt{n}}\end{aligned}$$

The proof is then complete.

## Appendix H. Proof of Theorem 18

As argued at the beginning of Appendix C.5, one can assume without loss of generality that $\|X - \mu\| \leq L$ almost surely with $L = C_L\sqrt{\log n}$ for some constant $C_L$ large enough as in (78). Recall the notation that $M_L := \gamma_\Lambda(L)$.

**Power under Frobenius norm:** First we demonstrate concentration of various quantities of interest and derive their consequences in Lemma 50 below. The proof is in Appendix H.1.

**Lemma 50** *Instate the notations and assumptions in Theorem 18. Assume in addition that $\|X - \mu\| \leq L$ almost surely. Then there exists an event $E_n$ that satisfies $\mathbb{P}(E_n) \geq 1 - O(n^{-100})$ for any $\mathbb{P} \in \mathfrak{P}$, under which the following inequalities*

$$\|X_i - \mu\| \leq L, \quad Q_i \in \mathcal{S}_d(M_L^{-1}, M_L) \qquad \textit{for} \quad i \in [n] \tag{198}$$

$$\sup_{x \in B_\mu(L)} \left\|\widehat{Q}_\rho(x) - Q^*(x)\right\|_{\mathrm{F}} \leq \frac{\mathrm{polylog}\,(n)}{\sqrt{n}} \tag{199}$$

$$\left\{\widehat{Q}_\rho(x) : x \in B_\mu(L)\right\} \subset \mathcal{S}_d\left(\left(2^{1/6}M_L\right)^{-1}, 2^{1/6}M_L\right) \tag{200}$$

$$\sum_{i=1}^n \|Q^*(X_i) - Q^*(\mu)\|_{\mathrm{F}}^2 \geq \frac{na_n^2}{2} \tag{201}$$

$$\left\|\widehat{Q}_\rho(\overline{X}) - Q^*(\mu)\right\|_{\mathrm{F}} \leq \frac{\mathrm{polylog}\,(n)}{\sqrt{n}} \tag{202}$$

$$\left|\widehat{\lambda}_i\right| \leq 2\lambda_1, \qquad i \in \left[d^2\right] \tag{203}$$

*hold for $n$ large enough.*

Next, note that ((**iii**)) in Lemma 21 implies that for any $Q, S \in \mathcal{S}_d\left(\left(2^{1/6}M_L\right)^{-1}, 2^{1/6}M_L\right)$ (here $2^{1/6}$ is chosen only for technical computation), one has

$$\frac{1}{2\sqrt{2}M_L^3} \leq \lambda_{\min}\left(-dT_S^Q\right) \leq \lambda_{\max}\left(-dT_S^Q\right) \leq \frac{\sqrt{2}}{2}M_L^3$$

which then implies that under $E_n$, the following holds.

$$\lambda_{\min}\left(\widehat{H}\right) \geq \frac{1}{n}\sum_{i=1}^n \lambda_{\min}\left(-dT^{Q_i}_{\widehat{Q}_\rho(\overline{X})}\right) \geq \frac{1}{2\sqrt{2}M_L^3}$$

Therefore, under $E_n$, one has

$$\widehat{\mathcal{T}}_\rho \geq \frac{1}{8M_L^6}\sum_{i=1}^n \left\|\widehat{Q}_\rho(X_i) - \widehat{Q}_\rho(\overline{X})\right\|_{\mathrm{F}}^2 \tag{204}$$

Then from the following decomposition

$$\widehat{Q}_\rho(X_i) - \widehat{Q}_\rho(\overline{X}) = Q^*(X_i) - Q^*(\mu) + \underbrace{\widehat{Q}_\rho(X_i) - Q^*(X_i) + Q^*(\mu) - \widehat{Q}_\rho(\overline{X})}_{\Delta_i}$$

one can obtain

$$\begin{aligned}
&\sum_{i=1}^n \left\|\widehat{Q}_\rho(X_i) - \widehat{Q}_\rho(\overline{X})\right\|_{\mathrm{F}}^2 \\
&\overset{(i)}{=} \sum_{i=1}^n \|Q^*(X_i) - Q^*(\mu)\|_{\mathrm{F}}^2 + 2\sum_{i=1}^n \langle Q^*(X_i) - Q^*(\mu), \Delta_i\rangle + \sum_{i=1}^n \|\Delta_i\|_{\mathrm{F}}^2 \\
&\overset{(ii)}{\geq} \sum_{i=1}^n \|Q^*(X_i) - Q^*(\mu)\|_{\mathrm{F}}^2 - 2\left(\sum_{i=1}^n \|Q^*(X_i) - Q^*(\mu)\|_{\mathrm{F}}^2\right)^{1/2} \cdot \left(\sum_{i=1}^n \|\Delta_i\|_{\mathrm{F}}^2\right)^{1/2} + \sum_{i=1}^n \|\Delta_i\|_{\mathrm{F}}^2 \\
&\geq \left(\sum_{i=1}^n \|Q^*(X_i) - Q^*(\mu)\|_{\mathrm{F}}^2\right) \cdot \left(1 - 2\left(\frac{\sum_{i=1}^n \|\Delta_i\|_{\mathrm{F}}^2}{\sum_{i=1}^n \|Q^*(X_i) - Q^*(\mu)\|_{\mathrm{F}}^2}\right)^{1/2}\right)
\end{aligned} \tag{205}$$

Here (i) follows by developing the square, (ii) is a consequence of the Cauchy-Schwarz inequality.

Lemma 50 implies that under $E_n$, one has

$$\|\Delta_i\|_{\mathrm{F}} \leq \left\|\widehat{Q}_\rho(X_i) - Q^*(X_i)\right\|_{\mathrm{F}} + \left\|Q^*(\mu) - \widehat{Q}_\rho(\overline{X})\right\|_{\mathrm{F}}$$

$$\lesssim \frac{\operatorname{polylog}(n)}{\sqrt{n}} \tag{206}$$

Therefore, (205) and (206) together imply the following inequality for $n$ large enough.

$$\sum_{i=1}^{n} \left\| \widehat{Q}_\rho(X_i) - \widehat{Q}_\rho(\overline{X}) \right\|_{\mathrm{F}}^2 \overset{(I)}{\geq} \frac{na_n^2}{2} \cdot \frac{3}{4} \tag{207}$$

Here (I) arises due to Lemma 50, (206) as well as the fact that $\operatorname{polylog}(n) = o\left(na_n^2\right)$.

Combining (204) and (207) then gives that under $E_n$, one has

$$\begin{aligned} \widehat{\mathcal{T}}_\rho &\geq \frac{3na_n^2}{64M_L^6} \\ &\geq \sqrt{na_n^2} \end{aligned} \tag{208}$$

for $n$ large enough.

Denote $\widetilde{q}_{1-\alpha}$ the $1-\alpha$ quantile of $\sum_{i=1}^{d^2} 2\lambda_1 w_i$, which is a fixed constant. Then as a consequence of Lemma 50, $\sum_{i=1}^{d^2} \widehat{\lambda}_i w_i$ is stochastically dominated by $\sum_{i=1}^{d^2} 2\lambda_1 w_i$ under $E_n$, which then implies that

$$\widehat{q}_{1-\alpha} < \widetilde{q}_{1-\alpha}, \qquad \text{under } E_n$$

Therefore, for any $\mathbb{P} \in \mathfrak{P}$, one can obtain

$$\begin{aligned} \mathbb{P}\left(\widehat{\mathcal{T}}_\rho > \widehat{q}_{1-\alpha}\right) &\geq \mathbb{P}\left(\left\{\widehat{\mathcal{T}}_\rho > \widetilde{q}_{1-\alpha}\right\} \cap \left\{\widetilde{q}_{1-\alpha} > \widehat{q}_{1-\alpha}\right\}\right) \\ &\geq \mathbb{P}\left(\left\{\widehat{\mathcal{T}}_\rho > \widetilde{q}_{1-\alpha}\right\} \cap E_n\right) \\ &\overset{(i)}{\geq} \mathbb{P}\left(\left\{\sqrt{na_n^2} > \widetilde{q}_{1-\alpha}\right\} \cap E_n\right) \end{aligned}$$

for $n$ large enough. Here (i) follows from (208).

Finally, one can take $n \to \infty$ to see that

$$\begin{aligned} \liminf_{n\to\infty} \inf_{\mathbb{P}\in\mathfrak{P}} \mathbb{P}\left(\widehat{\mathcal{T}}_\rho > \widehat{q}_{1-\alpha}\right) &\geq \liminf_{n\to\infty} \inf_{\mathbb{P}\in\mathfrak{P}} \mathbb{P}\left(\left\{\sqrt{na_n^2} > \widetilde{q}_{1-\alpha}\right\} \cap E_n\right) \\ &\geq 1 \end{aligned}$$

which implies

$$\lim_{n\to\infty} \inf_{\mathbb{P}\in\mathfrak{P}} \mathbb{P}\left(\widehat{\mathcal{T}}_\rho > \widehat{q}_{1-\alpha}\right) = 1$$

The proof is then complete.

**Power under Wasserstein distance:** by Lemma 21 and the boundedness assumption, one has

$$\mathbb{E}\left\|Q^*(X) - Q^*(\mu)\right\|_{\mathrm{F}}^2 \geq \frac{1}{\operatorname{polylog}(n)} \mathbb{E}W^2\left(Q^*(X), Q^*(\mu)\right)$$

Therefore, the 1st part of the proof (Frobenius norm) can be applied.

### H.1 Proof of Lemma 50

(198)-(200) are shown in the proof of Theorem 7 and (202) is due to Lemma 32.

**Proof of (201):** With these in place, we have almost surely that

$$\begin{aligned}\|Q^*(X) - Q^*(\mu)\|_{\mathrm{F}}^2 &\leq d\,\|Q^*(X) - Q^*(\mu)\|_{\mathrm{op}}^2 \\ &\leq dM_L^2 =: M_F \asymp \mathrm{polylog}\,(n) \end{aligned} \tag{209}$$

Define random variable $Y_i$ as follows.

$$Y_i := \|Q^*(X_i) - Q^*(\mu)\|_{\mathrm{F}}^2 - \mathbb{E}\,\|Q^*(X) - Q^*(\mu)\|_{\mathrm{F}}^2$$

With (209) in place, one then has $Y_i \in [-M_F, M_F]$, $\mathbb{E}Y_i = 0$ and

$$\begin{aligned}\mathrm{Var}\,Y_i &= \mathbb{E}Y_i^2 \\ &\leq \mathbb{E}\,|Y_i|\,M_F \\ &\leq 2\mathbb{E}\,\|Q^*(X_i) - Q^*(\mu)\|_{\mathrm{F}}^2 \cdot M_F \\ &= 2a_n^2 \cdot M_F\end{aligned}$$

To prove (201), it suffices to show that ,

$$\left|\sum_i^n Y_i\right| \leq \frac{na_n^2}{2} \quad \text{with probability at least } 1 - O(n^{-100}) \tag{210}$$

To this end, note that by Bernstein's inequality (Vershynin, 2018, Theorem 2.8.4), one has with probability at least $1 - O(n^{-100})$,

$$\begin{aligned}\left|\sum_i^n Y_i\right| &\lesssim M_F \log n + \sqrt{n\,\mathrm{Var}\,Y_i} \cdot \sqrt{\log n} \\ &\lesssim M_F \log n + \sqrt{na_n^2 M_F} \cdot \sqrt{\log n} \\ &\overset{(i)}{\leq} \mathrm{polylog}\,(n) \cdot \left(1 + \sqrt{na_n^2}\right)\end{aligned} \tag{211}$$

Then (211) implies (210) for $n$ large enough due to the assumption that $na_n^2 \gtrsim n^{2\alpha_2}$ for some constant $\alpha_2 > 0$. The proof is then complete.

**Proof of (203):** Note that the proof of Lemma 49 does not assume the null hypothesis. Therefore, with (202) in place, one can exactly follow (190)-(194) as in the proof of Lemma 49 to get

$$\left\|\frac{1}{n}\sum_{i=1}^n \left(T^{Q_i}_{\widehat{Q}_\rho(\overline{X})} - I_d\right) \otimes \left(T^{Q_i}_{\widehat{Q}_\rho(\overline{X})} - I_d\right) - \mathbb{E}\left(T^Q_{Q^*} - I_d\right) \otimes \left(T^Q_{Q^*} - I_d\right)\right\|_{\mathrm{F}} \leq \frac{\mathrm{polylog}\,(n)}{\sqrt{n}}$$

which implies (203).

## Appendix I. Additional simulations

We conduct simulations to compare different initialization methods and step sizes, as described in Section 5.1.

### I.1 Initialization

To assess the performance of different initialization methods, we conduct simulations based on Example 2 with parameters $n = 200$, $p = 5$ and $d = 6$, setting $\eta = 1$, $T = 30$ and $\text{eps} = 10^{-6}$ in Algorithm 1. We consider three different initialization methods:

1. Identity Initialization : $S_0 = I_d$.
2. Random Initialization: $S_0 = U \exp(M) U^\top$ where $U$ and $M$ are independent; $U \in \mathcal{O}_d$ is a random orthogonal matrix following the Haar measure, and $M$ is a random diagonal matrix with i.i.d. diagonal entries $M_{kk} \sim \mathcal{N}(0, 1/\sqrt{d})$, $k \in [d]$.
3. Mean Initialization: $S_0 = \sum_{i=1}^n Q_i$.

Table 1 reports the number of steps before termination and the relative error with respect to the identity initialization, averaged over 100 simulations. In each simulation, after generating $n = 200$ pairs of $(X_i, Q_i)$, we randomly generate $\widetilde{X} \sim \text{Uniform}[-1, 1]^p$ and compute $\widehat{Q}_\rho(\widetilde{X})$ using Algorithm 1 with the three initialization methods described above. It can be observed

Table 1: Comparison of different initialization methods.

| $\delta$ | Initialization method | Steps | Relative error ($\times$eps) |
|---|---|---|---|
| | $I_d$ | 3 | 0 |
| 0 | Random | 3.4 | 5.3e-4 |
| | Mean | 3 | 4.8e-6 |
| | $I_d$ | 3 | 0 |
| 0.2 | Random | 3.4 | 5.9e-4 |
| | Mean | 3 | 4.8e-6 |
| | $I_d$ | 3 | 0 |
| 0.4 | Random | 3.3 | 6.0e-4 |
| | Mean | 3 | 5.9e-6 |

that all three methods converge to the same point within nearly the same number of steps, as the relative error is significantly smaller than the threshold eps.

### I.2 Step size

To examine the performance of different step sizes, we conduct simulations based on Example 2 with parameters $n = 200$, $p = 5$ and $d = 6$, setting $S_0 = I_d$, $T = 30$ and $\text{eps} = 10^{-6}$ in Algorithm 1. Table 2 reports the number of steps before termination and the relative error with respect to step size 1, averaged over 100 simulations. In each simulation, after generating $n = 200$ pairs of $(X_i, Q_i)$, we randomly generate $\widetilde{X} \sim \text{Uniform}[-1, 1]^p$ and compute $\widehat{Q}_\rho(\widetilde{X})$ using Algorithm 1 with step size $\eta \in \{1, 0.9, 0.8, 0.7, 0.6\}$

Table 2: Comparison of different step sizes.

| $\delta$ | $\eta$ | Steps | Relative error ($\times$eps) |
|---|---|---|---|
| | 1 | 3 | 0 |
| | 0.9 | 8 | 0.013 |
| 0 | 0.8 | 10 | 0.13 |
| | 0.7 | 13 | 0.20 |
| | 0.6 | 17 | 0.22 |
| | 1 | 3 | 0 |
| | 0.9 | 8 | 0.013 |
| 0.2 | 0.8 | 10 | 0.13 |
| | 0.7 | 13 | 0.20 |
| | 0.6 | 17 | 0.22 |
| | 1 | 3 | 0 |
| | 0.9 | 7.94 | 0.017 |
| 0.4 | 0.8 | 10 | 0.13 |
| | 0.7 | 13 | 0.20 |
| | 0.6 | 15.97 | 0.22 |

It can be observed that all three methods converge to the same point, as the relative error is smaller than the threshold eps. Additionally, setting $\eta = 1$ results in the fastest convergence.

### I.3 Uniform convergence over a diverging domain

To investigate the convergence over a diverging domain, we slightly modify Example 2 by considering $X \sim \text{Uniform}[-\sqrt{\log n}, \sqrt{\log n}]^p$ with parameters $p = 5$, $d = 6$, $\delta = 0.1$ and $n \in \{100, 200, 400, 800, 1600\}$. For each $n$, we compute the maximum in-sample error

$$\text{maxErr}_n := \max_{i \in [n]} \left\| \widehat{Q}_\rho(X_i) - Q^*(X_i) \right\|_{\text{F}}, \quad \rho = 1/n$$

Figure 7 plots $\log \overline{\text{maxErr}_n}$ against $\log n$ where $\overline{\text{maxErr}_n}$ represents the average of $\text{maxErr}_n$ over 5 independent trials. The plot shows a linear trend with a slope $-0.46$, providing empirical support for the parametric rate of convergence up to logarithmic factors.

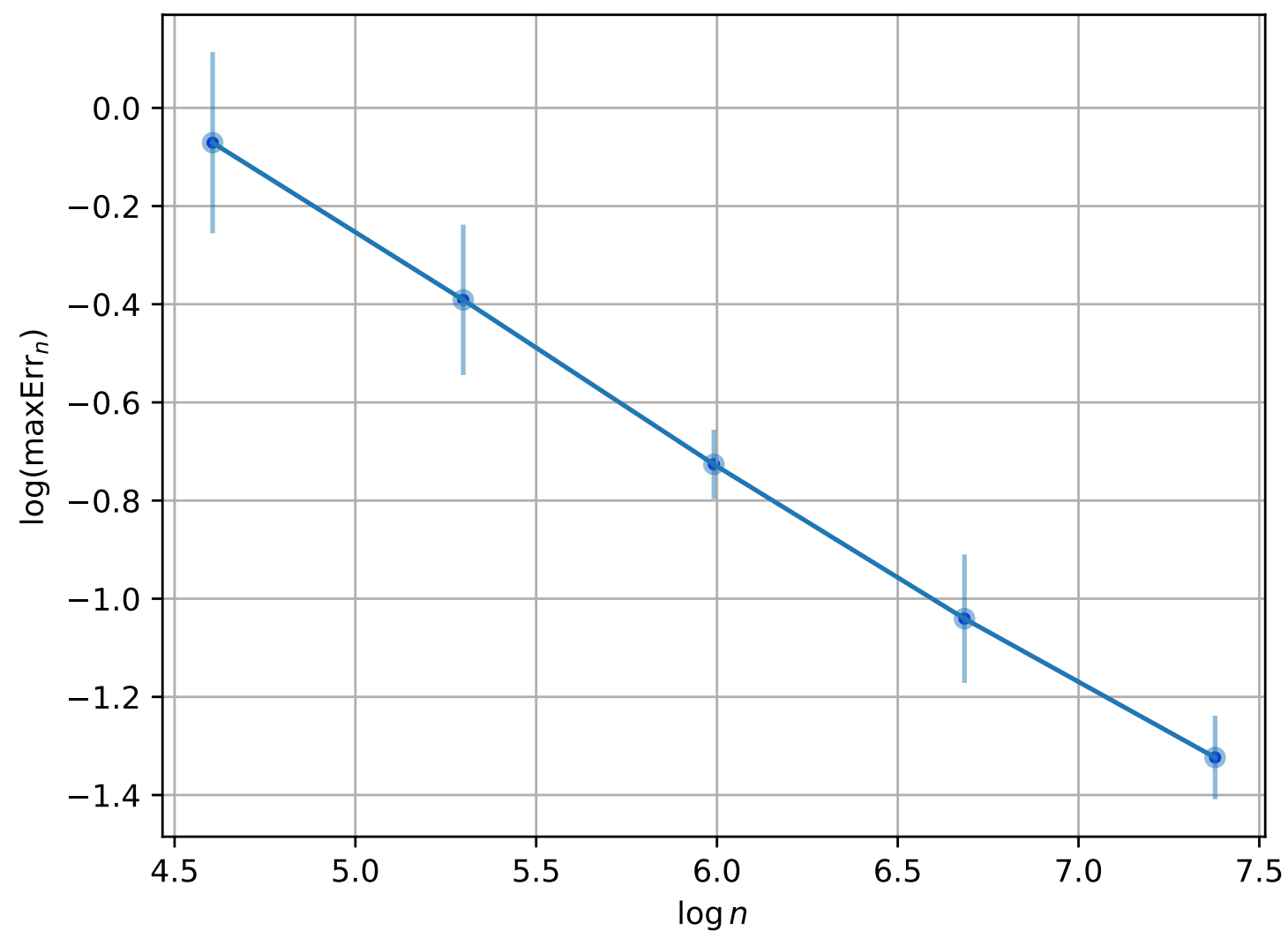


Figure 7: $\log \overline{\text{maxErr}_n}$ vs. $\log n$
